# Supplementary material for: Peptide-Bound Glycative, AGE and Oxidative Modifications as Biomarkers for the Diagnosis of Alzheimer’s Disease—A Feasibility Study
Source: Biomedicines. 2024 Sep 19;12(9):2127. doi: 10.3390/biomedicines12092127 (PMC11428617; doi:10.3390/biomedicines12092127)
Supplement: Supplementary file 1 [file biomedicines-12-02127-s001.zip › Data S1.pdf]

Sequence: RGRLKNAGGGIR, R12-Carboxymethyl (58.00548 Da), R1-Pentosyl (132.03955 Da), R3-Pentosyl (132.03955 Da)
Charge: +3, Monoisotopic m/z: 526.28229 Da (-0.81 mmu/-1.54 ppm), MH+: 1576.83231 Da, RT: 34.5034 min,
Identified with: Sequest HT (v1.17); XCorr:0.76, Percolator q-Value:7.2e-3, Percolator PEP:3.1e-2,
Fragment match tolerance used for search: 0.02 Da
Fragments used for search: -NH_3; y; b; b; -NH_3; y

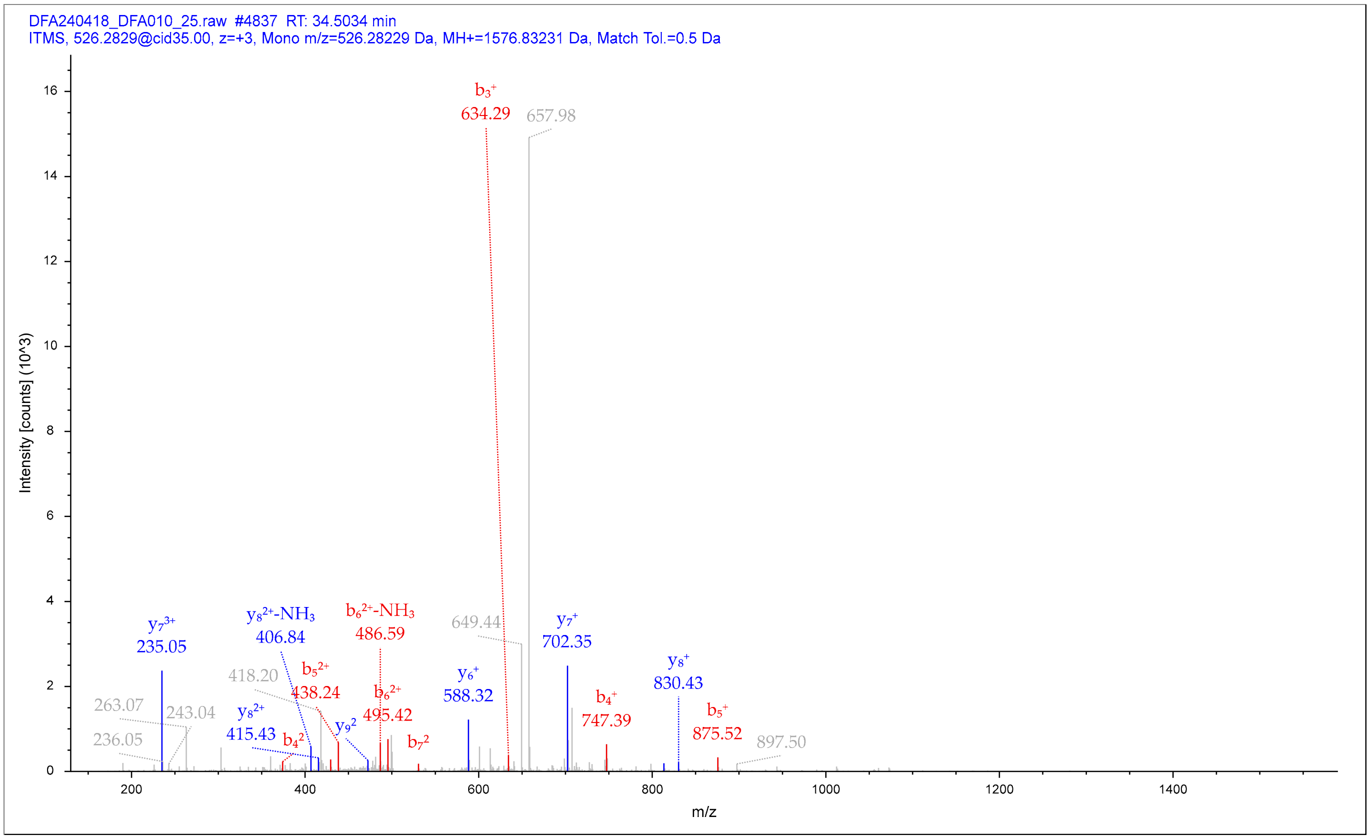

#1	b⁺	b²⁺	b³⁺	Seq.	y⁺	y²⁺	y³⁺	#2
1	289.14794	145.07761	97.05416	R-Pentosyl				12
2	346.16940	173.58834	116.06132	G	1288.69408	644.85068	430.23621	11
3	634.31006	317.65867	212.10821	R-Pentosyl	1231.67262	616.33995	411.22906	10
4	747.39413	374.20070	249.80289	L	943.53195	472.26962	315.18217	9
5	875.48909	438.24818	292.50121	K	830.44789	415.72758	277.48748	8
6	989.53202	495.26965	330.51552	N	702.35293	351.68010	234.78916	7
7	1060.56913	530.78820	354.19456	A	588.31000	294.65864	196.77485	6
8	1117.59059	559.29894	373.20172	G	517.27289	259.14008	173.09581	5
9	1174.61206	587.80967	392.20887	G	460.25142	230.62935	154.08866	4
10	1231.63352	616.32040	411.21602	G	403.22996	202.11862	135.08150	3
11	1344.71759	672.86243	448.91071	I	346.20850	173.60789	116.07435	2
12				R-Carboxymethyl	233.12443	117.06585	78.37966	1

Sequence: ILDMQQTYDMWLKK, K13-Glyoxal-imine (39.99554 Da), K14-Glyoxal-imine (39.99554 Da)
Charge: +2, Monoisotopic m/z: 946.95044 Da (+0.12 mmu/+0.12 ppm), MH+: 1892.89360 Da, RT: 53.5602 min,
Identified with: Sequest HT (v1.17); XCorr:0.52, Percolator q-Value:2.4e-3, Percolator PEP:1.8e-2,
Fragment match tolerance used for search: 0.02 Da
Fragments used for search: -H_2O; y; -NH_3; y; b; b; -H_2O; b; -NH_3; y

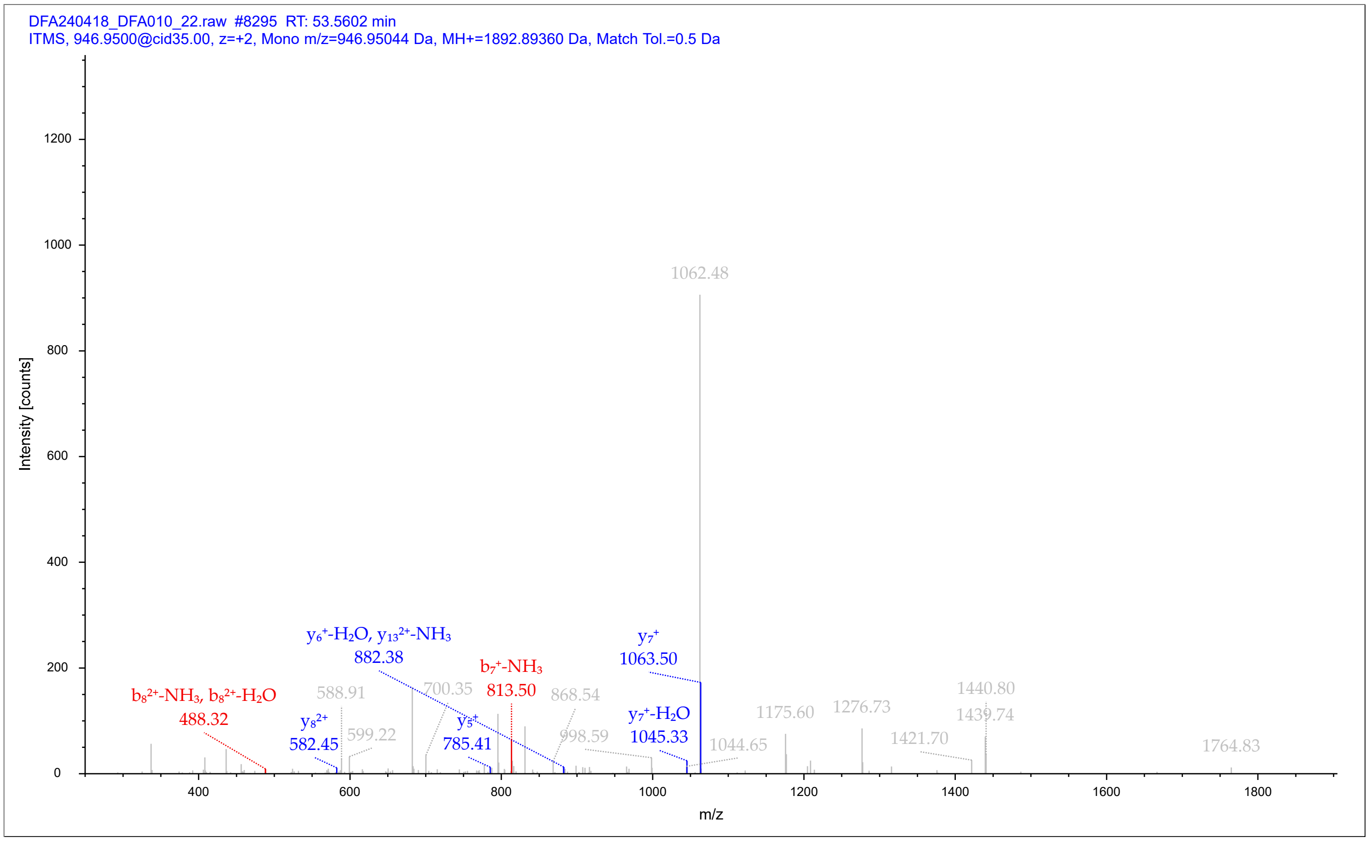

#1	b⁺	b²⁺	Seq.	y⁺	y²⁺	#2
1	114.09134	57.54931	I			14
2	227.17540	114.09134	L	1779.80930	890.40829	13
3	342.20235	171.60481	D	1666.72524	833.86626	12
4	473.24283	237.12505	M	1551.69830	776.35279	11
5	601.30141	301.15434	Q	1420.65781	710.83254	10
6	729.35999	365.18363	Q	1292.59924	646.80326	9
7	830.40767	415.70747	T	1164.54066	582.77397	8
8	993.47099	497.23914	Y	1063.49298	532.25013	7
9	1108.49794	554.75261	D	900.42965	450.71846	6
10	1239.53842	620.27285	M	785.40271	393.20499	5
11	1425.61773	713.31251	W	654.36222	327.68475	4
12	1538.70180	769.85454	L	468.28291	234.64509	3
13	1706.79230	853.89979	K-Glyoxal-imine	355.19885	178.10306	2
14			K-Glyoxal-imine	187.10834	94.05781	1

Sequence: SNLQEIFLPAFPCHER, C13-Carbamidomethyl (57.02146 Da), R16-Glarg (39.99949 Da)
Charge: +4, Monoisotopic m/z: 500.24475 Da (-0.3 mmu/-0.61 ppm), MH+: 1997.95717 Da, RT: 12.0677 min,
Identified with: Sequest HT (v1.17); XCorr:0.78, Percolator q-Value:7.1e-3, Percolator PEP:3.5e-2,
Fragment match tolerance used for search: 0.02 Da
Fragments used for search: -H_2O; y; -NH_3; y; b; b; -H_2O; b; -NH_3; y

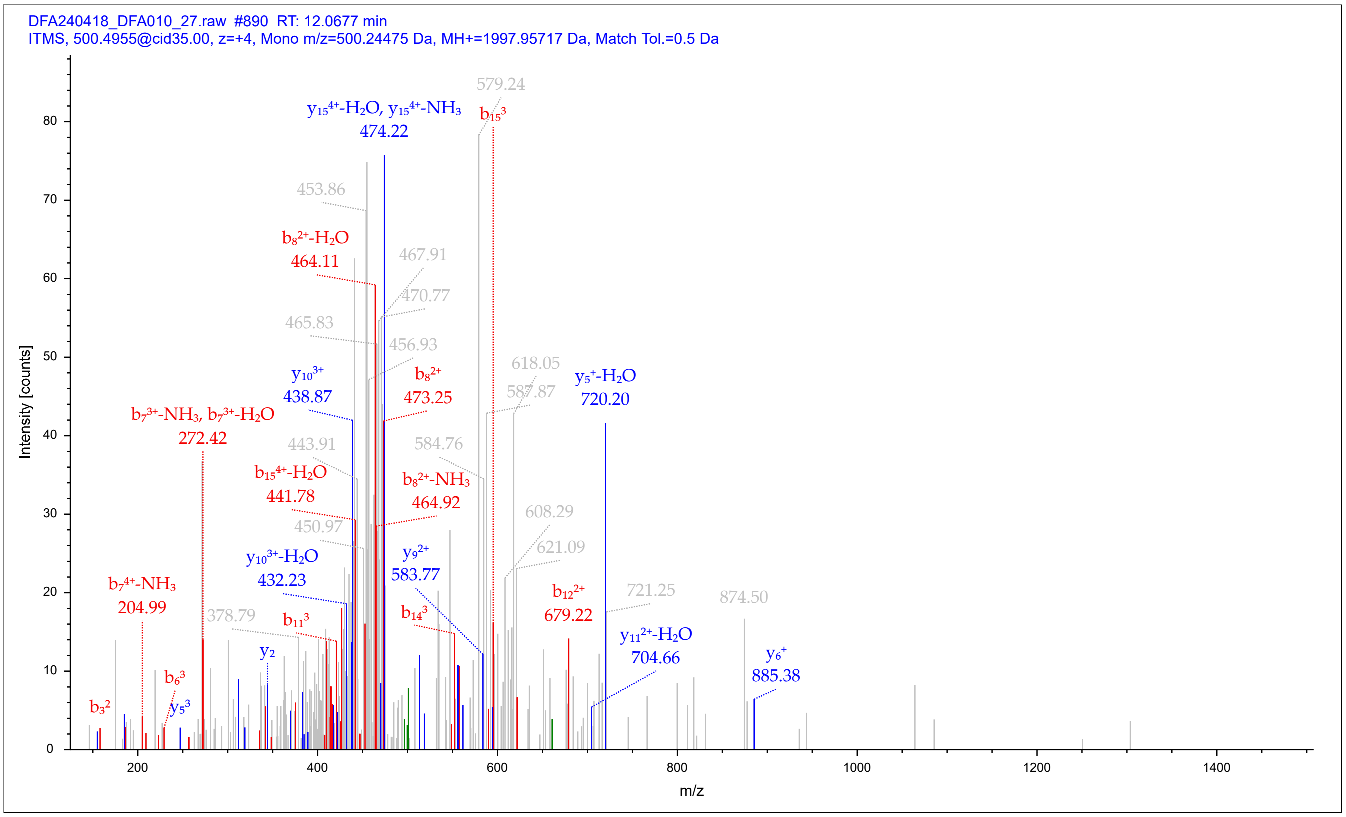

#1	b⁺	b²⁺	b³⁺	b⁴⁺	Seq.	y⁺	y²⁺	y³⁺	y⁴⁺	#2
1	88.03930	44.52329	30.01795	22.76528	S					16
2	202.08223	101.54475	68.03226	51.27602	N	1910.92636	955.96682	637.64697	478.48705	15
3	315.16630	158.08679	105.72695	79.54703	L	1796.88343	898.94535	599.63266	449.97631	14
4	443.22487	222.11608	148.41314	111.56168	Q	1683.79936	842.40332	561.93797	421.70530	13
5	572.26747	286.63737	191.42734	143.82232	E	1555.74079	778.37403	519.25178	389.69065	12
6	685.35153	343.17940	229.12203	172.09334	I	1426.69819	713.85274	476.23758	357.43001	11
7	832.41994	416.71361	278.14483	208.86044	F	1313.61413	657.31070	438.54289	329.15899	10
8	945.50401	473.25564	315.83952	237.13146	L	1166.54572	583.77650	389.52009	292.39189	9
9	1042.55677	521.78202	348.19044	261.39465	P	1053.46165	527.23446	351.82540	264.12087	8
10	1113.59389	557.30058	371.86948	279.15393	A	956.40889	478.70808	319.47448	239.85768	7
11	1260.66230	630.83479	420.89228	315.92103	F	885.37178	443.18953	295.79544	222.09840	6
12	1357.71506	679.36117	453.24321	340.18422	P	738.30336	369.65532	246.77264	185.33130	5
13	1517.74571	759.37649	506.58676	380.19189	Carbamidometl	641.25060	321.12894	214.42172	161.06811	4
14	1654.80462	827.90595	552.27306	414.45661	H	481.21995	241.11361	161.07817	121.06044	3
15	1783.84722	892.42725	595.28726	446.71726	E	344.16104	172.58416	115.39186	86.79572	2
16					R-Glarg	215.11844	108.06286	72.37767	54.53507	1

112_113

Sequence: RDPPDYWTIHGLWPDKSEGCNR, C20-Carbamidomethyl (57.02146 Da), Y6-Trioxidation (47.98474 Da), W7-Trp->Kynurenin (3.99492 Da), K16-Formyl (27.99492 Da), R1-Glarg (39.99949 Da)
Charge: +6, Monoisotopic m/z: 470.71017 Da (+1.45 mmu/+3.07 ppm), MH+: 2819.22466 Da, RT: 19.8473 min,
Identified with: Sequest HT (v1.17); XCorr:0.76, Percolator q-Value:1.5e-3, Percolator PEP:7.3e-3,
Fragment match tolerance used for search: 0.02 Da
Fragments used for search: -H_2O; y; -NH_3; y; b; b; -H_2O; b; -NH_3; y

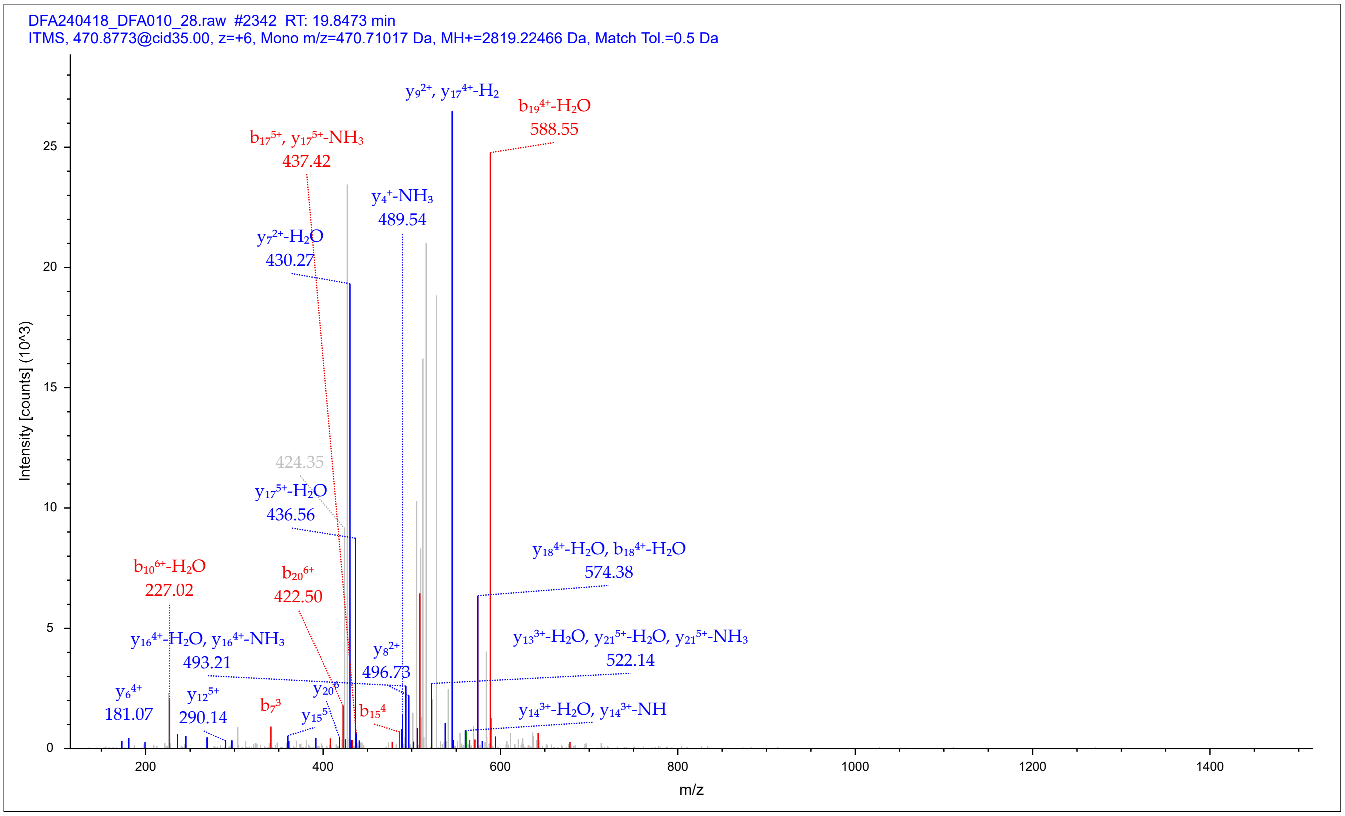

#1	b⁺	b²⁺	b³⁺	b⁴⁺	b⁵⁺	b⁶⁺	Seq.	y⁺	y²⁺	y³⁺	y⁴⁺	y⁵⁺	y⁶⁺	#2
1	197.10788	99.05758	66.37414	50.03243	40.22740	33.69071	R-Glarg							22
2	312.13482	156.57105	104.71646	78.78916	63.23279	52.86187	D	2623.11541	1312.06134	875.04332	656.53431	525.42890	438.02530	21
3	409.18759	205.09743	137.06738	103.05235	82.64334	69.03733	P	2508.08846	1254.54787	836.70101	627.77757	502.42351	418.85414	20
4	506.24035	253.62381	169.41830	127.31555	102.05389	85.21279	P	2411.03570	1206.02149	804.35008	603.51438	483.01296	402.67868	19
5	621.26729	311.13728	207.76062	156.07228	125.05928	104.38395	D	2313.98294	1157.49511	771.99916	579.25119	463.60241	386.50322	18
6	832.31537	416.66132	278.10997	208.83430	167.26889	139.55862	Y-Trioxidation	2198.95599	1099.98163	733.65685	550.49446	440.59702	367.33206	17
7	1022.38959	511.69844	341.46805	256.35286	205.28374	171.23766	W-Trp->Kynurenin	1987.90792	994.45760	663.30749	497.73244	398.38741	332.15738	16
8	1123.43727	562.22227	375.15061	281.61478	225.49328	188.07894	T	1797.83369	899.42048	599.94942	450.21388	360.37256	300.47835	15
9	1236.52134	618.76431	412.84530	309.88579	248.11009	206.92629	I	1696.78601	848.89665	566.26686	424.95196	340.16302	283.63707	14
10	1373.58025	687.29376	458.53160	344.15052	275.52187	229.76944	H	1583.70195	792.35461	528.57217	396.68095	317.54621	264.78972	13
11	1430.60171	715.80449	477.53875	358.40589	286.92616	239.27302	G	1446.64304	723.82516	482.88586	362.41622	290.13443	241.94657	12
12	1543.68578	772.34653	515.23344	386.67690	309.54298	258.12036	L	1389.62157	695.31443	463.87871	348.16085	278.73014	232.44299	11
13	1729.76509	865.38618	577.25988	433.19673	346.75884	289.13358	W	1276.53751	638.77239	426.18402	319.88984	256.11332	213.59565	10
14	1826.81785	913.91256	609.61080	457.45992	366.16939	305.30904	P	1090.45820	545.73274	364.15758	273.37001	218.89746	182.58243	9
15	1941.84479	971.42604	647.95312	486.21666	389.17478	324.48020	D	993.40543	497.20636	331.80666	249.10682	199.48691	166.40697	8
16	2097.93467	1049.47097	699.98308	525.23913	420.39276	350.49518	K-Formyl	878.37849	439.69288	293.46435	220.35008	176.48152	147.23581	7
17	2184.96670	1092.98699	728.99375	546.99713	437.79916	365.00051	S	722.28861	361.64794	241.43439	181.32761	145.26354	121.22083	6
18	2314.00929	1157.50829	772.00795	579.25778	463.60768	386.50761	E	635.25658	318.13193	212.42371	159.56960	127.85714	106.71549	5
19	2371.03076	1186.01902	791.01510	593.51315	475.01197	396.01119	G	506.21399	253.61063	169.40952	127.30896	102.04862	85.20840	4
20	2531.06141	1266.03434	844.35865	633.52081	507.01810	422.68296	C-Carbamidomethyl	449.19253	225.09990	150.40236	113.05359	90.64433	75.70482	3
21	2645.10433	1323.05581	882.37296	662.03154	529.82669	441.69012	N	289.16188	145.08458	97.05881	73.04593	58.63820	49.03304	2
22							R	175.11895	88.06311	59.04450	44.53520	35.82961	30.02589	1

Sequence: KCRPIICDKYCPLGLLK, C2-Carbamidomethyl (57.02146 Da), C7-Carbamidomethyl (57.02146 Da), C11-Carbamidomethyl (57.02146 Da), R3-Carboxymethyl (58.00548 Da), K9-GLAP (109.02805 Da), K17-GLAP (109.02805 Da)
Charge: +6, Monoisotopic m/z: 402.53790 Da (-0.65 mmu/-1.62 ppm), MH+: 2410.19103 Da, RT: 47.7813 min,
Identified with: Sequest HT (v1.17); XCorr:1.23, Percolator q-Value:4.9e-3, Percolator PEP:1.2e-2,
Fragment match tolerance used for search: 0.02 Da
Fragments used for search: -H_2O; y; b; b; -H_2O; b; -NH_3; y

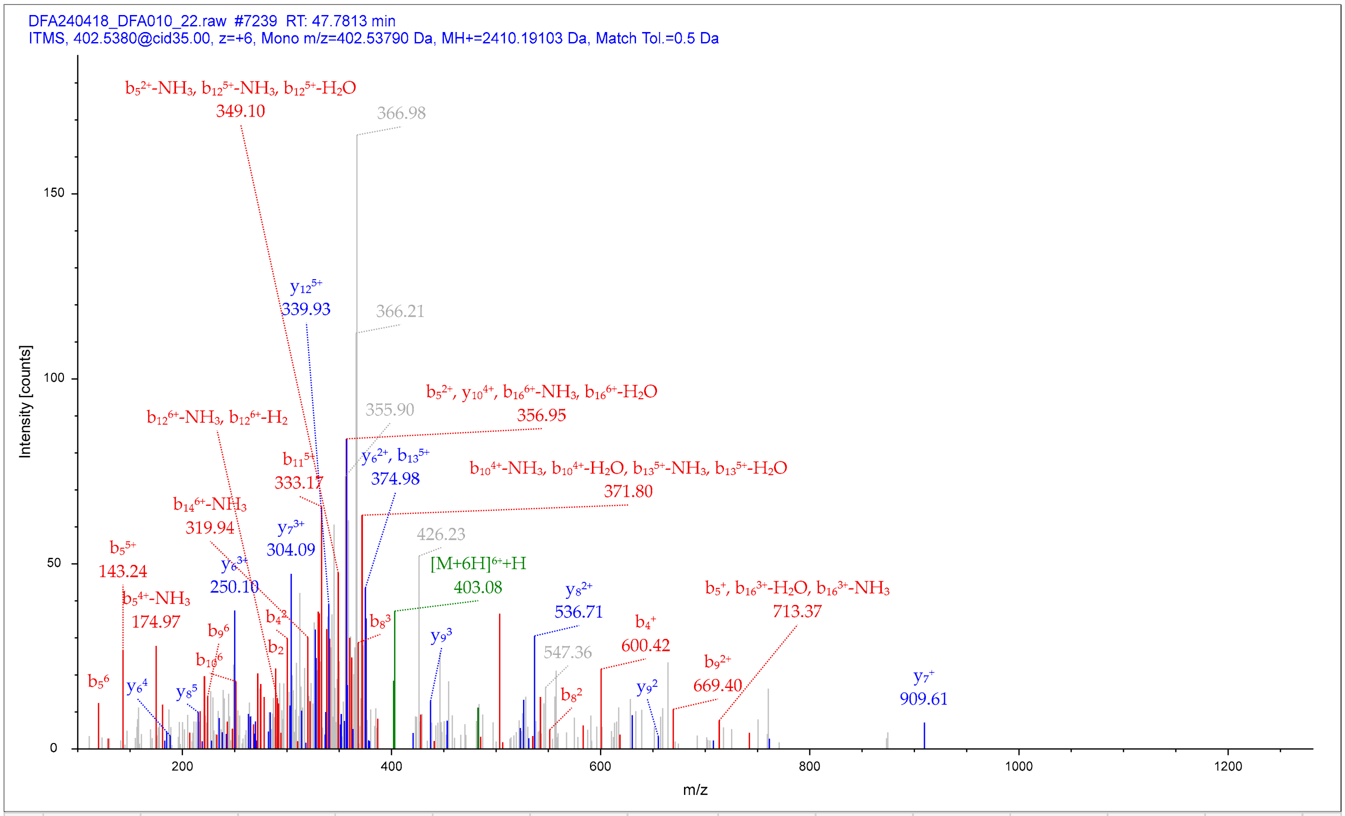

#1	b⁺	b²⁺	b³⁺	b⁴⁺	b⁵⁺	b⁶⁺	Seq.	y⁺	y²⁺	y³⁺	y⁴⁺	y⁵⁺	y⁶⁺	#2
1	129.10224	65.05476	43.70560	33.03102	26.62627	22.35644	K							17
2	289.13289	145.07008	97.04915	73.03868	58.63240	49.02821	C-Carbamido methyl	2282.09998	1141.55363	761.37151	571.28045	457.22582	381.18939	16
3	503.23948	252.12338	168.41801	126.56533	101.45372	84.71264	R-Carboxy methyl	2122.06934	1061.53831	708.02796	531.27279	425.21969	354.51762	15
4	600.29224	300.64976	200.76893	150.82852	120.86427	100.88810	P	1907.96275	954.48501	636.65910	477.74614	382.39837	318.83319	14
5	713.37631	357.19179	238.46362	179.09953	143.48108	119.73545	I	1810.90998	905.95863	604.30818	453.48295	362.98782	302.65773	13
6	826.46037	413.73382	276.15831	207.37055	166.09790	138.58279	I	1697.82592	849.41660	566.61349	425.21194	340.37101	283.81038	12
7	986.49102	493.74915	329.50186	247.37821	198.10402	165.25457	C-Carbamido methyl	1584.74185	792.87457	528.91880	396.94092	317.75419	264.96304	11
8	1101.51796	551.26262	367.84417	276.13495	221.10941	184.42572	D	1424.71121	712.85924	475.57525	356.93326	285.74806	238.29126	10
9	1338.64097	669.82413	446.88518	335.41570	268.53402	223.94623	K-GLAP	1309.68426	655.34577	437.23294	328.17652	262.74267	219.12011	9
10	1501.70430	751.35579	501.23962	376.18153	301.14668	251.12345	Y	1072.56125	536.78426	358.19193	268.89577	215.31807	179.59961	8
11	1661.73495	831.37111	554.58317	416.18920	333.15281	277.79522	C-Carbamido methyl	909.49792	455.25260	303.83749	228.12994	182.70541	152.42238	7
12	1758.78771	879.89750	586.93409	440.45239	352.56336	293.97068	P	749.46727	375.23728	250.49394	188.12228	150.69928	125.75061	6
13	1871.87178	936.43953	624.62878	468.72340	375.18018	312.81803	L	652.41451	326.71089	218.14302	163.85908	131.28872	109.57515	5
14	1928.89324	964.95026	643.63593	482.97877	386.58447	322.32160	G	539.33045	270.16886	180.44833	135.58807	108.67191	90.72780	4
15	2041.97731	1021.49229	681.33062	511.24978	409.20128	341.16895	L	482.30898	241.65813	161.44118	121.33270	97.26762	81.22423	3
16	2155.06137	1078.03432	719.02531	539.52080	431.81810	360.01629	L	369.22492	185.11610	123.74649	93.06169	74.65080	62.37688	2
17							K-GLAP	256.14085	128.57407	86.05180	64.79067	52.03399	43.52954	1

Sequence: LRLSLRNMPVVPC, C13-Carbamidomethyl (57.02146 Da), R2-Carboxyethyl (72.02113 Da)
Charge: +3, Monoisotopic m/z: 542.96558 Da (+0.12 mmu/+0.21 ppm), MH+: 1626.88218 Da, RT: 54.8216 min,
Identified with: Sequest HT (v1.17); XCorr:1.33, Percolator q-Value:6.5e-3, Percolator PEP:3.1e-2,
Fragment match tolerance used for search: 0.02 Da
Fragments used for search: -H_2O; y; -NH_3; y; b; b; -H_2O; b; -NH_3; y

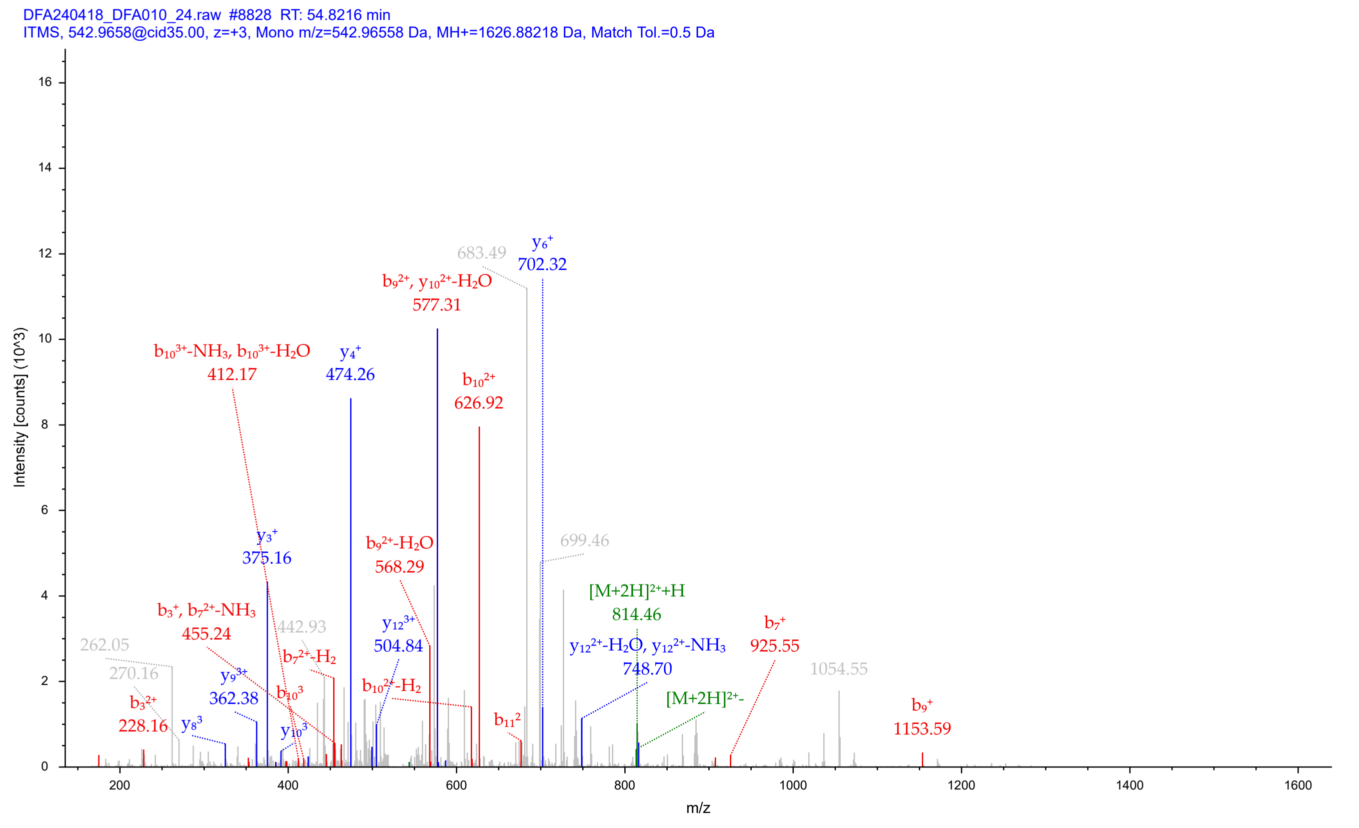

#1	b+	b2+	b3+	Seq.	y+	y2+	y3+	#2
1	114.09134	57.54931	38.70196	L				13
2	342.21358	171.61043	114.74271	R-Carboxy-ethyl	1513.79776	757.40252	505.27077	12
3	455.29764	228.15246	152.43740	L	1285.67552	643.34140	429.23003	11
4	542.32967	271.66847	181.44808	S	1172.59146	586.79937	391.53534	10
5	655.41374	328.21051	219.14276	L	1085.55943	543.28335	362.52466	9
6	811.51485	406.26106	271.17647	R	972.47537	486.74132	324.82997	8
7	925.55778	463.28253	309.19078	N	816.37426	408.69077	272.79627	7
8	1056.59826	528.80277	352.87094	M	702.33133	351.66930	234.78196	6
9	1153.65102	577.32915	385.22186	P	571.29085	286.14906	191.10180	5
10	1252.71944	626.86336	418.24466	V	474.23808	237.62268	158.75088	4
11	1351.78785	676.39756	451.26747	V	375.16967	188.08847	125.72807	3
12	1448.84062	724.92395	483.61839	P	276.10125	138.55427	92.70527	2
13				C-Carbamido-methyl	179.04849	90.02788	60.35435	1

Sequence: NIFPIWALGR, W6-Oxidation (15.99492 Da), R10-3-deoxyglucosone (144.04226 Da)
Charge: +2, Monoisotopic m/z: 673.85773 Da (-1.04 mmu/-1.54 ppm), MH+: 1346.70818 Da, RT: 63.3379 min,
Identified with: Sequest HT (v1.17); XCorr:0.55, Percolator q-Value:5.0e-3, Percolator PEP:4.5e-2,
Fragment match tolerance used for search: 0.02 Da
Fragments used for search: b; b; -NH_3; y

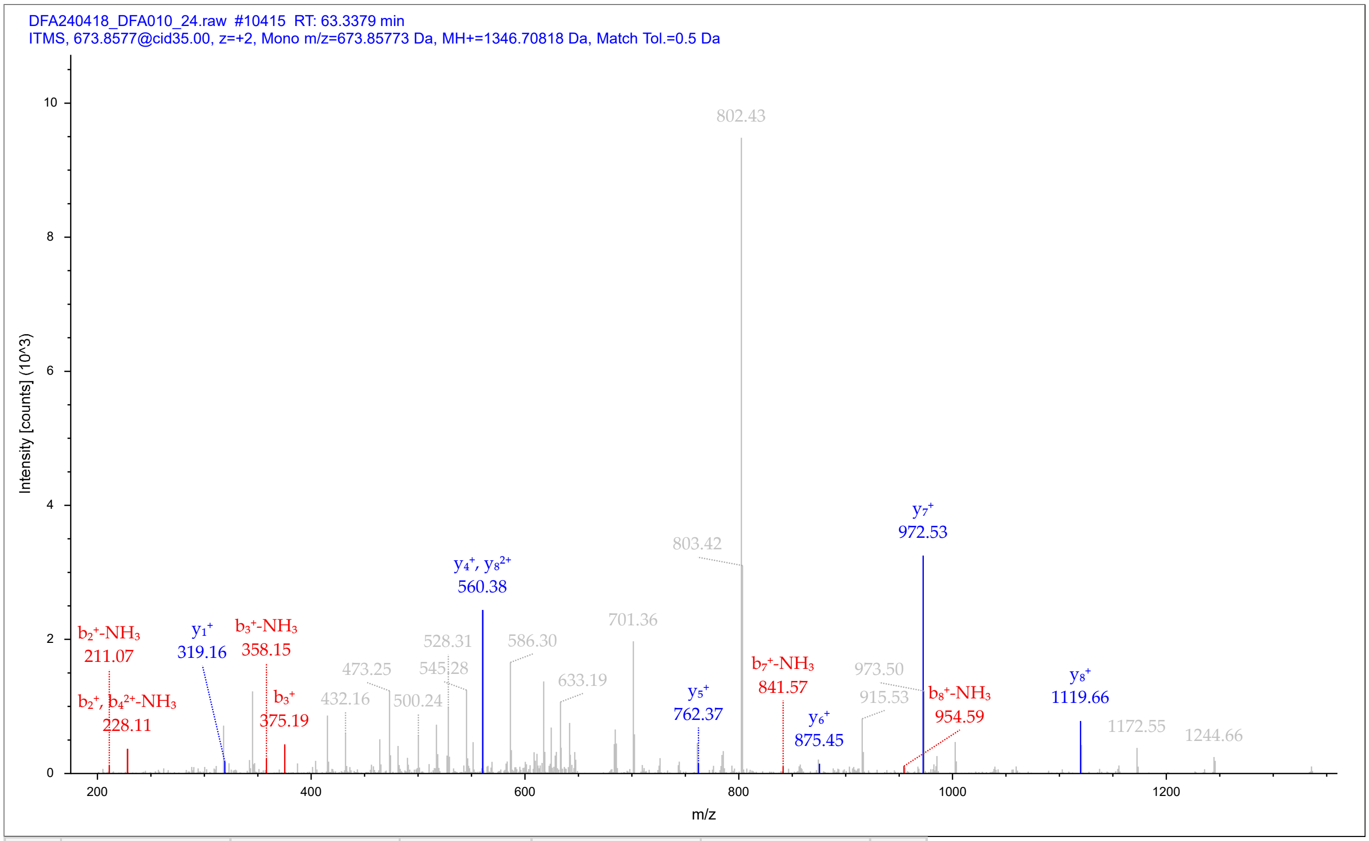

#1	b⁺	b²⁺	Seq.	y⁺	y²⁺	#2
1	115.05020	58.02874	N			10
2	228.13427	114.57077	I	1232.66739	616.83733	9
3	375.20268	188.10498	F	1119.58332	560.29530	8
4	472.25545	236.63136	P	972.51491	486.76109	7
5	585.33951	293.17339	I	875.46214	438.23471	6
6	787.41374	394.21051	W-Oxidation	762.37808	381.69268	5
7	858.45085	429.72906	A	560.30385	280.65556	4
8	971.53492	486.27110	L	489.26674	245.13701	3
9	1028.55638	514.78183	G	376.18267	188.59498	2
10			R-3-deoxy-glucosone	319.16121	160.08424	1

116_202_CEx

Sequence: SAAMLGNSEDHTALSR, R16-Carboxyethyl (72.02113 Da)
Charge: +2, Monoisotopic m/z: 866.40216 Da (+0.2 mmu/+0.24 ppm), MH+: 1731.79704 Da, RT: 73.7457 min,
Identified with: Sequest HT (v1.17); XCorr:0.41, Percolator q-Value:0.0e0, Percolator PEP:5.4e-3,
Fragment match tolerance used for search: 0.02 Da
Fragments used for search: $-H_2O$; y; $-NH_3$; y; b; b; $-H_2O$; b; $-NH_3$; y

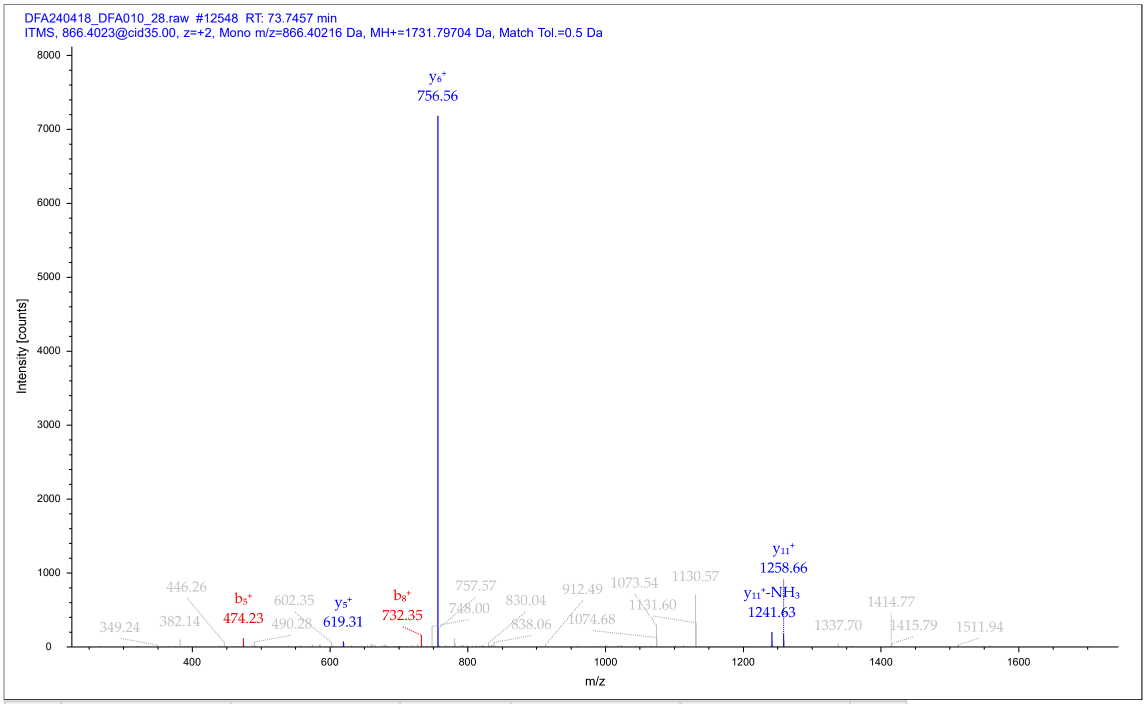

#1	b⁺	b²⁺	Seq.	y⁺	y²⁺	#2
1	88.03930	44.52329	S			16
2	159.07642	80.04185	A	1644.76461	822.88594	15
3	230.11353	115.56040	A	1573.72750	787.36739	14
4	361.15402	181.08065	M	1502.69038	751.84883	13
5	474.23808	237.62268	L	1371.64990	686.32859	12
6	531.25954	266.13341	G	1258.56583	629.78655	11
7	645.30247	323.15487	N	1201.54437	601.27582	10
8	732.33450	366.67089	S	1087.50144	544.25436	9
9	861.37709	431.19219	E	1000.46941	500.73835	8
10	976.40404	488.70566	D	871.42682	436.21705	7
11	1113.46295	557.23511	H	756.39988	378.70358	6
12	1214.51063	607.75895	T	619.34097	310.17412	5
13	1285.54774	643.27751	A	518.29329	259.65028	4
14	1398.63180	699.81954	L	447.25617	224.13173	3
15	1485.66383	743.33555	S	334.17211	167.58969	2
16			R-Carboxy-ethyl	247.14008	124.07368	1

116_202_MGH

Sequence: SAAMLGNSEDHTALSR, R16-Triosyl (72.01840 Da)
Charge: +2, Monoisotopic m/z: 866.40216 Da (+1.57 mmu/+1.81 ppm), MH+: 1731.79704 Da, RT: 73.7457 min,
Identified with: Sequest HT (v1.17); XCorr:0.41, Percolator q-Value:6.6e-3, Percolator PEP:3.4e-2,
Fragment match tolerance used for search: 0.02 Da
Fragments used for search: -H_2O; y; -NH_3; y; b; b; -H_2O; b; -NH_3; y

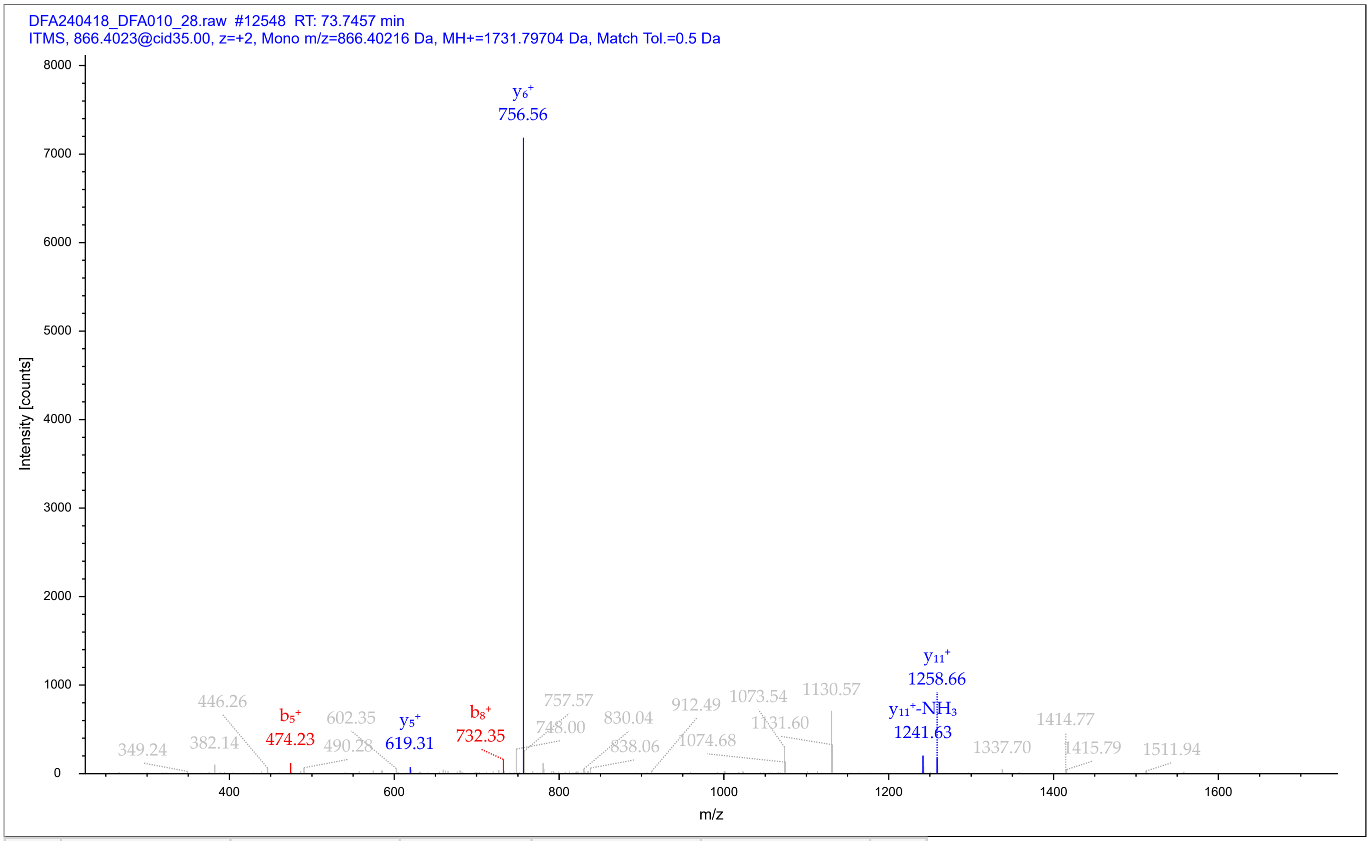

#1	b+	b2+	Seq.	y+	y2+	#2
1	88.03930	44.52329	S			16
2	159.07642	80.04185	A	1644.76188	822.88458	15
3	230.11353	115.56040	A	1573.72477	787.36602	14
4	361.15402	181.08065	M	1502.68765	751.84746	13
5	474.23808	237.62268	L	1371.64717	686.32722	12
6	531.25954	266.13341	G	1258.56310	629.78519	11
7	645.30247	323.15487	N	1201.54164	601.27446	10
8	732.33450	366.67089	S	1087.49871	544.25299	9
9	861.37709	431.19219	E	1000.46668	500.73698	8
10	976.40404	488.70566	D	871.42409	436.21568	7
11	1113.46295	557.23511	H	756.39715	378.70221	6
12	1214.51063	607.75895	T	619.33824	310.17276	5
13	1285.54774	643.27751	A	518.29056	259.64892	4
14	1398.63180	699.81954	L	447.25344	224.13036	3
15	1485.66383	743.33555	S	334.16938	167.58833	2
16			R-Triosyl	247.13735	124.07231	1

Sequence: WYNLAIGSTCPWLK, C10-Carbamidomethyl (57.02146 Da), W1-Trp->Kynurenin (3.99492 Da), W12-Trp->Kynurenin (3.99492 Da), K14-Lederers glucosone (144.04294 Da)
Charge: +2, Monoisotopic m/z: 930.94592 Da (+0.11 mmu/+0.12 ppm), MH+: 1860.88457 Da, RT: 84.5056 min, Identified with: Sequest HT (v1.17); XCorr:0.37, Percolator q-Value:7.0e-3, Percolator PEP:3.2e-2,
Fragment match tolerance used for search: 0.02 Da
Fragments used for search: -H_2O; y; -NH_3; y; b; b; -H_2O; b; -NH_3; y

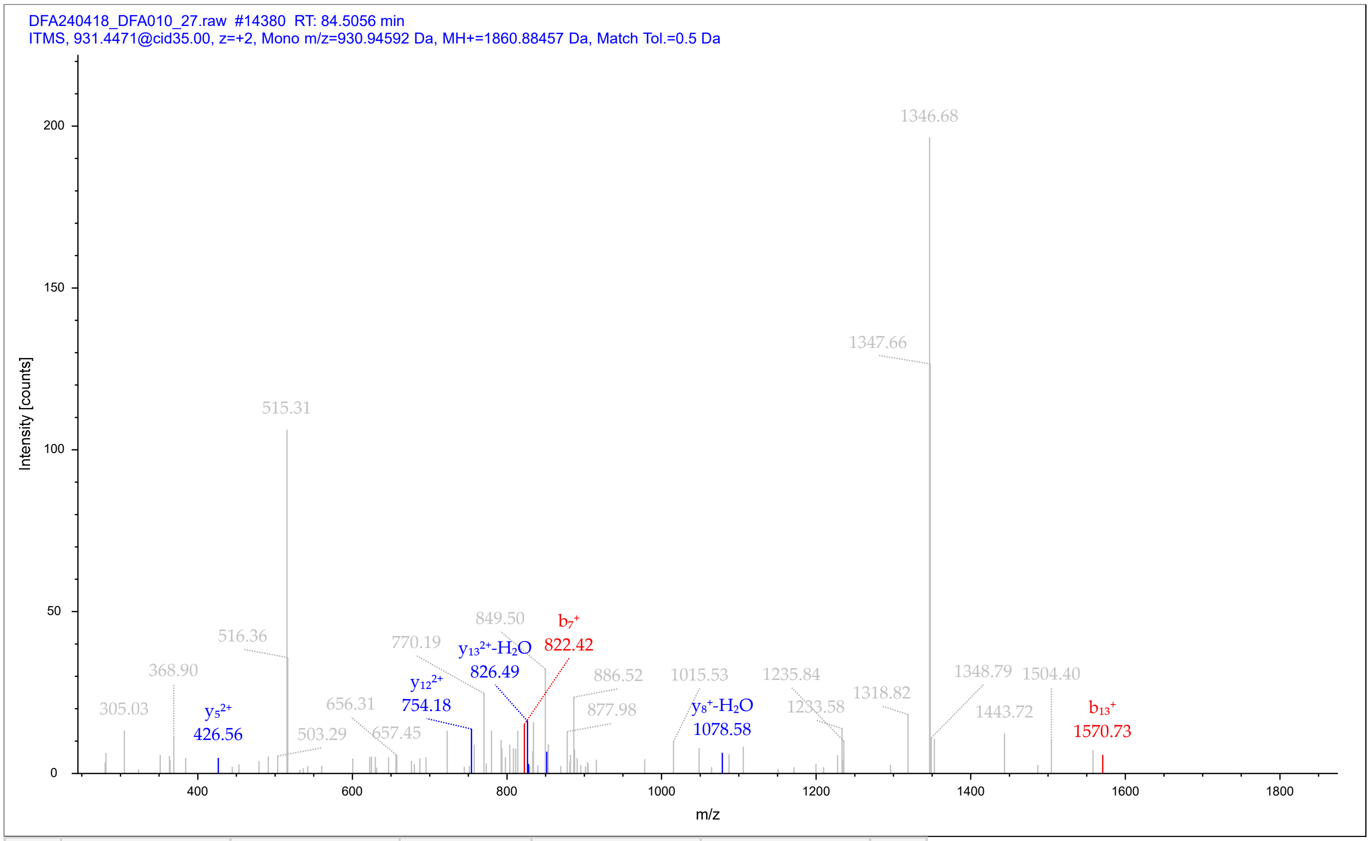

#1	b⁺	b²⁺	Seq.	y⁺	y²⁺	#2
1	191.08150	96.04439	W-Trp-> Kynurenin			14
2	354.14483	177.57605	Y	1670.81012	835.90870	13
3	468.18776	234.59752	N	1507.74679	754.37703	12
4	581.27182	291.13955	L	1393.70386	697.35557	11
5	652.30894	326.65811	A	1280.61980	640.81354	10
6	765.39300	383.20014	I	1209.58268	605.29498	9
7	822.41447	411.71087	G	1096.49862	548.75295	8
8	909.44649	455.22689	S	1039.47716	520.24222	7
9	1010.49417	505.75072	T	952.44513	476.72620	6
10	1170.52482	585.76605	C-Carbamido-methyl	851.39745	426.20236	5
11	1267.57758	634.29243	P	691.36680	346.18704	4
12	1457.65181	729.32954	W-Trp-> Kynurenin	594.31404	297.66066	3
13	1570.73588	785.87158	L	404.23981	202.62354	2
14			K-Lederers glucosone	291.15574	146.08151	1

159_158_CMx

Sequence: YNLAALTNKGNTVFANGDYEKAAEFYK, Y1-Oxidation (15.99492 Da), Y19-Trioxidation (47.98474 Da), Y26-Trioxidation (47.98474 Da), K27-Triosyl (72.01840 Da)
Charge: +6, Monoisotopic m/z: 533.58246 Da (+0.3 mmu/+0.56 ppm), MH+: 3196.45837 Da, RT: 34.5567 min,
Identified with: Sequest HT (v1.17); XCorr:1.36, Percolator q-Value:5.8e-3, Percolator PEP:3.7e-2,
Fragment match tolerance used for search: 0.02 Da
Fragments used for search: $-H_2O$; y; $-NH_3$; y; b; b; $-H_2O$; b; $-NH_3$; y

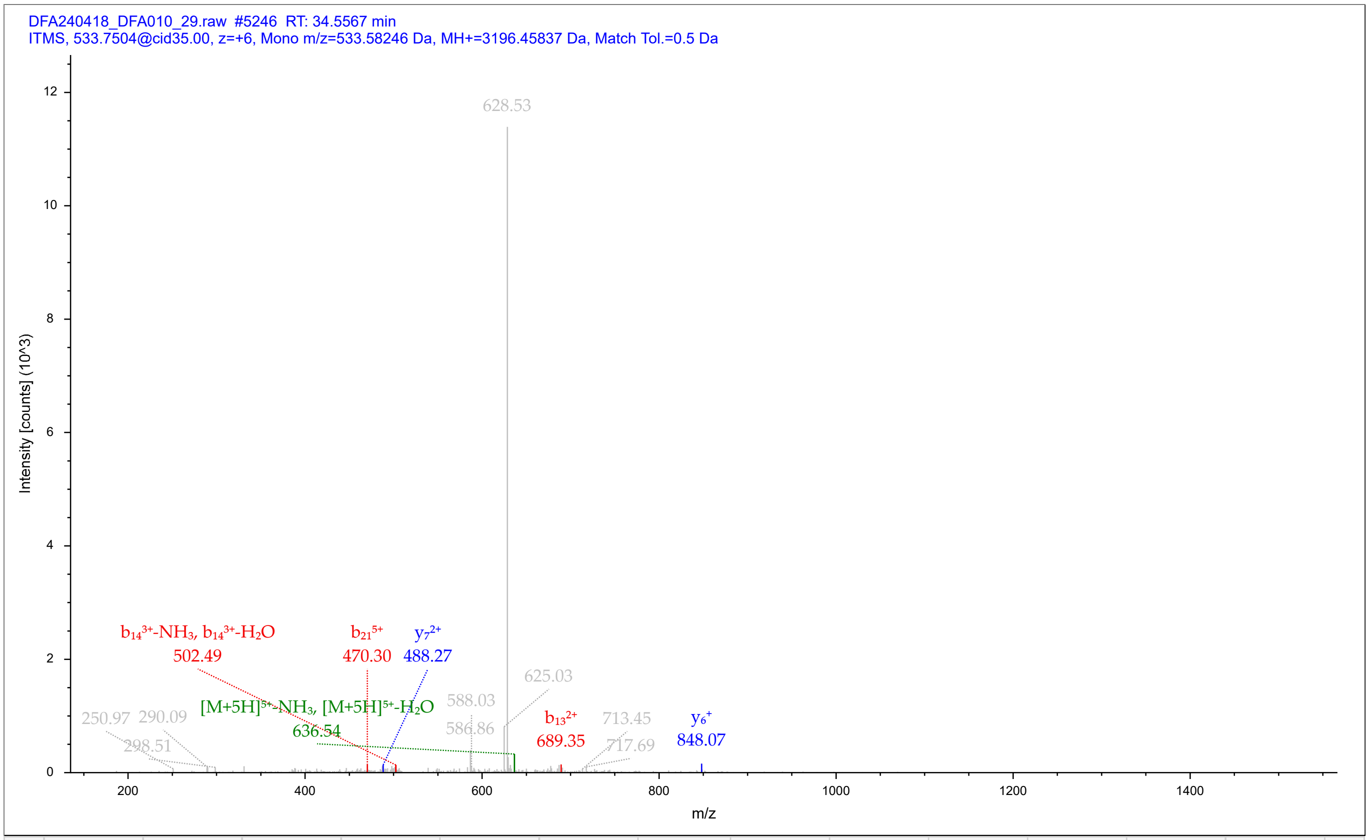

#1	b⁺	b²⁺	b³⁺	b⁴⁺	b⁵⁺	b⁶⁺	Seq.	y⁺	y²⁺	y³⁺	y⁴⁺	y⁵⁺	y⁶⁺	#2
1	180.06552	90.53640	60.69336	45.77184	36.81893	30.85032	Y-Oxidation							27
2	294.10845	147.55786	98.70767	74.28257	59.62751	49.85747	N	3017.39834	1509.20281	1006.47096	755.10504	604.28549	503.73912	26
3	407.19251	204.09989	136.40235	102.55359	82.24432	68.70482	L	2903.35541	1452.18134	968.45665	726.59431	581.47690	484.73197	25
4	478.22963	239.61845	160.08139	120.31286	96.45175	80.54433	A	2790.27134	1395.63931	930.76197	698.32329	558.86009	465.88462	24
5	549.26674	275.13701	183.76043	138.07214	110.65917	92.38385	A	2719.23423	1360.12075	907.08293	680.56402	544.65267	454.04510	23
6	662.35080	331.67904	221.45512	166.34316	133.27598	111.23120	L	2648.19712	1324.60220	883.40389	662.80474	530.44524	442.20558	22
7	763.39848	382.20288	255.13768	191.60508	153.48552	128.07248	T	2535.11305	1268.06016	845.70920	634.53372	507.82843	423.35824	21
8	877.44141	439.22434	293.15199	220.11581	176.29410	147.07963	N	2434.06537	1217.53633	812.02664	609.27180	487.61890	406.51696	20
9	1005.53637	503.27182	335.85031	252.13955	201.91310	168.42879	K	2320.02245	1160.51486	774.01233	580.76107	464.81031	387.50981	19
10	1062.55784	531.78256	354.85746	266.39492	213.31739	177.93237	G	2191.92748	1096.46738	731.31401	548.73733	439.19132	366.16064	18
11	1176.60076	588.80402	392.87177	294.90565	236.12597	196.93952	N	2134.90602	1067.95665	712.30686	534.48196	427.78703	356.65707	17
12	1277.64844	639.32786	426.55433	320.16757	256.33551	213.78080	T	2020.86309	1010.93519	674.29255	505.97123	404.97844	337.64991	16
13	1376.71685	688.86207	459.57714	344.93467	276.14919	230.29221	V	1919.81542	960.41135	640.60999	480.70931	384.76890	320.80863	15
14	1523.78527	762.39627	508.59994	381.70177	305.56288	254.80361	F	1820.74700	910.87714	607.58718	455.94221	364.95522	304.29723	14
15	1594.82238	797.91483	532.27898	399.46105	319.77030	266.64313	A	1673.67859	837.34293	558.56438	419.17510	335.54154	279.78583	13
16	1708.86531	854.93629	570.29329	427.97178	342.57888	285.65028	N	1602.64147	801.82438	534.88534	401.41583	321.33412	267.94631	12
17	1765.88677	883.44703	589.30044	442.22715	353.98318	295.15386	G	1488.59855	744.80291	496.87103	372.90509	298.52553	248.93915	11
18	1880.91372	940.96050	627.64276	470.98389	376.98856	314.32502	D	1431.57708	716.29218	477.86388	358.64973	287.12124	239.43558	10
19	2091.96179	1046.48453	697.99211	523.74590	419.19818	349.49970	Y-Trioxidation	1316.55014	658.77871	439.52156	329.89299	264.11585	220.26442	9
20	2221.00438	1111.00583	741.00631	556.00655	445.00670	371.00679	E	1105.50207	553.25467	369.17221	277.13097	221.90623	185.08974	8
21	2349.09935	1175.05331	783.70463	588.03029	470.62569	392.35595	K	976.45947	488.73338	326.15801	244.87033	196.09772	163.58264	7
22	2420.13646	1210.57187	807.38367	605.78957	484.83311	404.19547	A	848.36451	424.68589	283.45969	212.84659	170.47872	142.23348	6
23	2491.17357	1246.09042	831.06271	623.54885	499.04054	416.03499	A	777.32740	389.16734	259.78065	195.08731	156.27130	130.39396	5
24	2620.21617	1310.61172	874.07691	655.80950	524.84905	437.54209	E	706.29028	353.64878	236.10161	177.32803	142.06388	118.55444	4
25	2767.28458	1384.14593	923.09971	692.57660	554.26274	462.05349	F	577.24769	289.12748	193.08741	145.06738	116.25536	97.04735	3
26	2978.33265	1489.66996	993.44907	745.33862	596.47235	497.22817	Y-Trioxidation	430.17928	215.59328	144.06461	108.30028	86.84168	72.53594	2
27							K-Triosyl	219.13120	110.06924	73.71525	55.53826	44.63206	37.36126	1

158_159_CEx

Sequence: YNLAALTNKGNTVFANGDYEKAAEFYK, Y1-Oxidation (15.99492 Da), Y19-Trioxidation (47.98474 Da), Y26-Trioxidation (47.98474 Da), K27-Carboxyethyl (72.02113 Da)
Charge: +6, Monoisotopic m/z: 533.58246 Da (-0.16 mmu/-0.29 ppm), MH+: 3196.45837 Da, RT: 34.5567 min, Identified with: Sequest HT (v1.17); XCorr:1.24, Percolator q-Value:5.2e-3, Percolator PEP:2.6e-2, ptmRS: Best Site Probabilities:K27(Carboxyethyl): 99.94,
Fragment match tolerance used for search: 0.02 Da
Fragments used for search: -H_2O; y; -NH_3; y; b; b; -H_2O; b; -NH_3; y

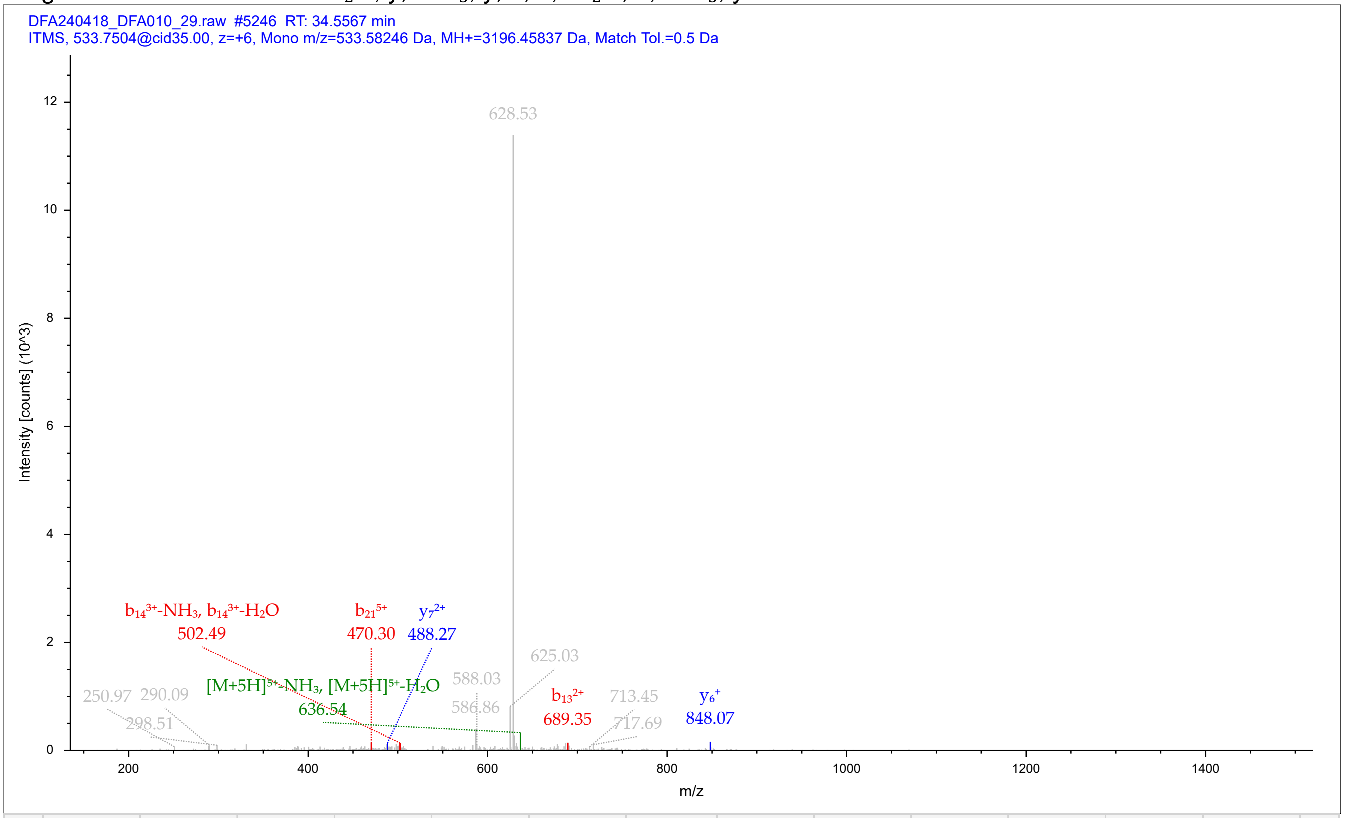

#1	b⁺	b²⁺	b³⁺	b⁴⁺	b⁵⁺	b⁶⁺	Seq.	y⁺	y²⁺	y³⁺	y⁴⁺	y⁵⁺	y⁶⁺	#2
1	180.06552	90.53640	60.69336	45.77184	36.81893	30.85032	Y-Oxidation							27
2	294.10845	147.55786	98.70767	74.28257	59.62751	49.85747	N	3017.40107	1509.20417	1006.47187	755.10572	604.28603	503.73957	26
3	407.19251	204.09989	136.40235	102.55359	82.24432	68.70482	L	2903.35814	1452.18271	968.45756	726.59499	581.47745	484.73242	25
4	478.22963	239.61845	160.08139	120.31286	96.45175	80.54433	A	2790.27407	1395.64068	930.76288	698.32398	558.86064	465.88508	24
5	549.26674	275.13701	183.76043	138.07214	110.65917	92.38385	A	2719.23696	1360.12212	907.08384	680.56470	544.65321	454.04556	23
6	662.35080	331.67904	221.45512	166.34316	133.27598	111.23120	L	2648.19985	1324.60356	883.40480	662.80542	530.44579	442.20604	22
7	763.39848	382.20288	255.13768	191.60508	153.48552	128.07248	T	2535.11578	1268.06153	845.71011	634.53440	507.82898	423.35869	21
8	877.44141	439.22434	293.15199	220.11581	176.29410	147.07963	N	2434.06810	1217.53769	812.02755	609.27248	487.61944	406.51741	20
9	1005.53637	503.27182	335.85031	252.13955	201.91310	168.42879	K	2320.02518	1160.51623	774.01324	580.76175	464.81086	387.51026	19
10	1062.55784	531.78256	354.85746	266.39492	213.31739	177.93237	G	2191.93021	1096.46875	731.31492	548.73801	439.19186	366.16110	18
11	1176.60076	588.80402	392.87177	294.90565	236.12597	196.93952	N	2134.90875	1067.95801	712.30777	534.48264	427.78757	356.65752	17
12	1277.64844	639.32786	426.55433	320.16757	256.33551	213.78080	T	2020.86582	1010.93655	674.29346	505.97191	404.97899	337.65037	16
13	1376.71685	688.86207	459.57714	344.93467	276.14919	230.29221	V	1919.81814	960.41271	640.61090	480.70999	384.76945	320.80909	15
14	1523.78527	762.39627	508.59994	381.70177	305.56288	254.80361	F	1820.74973	910.87850	607.58809	455.94289	364.95577	304.29769	14
15	1594.82238	797.91483	532.27898	399.46105	319.77030	266.64313	A	1673.68132	837.34430	558.56529	419.17579	335.54208	279.78628	13
16	1708.86531	854.93629	570.29329	427.97178	342.57888	285.65028	N	1602.64420	801.82574	534.88625	401.41651	321.33466	267.94676	12
17	1765.88677	883.44703	589.30044	442.22715	353.98318	295.15386	G	1488.60128	744.80428	496.87194	372.90578	298.52608	248.93961	11
18	1880.91372	940.96050	627.64276	470.98389	376.98856	314.32502	D	1431.57981	716.29354	477.86479	358.65041	287.12178	239.43603	10
19	2091.96179	1046.48453	697.99211	523.74590	419.19818	349.49970	Y-Trioxidation	1316.55287	658.78007	439.52247	329.89367	264.11640	220.26488	9
20	2221.00438	1111.00583	741.00631	556.00655	445.00670	371.00679	E	1105.50480	553.25604	369.17312	277.13166	221.90678	185.09020	8
21	2349.09935	1175.05331	783.70463	588.03029	470.62569	392.35595	K	976.46220	488.73474	326.15892	244.87101	196.09826	163.58310	7
22	2420.13646	1210.57187	807.38367	605.78957	484.83311	404.19547	A	848.36724	424.68726	283.46060	212.84727	170.47927	142.23394	6
23	2491.17357	1246.09042	831.06271	623.54885	499.04054	416.03499	A	777.33013	389.16870	259.78156	195.08799	156.27185	130.39442	5
24	2620.21617	1310.61172	874.07691	655.80950	524.84905	437.54209	E	706.29301	353.65014	236.10252	177.32871	142.06442	118.55490	4
25	2767.28458	1384.14593	923.09971	692.57660	554.26274	462.05349	F	577.25042	289.12885	193.08832	145.06806	116.25591	97.04780	3
26	2978.33265	1489.66996	993.44907	745.33862	596.47235	497.22817	Y-Trioxidation	430.18201	215.59464	144.06552	108.30096	86.84222	72.53640	2
27							K-Carboxyethy	219.13393	110.07060	73.71616	55.53894	44.63261	37.36172	1

93

Sequence: NLCQMSQAVLDEDLFNFLQAHWFTCELLWYMHVRK, C3-Carbamidomethyl (57.02146 Da), C25-Carbamidomethyl (57.02146 Da), W22-Trp->Oxolactone (13.97927 Da), R34-Carboxyethyl (72.02113 Da), F15-Dioxidation (31.98983 Da), K35-Pyrazine (46.99344 Da)

Charge: +3, Monoisotopic m/z: 1536.68481 Da (-13.6 mmu/-8.85 ppm), MH+: 4608.03989 Da, RT: 28.2688 min, Identified with: Sequest HT (v1.17); XCorr:0.37, Percolator q-Value:4.7e-3, Percolator PEP:1.6e-2,

Fragment match tolerance used for search: 0.02 Da

Fragments used for search: -H_2O; y; -NH_3; y; b; b; -H_2O; b; -NH_3; y

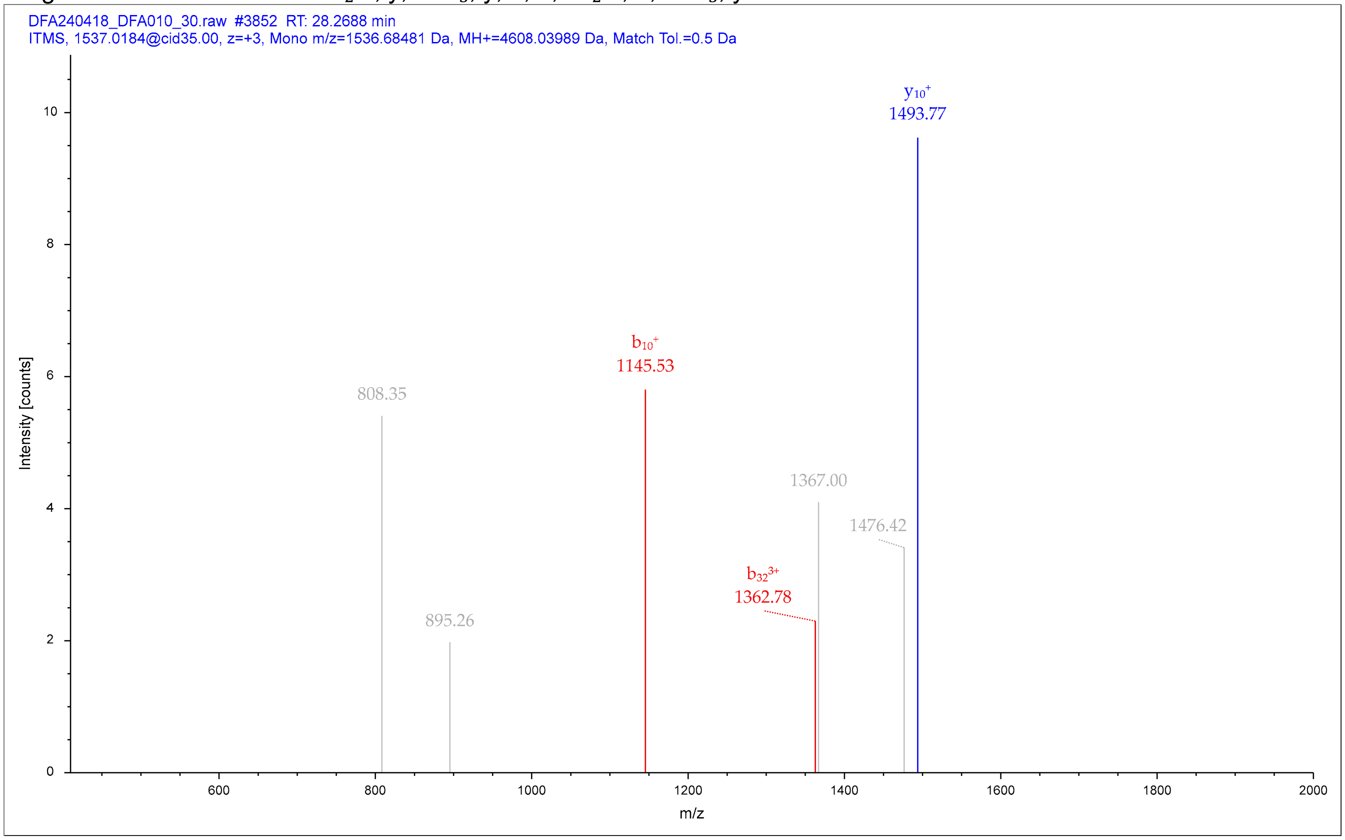

#1	b⁺	b²⁺	b³⁺	Seq.	y⁺	y²⁺	y³⁺	#2
1	115.05020	58.02874	39.02159	N				35
2	228.13427	114.57077	76.71627	L	4494.03774	2247.52251	1498.68410	34
3	388.16492	194.58610	130.05982	C-Carbamidomethyl	4380.95368	2190.98048	1460.98941	33
4	516.22349	258.61539	172.74602	Q	4220.92303	2110.96515	1407.64586	32
5	647.26398	324.13563	216.42618	M	4092.86445	2046.93586	1364.95967	31
6	734.29601	367.65164	245.43685	S	3961.82397	1981.41562	1321.27951	30
7	862.35458	431.68093	288.12305	Q	3874.79194	1937.89961	1292.26883	29
8	933.39170	467.19949	311.80208	A	3746.73336	1873.87032	1249.58264	28
9	1032.46011	516.73369	344.82489	V	3675.69625	1838.35176	1225.90360	27
10	1145.54418	573.27573	382.51958	L	3576.62783	1788.81755	1192.88080	26
11	1260.57112	630.78920	420.86189	D	3463.54377	1732.27552	1155.18611	25
12	1389.61371	695.31049	463.87609	E	3348.51683	1674.76205	1116.84379	24
13	1504.64065	752.82397	502.21840	D	3219.47423	1610.24075	1073.82960	23
14	1617.72472	809.36600	539.91309	L	3104.44729	1552.72728	1035.48728	22
15	1796.78296	898.89512	599.59917	F-Dioxidation	2991.36323	1496.18525	997.79259	21
16	1910.82589	955.91658	637.61348	N	2812.30498	1406.65613	938.10651	20
17	2057.89430	1029.45079	686.63629	F	2698.26206	1349.63467	900.09220	19
18	2170.97837	1085.99282	724.33097	L	2551.19364	1276.10046	851.06940	18
19	2299.03694	1150.02211	767.01717	Q	2438.10958	1219.55843	813.37471	17
20	2370.07406	1185.54067	790.69620	A	2310.05100	1155.52914	770.68852	16
21	2507.13297	1254.07012	836.38251	H	2239.01389	1120.01058	747.00948	15
22	2707.19155	1354.09941	903.06870	W-Trp->Oxolactone	2101.95498	1051.48113	701.32318	14
23	2854.25996	1427.63362	952.09150	F	1901.89640	951.45184	634.63698	13
24	2955.30764	1478.15746	985.77406	T	1754.82798	877.91763	585.61418	12
25	3115.33829	1558.17278	1039.11761	C-Carbamidomethyl	1653.78031	827.39379	551.93162	11
26	3244.38088	1622.69408	1082.13181	E	1493.74966	747.37847	498.58807	10
27	3357.46495	1679.23611	1119.82650	L	1364.70706	682.85717	455.57387	9
28	3470.54901	1735.77814	1157.52119	L	1251.62300	626.31514	417.87918	8
29	3656.62832	1828.81780	1219.54763	W	1138.53894	569.77311	380.18450	7
30	3819.69165	1910.34946	1273.90207	Y	952.45962	476.73345	318.15806	6
31	3950.73214	1975.86971	1317.58223	M	789.39629	395.20179	263.80362	5
32	4087.79105	2044.39916	1363.26853	H	658.35581	329.68154	220.12345	4
33	4186.85946	2093.93337	1396.29134	V	521.29690	261.15209	174.43715	3
34	4414.98170	2207.99449	1472.33208	R-Carboxyethyl	422.22848	211.61788	141.41435	2
35				K-Pyrazine	194.10624	97.55676	65.37360	1

51

Sequence: ITFSDVRPNQQEYKISSFEQR, Y13-Oxidation (15.99492 Da), F18-Oxidation (15.99492 Da), K14-Glycerinyl (88.01674 Da), R7-Delta:H(2)C(3)O(1) (54.01057 Da)

Charge: +6, Monoisotopic m/z: 458.55310 Da (-2.33 mmu/-5.08 ppm), MH+: 2746.28222 Da, RT: 35.8377 min,

Identified with: Sequest HT (v1.17); XCorr:1.08, Percolator q-Value:3.4e-3, Percolator PEP:1.2e-2,

Fragment match tolerance used for search: 0.02 Da

Fragments used for search: -H_2O; y; -NH_3; y; b; b; -H_2O; b; -NH_3; y

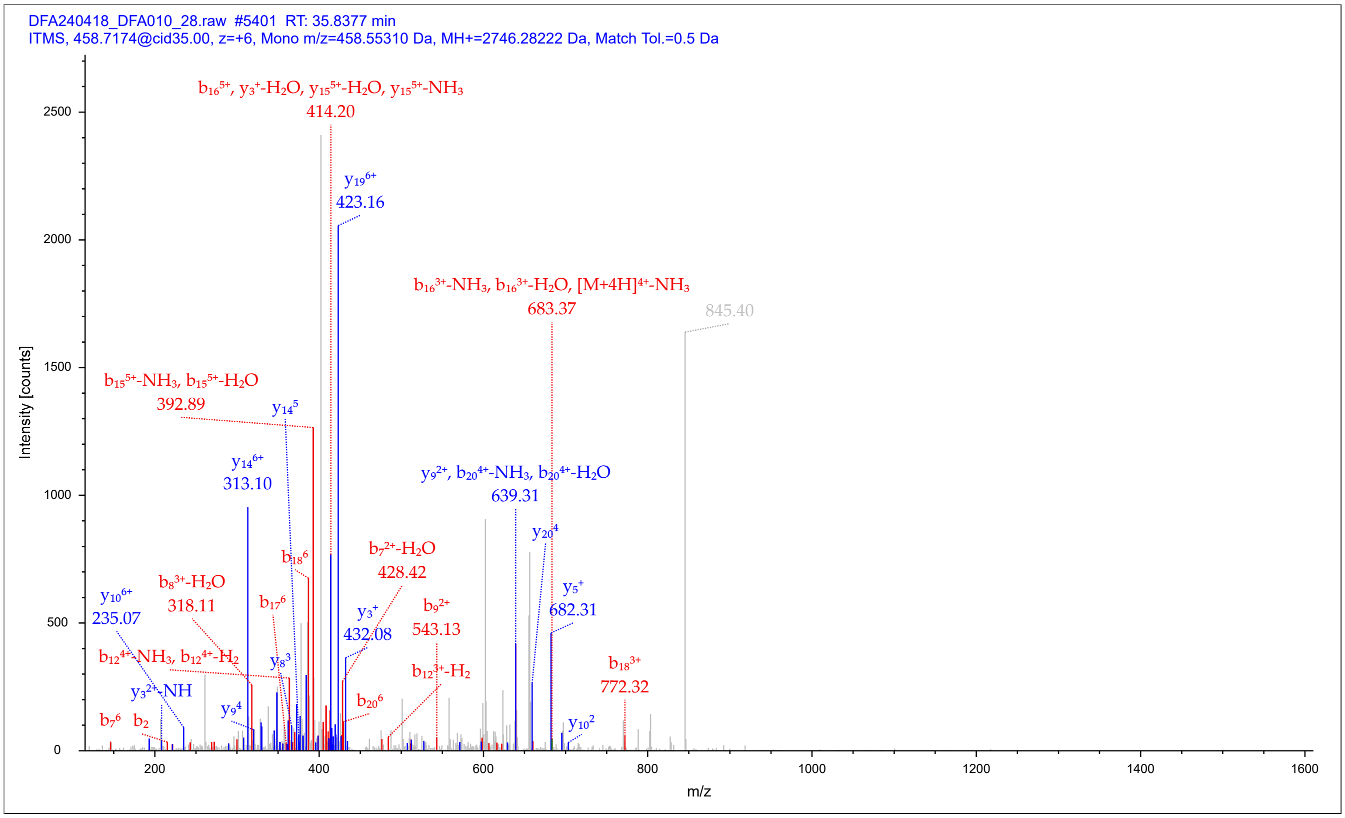

#1	b⁺	b²⁺	b³⁺	b⁴⁺	b⁵⁺	b⁶⁺	Seq.	y⁺	y²⁺	y³⁺	y⁴⁺	y⁵⁺	y⁶⁺	#2
1	114.09134	57.54931	38.70196	29.27829	23.62409	19.85462	I							21
2	215.13902	108.07315	72.38452	54.54021	43.83363	36.69590	T	2633.21211	1317.10969	878.40889	659.05849	527.44824	439.70808	20
3	362.20743	181.60735	121.40733	91.30732	73.24731	61.20730	F	2532.16443	1266.58585	844.72633	633.79657	507.23871	422.86680	19
4	449.23946	225.12337	150.41800	113.06532	90.65371	75.71264	S	2385.09602	1193.05165	795.70352	597.02946	477.82503	398.35540	18
5	564.26640	282.63684	188.76032	141.82206	113.65910	94.88380	D	2298.06399	1149.53563	766.69285	575.27146	460.41862	383.85006	17
6	663.33482	332.17105	221.78312	166.58916	133.47278	111.39520	V	2183.03705	1092.02216	728.35053	546.51472	437.41323	364.67891	16
7	873.44649	437.22689	291.82035	219.11708	175.49512	146.41381	R-Delta: H(2)C(3)O(1)	2083.96863	1042.48796	695.32773	521.74762	417.59955	348.16750	15
8	970.49926	485.75327	324.17127	243.38027	194.90567	162.58927	P	1873.85696	937.43212	625.29050	469.21970	375.57721	313.14889	14
9	1084.54219	542.77473	362.18558	271.89100	217.71426	181.59643	N	1776.80419	888.90574	592.93958	444.95651	356.16666	296.97343	13
10	1212.60076	606.80402	404.87177	303.90565	243.32597	202.93952	Q	1662.76127	831.88427	554.92527	416.44577	333.35807	277.96627	12
11	1340.65934	670.83331	447.55796	335.92029	268.93769	224.28262	Q	1534.70269	767.85498	512.23908	384.43113	307.74636	256.62318	11
12	1469.70193	735.35460	490.57216	368.18094	294.74621	245.78972	E	1406.64411	703.82569	469.55289	352.41649	282.13464	235.28008	10
13	1648.76018	824.88373	550.25824	412.94550	330.55786	275.63276	Y-Oxidation	1277.60152	639.30440	426.53869	320.15584	256.32613	213.77298	9
14	1864.87188	932.93958	622.29548	466.97343	373.78020	311.65138	K-Glycerinyl	1098.54328	549.77528	366.85261	275.39128	220.51448	183.92994	8
15	1977.95594	989.48161	659.99017	495.24444	396.39701	330.49872	I	882.43157	441.71942	294.81538	221.36335	177.29214	147.91133	7
16	2064.98797	1032.99762	689.00084	517.00245	413.80342	345.00406	S	769.34751	385.17739	257.12069	193.09233	154.67532	129.06398	6
17	2152.02000	1076.51364	718.01152	538.76046	431.20982	359.50940	S	682.31548	341.66138	228.11001	171.33433	137.26892	114.55864	5
18	2315.08333	1158.04530	772.36596	579.52629	463.82249	386.68662	F-Oxidation	595.28345	298.14536	199.09933	149.57632	119.86251	100.05331	4
19	2444.12592	1222.56660	815.38016	611.78694	489.63101	408.19372	E	432.22012	216.61370	144.74489	108.81049	87.24985	72.87608	3
20	2572.18450	1286.59589	858.06635	643.80158	515.24272	429.53681	Q	303.17753	152.09240	101.73069	76.54984	61.44133	51.36899	2
21							R	175.11895	88.06311	59.04450	44.53520	35.82961	30.02589	1

157

Sequence: YLGIMKPLTYPMR, M5-Oxidation (15.99492 Da), R13-Carboxyethyl (72.02113 Da), K6-Pyrazine (46.99344 Da)
Charge: +3, Monoisotopic m/z: 573.28918 Da (-1.63 mmu/-2.84 ppm), MH+: 1717.85300 Da, RT: 42.0660 min, Identified with: Sequest HT (v1.17); XCorr:0.82, Percolator q-Value:3.6e-3, Percolator PEP:2.8e-2,
Fragment match tolerance used for search: 0.02 Da
Fragments used for search: -H_2O; y; b; b; -H_2O; y

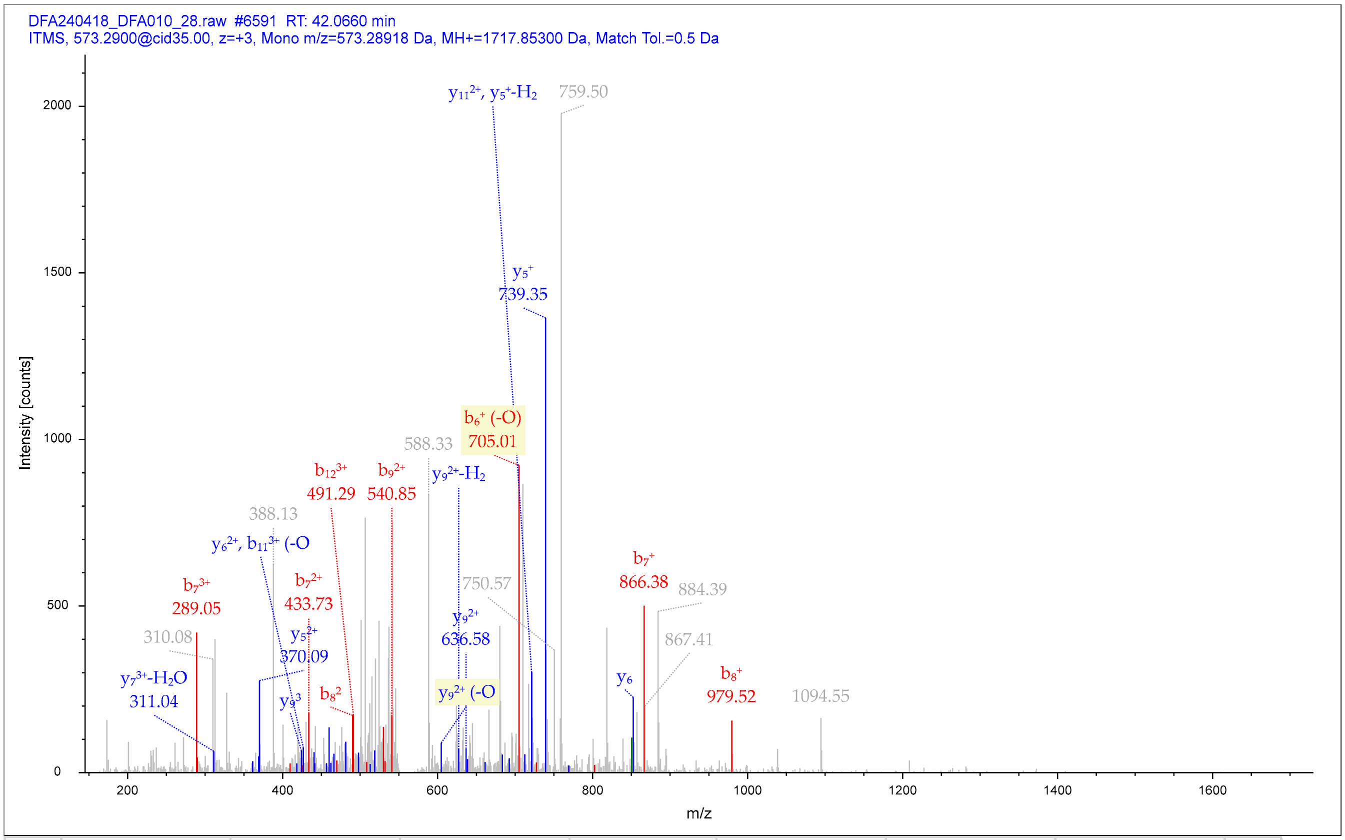

#1	b+	b2+	b3+	Seq.	y+	y2+	y3+	#2
1	164.07061	82.53894	55.36172	Y				13
2	277.15467	139.08097	93.05641	L	1554.79456	777.90092	518.93637	12
3	334.17613	167.59170	112.06356	G	1441.71049	721.35889	481.24168	11
4	447.26020	224.13374	149.75825	I	1384.68903	692.84815	462.23453	10
5	594.29560	297.65144	198.77005	M-Oxidation	1271.60497	636.30612	424.53984	9
6	769.38400	385.19564	257.13285	K-Pyrazine	1124.56957	562.78842	375.52804	8
7	866.43676	433.72202	289.48377	P	949.48116	475.24422	317.16524	7
8	979.52083	490.26405	327.17846	L	852.42840	426.71784	284.81432	6
9	1080.56851	540.78789	360.86102	T	739.34434	370.17581	247.11963	5
10	1243.63183	622.31956	415.21546	Y	638.29666	319.65197	213.43707	4
11	1340.68460	670.84594	447.56638	P	475.23333	238.12030	159.08263	3
12	1471.72508	736.36618	491.24655	M	378.18057	189.59392	126.73171	2
13				R-Carboxyethyl	247.14008	124.07368	83.05154	1

179_Glarg

Sequence: FMQAVTGWK, M2-Oxidation (15.99492 Da), W8-Trp->Kynurenin (3.99492 Da)
Charge: +2, Monoisotopic m/z: 544.26459 Da (-1.1 mmu/-2.02 ppm), MH+: 1087.52190 Da, RT: 42.4771 min,
Identified with: Sequest HT (v1.17); XCorr:1.09, Percolator q-Value:7.9e-3, Percolator PEP:6.8e-2,
Fragment match tolerance used for search: 0.02 Da
Fragments used for search: -H_2O; y; -NH_3; y; b; b; -H_2O; b; -NH_3; y

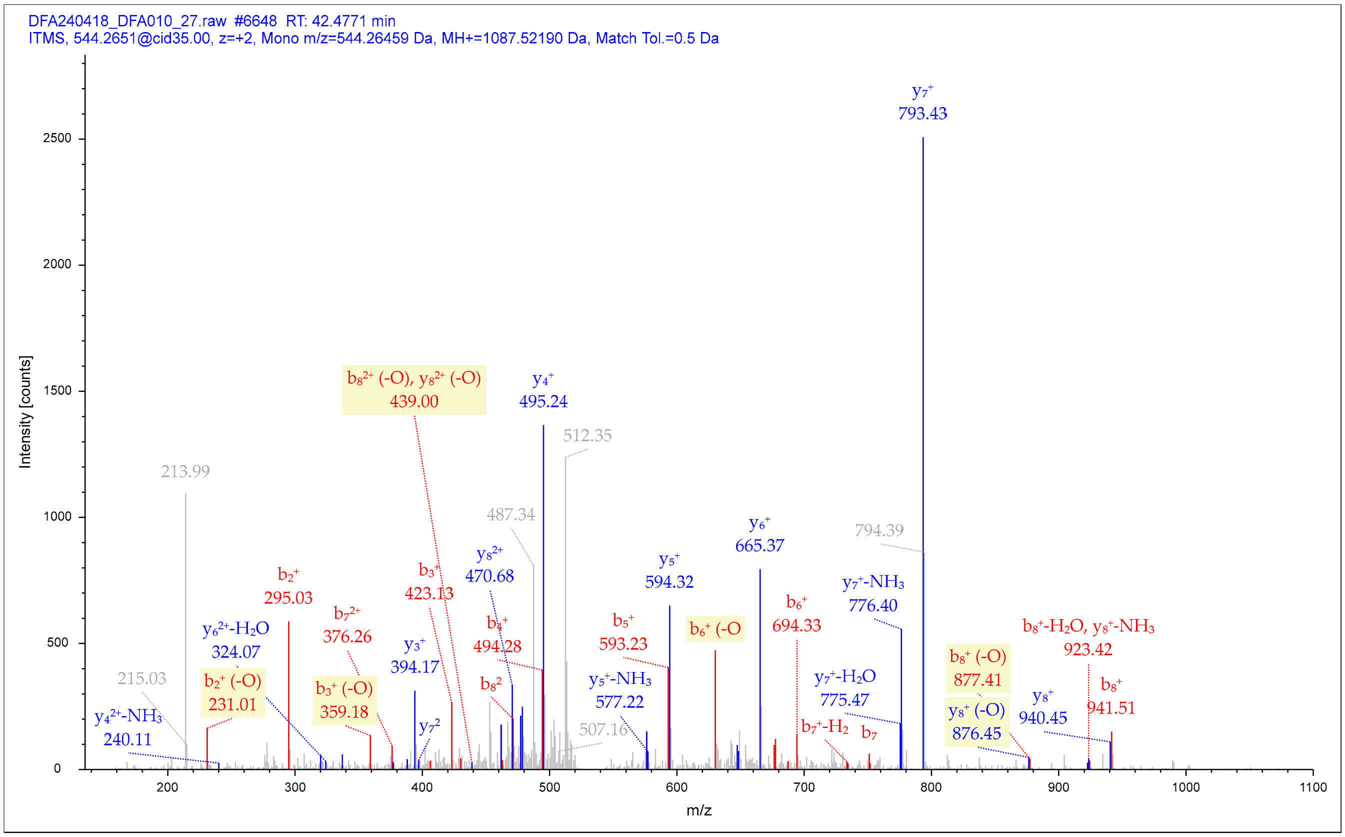

#1	b⁺	b²⁺	Seq.	y⁺	y²⁺	#2
1	148.07569	74.54148	F			9
2	295.11109	148.05918	M-Oxidation	940.45568	470.73148	8
3	423.16967	212.08847	Q	793.42028	397.21378	7
4	494.20678	247.60703	A	665.36170	333.18449	6
5	593.27520	297.14124	V	594.32459	297.66593	5
6	694.32287	347.66508	T	495.25617	248.13173	4
7	751.34434	376.17581	G	394.20850	197.60789	3
8	941.41857	471.21292	W-Trp->Kynurenin	337.18703	169.09715	2
9			K	147.11280	74.06004	1

Sequence: QWWRPWVDHASSR, W3-Trp->Oxolactone (13.97927 Da), R4-Glarg (39.99949 Da), R13-3-deoxyglucosone (144.04226 Da)
Charge: +3, Monoisotopic m/z: 636.95343 Da (0 mmu/0 ppm), MH+: 1908.84574 Da, RT: 60.4263 min,
Identified with: Sequest HT (v1.17); XCorr:0.59, Percolator q-Value:6.7e-3, Percolator PEP:2.0e-2,
Fragment match tolerance used for search: 0.02 Da
Fragments used for search: -H_2O; y; b; b; -H_2O; b; -NH_3; y

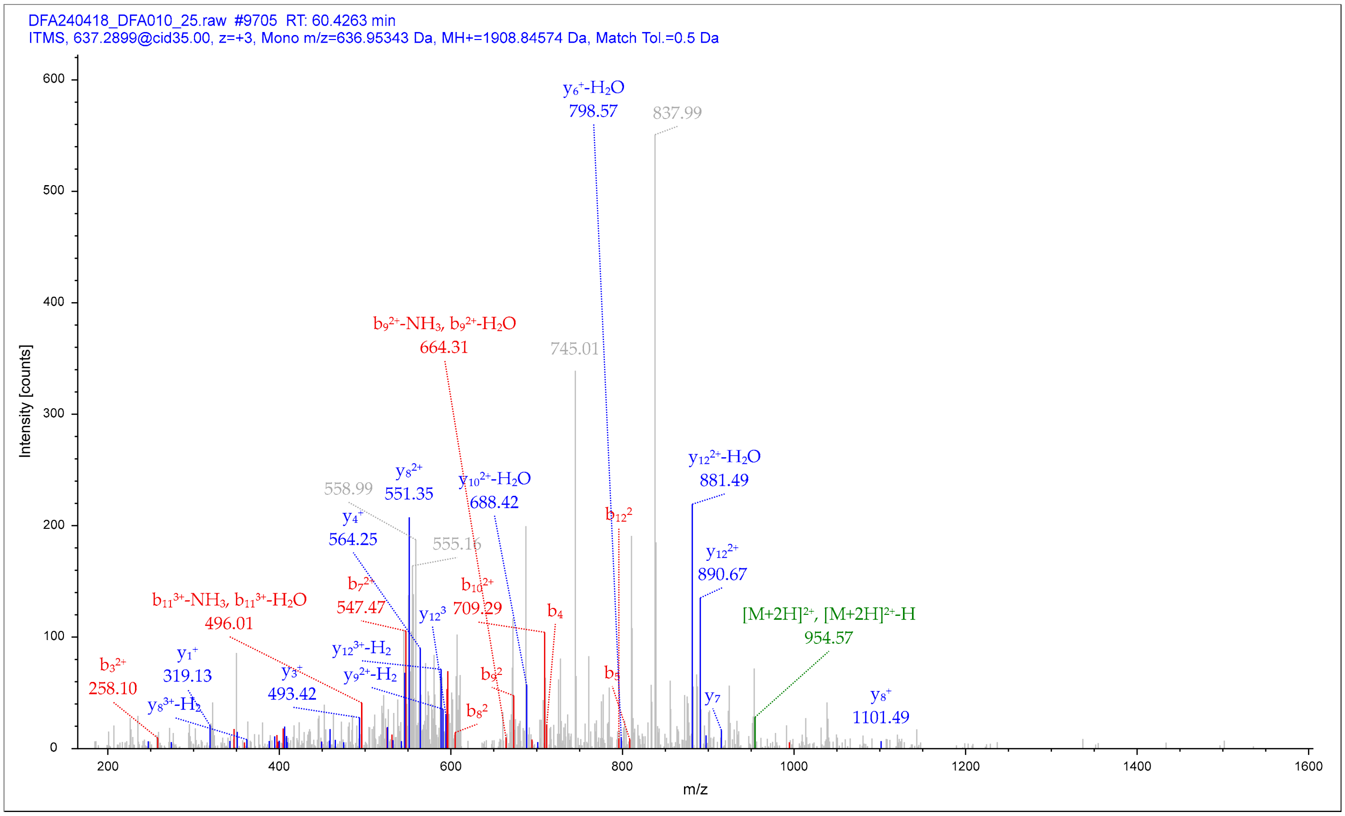

#1	b⁺	b²⁺	b³⁺	Seq.	y⁺	y²⁺	y³⁺	#2
1	129.06585	65.03657	43.69347	Q				13
2	315.14517	158.07622	105.71991	W	1780.78722	890.89725	594.26726	12
3	515.20374	258.10551	172.40610	W-Trp->Oxolactone	1594.70791	797.85759	532.24082	11
4	711.30435	356.15581	237.77297	R-Glarg	1394.64933	697.82830	465.55463	10
5	808.35711	404.68219	270.12389	P	1198.54873	599.77800	400.18776	9
6	994.43642	497.72185	332.15033	W	1101.49596	551.25162	367.83684	8
7	1093.50484	547.25606	365.17313	V	915.41665	458.21196	305.81040	7
8	1208.53178	604.76953	403.51544	D	816.34824	408.67776	272.78760	6
9	1345.59069	673.29898	449.20175	H	701.32129	351.16429	234.44528	5
10	1416.62781	708.81754	472.88079	A	564.26238	282.63483	188.75898	4
11	1503.65984	752.33356	501.89146	S	493.22527	247.11627	165.07994	3
12	1590.69186	795.84957	530.90214	S	406.19324	203.60026	136.06926	2
13				R-3-deoxyglucosone	319.16121	160.08424	107.05859	1

Sequence: KLGSDMGNAER, K1-Triosyl (72.01840 Da)
Charge: +3, Monoisotopic m/z: 417.19836 Da (-0.29 mmu/-0.69 ppm), MH+: 1249.58054 Da, RT: 19.5583 min,
Identified with: Sequest HT (v1.17); XCorr:1.37, Percolator q-Value:4.5e-3, Percolator PEP:2.6e-2,
Fragment match tolerance used for search: 0.02 Da
Fragments used for search: -H_2O; y; -NH_3; y; b; b; -H_2O; b; -NH_3; y

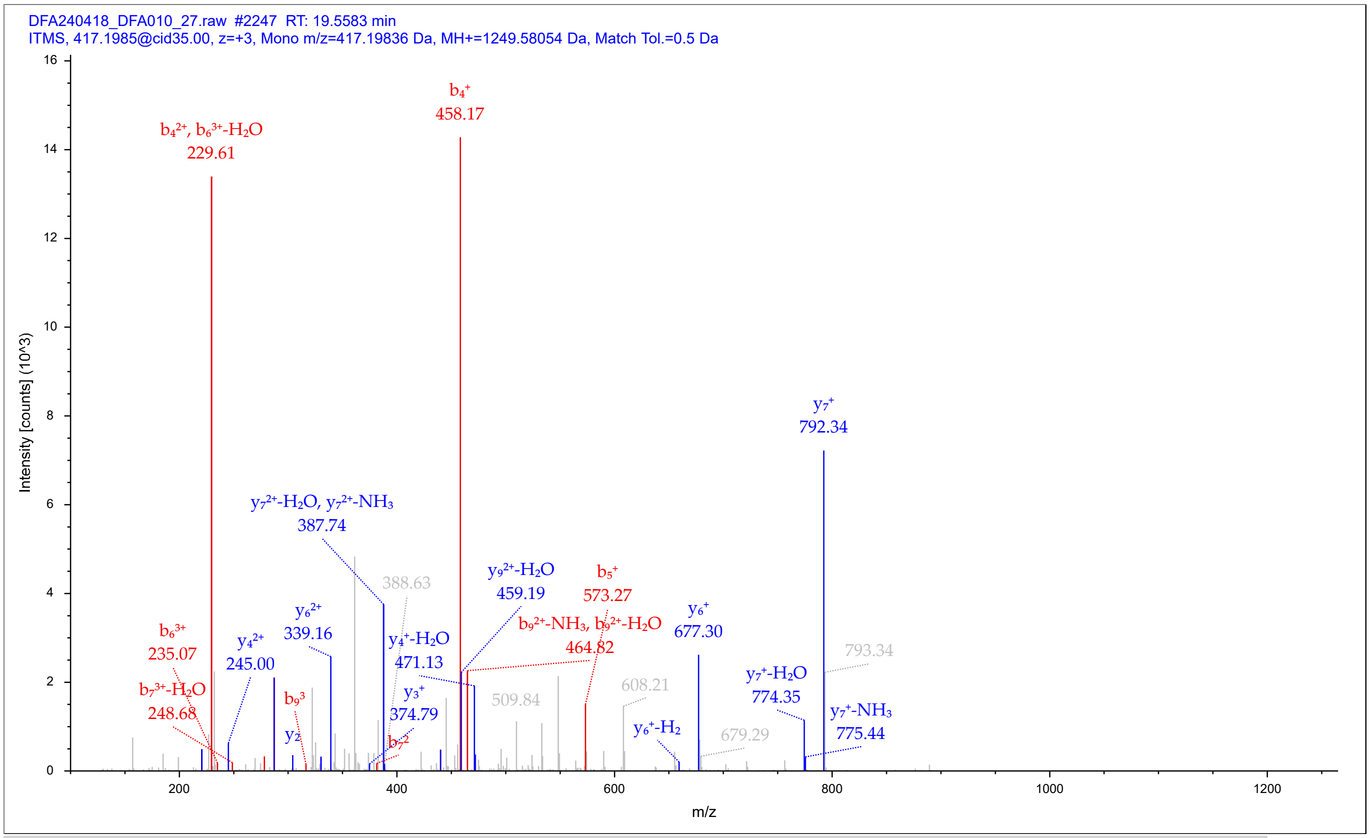

#1	b+	b2+	b3+	Seq.	y+	y2+	y3+	#2
1	201.12064	101.06396	67.71173	K-Triosyl				11
2	314.20470	157.60599	105.40642	L	1049.46803	525.23766	350.49420	10
3	371.22617	186.11672	124.41357	G	936.38397	468.69562	312.79951	9
4	458.25820	229.63274	153.42425	S	879.36251	440.18489	293.79235	8
5	573.28514	287.14621	191.76656	D	792.33048	396.66888	264.78168	7
6	704.32562	352.66645	235.44673	M	677.30353	339.15541	226.43936	6
7	761.34709	381.17718	254.45388	G	546.26305	273.63516	182.75920	5
8	875.39001	438.19865	292.46819	N	489.24159	245.12443	163.75205	4
9	946.42713	473.71720	316.14723	A	375.19866	188.10297	125.73774	3
10	1075.46972	538.23850	359.16142	E	304.16155	152.58441	102.05870	2
11				R	175.11895	88.06311	59.04450	1

Sequence: SLYSLGGVRSLNVASGSGKSGGYGFGR, Y3-Trioxidation (47.98474 Da), R27-Glarg (39.99949 Da), K19-Lederers pentosone (114.03234 Da)
Charge: +6, Monoisotopic m/z: 473.39737 Da (-1.97 mmu/-4.15 ppm), MH+: 2835.34783 Da, RT: 32.0420 min,
Identified with: Sequest HT (v1.17); XCorr:0.92, Percolator q-Value:7.1e-3, Percolator PEP:2.9e-2,
Fragment match tolerance used for search: 0.02 Da
Fragments used for search: $-H_2O$; y; $-NH_3$; y; b; b; $-H_2O$; b; $-NH_3$; y

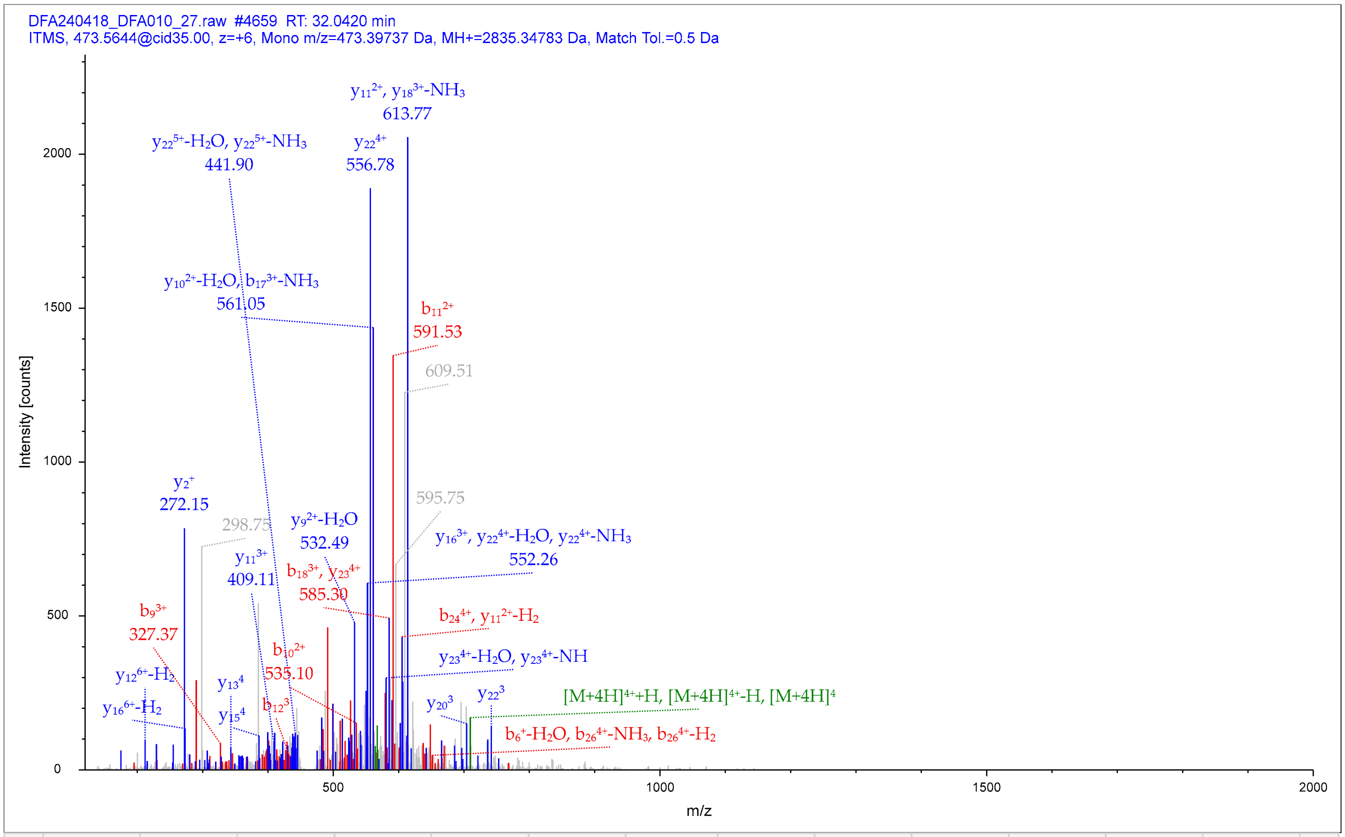

#1	b⁺	b²⁺	b³⁺	b⁴⁺	b⁵⁺	b⁶⁺	Seq.	y⁺	y²⁺	y³⁺	y⁴⁺	y⁵⁺	y⁶⁺	#2
1	88.03930	44.52329	30.01795	22.76528	18.41368	15.51261	S							27
2	201.12337	101.06532	67.71264	51.03630	41.03050	34.35996	L	2748.32758	1374.66743	916.78071	687.83735	550.47134	458.89399	26
3	412.17144	206.58936	138.06200	103.79832	83.24011	69.53464	Y-Trioxidation	2635.24352	1318.12540	879.08602	659.56634	527.85453	440.04665	25
4	499.20347	250.10537	167.07267	125.55632	100.64652	84.03998	S	2424.19545	1212.60136	808.73667	606.80432	485.64491	404.87197	24
5	612.28753	306.64741	204.76736	153.82734	123.26333	102.88732	L	2337.16342	1169.08535	779.72599	585.04631	468.23851	390.36663	23
6	669.30900	335.15814	223.77452	168.08271	134.66762	112.39090	G	2224.07936	1112.54332	742.03130	556.77530	445.62169	371.51929	22
7	726.33046	363.66887	242.78167	182.33807	146.07191	121.89447	G	2167.05789	1084.03258	723.02415	542.51993	434.21740	362.01571	21
8	825.39887	413.20308	275.80448	207.10518	165.88560	138.40588	V	2110.03643	1055.52185	704.01699	528.26456	422.81311	352.51214	20
9	981.49999	491.25363	327.83818	246.13045	197.10582	164.42273	R	2010.96801	1005.98765	670.99419	503.49746	402.99942	336.00073	19
10	1068.53201	534.76965	356.84886	267.88846	214.51222	178.92807	S	1854.86690	927.93709	618.96049	464.47218	371.77920	309.98388	18
11	1181.61608	591.31168	394.54354	296.15948	237.12904	197.77541	L	1767.83488	884.42108	589.94981	442.71418	354.37280	295.47854	17
12	1295.65901	648.33314	432.55785	324.67021	259.93762	216.78256	N	1654.75081	827.87904	552.25512	414.44316	331.75598	276.63120	16
13	1394.72742	697.86735	465.58066	349.43731	279.75131	233.29397	V	1540.70788	770.85758	514.24081	385.93243	308.94740	257.62404	15
14	1465.76453	733.38590	489.25970	367.19659	293.95873	245.13349	A	1441.63947	721.32337	481.21801	361.16533	289.13372	241.11264	14
15	1552.79656	776.90192	518.27037	388.95460	311.36513	259.63882	S	1370.60236	685.80482	457.53897	343.40605	274.92629	229.27312	13
16	1609.81803	805.41265	537.27753	403.20996	322.76943	269.14240	G	1283.57033	642.28880	428.52829	321.64804	257.51989	214.76779	12
17	1696.85005	848.92867	566.28820	424.96797	340.17583	283.64774	S	1226.54886	613.77807	409.52114	307.39267	246.11559	205.26421	11
18	1753.87152	877.43940	585.29536	439.22334	351.58012	293.15132	G	1139.51684	570.26206	380.51046	285.63467	228.70919	190.75887	10
19	1995.99882	998.50305	666.00446	499.75516	400.00559	333.50587	K-Lederers pentosone	1082.49537	541.75132	361.50331	271.37930	217.30490	181.25529	9
20	2083.03085	1042.01906	695.01513	521.51317	417.41199	348.01121	S	840.36807	420.68767	280.79421	210.84747	168.87944	140.90074	8
21	2140.05231	1070.52979	714.02229	535.76854	428.81628	357.51478	G	753.33604	377.17166	251.78353	189.08947	151.47303	126.39540	7
22	2197.07378	1099.04053	733.02944	550.02390	440.22058	367.01836	G	696.31458	348.66093	232.77638	174.83410	140.06874	116.89183	6
23	2360.13710	1180.57219	787.38389	590.78973	472.83324	394.19558	Y	639.29311	320.15020	213.76922	160.57874	128.66444	107.38825	5
24	2417.15857	1209.08292	806.39104	605.04510	484.23753	403.69916	G	476.22979	238.61853	159.41478	119.81290	96.05178	80.21103	4
25	2564.22698	1282.61713	855.41385	641.81220	513.65122	428.21056	F	419.20832	210.10780	140.40763	105.55754	84.64749	70.70745	3
26	2621.24845	1311.12786	874.42100	656.06757	525.05551	437.71414	G	272.13991	136.57359	91.38482	68.79043	55.23380	46.19605	2
27							R-Glarg	215.11844	108.06286	72.37767	54.53507	43.82951	36.69247	1

Sequence: DANNLASEVKYK, Y11-Oxidation (15.99492 Da), K12-Fructosyl (162.05354 Da)
Charge: +3, Monoisotopic m/z: 510.58252 Da (-0.22 mmu/-0.44 ppm), MH+: 1529.73301 Da, RT: 36.4609 min,
Identified with: Sequest HT (v1.17); XCorr:0.97, Percolator q-Value:5.9e-3, Percolator PEP:3.6e-2,
Fragment match tolerance used for search: 0.02 Da
Fragments used for search: -H_2O; y; -NH_3; y; b; b; -H_2O; b; -NH_3; y

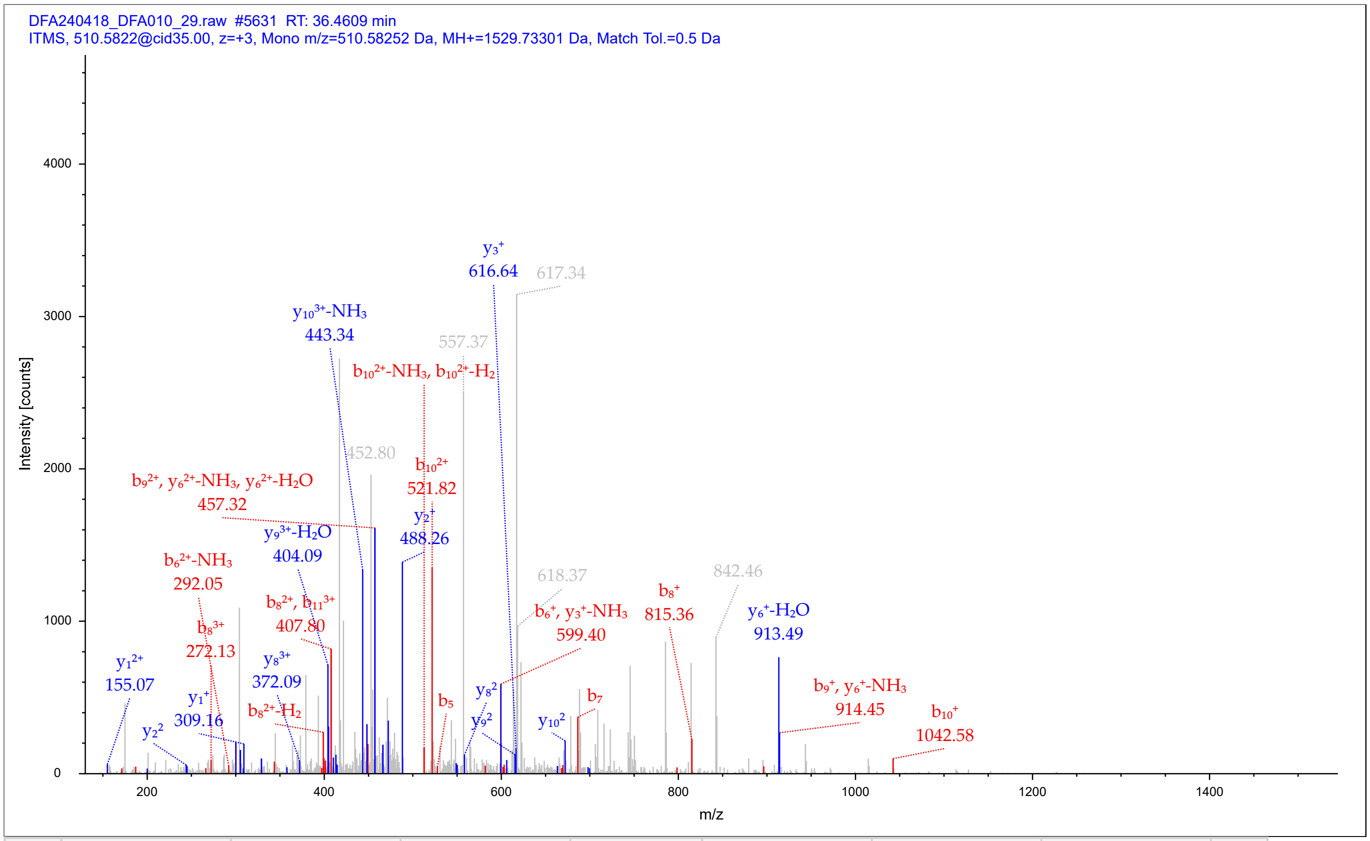

#1	b⁺	b²⁺	b³⁺	Seq.	y⁺	y²⁺	y³⁺	#2
1	116.03422	58.52075	39.34959	D				12
2	187.07133	94.03930	63.02863	A	1414.70673	707.85700	472.24043	11
3	301.11426	151.06077	101.04294	N	1343.66962	672.33845	448.56139	10
4	415.15719	208.08223	139.05725	N	1229.62669	615.31698	410.54708	9
5	528.24125	264.62426	176.75194	L	1115.58376	558.29552	372.53277	8
6	599.27837	300.14282	200.43097	A	1002.49970	501.75349	334.83808	7
7	686.31039	343.65884	229.44165	S	931.46259	466.23493	311.15905	6
8	815.35299	408.18013	272.45585	E	844.43056	422.71892	282.14837	5
9	914.42140	457.71434	305.47865	V	715.38796	358.19762	239.13417	4
10	1042.51636	521.76182	348.17697	K	616.31955	308.66341	206.11137	3
11	1221.57461	611.29094	407.86305	Y-Oxidation	488.22459	244.61593	163.41305	2
12				K-Fructosyl	309.16634	155.08681	103.72697	1

1

Sequence: AADDTWEPFASGK, W6-Trp->Kynurenin (3.99492 Da), K13-Formyl (27.99492 Da)
Charge: +2, Monoisotopic m/z: 713.80786 Da (-1.84 mmu/-2.57 ppm), MH+: 1426.60845 Da, RT: 47.8607 min,
Identified with: Sequest HT (v1.17); XCorr:1.06, Percolator q-Value:4.4e-2, Percolator PEP:1.6e-1,
Fragment match tolerance used for search: 0.02 Da
Fragments used for search: -H_2O; y; b; b; -H_2O; y

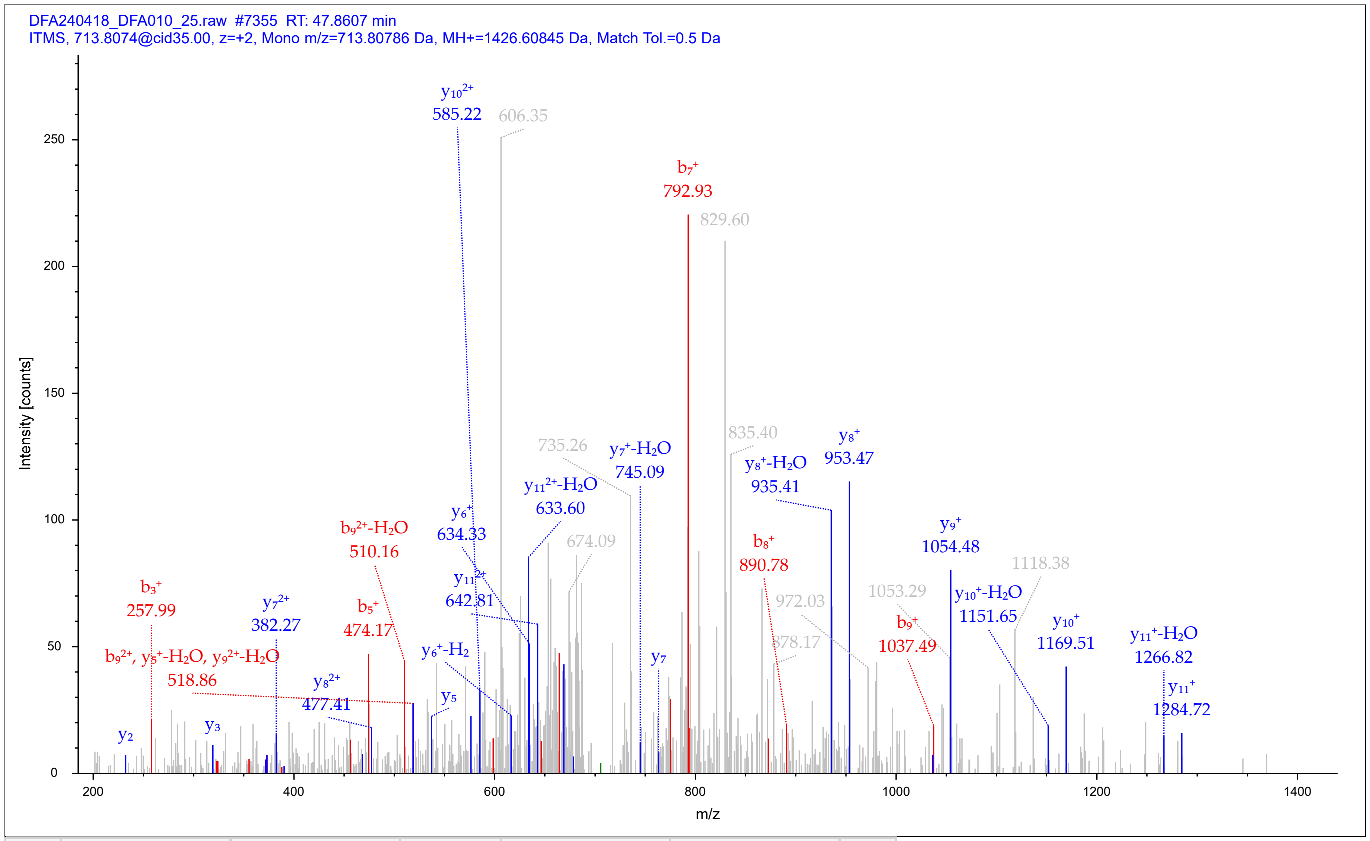

#1	b⁺	b²⁺	Seq.	y⁺	y²⁺	#2
1	72.04439	36.52583	A			13
2	143.08150	72.04439	A	1355.57500	678.29114	12
3	258.10845	129.55786	D	1284.53789	642.77258	11
4	373.13539	187.07133	D	1169.51094	585.25911	10
5	474.18307	237.59517	T	1054.48400	527.74564	9
6	664.25730	332.63229	W-Trp-> Kynurenin	953.43632	477.22180	8
7	793.29989	397.15358	E	763.36210	382.18469	7
8	890.35265	445.67996	P	634.31950	317.66339	6
9	1037.42107	519.21417	F	537.26674	269.13701	5
10	1108.45818	554.73273	A	390.19833	195.60280	4
11	1195.49021	598.24874	S	319.16121	160.08424	3
12	1252.51167	626.75947	G	232.12918	116.56823	2
13			K-Formyl	175.10772	88.05750	1

Sequence: LWTGGLDNTVRSWDLR, W2-Trp->Hydroxykynurenin (19.98983 Da), W13-Trp->Oxolactone (13.97927 Da), R11-Delta:H(2)C(3)O(1) (54.01057 Da)
Charge: +3, Monoisotopic m/z: 659.65552 Da (+1.98 mmu/+2.99 ppm), MH+: 1976.95200 Da, RT: 66.7030 min,
Identified with: Sequest HT (v1.17); XCorr:0.85, Percolator q-Value:5.5e-3, Percolator PEP:3.3e-2,
Fragment match tolerance used for search: 0.02 Da
Fragments used for search: -H_2O; y; -NH_3; y; b; b; -H_2O; b; -NH_3; y

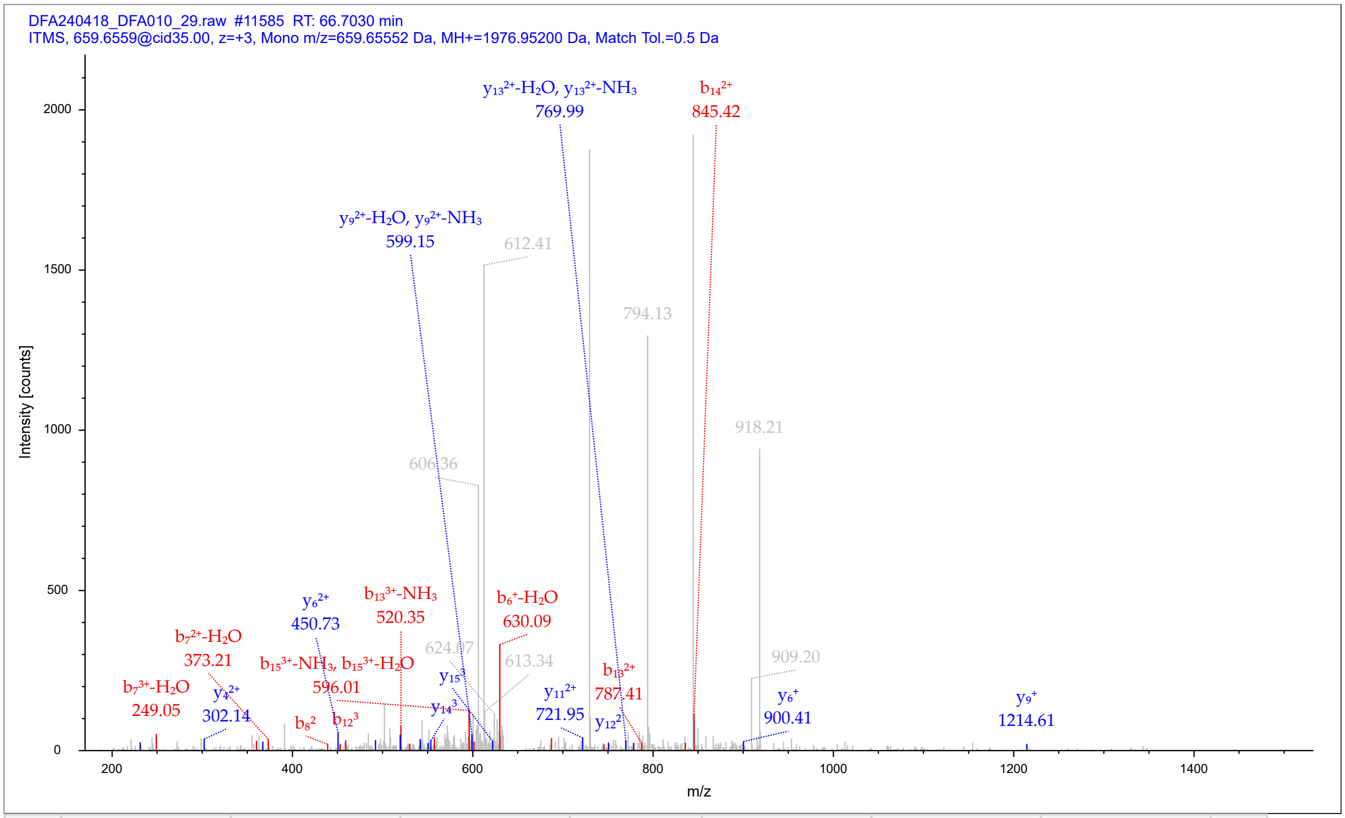

#1	b⁺	b²⁺	b³⁺	Seq.	y⁺	y²⁺	y³⁺	#2
1	114.09134	57.54931	38.70196	L				16
2	320.16048	160.58388	107.39168	W-Trp->Hydroxy-kynurenin	1863.86202	932.43465	621.95886	15
3	421.20816	211.10772	141.07424	T	1657.79287	829.40008	553.26914	14
4	478.22962	239.61845	160.08139	G	1556.74520	778.87624	519.58658	13
5	535.25109	268.12918	179.08855	G	1499.72373	750.36550	500.57943	12
6	648.33515	324.67121	216.78324	L	1442.70227	721.85477	481.57227	11
7	763.36210	382.18469	255.12555	D	1329.61820	665.31274	443.87759	10
8	877.40502	439.20615	293.13986	N	1214.59126	607.79927	405.53527	9
9	978.45270	489.72999	326.82242	T	1100.54833	550.77781	367.52096	8
10	1077.52111	539.26420	359.84522	V	999.50066	500.25397	333.83840	7
11	1287.63279	644.32003	429.88245	R-Delta: H(2)C(3)O(1)	900.43224	450.71976	300.81560	6
12	1374.66482	687.83605	458.89312	S	690.32057	345.66392	230.77837	5
13	1574.72340	787.86534	525.57932	W-Trp->Oxo-lactone	603.28854	302.14791	201.76770	4
14	1689.75034	845.37881	563.92163	D	403.22996	202.11862	135.08150	3
15	1802.83440	901.92084	601.61632	L	288.20302	144.60515	96.73919	2
16				R	175.11895	88.06311	59.04450	1

Sequence: DLRFIPDPKAGIMIK, F4-Oxidation (15.99492 Da), K15-3-deoxyglucosone intermediate 2 (126.03170 Da)
Charge: +2, Monoisotopic m/z: 928.50006 Da (-2.9 mmu/-3.12 ppm), MH+: 1855.99285 Da, RT: 74.4345 min, Identified with: Sequest HT (v1.17); XCorr:0.46, Percolator q-Value:5.3e-3, Percolator PEP:1.6e-2,
Fragment match tolerance used for search: 0.02 Da
Fragments used for search: $-H_2O$; y; $-NH_3$; y; b; b; $-H_2O$; b; $-NH_3$; y

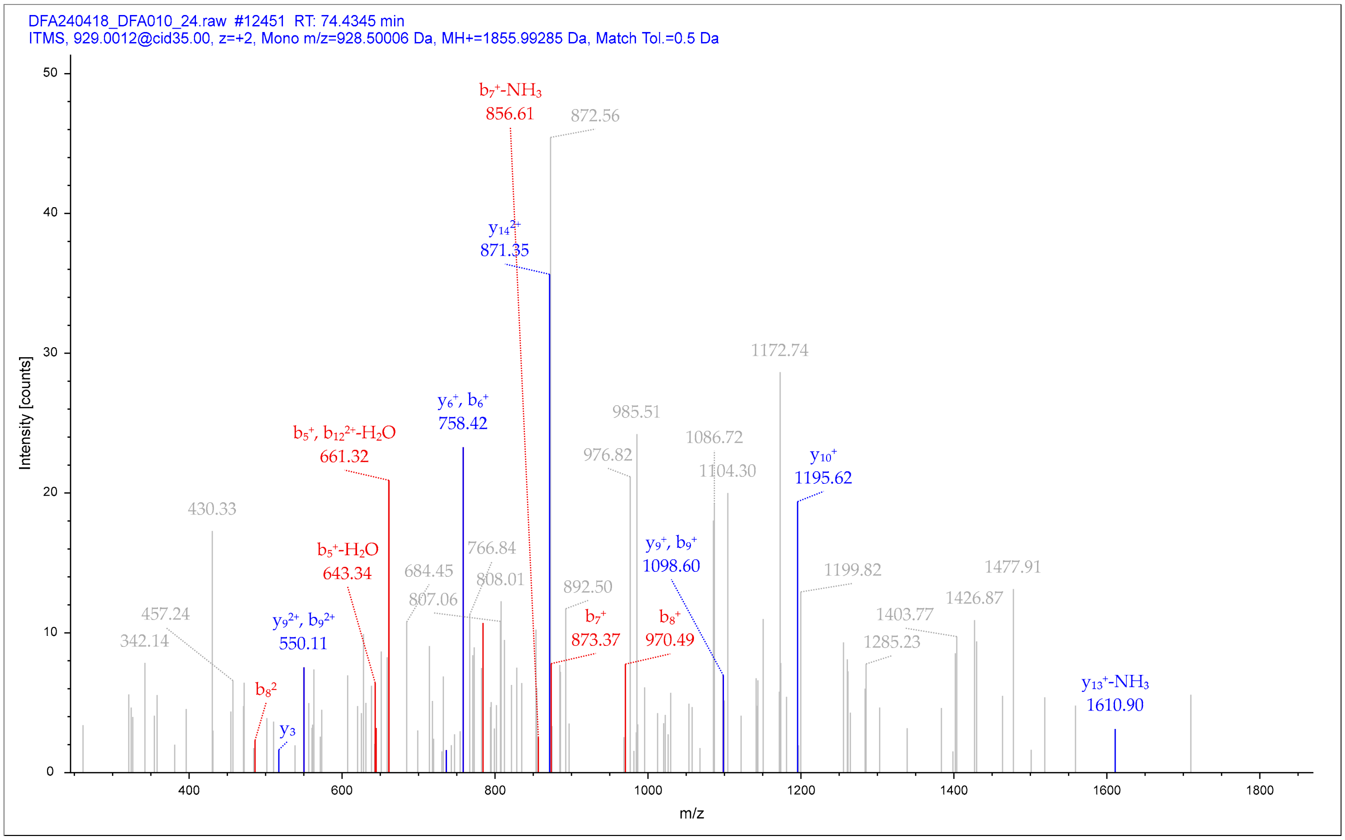

#1	b⁺	b²⁺	Seq.	y⁺	y²⁺	#2
1	116.03422	58.52075	D			15
2	229.11828	115.06278	L	1740.97170	870.98949	14
3	385.21939	193.11334	R	1627.88763	814.44745	13
4	548.28272	274.64500	F-Oxidation	1471.78652	736.39690	12
5	661.36679	331.18703	I	1308.72319	654.86523	11
6	758.41955	379.71341	P	1195.63913	598.32320	10
7	873.44649	437.22689	D	1098.58636	549.79682	9
8	970.49926	485.75327	P	983.55942	492.28335	8
9	1098.59422	549.80075	K	886.50666	443.75697	7
10	1169.63133	585.31931	A	758.41169	379.70949	6
11	1226.65280	613.83004	G	687.37458	344.19093	5
12	1339.73686	670.37207	I	630.35312	315.68020	4
13	1470.77735	735.89231	M	517.26905	259.13816	3
14	1583.86141	792.43434	I	386.22857	193.61792	2
15			K-3-deoxy-glucosone intermediate 2	273.14450	137.07589	1

5

Sequence: AEVIWTSSDHQVLSGK, W5-Trp->Hydroxykynurenin (19.98983 Da), K16-Triosyl (72.01840 Da)
Charge: +2, Monoisotopic m/z: 924.94318 Da (-7.8 mmu/-8.43 ppm), MH+: 1848.87908 Da, RT: 90.4011 min,
Identified with: Sequest HT (v1.17); XCorr:0.70, Percolator q-Value:7.1e-3, Percolator PEP:2.7e-2,
Fragment match tolerance used for search: 0.02 Da
Fragments used for search: -H_2O; y; -NH_3; y; b; b; -H_2O; b; -NH_3; y

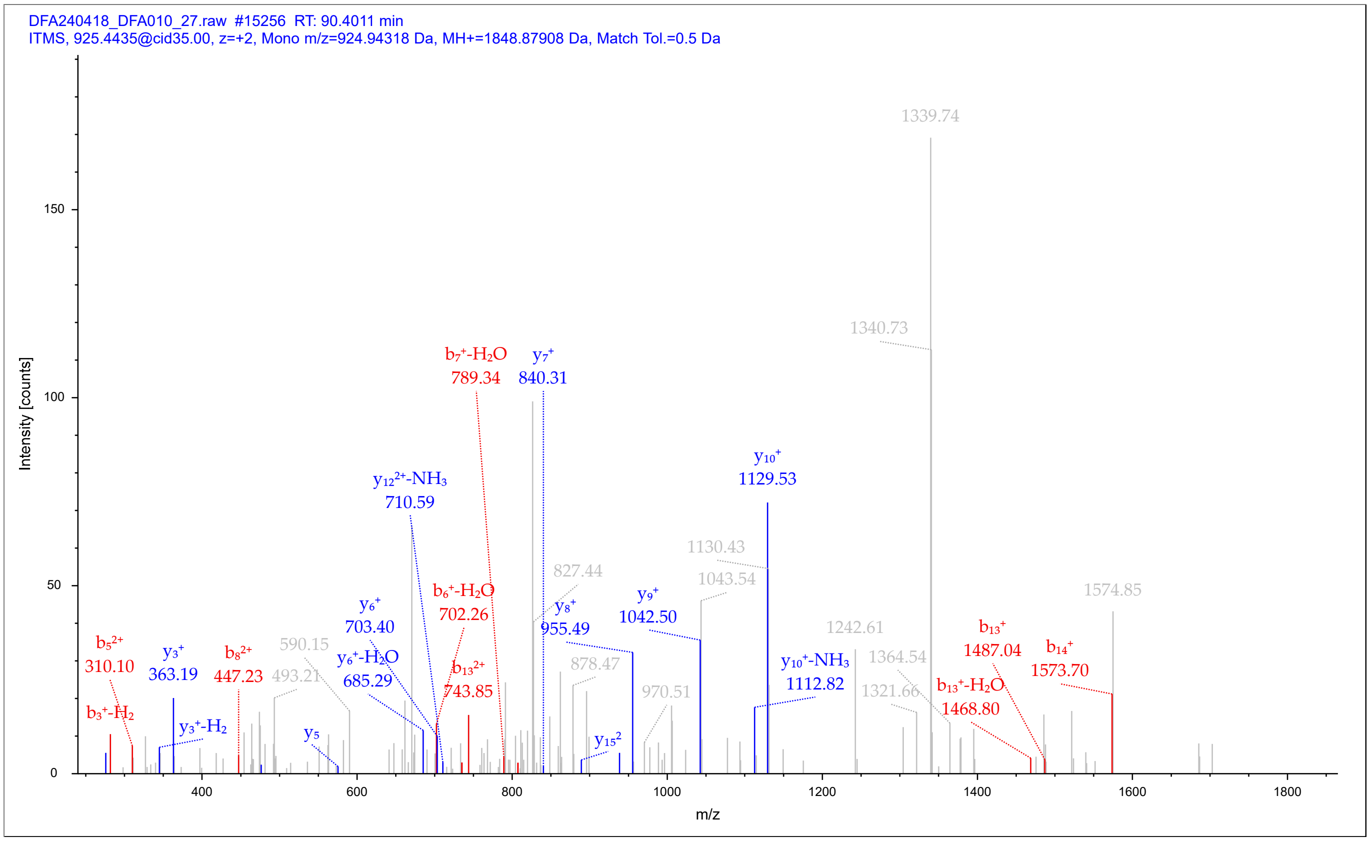

#1	b⁺	b²⁺	Seq.	y⁺	y²⁺	#2
1	72.04439	36.52583	A			16
2	201.08698	101.04713	E	1777.85755	889.43242	15
3	300.15540	150.58134	V	1648.81496	824.91112	14
4	413.23946	207.12337	I	1549.74655	775.37691	13
5	619.30860	310.15794	W-Trp->Hydroxy kynurenin	1436.66248	718.83488	12
6	720.35628	360.68178	T	1230.59334	615.80031	11
7	807.38831	404.19779	S	1129.54566	565.27647	10
8	894.42034	447.71381	S	1042.51363	521.76046	9
9	1009.44728	505.22728	D	955.48161	478.24444	8
10	1146.50619	573.75673	H	840.45466	420.73097	7
11	1274.56477	637.78602	Q	703.39575	352.20151	6
12	1373.63318	687.32023	V	575.33717	288.17223	5
13	1486.71725	743.86226	L	476.26876	238.63802	4
14	1573.74928	787.37828	S	363.18470	182.09599	3
15	1630.77074	815.88901	G	276.15267	138.57997	2
16			K-Triosyl	219.13120	110.06924	1

Sequence: LSKDLDRPESQSPK, K14-C4-amide (118.02724 Da), R7-Glarg (39.99949 Da)
Charge: +2, Monoisotopic m/z: 879.43378 Da (-0.07 mmu/-0.07 ppm), MH+: 1757.86028 Da, RT: 90.6152 min,
Identified with: Sequest HT (v1.17); XCorr:0.97, Percolator q-Value:5.9e-3, Percolator PEP:3.5e-2,
Fragment match tolerance used for search: 0.02 Da
Fragments used for search: $-H_2O$; y; $-NH_3$; y; b; b; $-H_2O$; b; $-NH_3$; y

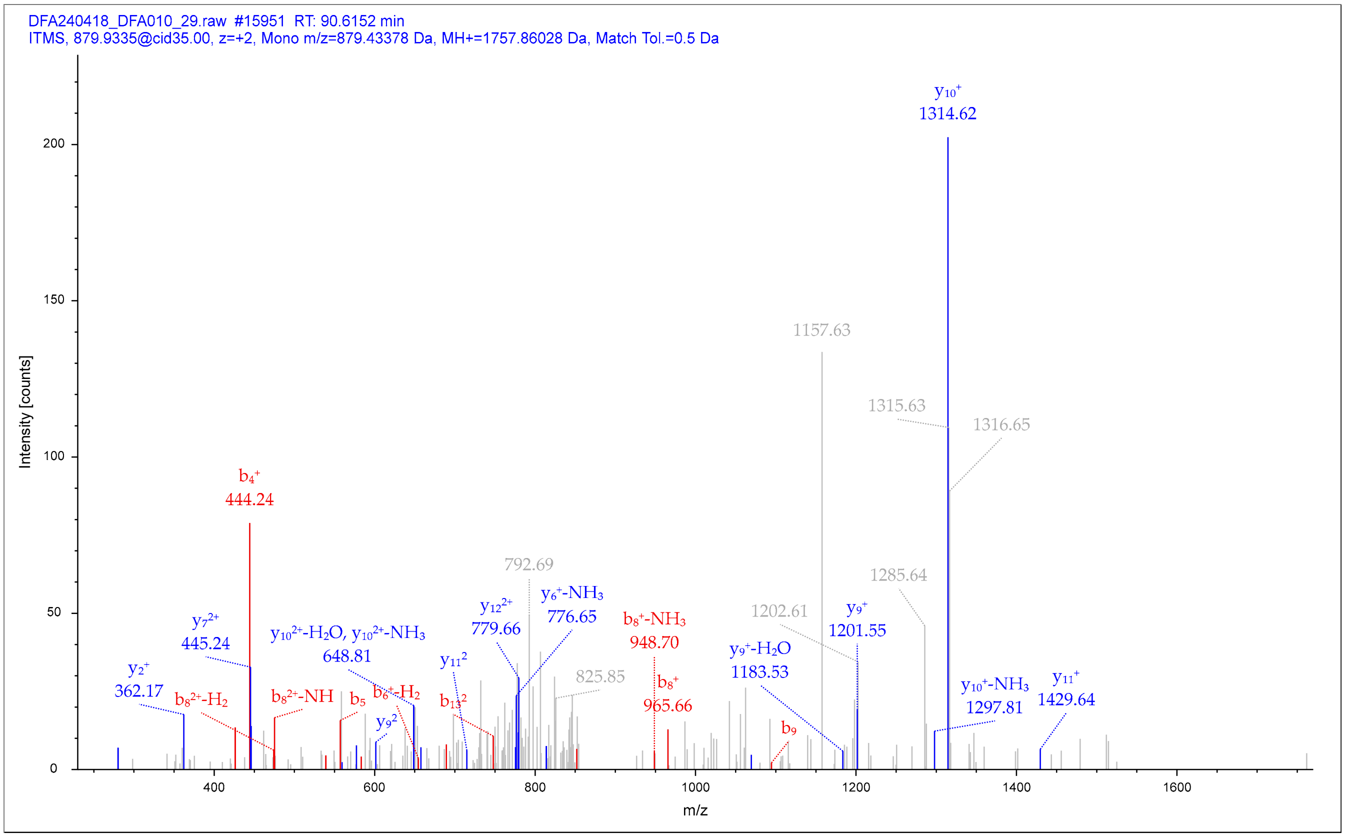

#1	b⁺	b²⁺	Seq.	y⁺	y²⁺	#2
1	114.09134	57.54931	L			14
2	201.12337	101.06532	S	1644.77634	822.89181	13
3	329.21833	165.11280	K	1557.74431	779.37580	12
4	444.24527	222.62628	D	1429.64935	715.32831	11
5	557.32934	279.16831	L	1314.62241	657.81484	10
6	672.35628	336.68178	D	1201.53834	601.27281	9
7	868.45688	434.73208	R-Glarg	1086.51140	543.75934	8
8	965.50965	483.25846	P	890.41080	445.70904	7
9	1094.55224	547.77976	E	793.35804	397.18266	6
10	1181.58427	591.29577	S	664.31544	332.66136	5
11	1309.64285	655.32506	Q	577.28341	289.14535	4
12	1396.67488	698.84108	S	449.22484	225.11606	3
13	1493.72764	747.36746	P	362.19281	181.60004	2
14			K-C4-amide	265.14004	133.07366	1

Sequence: NQLNKETDPVK, K11-C5-amide (148.03784 Da)
Charge: +3, Monoisotopic m/z: 478.57593 Da (+0.25 mmu/+0.51 ppm), MH+: 1433.71323 Da, RT: 23.0981 min,
Identified with: Sequest HT (v1.17); XCorr:0.52, Percolator q-Value:4.3e-3, Percolator PEP:2.2e-2,
Fragment match tolerance used for search: 0.02 Da
Fragments used for search: -H_2O; y; -NH_3; y; b; b; -H_2O; b; -NH_3; y

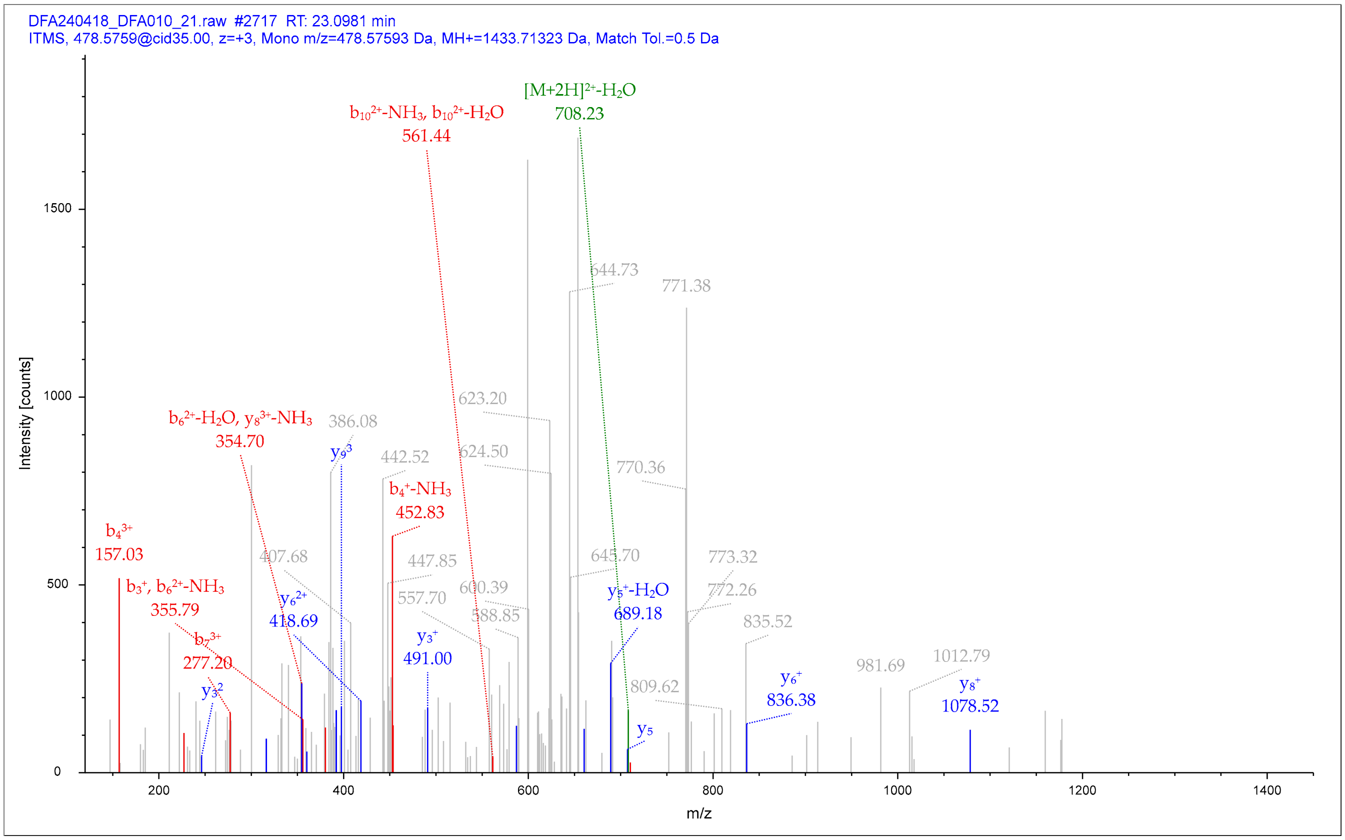

#1	b⁺	b²⁺	b³⁺	Seq.	y⁺	y²⁺	y³⁺	#2
1	115.05020	58.02874	39.02159	N				11
2	243.10878	122.05803	81.70778	Q	1319.66957	660.33842	440.56137	10
3	356.19285	178.60006	119.40247	L	1191.61099	596.30913	397.87518	9
4	470.23577	235.62152	157.41678	N	1078.52693	539.76710	360.18049	8
5	598.33074	299.66901	200.11510	K	964.48400	482.74564	322.16618	7
6	727.37333	364.19030	243.12929	E	836.38904	418.69816	279.46786	6
7	828.42101	414.71414	276.81185	T	707.34644	354.17686	236.45367	5
8	943.44795	472.22761	315.15417	D	606.29876	303.65302	202.77111	4
9	1040.50071	520.75400	347.50509	P	491.27182	246.13955	164.42879	3
10	1139.56913	570.28820	380.52789	V	394.21906	197.61317	132.07787	2
11				K-C5-amide	295.15064	148.07896	99.05507	1

Sequence: WLNGLENAPQK, W1-Oxidation (15.99492 Da), K11-Ethanalyl (42.01830 Da)
Charge: +2, Monoisotopic m/z: 664.33960 Da (+0.05 mmu/+0.07 ppm), MH+: 1327.67192 Da, RT: 51.2875 min,
Identified with: Sequest HT (v1.17); XCorr:0.63, Percolator q-Value:3.6e-3, Percolator PEP:1.2e-2,
Fragment match tolerance used for search: 0.02 Da
Fragments used for search: -H_2O; y; -NH_3; y; b; b; -H_2O; b; -NH_3; y

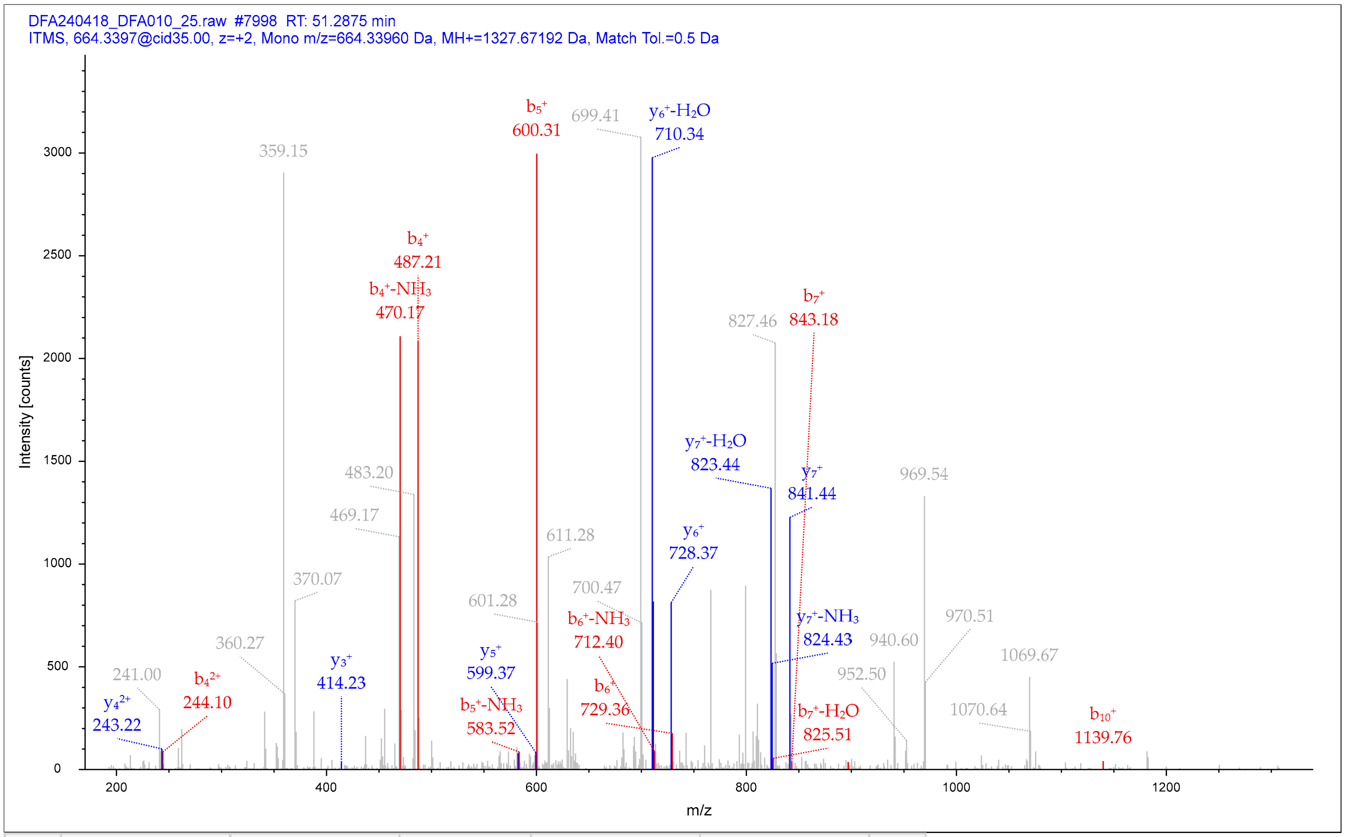

#1	b⁺	b²⁺	Seq.	y⁺	y²⁺	#2
1	203.08150	102.04439	W-Oxidation			11
2	316.16557	158.58642	L	1125.59760	563.30244	10
3	430.20850	215.60789	N	1012.51353	506.76041	9
4	487.22996	244.11862	G	898.47061	449.73894	8
5	600.31402	300.66065	L	841.44914	421.22821	7
6	729.35662	365.18195	E	728.36508	364.68618	6
7	843.39954	422.20341	N	599.32249	300.16488	5
8	914.43666	457.72197	A	485.27956	243.14342	4
9	1011.48942	506.24835	P	414.24245	207.62486	3
10	1139.54800	570.27764	Q	317.18968	159.09848	2
11			K-Ethanalyl	189.13110	95.06919	1

97_98_MGH

Sequence: NVLQMTWMLPRPK, R11-Delta:H(2)C(3)O(1) (54.01057 Da), K13-Triosyl (72.01840 Da)
Charge: +3, Monoisotopic m/z: 580.63690 Da (+0.58 mmu/+1 ppm), MH+: 1739.89615 Da, RT: 53.9106 min,
Identified with: Sequest HT (v1.17); XCorr:1.01, Percolator q-Value:3.5e-3, Percolator PEP:1.3e-2,
Fragment match tolerance used for search: 0.02 Da
Fragments used for search: -H_2O; y; -NH_3; y; b; b; -H_2O; b; -NH_3; y

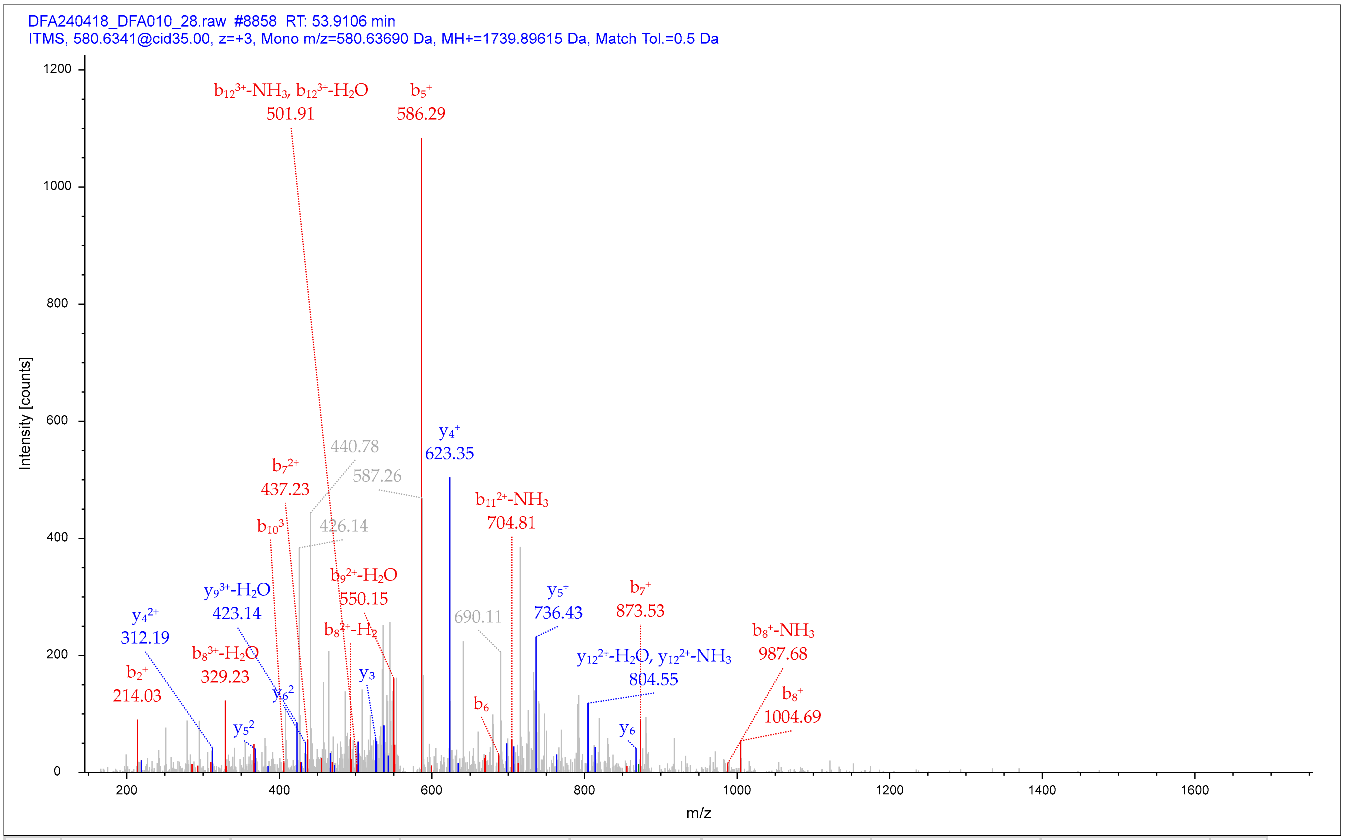

#1	b⁺	b²⁺	b³⁺	Seq.	y⁺	y²⁺	y³⁺	#2
1	115.05020	58.02874	39.02159	N				13
2	214.11862	107.56295	72.04439	V	1625.85149	813.42938	542.62201	12
3	327.20268	164.10498	109.73908	L	1526.78307	763.89518	509.59921	11
4	455.26126	228.13427	152.42527	Q	1413.69901	707.35314	471.90452	10
5	586.30174	293.65451	196.10543	M	1285.64043	643.32385	429.21833	9
6	687.34942	344.17835	229.78799	T	1154.59995	577.80361	385.53817	8
7	873.42874	437.21801	291.81443	W	1053.55227	527.27977	351.85561	7
8	1004.46922	502.73825	335.49459	M	867.47296	434.24012	289.82917	6
9	1117.55328	559.28028	373.18928	L	736.43247	368.71987	246.14901	5
10	1214.60605	607.80666	405.54020	P	623.34841	312.17784	208.45432	4
11	1424.71772	712.86250	475.57743	R-Delta:H(2)C(3)O(1)	526.29564	263.65146	176.10340	3
12	1521.77049	761.38888	507.92835	P	316.18397	158.59562	106.06617	2
13				K-Triosyl	219.13120	110.06924	73.71525	1

97_98_Glarg

Sequence: NVLQMTWMLPRPK, K13-3-deoxyglucosone intermediate 2 (126.03170 Da)
Charge: +3, Monoisotopic m/z: 580.63690 Da (-0.33 mmu/-0.57 ppm), MH+: 1739.89615 Da, RT: 53.9106 min,
Identified with: Sequest HT (v1.17); XCorr:0.69, Percolator q-Value:5.2e-3, Percolator PEP:3.6e-2,
Fragment match tolerance used for search: 0.02 Da
Fragments used for search: -H_2O; y; -NH_3; y; b; b; -H_2O; b; -NH_3; y

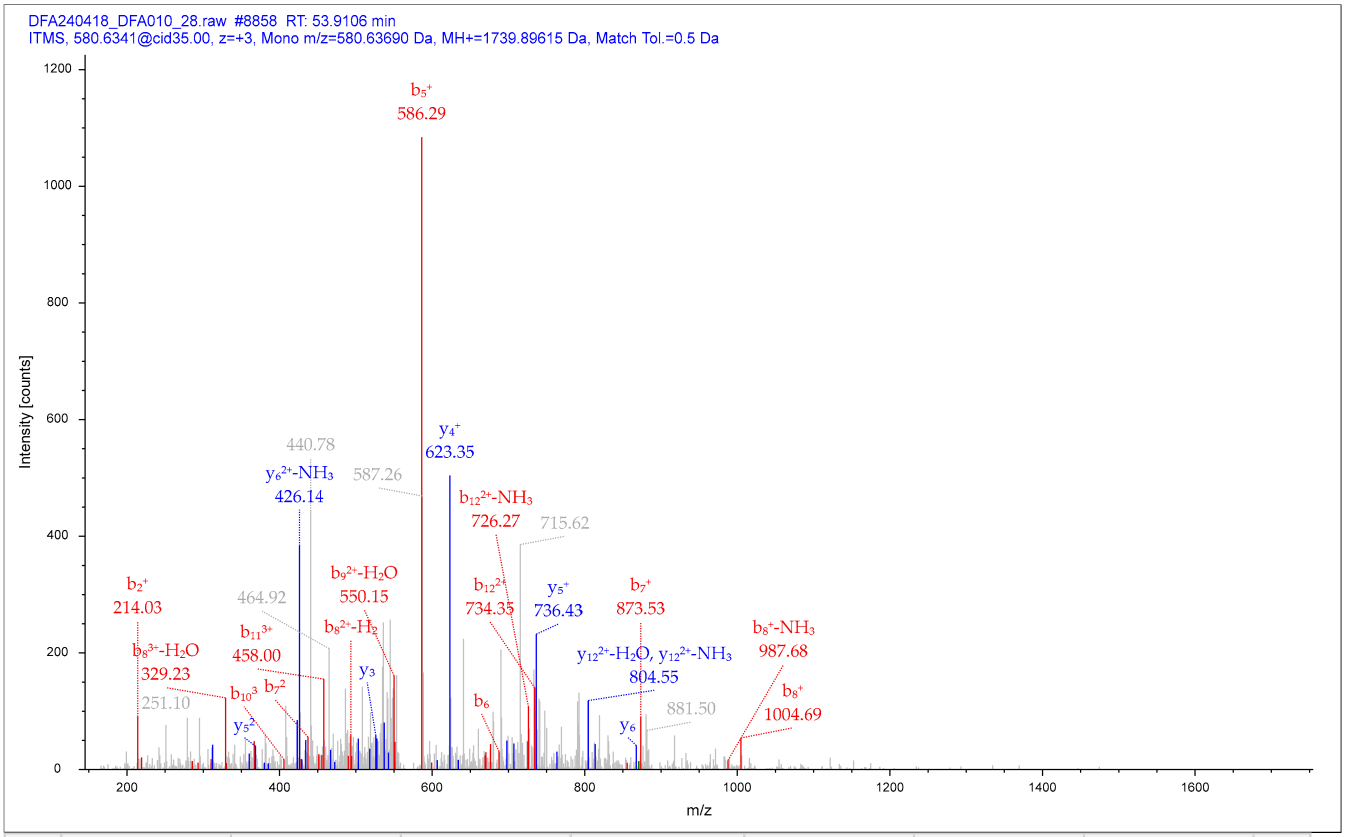

#1	b+	b2+	b3+	Seq.	y+	y2+	y3+	#2
1	115.05020	58.02874	39.02159	N				13
2	214.11862	107.56295	72.04439	V	1625.85422	813.43075	542.62293	12
3	327.20268	164.10498	109.73908	L	1526.78581	763.89654	509.60012	11
4	455.26126	228.13427	152.42527	Q	1413.70174	707.35451	471.90543	10
5	586.30174	293.65451	196.10543	M	1285.64317	643.32522	429.21924	9
6	687.34942	344.17835	229.78799	T	1154.60268	577.80498	385.53908	8
7	873.42874	437.21801	291.81443	W	1053.55500	527.28114	351.85652	7
8	1004.46922	502.73825	335.49459	M	867.47569	434.24148	289.83008	6
9	1117.55328	559.28028	373.18928	L	736.43521	368.72124	246.14992	5
10	1214.60605	607.80666	405.54020	P	623.35114	312.17921	208.45523	4
11	1370.70716	685.85722	457.57390	R	526.29838	263.65283	176.10431	3
12	1467.75992	734.38360	489.92483	P	370.19727	185.60227	124.07061	2
13				K-3-deoxyglucosone intermediate 2	273.14450	137.07589	91.71969	1

115

Sequence: RVYQCNICSK, C5-Carbamidomethyl (57.02146 Da), C8-Carbamidomethyl (57.02146 Da), K10-Carboxyethyl (72.02113 Da), R1-Pentosyl (132.03955 Da)
Charge: +2, Monoisotopic m/z: 766.34650 Da (+0.24 mmu/+0.32 ppm), MH+: 1531.68572 Da, RT: 90.9431 min, Identified with: Sequest HT (v1.17); XCorr:0.70, Percolator q-Value:4.1e-3, Percolator PEP:2.1e-2, ptmRS: Best Site Probabilities:K10(Carboxyethyl): 100,
Fragment match tolerance used for search: 0.02 Da
Fragments used for search: -H_2O; y; -NH_3; y; b; b; -H_2O; b; -NH_3; y

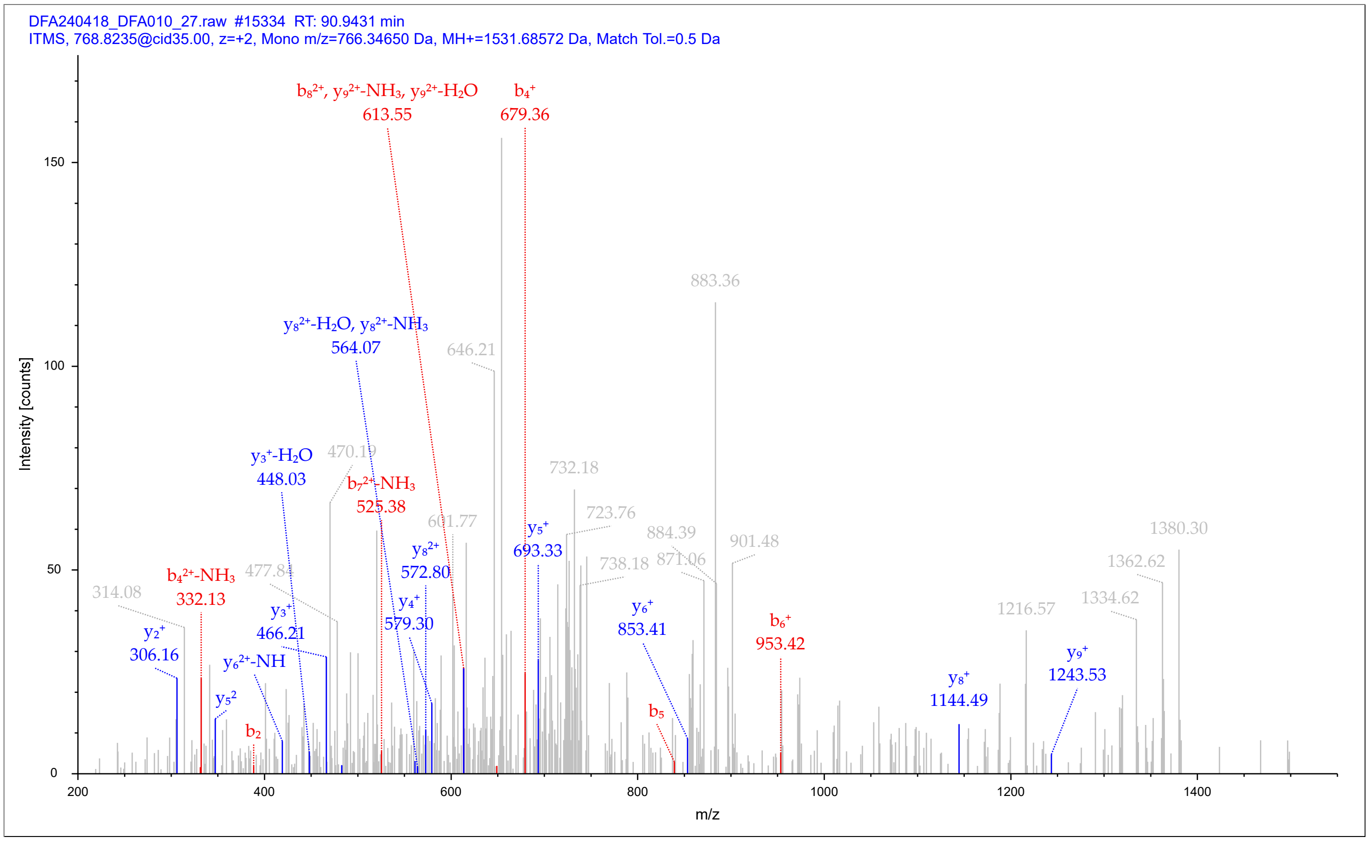

#1	b⁺	b²⁺	Seq.	y⁺	y²⁺	#2
1	289.14794	145.07761	R-Pentosyl			10
2	388.21635	194.61181	V	1243.54457	622.27592	9
3	551.27968	276.14348	Y	1144.47616	572.74172	8
4	679.33826	340.17277	Q	981.41283	491.21005	7
5	839.36891	420.18809	C-Carbamido-methyl	853.35425	427.18076	6
6	953.41183	477.20955	N	693.32360	347.16544	5
7	1066.49590	533.75159	I	579.28067	290.14398	4
8	1226.52655	613.76691	C-Carbamido-methyl	466.19661	233.60194	3
9	1313.55857	657.28293	S	306.16596	153.58662	2
10			K-Carboxy-ethyl	219.13393	110.07060	1

Sequence: YAHLSEHYR, R9-Carboxymethyl (58.00548 Da), Y1-Oxidation (15.99492 Da), Y8-Trioxidation (47.98474 Da)
Charge: +3, Monoisotopic m/z: 433.18796 Da (+1.65 mmu/+3.82 ppm), MH+: 1297.54932 Da, RT: 23.8272 min,
Identified with: Sequest HT (v1.17); XCorr:0.70, Percolator q-Value:2.1e-3, Percolator PEP:1.6e-2,
Fragment match tolerance used for search: 0.02 Da
Fragments used for search: -H_2O; y; b; b; -H_2O; y

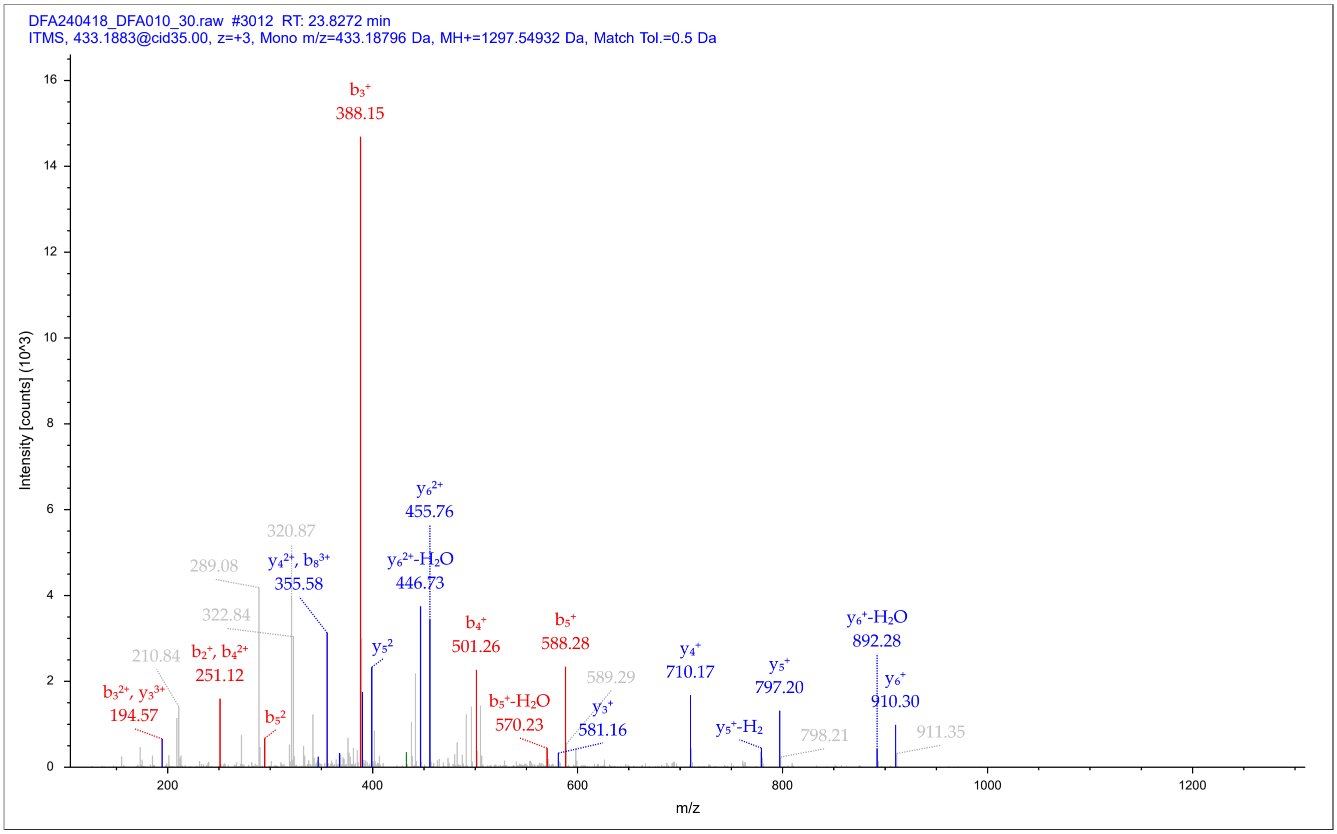

#1	b+	b2+	b3+	Seq.	y+	y2+	y3+	#2
1	180.06552	90.53640	60.69336	Y-Oxidation				9
2	251.10263	126.05496	84.37240	A	1118.48613	559.74670	373.50023	8
3	388.16155	194.58441	130.05870	H	1047.44901	524.22814	349.82119	7
4	501.24561	251.12644	167.75339	L	910.39010	455.69869	304.13488	6
5	588.27764	294.64246	196.76406	S	797.30604	399.15666	266.44020	5
6	717.32023	359.16375	239.77826	E	710.27401	355.64064	237.42952	4
7	854.37914	427.69321	285.46457	H	581.23142	291.11935	194.41532	3
8	1065.42722	533.21725	355.81392	Y-Trioxidation	444.17250	222.58989	148.72902	2
9				R-Carboxymethyl	233.12443	117.06585	78.37966	1

Sequence: SWTAADLAAQITK, K13-b-ketonium ion (96.02056 Da)
Charge: +3, Monoisotopic m/z: 491.25302 Da (+0.78 mmu/+1.59 ppm), MH+: 1471.74451 Da, RT: 34.4606 min,
Identified with: Sequest HT (v1.17); XCorr:0.44, Percolator q-Value:4.6e-3, Percolator PEP:2.4e-2,
Fragment match tolerance used for search: 0.02 Da
Fragments used for search: -H_2O; y; -NH_3; y; b; b; -H_2O; b; -NH_3; y

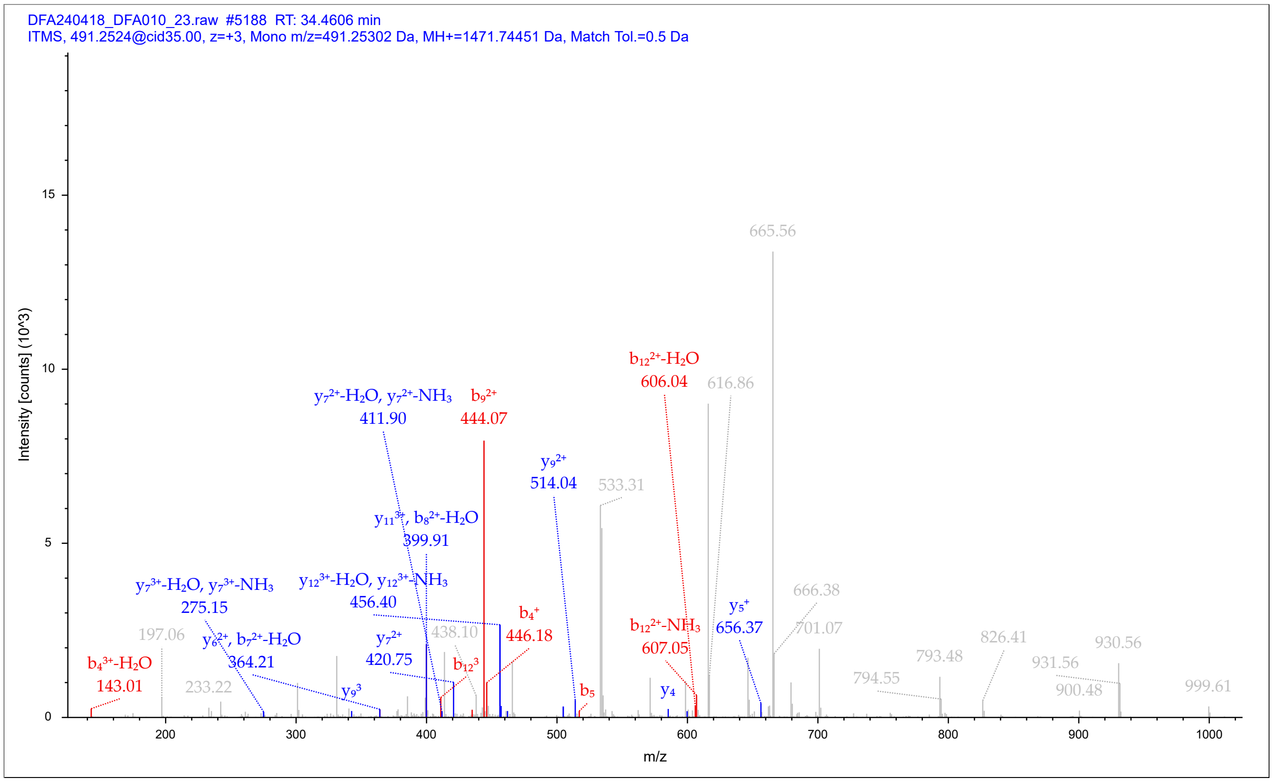

#1	b⁺	b²⁺	b³⁺	Seq.	y⁺	y²⁺	y³⁺	#2
1	88.03930	44.52329	30.01795	S				13
2	274.11862	137.56295	92.04439	W	1384.71014	692.85871	462.24156	12
3	375.16630	188.08679	125.72695	T	1198.63082	599.81905	400.21513	11
4	446.20341	223.60534	149.40599	A	1097.58315	549.29521	366.53257	10
5	517.24052	259.12390	173.08503	A	1026.54603	513.77665	342.85353	9
6	632.26747	316.63737	211.42734	D	955.50892	478.25810	319.17449	8
7	745.35153	373.17940	249.12203	L	840.48198	420.74463	280.83218	7
8	816.38864	408.69796	272.80107	A	727.39791	364.20259	243.13749	6
9	887.42576	444.21652	296.48010	A	656.36080	328.68404	219.45845	5
10	1015.48434	508.24581	339.16630	Q	585.32368	293.16548	195.77941	4
11	1128.56840	564.78784	376.86098	I	457.26511	229.13619	153.09322	3
12	1229.61608	615.31168	410.54354	T	344.18104	172.59416	115.39853	2
13				K-b-ketonium ion	243.13336	122.07032	81.71597	1

15

Sequence: CVLPPMDGYPHCEGK, C1-Carbamidomethyl (57.02146 Da), C12-Carbamidomethyl (57.02146 Da), K15-Glyoxal-imine (39.99554 Da)
Charge: +3, Monoisotopic m/z: 600.59027 Da (+0.22 mmu/+0.37 ppm), MH+: 1799.75626 Da, RT: 42.8135 min,
Identified with: Sequest HT (v1.17); XCorr:0.66, Percolator q-Value:4.8e-3, Percolator PEP:4.4e-2,
Fragment match tolerance used for search: 0.02 Da
Fragments used for search: -H_2O; y; b; b; -H_2O; y

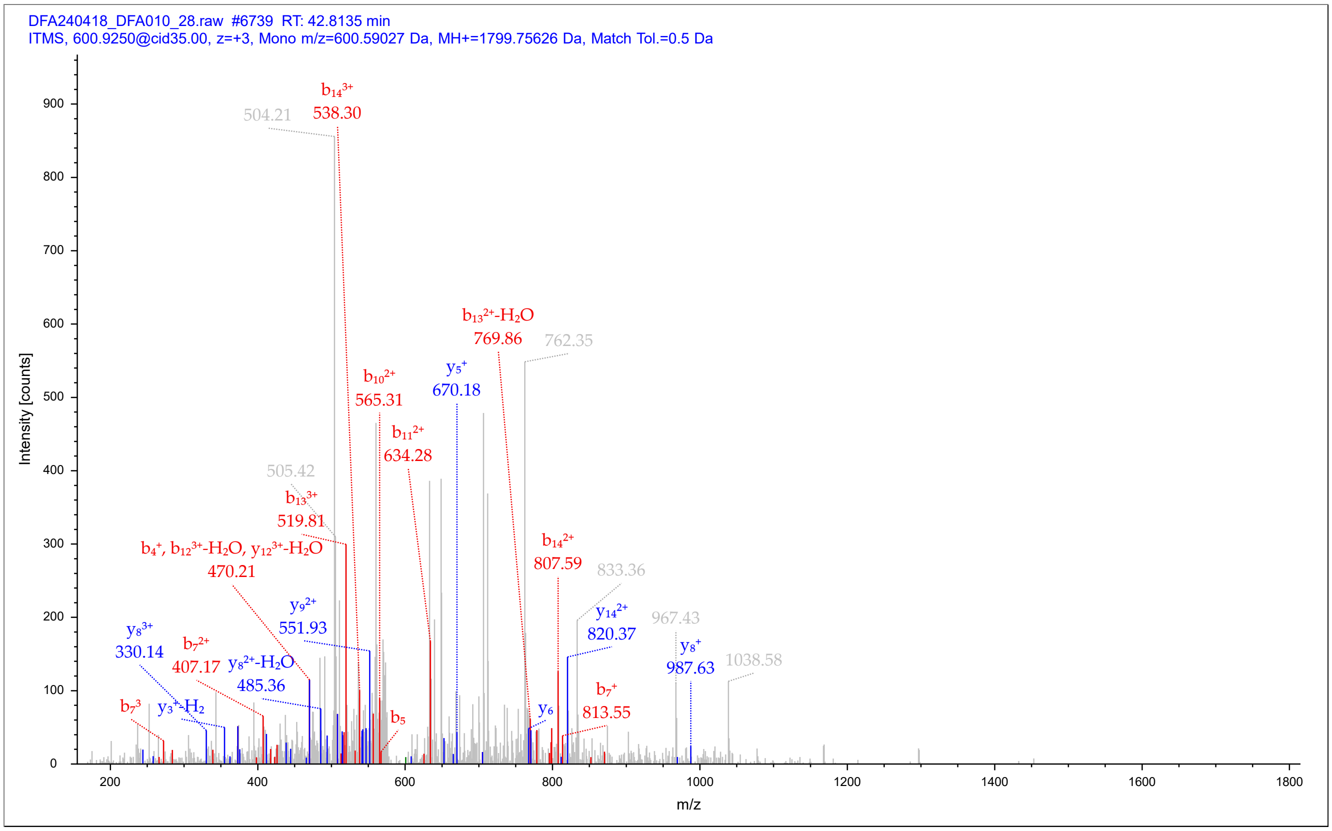

#1	b+	b2+	b3+	Seq.	y+	y2+	y3+	#2
1	161.03793	81.02260	54.35083	C-Carbamido methyl				15
2	260.10634	130.55681	87.37363	V	1639.72495	820.36611	547.24650	14
3	373.19040	187.09884	125.06832	L	1540.65654	770.83191	514.22370	13
4	470.24317	235.62522	157.41924	P	1427.57247	714.28987	476.52901	12
5	567.29593	284.15160	189.77016	P	1330.51971	665.76349	444.17809	11
6	698.33642	349.67185	233.45032	M	1233.46694	617.23711	411.82717	10
7	813.36336	407.18532	271.79264	D	1102.42646	551.71687	368.14700	9
8	870.38482	435.69605	290.79979	G	987.39952	494.20340	329.80469	8
9	1033.44815	517.22771	345.15423	Y	930.37805	465.69267	310.79754	7
10	1130.50091	565.75410	377.50516	P	767.31473	384.16100	256.44309	6
11	1267.55983	634.28355	423.19146	H	670.26196	335.63462	224.09217	5
12	1427.59047	714.29888	476.53501	C-Carbamido methyl	533.20305	267.10516	178.40587	4
13	1556.63307	778.82017	519.54921	E	373.17240	187.08984	125.06232	3
14	1613.65453	807.33090	538.55636	G	244.12981	122.56854	82.04812	2
15				K-Glyoxal-imine	187.10834	94.05781	63.04097	1

213

Sequence: VGYVSGWGR, W7-Trp->Kynurenin (3.99492 Da)
Charge: +2, Monoisotopic m/z: 492.74860 Da (+0.08 mmu/+0.16 ppm), MH+: 984.48992 Da, RT: 43.6620 min,
Identified with: Sequest HT (v1.17); XCorr:0.92, Percolator q-Value:6.3e-3, Percolator PEP:1.3e-2,
Fragment match tolerance used for search: 0.02 Da
Fragments used for search: -H_2O; y; -NH_3; y; b; b; -H_2O; y

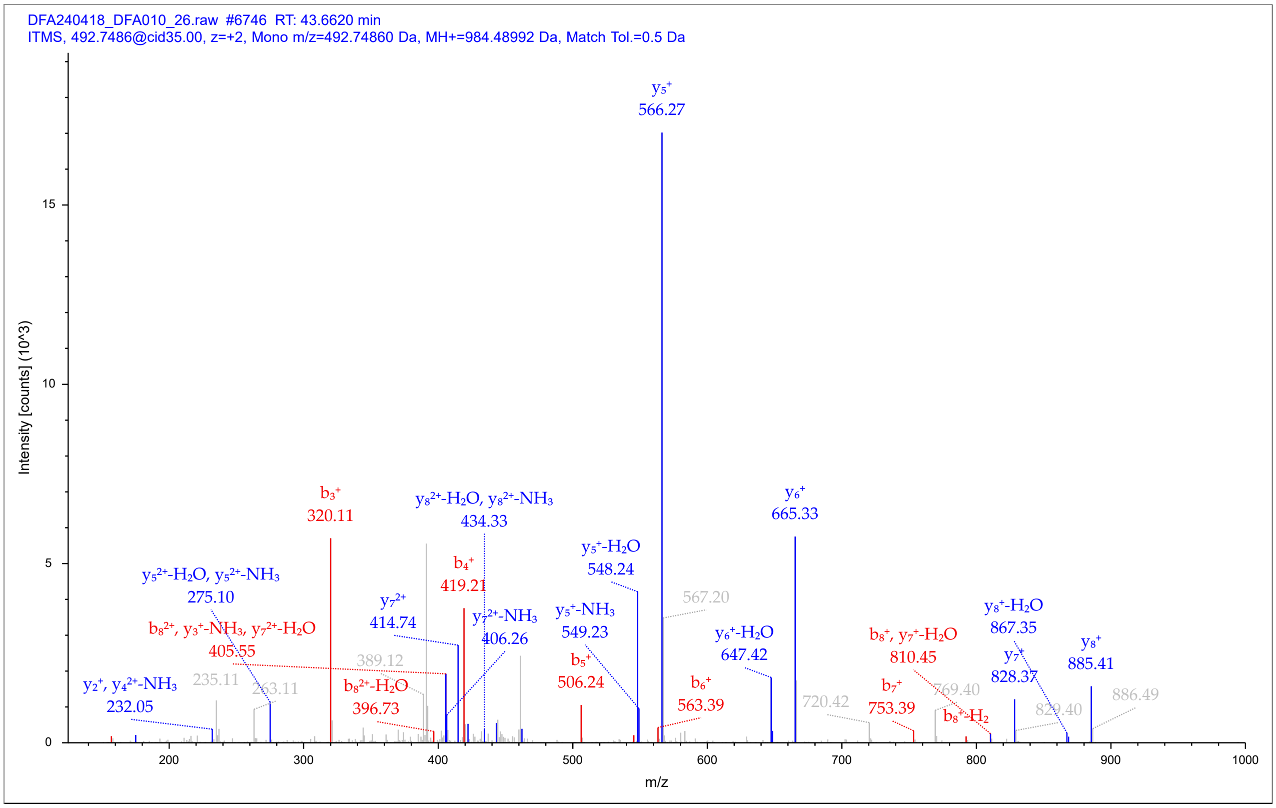

#1	b⁺	b²⁺	Seq.	y⁺	y²⁺	#2
1	100.07569	50.54148	V			9
2	157.09715	79.05222	G	885.42134	443.21431	8
3	320.16048	160.58388	Y	828.39988	414.70358	7
4	419.22890	210.11809	V	665.33655	333.17191	6
5	506.26092	253.63410	S	566.26814	283.63771	5
6	563.28239	282.14483	G	479.23611	240.12169	4
7	753.35662	377.18195	W-Trp->Kynurenin	422.21464	211.61096	3
8	810.37808	405.69268	G	232.14042	116.57385	2
9			R	175.11895	88.06311	1

Sequence: KAVSKLYASK, K10-Carboxyethyl (72.02113 Da), K1-Glycerinyl (88.01674 Da)
Charge: +3, Monoisotopic m/z: 418.90298 Da (-0.1 mmu/-0.23 ppm), MH+: 1254.69440 Da, RT: 49.8492 min,
Identified with: Sequest HT (v1.17); XCorr:0.90, Percolator q-Value:4.1e-3, Percolator PEP:2.0e-2, ptmRS: Best Site Probabilities:K10(Carboxyethyl): 100,
Fragment match tolerance used for search: 0.02 Da
Fragments used for search: -H_2O; y; -NH_3; y; b; b; -H_2O; b; -NH_3; y

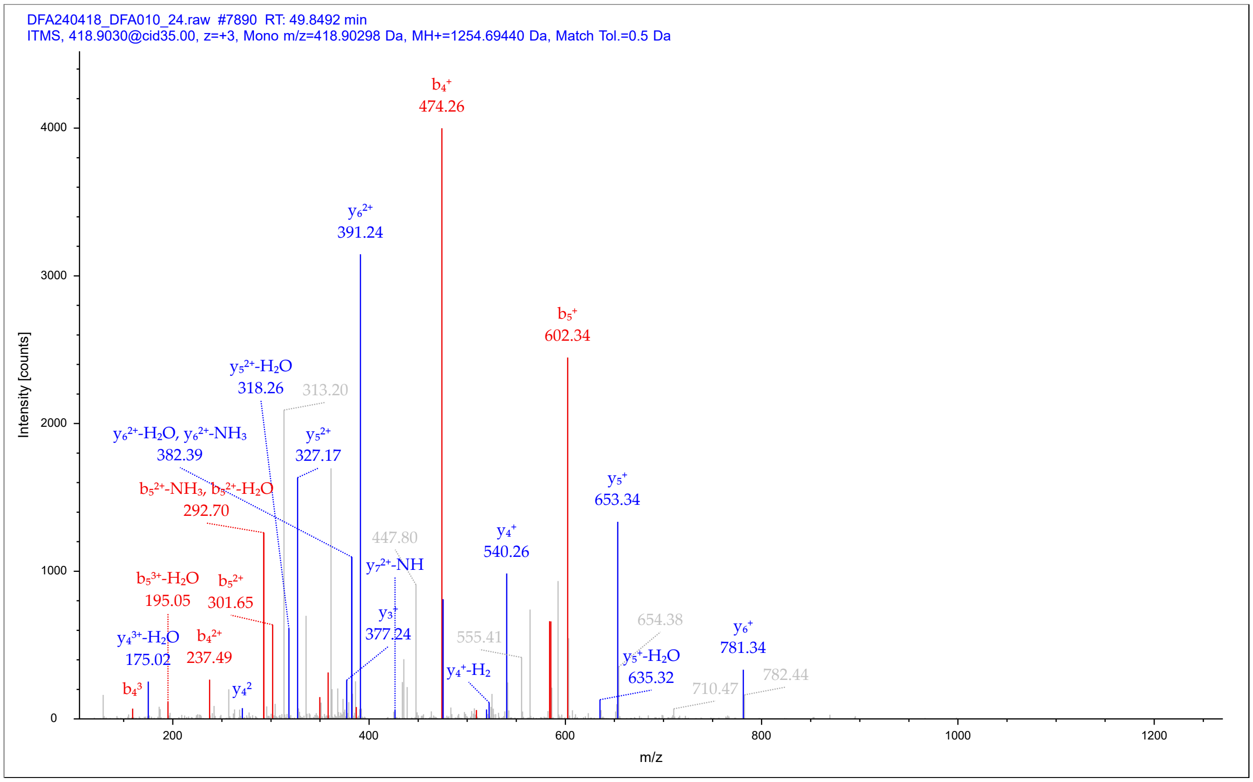

#1	b+	b2+	b3+	Seq.	y+	y2+	y3+	#2
1	217.11898	109.06313	73.04451	K-Glycerinyl				10
2	288.15609	144.58168	96.72355	A	1038.58299	519.79513	346.86585	9
3	387.22451	194.11589	129.74635	V	967.54587	484.27657	323.18681	8
4	474.25654	237.63191	158.75703	S	868.47746	434.74237	290.16400	7
5	602.35150	301.67939	201.45535	K	781.44543	391.22635	261.15333	6
6	715.43556	358.22142	239.15004	L	653.35047	327.17887	218.45501	5
7	878.49889	439.75308	293.50448	Y	540.26640	270.63684	180.76032	4
8	949.53600	475.27164	317.18352	A	377.20308	189.10518	126.40588	3
9	1036.56803	518.78765	346.19420	S	306.16596	153.58662	102.72684	2
10				K-Carboxy ethyl	219.13393	110.07060	73.71616	1

Sequence: VRWGWTRR, W3-Trp->Kynurenin (3.99492 Da), R7-Carboxyethyl (72.02113 Da), R8-Carboxyethyl (72.02113 Da)
Charge: +2, Monoisotopic m/z: 632.82953 Da (-1.38 mmu/-2.17 ppm), MH+: 1264.65178 Da, RT: 59.9587 min,
Identified with: Sequest HT (v1.17); XCorr:0.82, Percolator q-Value:6.8e-3, Percolator PEP:3.5e-2,
Fragment match tolerance used for search: 0.02 Da
Fragments used for search: -H_2O; y; -NH_3; y; b; b; -H_2O; b; -NH_3; y

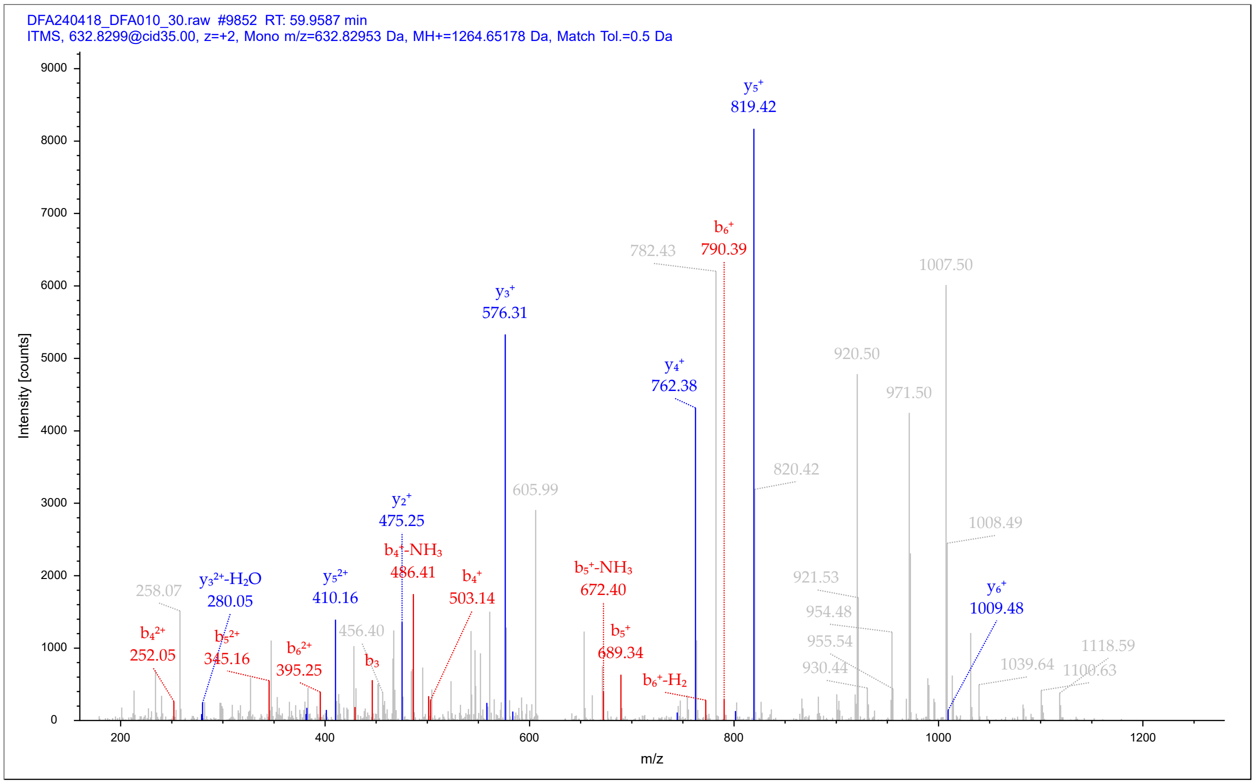

#1	b⁺	b²⁺	Seq.	y⁺	y²⁺	#2
1	100.07569	50.54148	V			8
2	256.17680	128.59204	R	1165.58612	583.29670	7
3	446.25103	223.62915	W-Trp-> Kynurenin	1009.48500	505.24614	6
4	503.27249	252.13988	G	819.41078	410.20903	5
5	689.35181	345.17954	W	762.38931	381.69829	4
6	790.39948	395.70338	T	576.31000	288.65864	3
7	1018.52172	509.76450	R-Carboxy ethyl	475.26232	238.13480	2
8			R-Carboxy ethyl	247.14008	124.07368	1

Sequence: MAPLCSLAR, C5-Carbamidomethyl (57.02146 Da), M1-Oxidation (15.99492 Da), R9-Delta:H(2)C(3)O(1) (54.01057 Da)
Charge: +2, Monoisotopic m/z: 544.76337 Da (-1.63 mmu/-2.99 ppm), MH+: 1088.51946 Da, RT: 71.9184 min,
Identified with: Sequest HT (v1.17); XCorr:0.73, Percolator q-Value:4.9e-3, Percolator PEP:3.2e-2,
Fragment match tolerance used for search: 0.02 Da
Fragments used for search: -H_2O; y; b; b; -H_2O; y

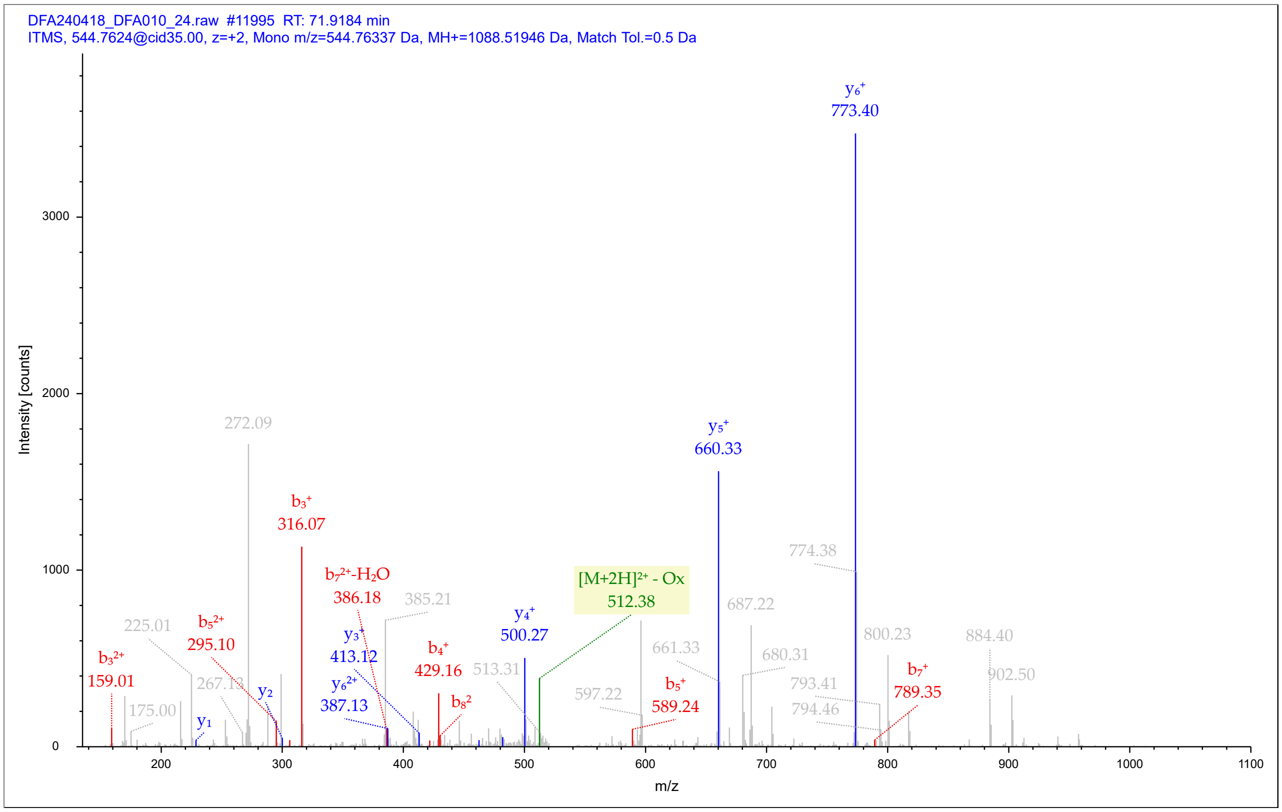

#1	b+	b2+	Seq.	y+	y2+	#2
1	148.04268	74.52498	M-Oxidation			9
2	219.07979	110.04353	A	941.48731	471.24729	8
3	316.13255	158.56992	P	870.45020	435.72874	7
4	429.21662	215.11195	L	773.39744	387.20236	6
5	589.24727	295.12727	C-Carbamido methyl	660.31337	330.66032	5
6	676.27929	338.64329	S	500.28272	250.64500	4
7	789.36336	395.18532	L	413.25069	207.12899	3
8	860.40047	430.70387	A	300.16663	150.58695	2
9			MGH	229.12952	115.06840	1

Sequence: MDQSRVLLWVK, W9-Trp->Oxolactone (13.97927 Da), K11-Glycerinyl (88.01674 Da)
Charge: +2, Monoisotopic m/z: 738.87952 Da (-0.23 mmu/-0.32 ppm), MH+: 1476.75176 Da, RT: 72.2637 min,
Identified with: Sequest HT (v1.17); XCorr:0.80, Percolator q-Value:3.2e-3, Percolator PEP:1.1e-2,
Fragment match tolerance used for search: 0.02 Da
Fragments used for search: -H_2O; y; -NH_3; y; b; b; -H_2O; b; -NH_3; y

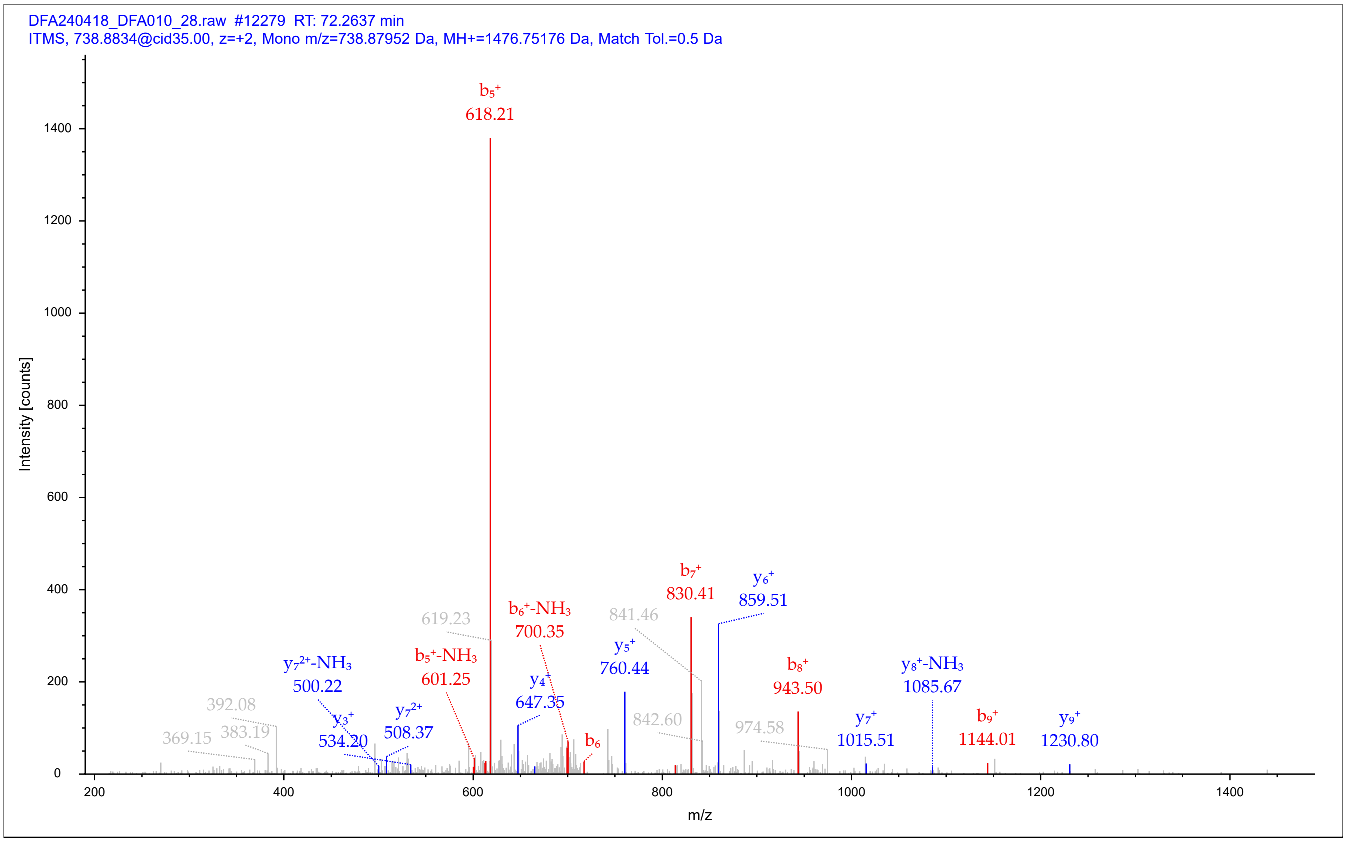

#1	b^+	b^{2+}	Seq.	y^+	y^{2+}	#2
1	132.04776	66.52752	M			11
2	247.07470	124.04099	D	1345.71174	673.35951	10
3	375.13328	188.07028	Q	1230.68479	615.84604	9
4	462.16531	231.58629	S	1102.62622	551.81675	8
5	618.26642	309.63685	R	1015.59419	508.30073	7
6	717.33483	359.17106	V	859.49308	430.25018	6
7	830.41890	415.71309	L	760.42466	380.71597	5
8	943.50296	472.25512	L	647.34060	324.17394	4
9	1143.56154	572.28441	W-Trp->Oxolactone	534.25654	267.63191	3
10	1242.62995	621.81862	V	334.19796	167.60262	2
11			K-Glycerinyl	235.12954	118.06841	1

Sequence: NQGNTWLTAFVLK, W6-Trp->Kynurenin (3.99492 Da)
Charge: +2, Monoisotopic m/z: 748.39832 Da (-0.5 mmu/-0.67 ppm), MH+: 1495.78935 Da, RT: 90.2615 min,
Identified with: Sequest HT (v1.17); XCorr:0.75, Percolator q-Value:5.3e-3, Percolator PEP:1.7e-2,
Fragment match tolerance used for search: 0.02 Da
Fragments used for search: -H_2O; y; -NH_3; y; b; b; -H_2O; b; -NH_3; y

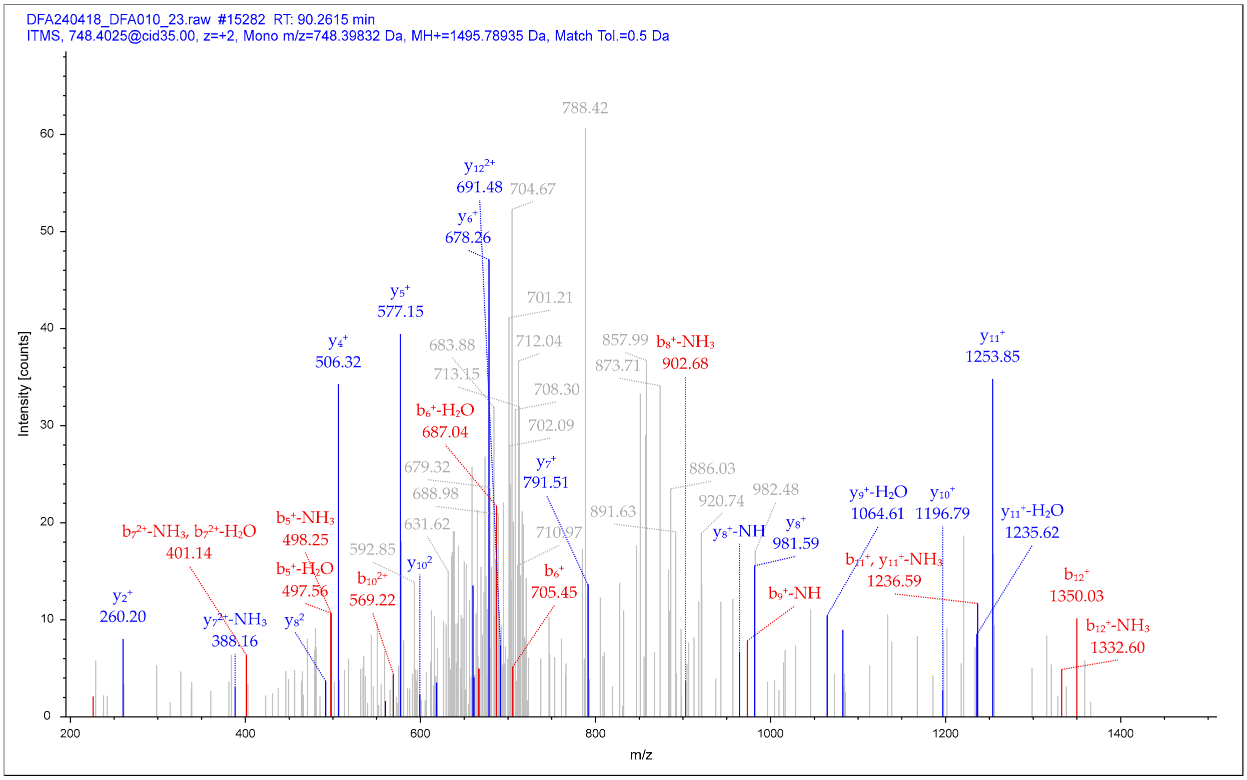

#1	b+	b2+	Seq.	y+	y2+	#2
1	115.05020	58.02874	N			13
2	243.10878	122.05803	Q	1381.74743	691.37735	12
3	300.13025	150.56876	G	1253.68885	627.34806	11
4	414.17317	207.59022	N	1196.66739	598.83733	10
5	515.22085	258.11406	T	1082.62446	541.81587	9
6	705.29508	353.15118	W-Trp->Kynurenin	981.57678	491.29203	8
7	818.37914	409.69321	L	791.50255	396.25491	7
8	919.42682	460.21705	T	678.41849	339.71288	6
9	990.46393	495.73561	A	577.37081	289.18904	5
10	1137.53235	569.26981	F	506.33370	253.67049	4
11	1236.60076	618.80402	V	359.26528	180.13628	3
12	1349.68483	675.34605	L	260.19687	130.60207	2
13			K	147.11280	74.06004	1

Sequence: FSPGLPGYPR, R10-Fructosyl (162.05354 Da)
Charge: +2, Monoisotopic m/z: 626.81372 Da (-0.69 mmu/-1.1 ppm), MH+: 1252.62016 Da, RT: 57.2251 min,
Identified with: Sequest HT (v1.17); XCorr:0.55, Percolator q-Value:3.3e-3, Percolator PEP:2.0e-2,
Fragment match tolerance used for search: 0.02 Da
Fragments used for search: -H_2O; y; b; b; -H_2O; y

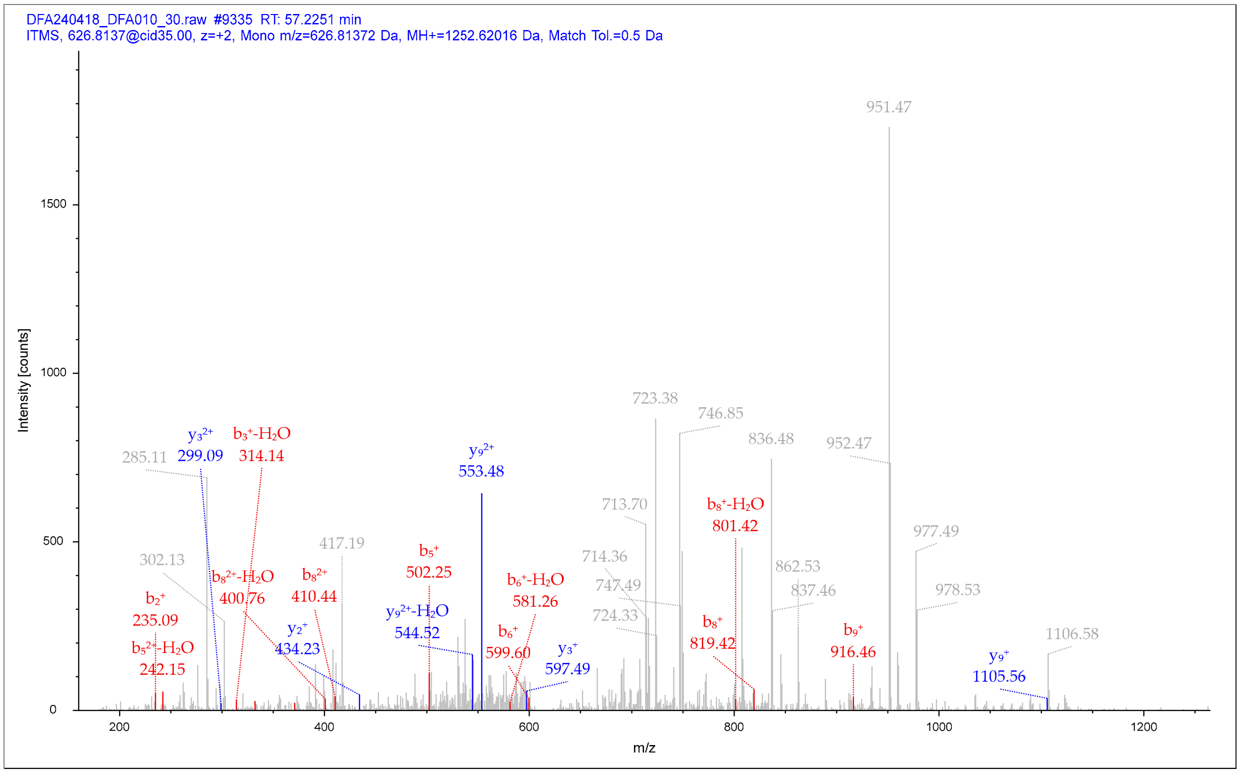

#1	b+	b2+	Seq.	y+	y2+	#2
1	148.07569	74.54148	F			10
2	235.10772	118.05750	S	1105.55313	553.28020	9
3	332.16048	166.58388	P	1018.52110	509.76419	8
4	389.18195	195.09461	G	921.46834	461.23781	7
5	502.26601	251.63664	L	864.44688	432.72708	6
6	599.31877	300.16303	P	751.36281	376.18504	5
7	656.34024	328.67376	G	654.31005	327.65866	4
8	819.40357	410.20542	Y	597.28858	299.14793	3
9	916.45633	458.73180	P	434.22526	217.61627	2
10			R-Fructosyl	337.17249	169.08988	1

2_166 (CEX)

Sequence: AAFALGGLGSGFASNR, R16-Carboxyethyl (72.02113 Da)
Charge: +2, Monoisotopic m/z: 784.39642 Da (-0.38 mmu/-0.49 ppm), MH+: 1567.78557 Da, RT: 66.4761 min,
Identified with: Sequest HT (v1.17); XCorr:0.52, Percolator q-Value:2.3e-3, Percolator PEP:3.7e-3,
Fragment match tolerance used for search: 0.02 Da
Fragments used for search: $-H_2O$; y; $-NH_3$; y; b; b; $-H_2O$; b; $-NH_3$; y

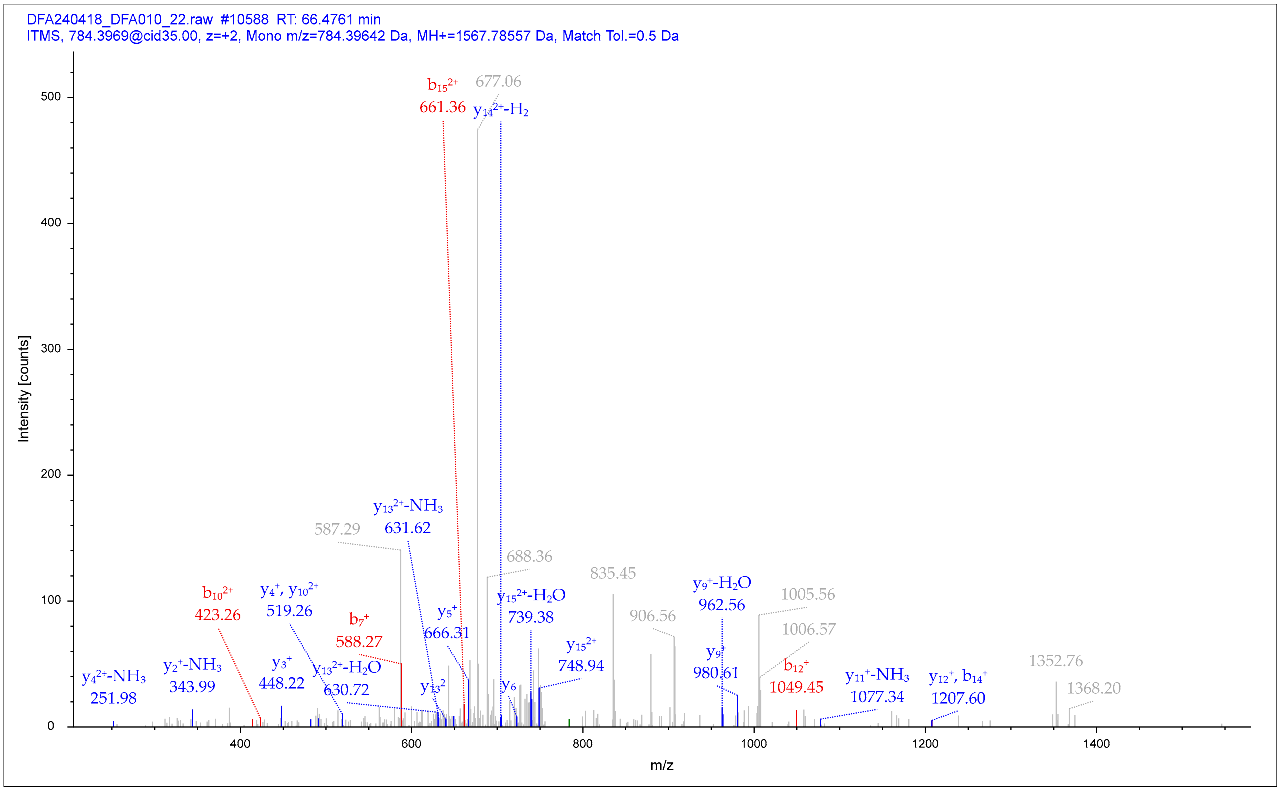

#1	b⁺	b²⁺	Seq.	y⁺	y²⁺	#2
1	72.04439	36.52583	A			16
2	143.08150	72.04439	A	1496.74922	748.87825	15
3	290.14992	145.57860	F	1425.71210	713.35969	14
4	361.18703	181.09715	A	1278.64369	639.82548	13
5	474.27110	237.63919	L	1207.60658	604.30693	12
6	531.29256	266.14992	G	1094.52251	547.76489	11
7	588.31402	294.66065	G	1037.50105	519.25416	10
8	701.39809	351.20268	L	980.47958	490.74343	9
9	758.41955	379.71341	G	867.39552	434.20140	8
10	845.45158	423.22943	S	810.37406	405.69067	7
11	902.47304	451.74016	G	723.34203	362.17465	6
12	1049.54146	525.27437	F	666.32056	333.66392	5
13	1120.57857	560.79292	A	519.25215	260.12971	4
14	1207.61060	604.30894	S	448.21504	224.61116	3
15	1321.65353	661.33040	N	361.18301	181.09514	2
16			R-Carboxy ethyl	247.14008	124.07368	1

2_166

equence: AAFALGGLGSGFASNR, R16-Triosyl (72.01840 Da)
Charge: +2, Monoisotopic m/z: 784.39642 Da (+0.98 mmu/+1.26 ppm), MH+: 1567.78557 Da, RT: 66.4761 min,
Identified with: Sequest HT (v1.17); XCorr:0.45, Percolator q-Value:8.4e-3, Percolator PEP:4.2e-2,
Fragment match tolerance used for search: 0.02 Da
Fragments used for search: -H_2O; y; -NH_3; y; b; b; -H_2O; b; -NH_3; y

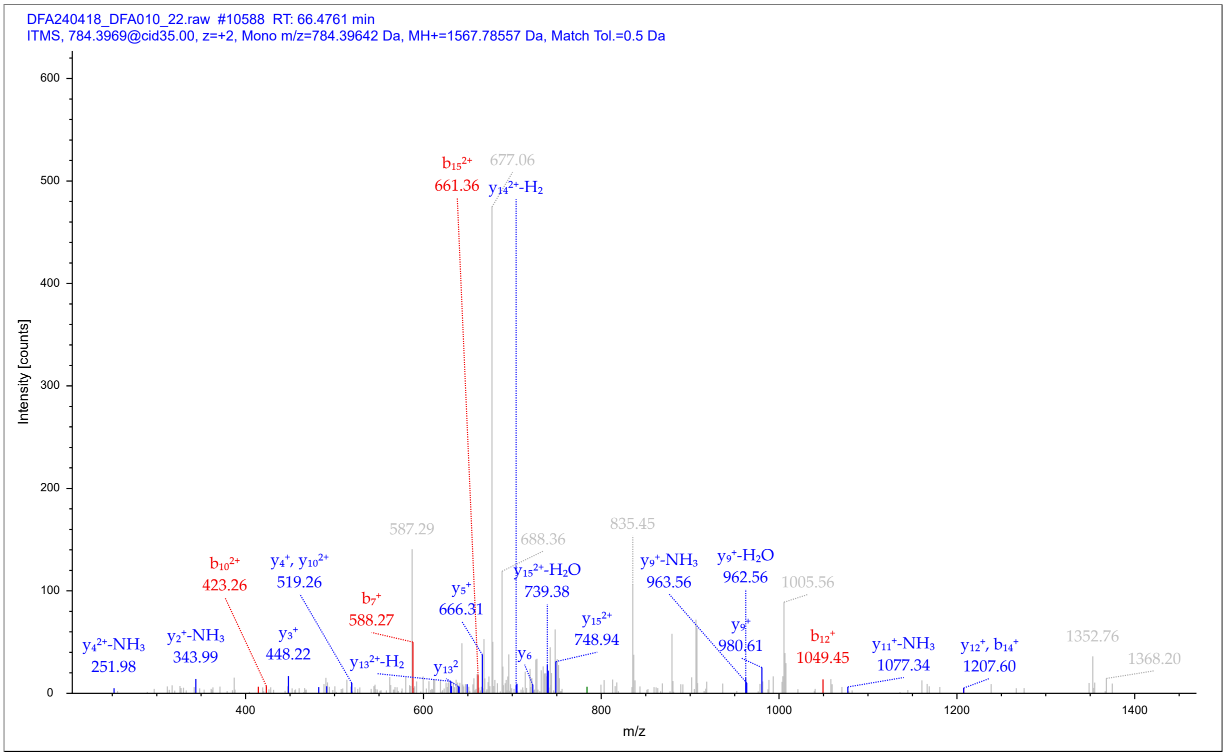

#1	b⁺	b²⁺	Seq.	y⁺	y²⁺	#2
1	72.04439	36.52583	A			16
2	143.08150	72.04439	A	1496.74649	748.87688	15
3	290.14992	145.57860	F	1425.70937	713.35833	14
4	361.18703	181.09715	A	1278.64096	639.82412	13
5	474.27110	237.63919	L	1207.60385	604.30556	12
6	531.29256	266.14992	G	1094.51978	547.76353	11
7	588.31402	294.66065	G	1037.49832	519.25280	10
8	701.39809	351.20268	L	980.47686	490.74207	9
9	758.41955	379.71341	G	867.39279	434.20003	8
10	845.45158	423.22943	S	810.37133	405.68930	7
11	902.47304	451.74016	G	723.33930	362.17329	6
12	1049.54146	525.27437	F	666.31784	333.66256	5
13	1120.57857	560.79292	A	519.24942	260.12835	4
14	1207.61060	604.30894	S	448.21231	224.60979	3
15	1321.65353	661.33040	N	361.18028	181.09378	2
16			R-Triosyl	247.13735	124.07231	1

137

Sequence: VDMTIAEACQKSK, K11-Acetyl (42.01057 Da), C9-Carbamidomethyl (57.02146 Da), K13-Carboxymethyl (58.00548 Da), M3-Trioxidation (47.98474 Da)
Charge: +4, Monoisotopic m/z: 407.93307 Da (-0.94 mmu/-2.3 ppm), MH+: 1628.71047 Da, RT: 19.5089 min, Identified with: Sequest HT (v1.17); XCorr:0.73, Percolator q-Value:6.8e-3, Percolator PEP:2.9e-2, ptmRS: Best Site Probabilities:K11(Acetyl): 100,
Fragment match tolerance used for search: 0.02 Da
Fragments used for search: -H_2O; y; -NH_3; y; b; b; -H_2O; b; -NH_3; y

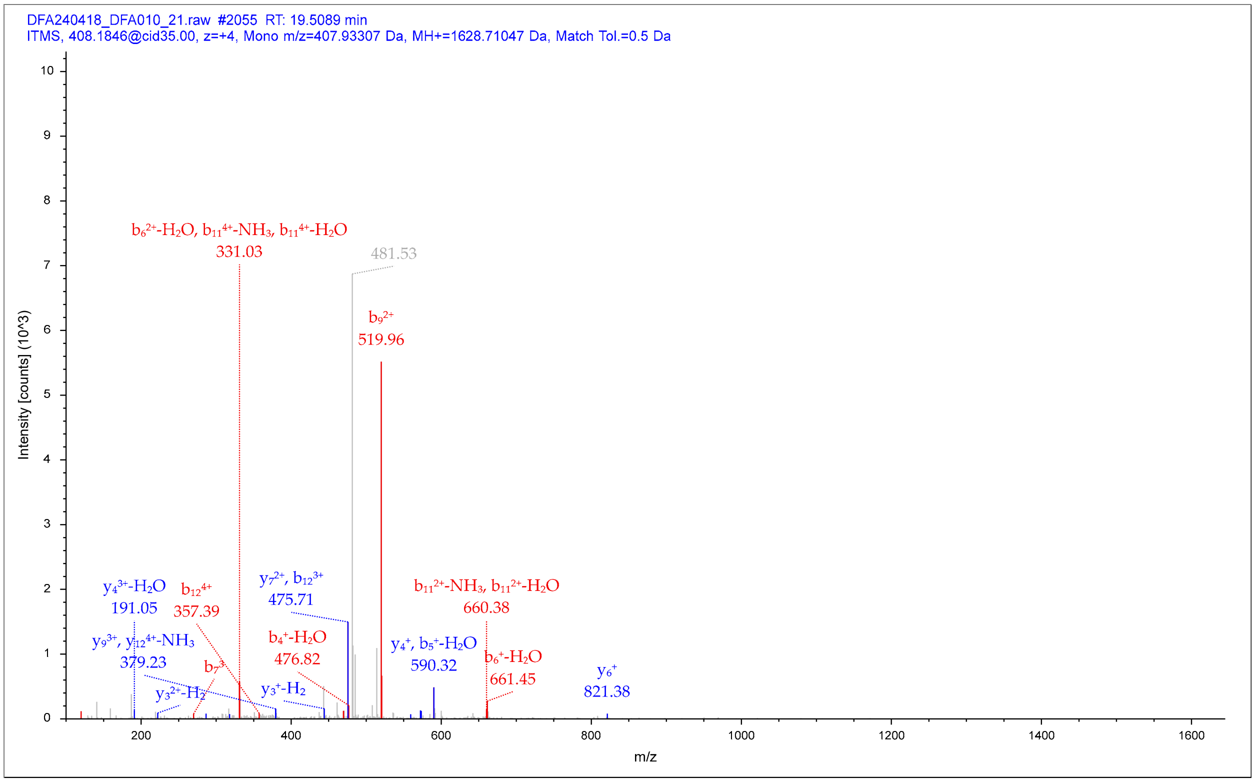

#1	b+	b2+	b3+	b4+	Seq.	y+	y2+	y3+	y4+	#2
1	100.07569	50.54148	34.03008	25.77438	V					13
2	215.10263	108.05496	72.37240	54.53112	D	1529.64580	765.32654	510.55345	383.16691	12
3	394.12786	197.56757	132.04747	99.28742	M-Trioxidation	1414.61886	707.81307	472.21114	354.41017	11
4	495.17554	248.09141	165.73003	124.54934	T	1235.59363	618.30045	412.53606	309.65386	10
5	608.25960	304.63344	203.42472	152.82036	I	1134.54595	567.77661	378.85350	284.39194	9
6	679.29672	340.15200	227.10376	170.57964	A	1021.46189	511.23458	341.15881	256.12093	8
7	808.33931	404.67329	270.11795	202.84029	E	950.42477	475.71602	317.47978	238.36165	7
8	879.37642	440.19185	293.79699	220.59956	A	821.38218	411.19473	274.46558	206.10100	6
9	1039.40707	520.20717	347.14054	260.60723	C-Carbamido-methyl	750.34507	375.67617	250.78654	188.34172	5
10	1167.46565	584.23646	389.82673	292.62187	Q	590.31442	295.66085	197.44299	148.33406	4
11	1337.57118	669.28923	446.52858	335.14825	K-Acetyl	462.25584	231.63156	154.75680	116.31942	3
12	1424.60321	712.80524	475.53925	356.90626	S	292.15031	146.57879	98.05495	73.79304	2
13					K-Carboxy-methyl	205.11828	103.06278	69.04428	52.03503	1

Sequence: QVQNLVFHSLTVTQMAFSPNEK, M15-Trioxidation (47.98474 Da), K22-Triosyl (72.01840 Da)
Charge: +6, Monoisotopic m/z: 440.55322 Da (+0.66 mmu/+1.49 ppm), MH+: 2638.28295 Da, RT: 33.6673 min,
Identified with: Sequest HT (v1.17); XCorr:1.09, Percolator q-Value:3.2e-3, Percolator PEP:1.3e-2,
Fragment match tolerance used for search: 0.02 Da
Fragments used for search: $-H_2O$; y; $-NH_3$; y; b; b; $-H_2O$; b; $-NH_3$; y

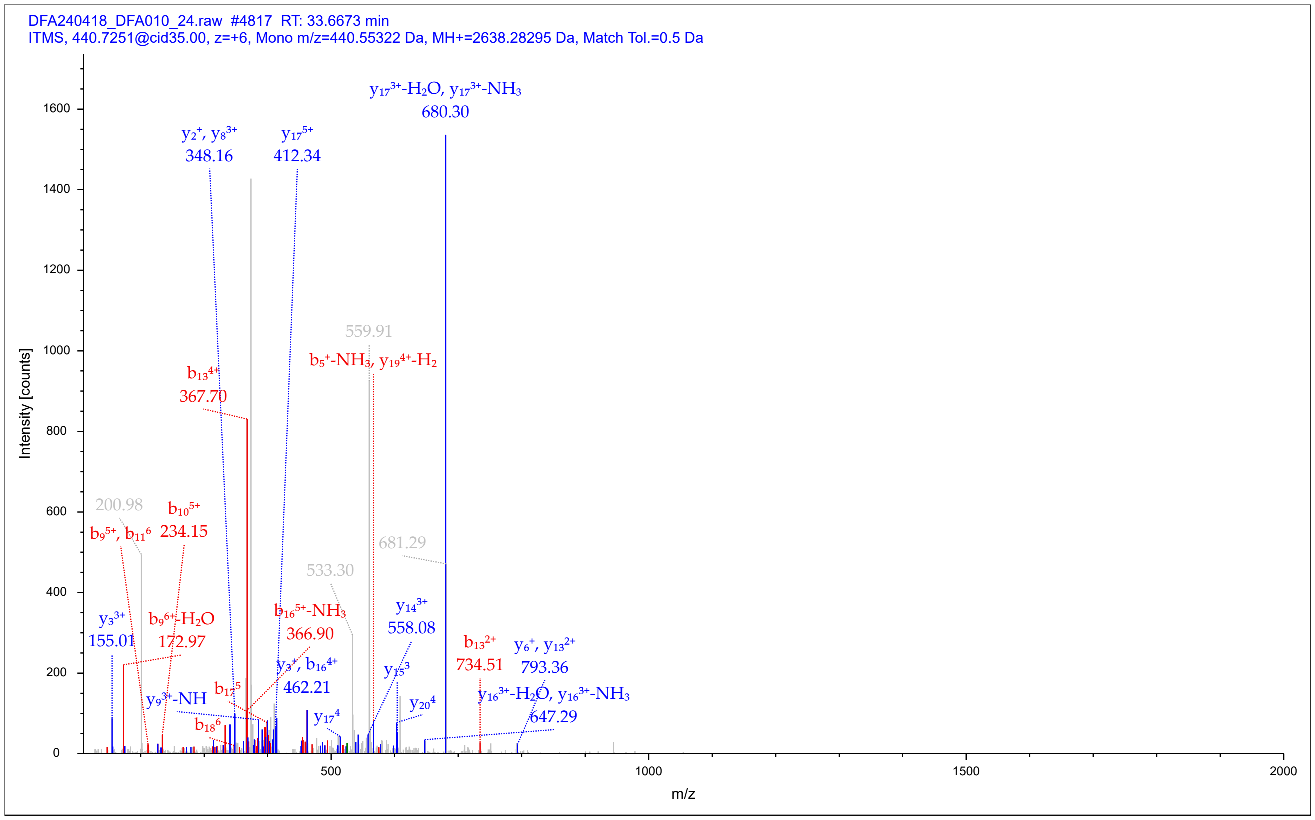

#1	b^+	b^{2+}	b^{3+}	b^{4+}	b^{5+}	b^{6+}	Seq.	y^+	y^{2+}	y^{3+}	y^{4+}	y^{5+}	y^{6+}	#2
1	129.06585	65.03657	43.69347	33.02192	26.61899	22.35037	Q							22
2	228.13427	114.57077	76.71627	57.78902	46.43267	38.86178	V	2510.22044	1255.61386	837.41166	628.31057	502.84991	419.20947	21
3	356.19285	178.60006	119.40247	89.80367	72.04439	60.20487	Q	2411.15202	1206.07965	804.38886	603.54346	483.03623	402.69807	20
4	470.23577	235.62152	157.41678	118.31440	94.85298	79.21203	N	2283.09344	1142.05036	761.70267	571.52882	457.42451	381.35497	19
5	583.31984	292.16356	195.11146	146.58542	117.46979	98.05937	L	2169.05052	1085.02890	723.68836	543.01809	434.61592	362.34782	18
6	682.38825	341.69776	228.13427	171.35252	137.28347	114.57077	V	2055.96645	1028.48686	685.99367	514.74707	411.99911	343.50047	17
7	829.45666	415.23197	277.15707	208.11962	166.69715	139.08217	F	1956.89804	978.95266	652.97086	489.97997	392.18543	326.98907	16
8	966.51558	483.76143	322.84338	242.38435	194.10894	161.92533	H	1809.82963	905.41845	603.94806	453.21286	362.77175	302.47767	15
9	1053.54760	527.27744	351.85405	264.14236	211.51534	176.43066	S	1672.77071	836.88900	558.26176	418.94814	335.35996	279.63452	14
10	1166.63167	583.81947	389.54874	292.41337	234.13216	195.27801	L	1585.73869	793.37298	529.25108	397.19013	317.95356	265.12918	13
11	1267.67935	634.34331	423.23130	317.67529	254.34169	212.11929	T	1472.65462	736.83095	491.55639	368.91911	295.33675	246.28183	12
12	1366.74776	683.87752	456.25410	342.44240	274.15537	228.63069	V	1371.60694	686.30711	457.87383	343.65719	275.12721	229.44055	11
13	1467.79544	734.40136	489.93666	367.70432	294.36491	245.47197	T	1272.53853	636.77290	424.85103	318.89009	255.31353	212.92915	10
14	1595.85402	798.43065	532.62286	399.71896	319.97662	266.81507	Q	1171.49085	586.24906	391.16847	293.62817	235.10399	196.08787	9
15	1774.87925	887.94326	592.29793	444.47527	355.78167	296.65260	M-Triox	1043.43227	522.21977	348.48228	261.61353	209.49228	174.74478	8
16	1845.91636	923.46182	615.97697	462.23455	369.98909	308.49212	A	864.40704	432.70716	288.80720	216.85722	173.68723	144.90724	7
17	1992.98477	996.99602	664.99978	499.00165	399.40278	333.00353	F	793.36993	397.18860	265.12816	199.09794	159.47981	133.06772	6
18	2080.01680	1040.51204	694.01045	520.75966	416.80918	347.50886	S	646.30152	323.65440	216.10536	162.33084	130.06612	108.55632	5
19	2177.06957	1089.03842	726.36137	545.02285	436.21973	363.68432	P	559.26949	280.13838	187.09468	140.57283	112.65972	94.05098	4
20	2291.11249	1146.05988	764.37568	573.53358	459.02832	382.69148	N	462.21672	231.61200	154.74376	116.30964	93.24917	77.87552	3
21	2420.15509	1210.58118	807.38988	605.79423	484.83684	404.19858	E	348.17380	174.59054	116.72945	87.79891	70.44058	58.86836	2
22							K-Triosyl	219.13120	110.06924	73.71525	55.53826	44.63206	37.36126	1

Sequence: MKCTLTSR, C3-Carbamidomethyl (57.02146 Da), K2-Glyoxal-imine (39.99554 Da)
Charge: +2, Monoisotopic m/z: 518.75232 Da (+2.66 mmu/+5.14 ppm), MH+: 1036.49736 Da, RT: 34.3299 min,
Identified with: Sequest HT (v1.17); XCorr:1.00, Percolator q-Value:0.0e0, Percolator PEP:2.7e-2,
Fragment match tolerance used for search: 0.02 Da
Fragments used for search: -H_2O; y; -NH_3; y; b; b; -H_2O; y

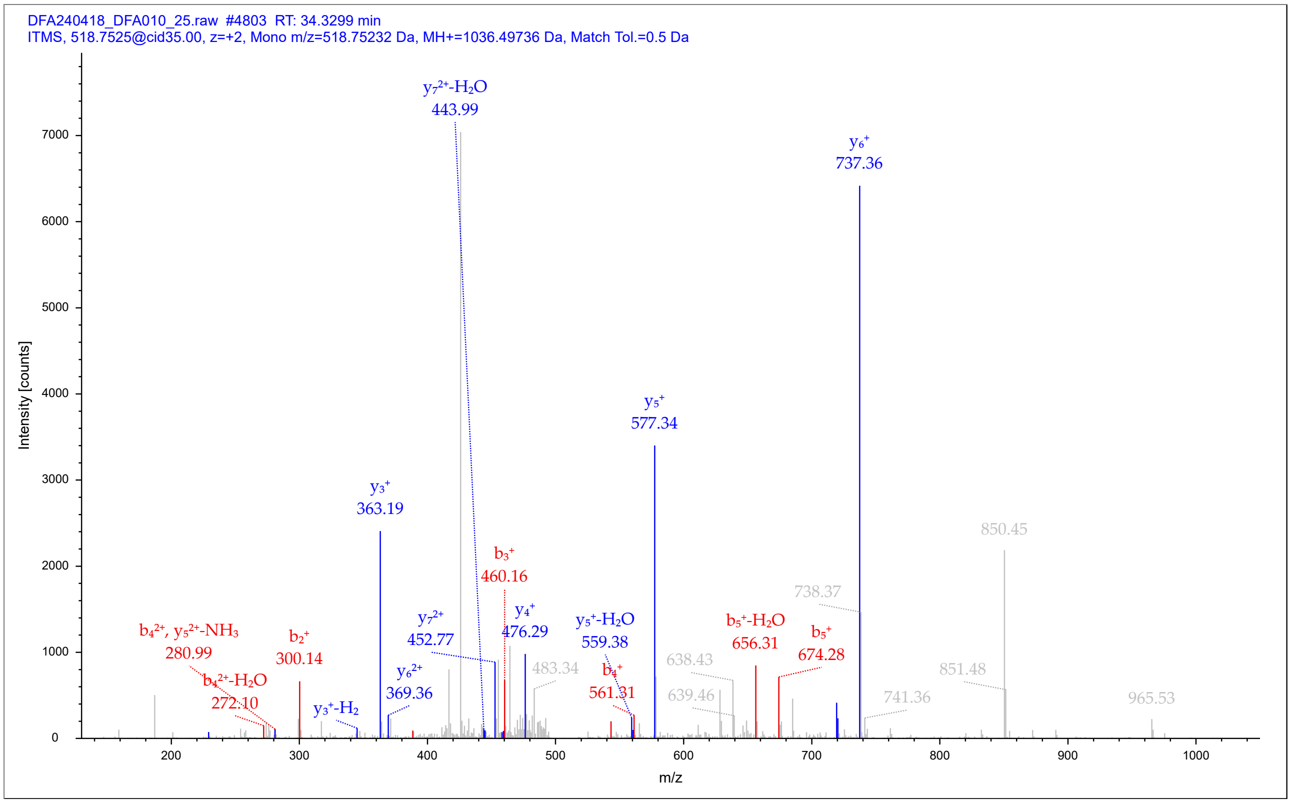

#1	b⁺	b²⁺	Seq.	y⁺	y²⁺	#2
1	132.04776	66.52752	M			8
2	300.13826	150.57277	K-Glyoxal-imine	905.45155	453.22941	7
3	460.16891	230.58809	C-Carbamidomethyl	737.36105	369.18416	6
4	561.21659	281.11193	T	577.33040	289.16884	5
5	674.30065	337.65397	L	476.28272	238.64500	4
6	775.34833	388.17780	T	363.19866	182.10297	3
7	862.38036	431.69382	S	262.15098	131.57913	2
8			R	175.11895	88.06311	1

6

Sequence: AIAINTFLPKNGFRPLDVSLYAQQR, R14-Carboxymethyl (58.00548 Da), R25-Carboxymethyl (58.00548 Da), F7-Dioxidation (31.98983 Da), F13-Dioxidation (31.98983 Da)
Charge: +5, Monoisotopic m/z: 603.31573 Da (+1.41 mmu/+2.34 ppm), MH+: 3012.54957 Da, RT: 40.0778 min, Identified with: Sequest HT (v1.17); XCorr:0.55, Percolator q-Value:7.5e-3, Percolator PEP:1.4e-2,
Fragment match tolerance used for search: 0.02 Da
Fragments used for search: -H_2O; y; -NH_3; y; b; b; -H_2O; b; -NH_3; y

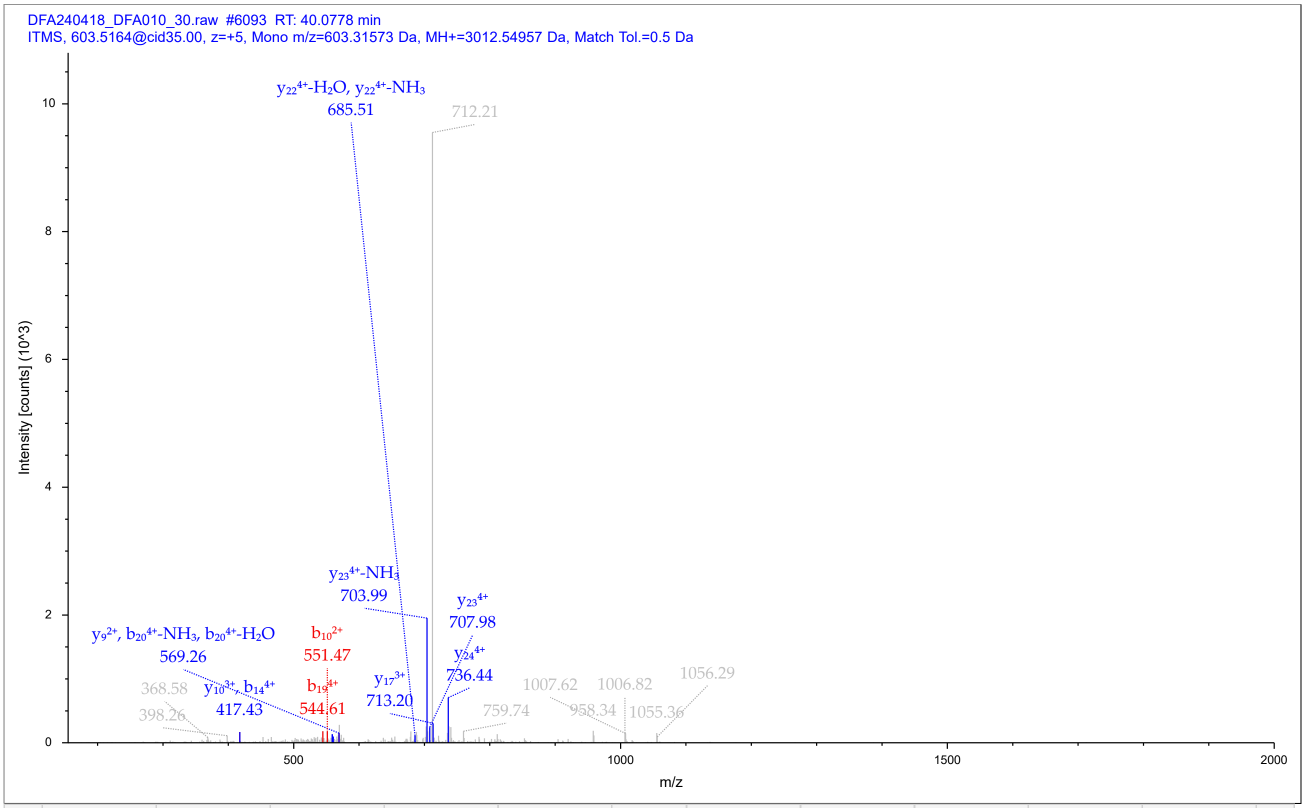

#1	b⁺	b²⁺	b³⁺	b⁴⁺	b⁵⁺	Seq.	y⁺	y²⁺	y³⁺	y⁴⁺	y⁵⁺	#2
1	72.04439	36.52583	24.68631	18.76656	15.21470	A						25
2	185.12845	93.06787	62.38100	47.03757	37.83151	I	2941.50541	1471.25634	981.17332	736.13181	589.10690	24
3	256.16557	128.58642	86.06004	64.79685	52.03893	A	2828.42135	1414.71431	943.47863	707.86079	566.49009	23
4	369.24963	185.12845	123.75473	93.06787	74.65575	I	2757.38423	1379.19575	919.79960	690.10152	552.28267	22
5	483.29256	242.14992	161.76904	121.57860	97.46433	N	2644.30017	1322.65372	882.10491	661.83050	529.66585	21
6	584.34024	292.67376	195.45160	146.84052	117.67387	T	2530.25724	1265.63226	844.09060	633.31977	506.85727	20
7	763.39848	382.20288	255.13768	191.60508	153.48552	F-Diox	2429.20956	1215.10842	810.40804	608.05785	486.64773	19
8	876.48254	438.74491	292.83237	219.87609	176.10233	L	2250.15132	1125.57930	750.72196	563.29329	450.83609	18
9	973.53531	487.27129	325.18329	244.13928	195.51288	P	2137.06726	1069.03727	713.02727	535.02227	428.21927	17
10	1101.63027	551.31877	367.88161	276.16303	221.13188	K	2040.01449	1020.51088	680.67635	510.75908	408.80872	16
11	1215.67320	608.34024	405.89592	304.67376	243.94046	N	1911.91953	956.46340	637.97803	478.73534	383.18973	15
12	1272.69466	636.85097	424.90307	318.92912	255.34475	G	1797.87660	899.44194	599.96372	450.22461	360.38114	14
13	1451.75291	726.38009	484.58915	363.69368	291.15640	F-Diox	1740.85514	870.93121	580.95656	435.96924	348.97685	13
14	1665.85950	833.43339	555.95802	417.22033	333.97772	R-Carboxy-methyl	1561.79690	781.40209	521.27048	391.20468	313.16520	12
15	1762.91226	881.95977	588.30894	441.48352	353.38827	P	1347.69031	674.34879	449.90162	337.67803	270.34388	11
16	1875.99632	938.50180	626.00363	469.75454	376.00509	L	1250.63754	625.82241	417.55070	313.41484	250.93333	10
17	1991.02327	996.01527	664.34594	498.51127	399.01047	D	1137.55348	569.28038	379.85601	285.14383	228.31652	9
18	2090.09168	1045.54948	697.36874	523.27838	418.82416	V	1022.52653	511.76691	341.51370	256.38709	205.31113	8
19	2177.12371	1089.06549	726.37942	545.03638	436.23056	S	923.45812	462.23270	308.49089	231.61999	185.49745	7
20	2290.20777	1145.60752	764.07411	573.30740	458.84738	L	836.42609	418.71668	279.48022	209.86198	168.09104	6
21	2453.27110	1227.13919	818.42855	614.07323	491.46004	Y	723.34203	362.17465	241.78553	181.59096	145.47423	5
22	2524.30821	1262.65775	842.10759	631.83251	505.66746	A	560.27870	280.64299	187.43108	140.82513	112.86156	4
23	2652.36679	1326.68703	884.79378	663.84716	531.27918	Q	489.24159	245.12443	163.75205	123.06585	98.65414	3
24	2780.42537	1390.71632	927.47997	695.86180	556.89090	Q	361.18301	181.09514	121.06585	91.05121	73.04242	2
25						R-Carboxy-methyl	233.12443	117.06585	78.37966	59.03657	47.43071	1

Sequence: MLQEICTLKK, C6-Carbamidomethyl (57.02146 Da), M1-Dioxidation (31.98983 Da), K9-GLAP (109.02805 Da), K10-GLAP (109.02805 Da)
Charge: +2, Monoisotopic m/z: 757.36700 Da (+0.43 mmu/+0.57 ppm), MH+: 1513.72673 Da, RT: 43.8780 min, Identified with: Sequest HT (v1.17); XCorr:0.92, Percolator q-Value:3.0e-3, Percolator PEP:2.2e-2, ptmRS: Best Site Probabilities:M1(Dioxidation): 100,
Fragment match tolerance used for search: 0.02 Da
Fragments used for search: -H_2O; y; -NH_3; y; b; b; -H_2O; b; -NH_3; y

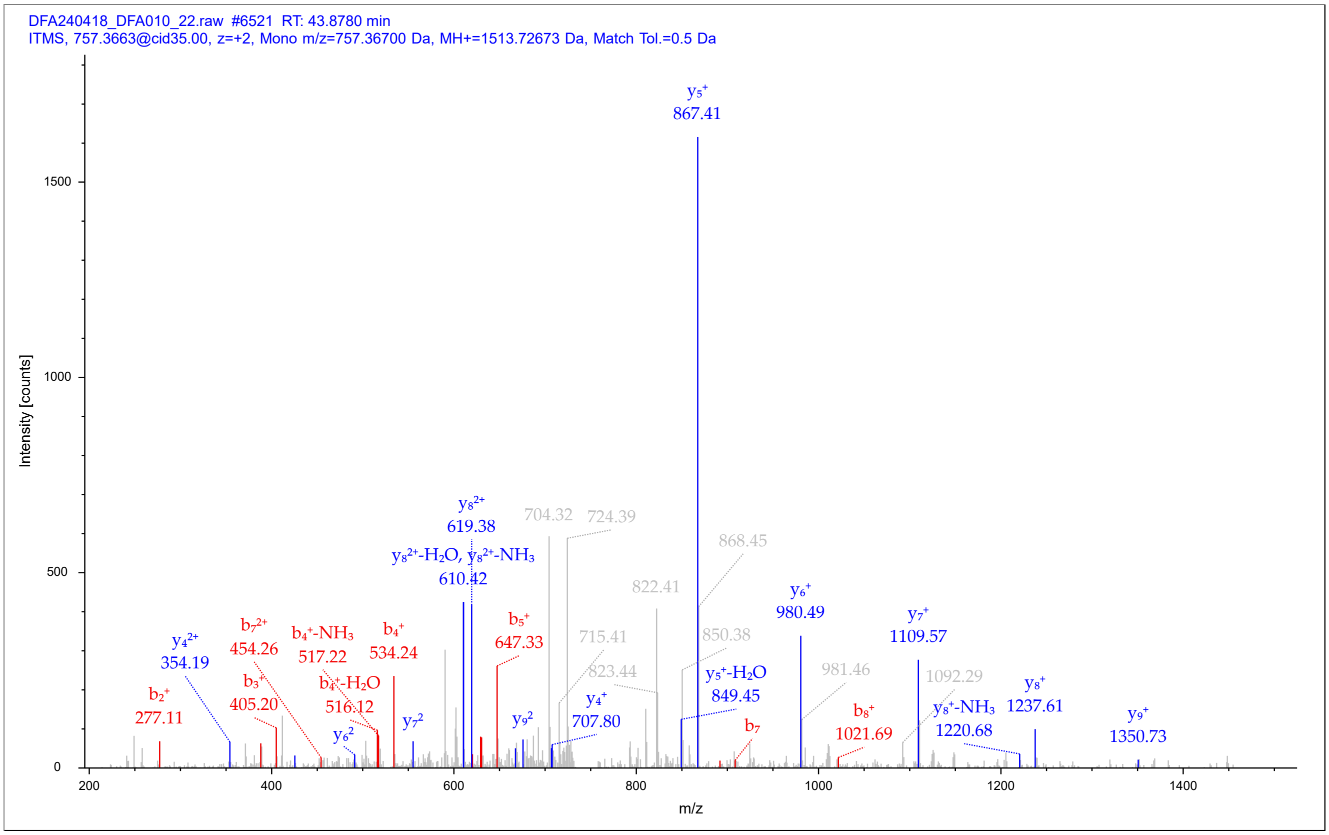

#1	b⁺	b²⁺	Seq.	y⁺	y²⁺	#2
1	164.03759	82.52243	M-Dioxidation			10
2	277.12165	139.06447	L	1350.69556	675.85142	9
3	405.18023	203.09375	Q	1237.61149	619.30938	8
4	534.22282	267.61505	E	1109.55291	555.28010	7
5	647.30689	324.15708	I	980.51032	490.75880	6
6	807.33754	404.17241	C-Carbamido-methyl	867.42626	434.21677	5
7	908.38522	454.69625	T	707.39561	354.20144	4
8	1021.46928	511.23828	L	606.34793	303.67760	3
9	1258.59229	629.79978	K-GLAP	493.26387	247.13557	2
10			K-GLAP	256.14085	128.57407	1

Sequence: TQNLEQKLSGDSRACR, C15-Carbamidomethyl (57.02146 Da), R16-Delta:H(2)C(3)O(1) (54.01057 Da), K7-Triosyl (72.01840 Da), R13-Triosyl (72.01840 Da)
Charge: +4, Monoisotopic m/z: 515.99487 Da (-0.86 mmu/-1.67 ppm), MH+: 2060.95766 Da, RT: 44.0986 min,
Identified with: Sequest HT (v1.17); XCorr:0.73, Percolator q-Value:6.6e-3, Percolator PEP:3.9e-2,
Fragment match tolerance used for search: 0.02 Da
Fragments used for search: -H_2O; y; -NH_3; y; b; b; -H_2O; b; -NH_3; y

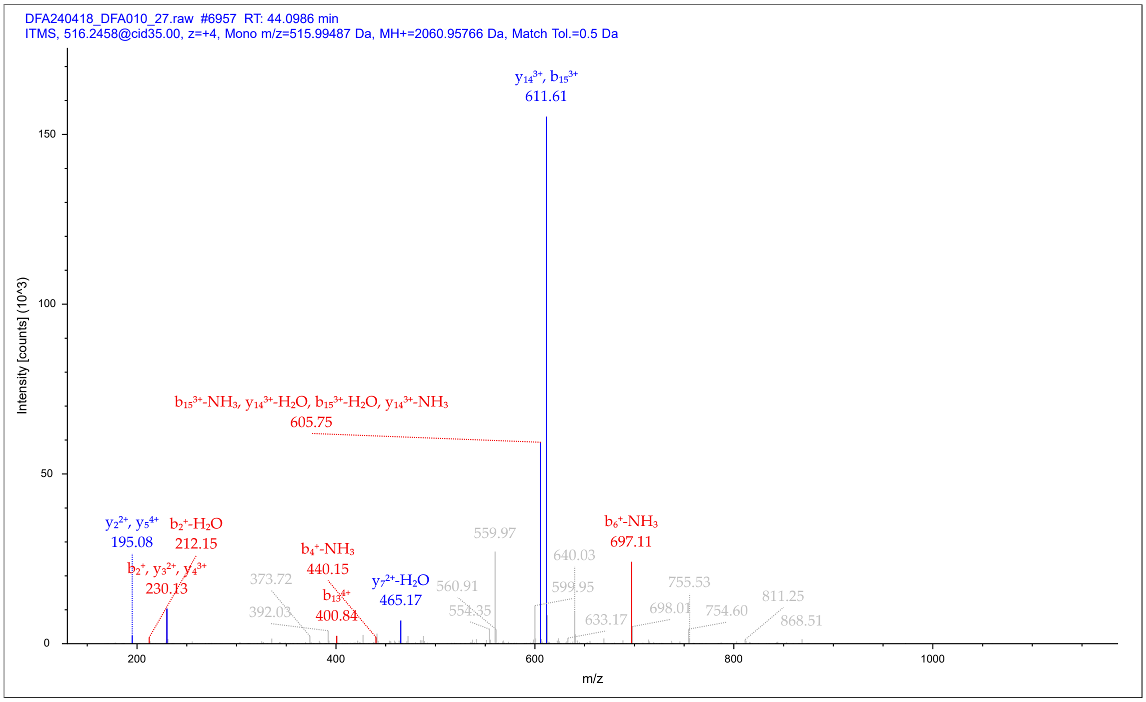

#1	b⁺	b²⁺	b³⁺	b⁴⁺	Seq.	y⁺	y²⁺	y³⁺	y⁴⁺	#2
1	102.05496	51.53112	34.68984	26.26920	T					16
2	230.11353	115.56040	77.37603	58.28384	Q	1959.91342	980.46035	653.97599	490.73381	15
3	344.15646	172.58187	115.39034	86.79457	N	1831.85484	916.43106	611.28980	458.71917	14
4	457.24052	229.12390	153.08503	115.06559	L	1717.81192	859.40960	573.27549	430.20844	13
5	586.28312	293.64520	196.09922	147.32624	E	1604.72785	802.86756	535.58080	401.93742	12
6	714.34169	357.67449	238.78542	179.34088	Q	1475.68526	738.34627	492.56660	369.67677	11
7	914.45506	457.73117	305.48987	229.36922	K-Triosyl	1347.62668	674.31698	449.88041	337.66213	10
8	1027.53912	514.27320	343.18456	257.64024	L	1147.51332	574.26030	383.17596	287.63379	9
9	1114.57115	557.78921	372.19523	279.39824	S	1034.42925	517.71827	345.48127	259.36277	8
10	1171.59261	586.29994	391.20239	293.65361	G	947.39723	474.20225	316.47059	237.60476	7
11	1286.61956	643.81342	429.54470	322.41035	D	890.37576	445.69152	297.46344	223.34940	6
12	1373.65158	687.32943	458.55538	344.16835	S	775.34882	388.17805	259.12112	194.59266	5
13	1601.77110	801.38919	534.59522	401.19823	R-Triosyl	688.31679	344.66203	230.11045	172.83466	4
14	1672.80821	836.90774	558.27425	418.95751	A	460.19728	230.60228	154.07061	115.80478	3
15	1832.83886	916.92307	611.61780	458.96517	C-Carbamido methyl	389.16017	195.08372	130.39157	98.04550	2
16					MGH	229.12952	115.06840	77.04802	58.03784	1

150
Sequence: WRNSEIQCYVNGQLVSYGDMAWHVNTNDSYDK, C8-Carbamidomethyl (57.02146 Da), W22-Trp->Kynurenin (3.99492 Da), K32-Formyl (27.99492 Da), R2-Glarg (39.99949 Da)
Charge: +6, Monoisotopic m/z: 654.45374 Da (-0.89 mmu/-1.36 ppm), MH+: 3921.68603 Da, RT: 45.0960 min,
Identified with: Sequest HT (v1.17); XCorr:0.82, Percolator q-Value:0.0e0, Percolator PEP:2.1e-3,
Fragment match tolerance used for search: 0.02 Da
Fragments used for search: $-H_2O$; y; $-NH_3$; y; b; b; $-H_2O$; b; $-NH_3$; y

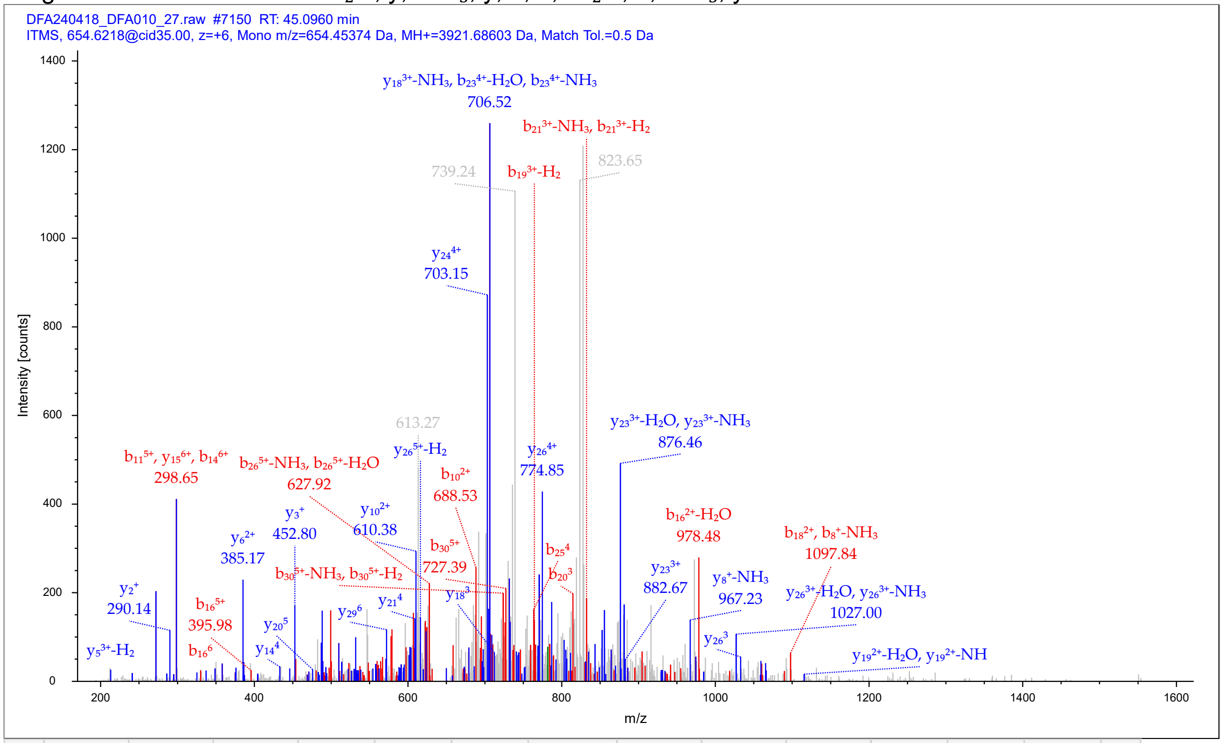

#1	b+	b2+	b3+	b4+	b5+	b6+	Seq.	y+	y2+	y3+	y4+	y5+	y6+	#2
1	187.08659	94.04693	63.03371	47.52710	38.22314	32.02050	W							32
2	383.18719	192.09723	128.40058	96.55226	77.44326	64.70393	R-Glarg	3735.61204	1868.30966	1245.87553	934.65847	747.92823	623.44140	31
3	497.23012	249.11870	166.41489	125.06299	100.25185	83.71108	N	3539.51144	1770.25936	1180.50866	885.63332	708.70811	590.75797	30
4	584.26215	292.63471	195.42557	146.82099	117.65825	98.21642	S	3425.46851	1713.23789	1142.49435	857.12259	685.89952	571.75082	29
5	713.30474	357.15601	238.43976	179.08164	143.46677	119.72352	E	3338.43648	1669.72188	1113.48368	835.36458	668.49312	557.24548	28
6	826.38881	413.69804	276.13445	207.35266	166.08358	138.57086	I	3209.39389	1605.20058	1070.46948	803.10393	642.68460	535.73838	27
7	954.44738	477.72733	318.82065	239.36730	191.69530	159.91396	Q	3096.30983	1548.65855	1032.77479	774.83291	620.06779	516.89103	26
8	1114.47803	557.74265	372.16419	279.37497	223.70143	186.58574	C-Carbamidomethyl	2968.25125	1484.62926	990.08860	742.81827	594.45607	495.54794	25
9	1277.54136	639.27432	426.51864	320.14080	256.31409	213.76296	Y	2808.22060	1404.61394	936.74505	702.81061	562.44994	468.87616	24
10	1376.60977	688.80853	459.54144	344.90790	276.12778	230.27436	V	2645.15727	1323.08227	882.39061	662.04478	529.83728	441.69894	23
11	1490.65270	745.82999	497.55575	373.41863	298.93636	249.28151	N	2546.08886	1273.54807	849.36780	637.27767	510.02359	425.18754	22
12	1547.67416	774.34072	516.56291	387.67400	310.34065	258.78509	G	2432.04593	1216.52660	811.35349	608.76694	487.21501	406.18039	21
13	1675.73274	838.37001	559.24910	419.68864	335.95237	280.12819	Q	2375.02447	1188.01587	792.34634	594.51157	475.81071	396.67681	20
14	1788.81681	894.91204	596.94379	447.95966	358.56918	298.97553	L	2246.96589	1123.98658	749.66015	562.49693	450.19900	375.33371	19
15	1887.88522	944.44625	629.96659	472.72676	378.38287	315.48693	V	2133.88182	1067.44455	711.96546	534.22591	427.58219	356.48637	18
16	1974.91725	987.96226	658.97727	494.48477	395.78927	329.99227	S	2034.81341	1017.91034	678.94265	509.45881	407.76850	339.97497	17
17	2137.98058	1069.49393	713.33171	535.25060	428.40194	357.16949	Y	1947.78138	974.39433	649.93198	487.70080	390.36210	325.46963	16
18	2195.00204	1098.00466	732.33886	549.50597	439.80623	366.67307	G	1784.71805	892.86267	595.57754	446.93497	357.74943	298.29241	15
19	2310.02898	1155.51813	770.68118	578.26270	462.81162	385.84423	D	1727.69659	864.35193	576.57038	432.67960	346.34514	288.78883	14
20	2441.06947	1221.03837	814.36134	611.02282	489.01971	407.68431	M	1612.66965	806.83846	538.22807	403.92287	323.33975	269.61767	13
21	2512.10658	1256.55693	838.04038	628.78210	503.22714	419.52383	A	1481.62916	741.31822	494.54791	371.16275	297.13165	247.77759	12
22	2702.18081	1351.59404	901.39845	676.30066	541.24198	451.20287	W-Trp->Kynurenin	1410.59205	705.79966	470.86887	353.40347	282.92423	235.93807	11
23	2839.23972	1420.12350	947.08476	710.56539	568.65377	474.04602	H	1220.51782	610.76255	407.51079	305.88491	244.90939	204.25903	10
24	2938.30814	1469.65771	980.10756	735.33249	588.46745	490.55742	V	1083.45891	542.23309	361.82449	271.62018	217.49760	181.41588	9
25	3052.35106	1526.67917	1018.12187	763.84322	611.27603	509.56457	N	984.39050	492.69889	328.80168	246.85308	197.68392	164.90448	8
26	3153.39874	1577.20301	1051.80443	789.10514	631.48557	526.40585	T	870.34757	435.67742	290.78737	218.34235	174.87533	145.89733	7
27	3267.44167	1634.22447	1089.81874	817.61587	654.29415	545.41301	N	769.29989	385.15358	257.10481	193.08043	154.66580	129.05605	6
28	3382.46861	1691.73794	1128.16105	846.37261	677.29954	564.58417	D	655.25696	328.13212	219.09051	164.56970	131.85721	110.04889	5
29	3469.50064	1735.25396	1157.17173	868.13062	694.70595	579.08950	S	540.23002	270.61865	180.74819	135.81296	108.85183	90.87773	4
30	3632.56397	1816.78562	1211.52617	908.89645	727.31861	606.26673	Y	453.19799	227.10263	151.73751	114.05496	91.44542	76.37240	3
31	3747.59091	1874.29909	1249.86849	937.65319	750.32400	625.43788	D	290.13466	145.57097	97.38307	73.28912	58.83275	49.19517	2
32							K-Formyl	175.10772	88.05750	59.04076	44.53239	35.82737	30.02402	1

131

Sequence: TKEKYIDQEELNKTKPIWTR, W18-Trp->Hydroxykynurenin (19.98983 Da), K13-Carboxyethyl (72.02113 Da), K2-HydroxymethylOP (108.02113 Da)
Charge: +6, Monoisotopic m/z: 454.23401 Da (-1.68 mmu/-3.71 ppm), MH+: 2720.36767 Da, RT: 54.5351 min,
Identified with: Sequest HT (v1.17); XCorr:0.96, Percolator q-Value:6.3e-3, Percolator PEP:1.4e-2, ptmRS: Best Site Probabilities:K2(Carboxyethyl): 84.96,
Fragment match tolerance used for search: 0.02 Da
Fragments used for search: -H_2O; y; -NH_3; y; b; b; -H_2O; b; -NH_3; y

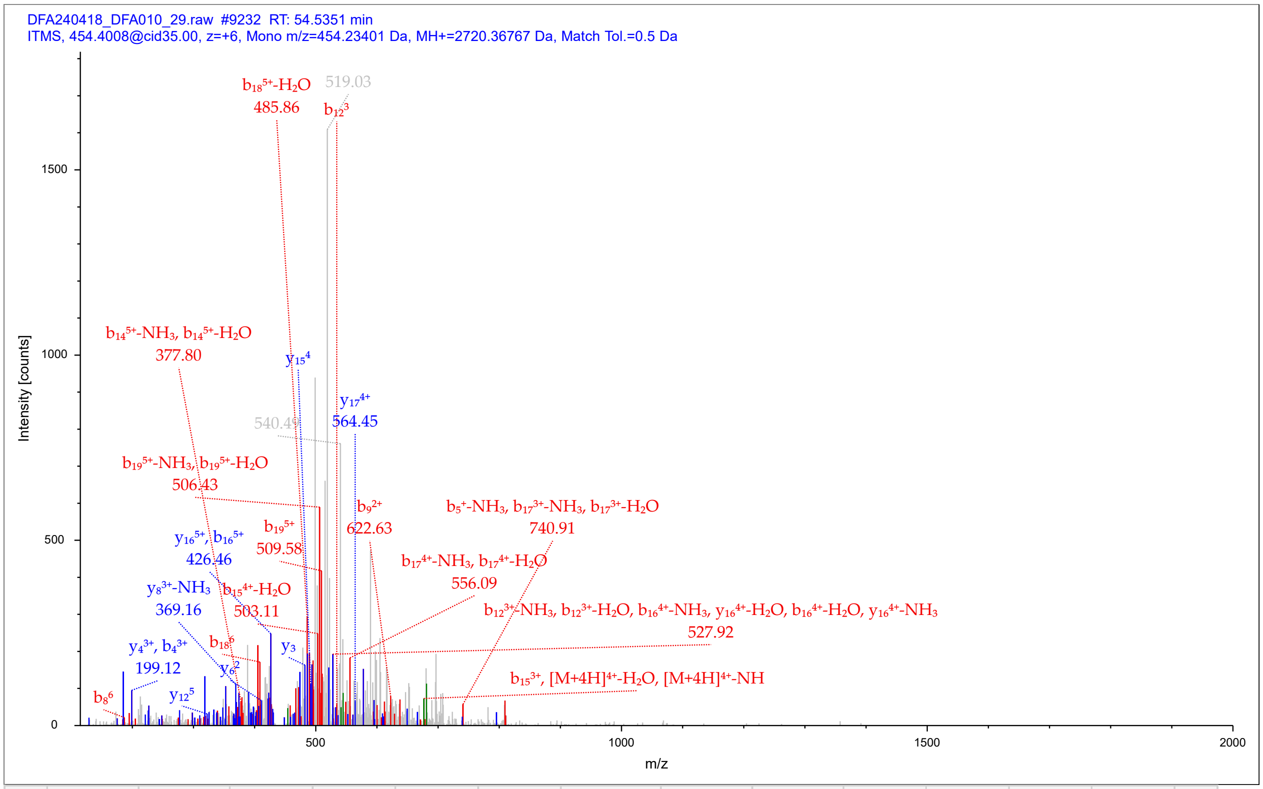

#1	b⁺	b²⁺	b³⁺	b⁴⁺	b⁵⁺	b⁶⁺	Seq.	y⁺	y²⁺	y³⁺	y⁴⁺	y⁵⁺	y⁶⁺	#2
1	102.05496	51.53112	34.68984	26.26920	21.21681	17.84856	T							20
2	338.17105	169.58916	113.39520	85.29822	68.44003	57.20124	K-Hydroxy-methylOP	2619.33007	1310.16867	873.78154	655.58798	524.67184	437.39441	19
3	467.21364	234.11046	156.40940	117.55887	94.24855	78.70834	E	2383.21398	1192.11063	795.07618	596.55895	477.44862	398.04173	18
4	595.30860	298.15794	199.10772	149.58261	119.86754	100.05750	K	2254.17139	1127.58933	752.06198	564.29830	451.64010	376.53463	17
5	758.37193	379.68960	253.46216	190.34844	152.48021	127.23472	Y	2126.07642	1063.54185	709.36366	532.27456	426.02111	355.18547	16
6	871.45600	436.23164	291.15685	218.61946	175.09702	146.08206	I	1963.01310	982.01019	655.00922	491.50873	393.40844	328.00825	15
7	986.48294	493.74511	329.49916	247.37619	198.10241	165.25322	D	1849.92903	925.46815	617.31453	463.23772	370.79163	309.16090	14
8	1114.54152	557.77440	372.18536	279.39084	223.71412	186.59632	Q	1734.90209	867.95468	578.97221	434.48098	347.78624	289.98975	13
9	1243.58411	622.29569	415.19955	311.65148	249.52264	208.10342	E	1606.84351	803.92539	536.28602	402.46634	322.17452	268.64665	12
10	1372.62670	686.81699	458.21375	343.91213	275.33116	229.61051	E	1477.80092	739.40410	493.27182	370.20569	296.36600	247.13955	11
11	1485.71077	743.35902	495.90844	372.18315	297.94797	248.45786	L	1348.75832	674.88280	450.25763	337.94504	270.55749	225.63245	10
12	1599.75369	800.38048	533.92275	400.69388	320.75656	267.46501	N	1235.67426	618.34077	412.56294	309.67402	247.94067	206.78511	9
13	1799.86979	900.43853	600.62811	450.72290	360.77978	300.81769	K-Carboxy-ethyl	1121.63133	561.31931	374.54863	281.16329	225.13209	187.77795	8
14	1900.91746	950.96237	634.31067	475.98482	380.98931	317.65897	T	921.51524	461.26126	307.84326	231.13427	185.10887	154.42527	7
15	2029.01243	1015.00985	677.00899	508.00856	406.60831	339.00813	K	820.46756	410.73742	274.16071	205.87235	164.89933	137.58399	6
16	2126.06519	1063.53623	709.35991	532.27176	426.01886	355.18360	P	692.37260	346.68994	231.46238	173.84861	139.28034	116.23483	5
17	2239.14925	1120.07827	747.05460	560.54277	448.63567	374.03094	I	595.31984	298.16356	199.11146	149.58542	119.86979	100.05937	4
18	2445.21840	1223.11284	815.74432	612.06006	489.84950	408.37580	W-Trp->Hydroxy-kynurenin	482.23577	241.62152	161.41678	121.31440	97.25298	81.21203	3
19	2546.26607	1273.63668	849.42688	637.32198	510.05904	425.21708	T	276.16663	138.58695	92.72706	69.79712	56.03915	46.86717	2
20							R	175.11895	88.06311	59.04450	44.53520	35.82961	30.02589	1

Sequence: LLIYGASSR, R9-Carboxyethyl (72.02113 Da)
Charge: +2, Monoisotopic m/z: 526.29309 Da (+0.34 mmu/+0.64 ppm), MH+: 1051.57891 Da, RT: 55.2309 min,
Identified with: Sequest HT (v1.17); XCorr:0.65, Percolator q-Value:5.4e-3, Percolator PEP:4.0e-2,
Fragment match tolerance used for search: 0.02 Da
Fragments used for search: -H_2O; y; b; b; -H_2O; y

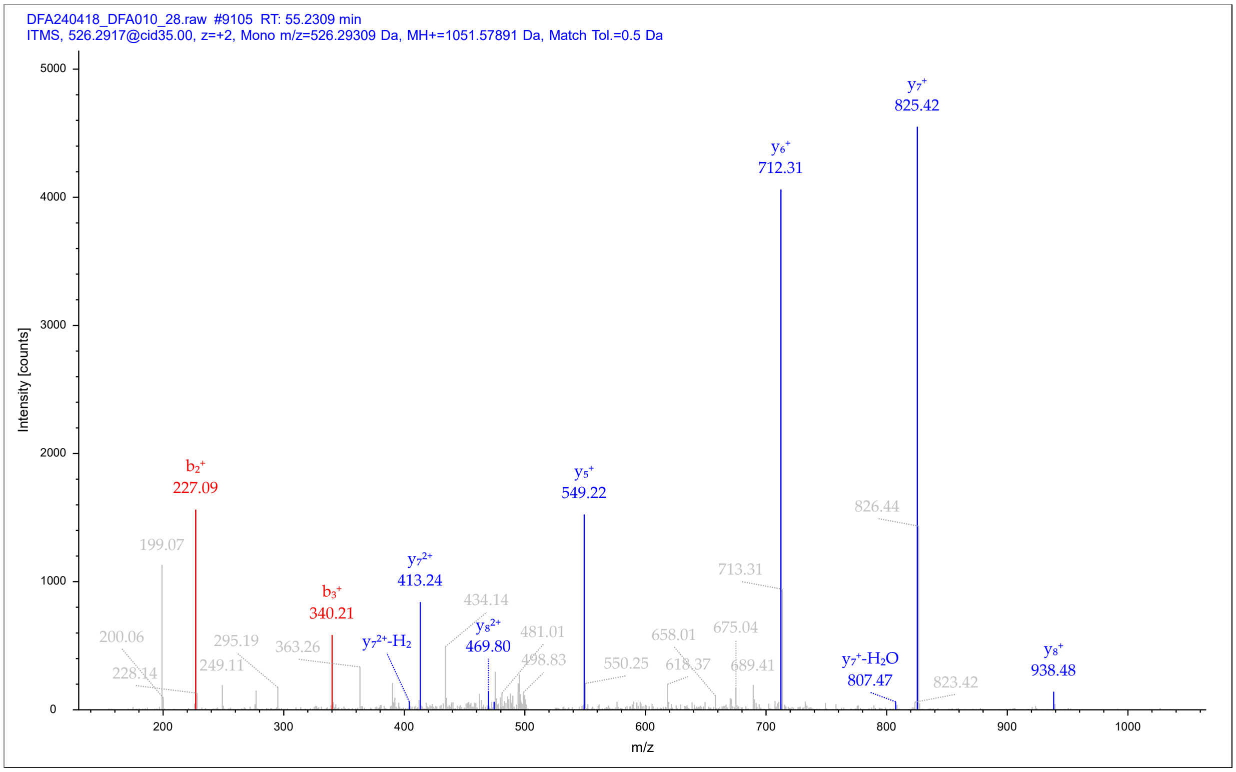

#1	b⁺	b²⁺	Seq.	y⁺	y²⁺	#2
1	114.09134	57.54931	L			9
2	227.17540	114.09134	L	938.49417	469.75072	8
3	340.25947	170.63337	I	825.41011	413.20869	7
4	503.32280	252.16504	Y	712.32604	356.66666	6
5	560.34426	280.67577	G	549.26272	275.13500	5
6	631.38137	316.19433	A	492.24125	246.62426	4
7	718.41340	359.71034	S	421.20414	211.10571	3
8	805.44543	403.22635	S	334.17211	167.58969	2
9			R-Carboxy ethyl	247.14008	124.07368	1

Sequence: KLSSWVLLMK, M9-Oxidation (15.99492 Da), W5-Trp->Kynurenin (3.99492 Da)
Charge: +2, Monoisotopic m/z: 612.85382 Da (-0.85 mmu/-1.38 ppm), MH+: 1224.70036 Da, RT: 59.0489 min,
Identified with: Sequest HT (v1.17); XCorr:0.74, Percolator q-Value:3.3e-3, Percolator PEP:5.3e-2,
Fragment match tolerance used for search: 0.02 Da
Fragments used for search: -H_2O; y; -NH_3; y; b; b; -H_2O; b; -NH_3; y

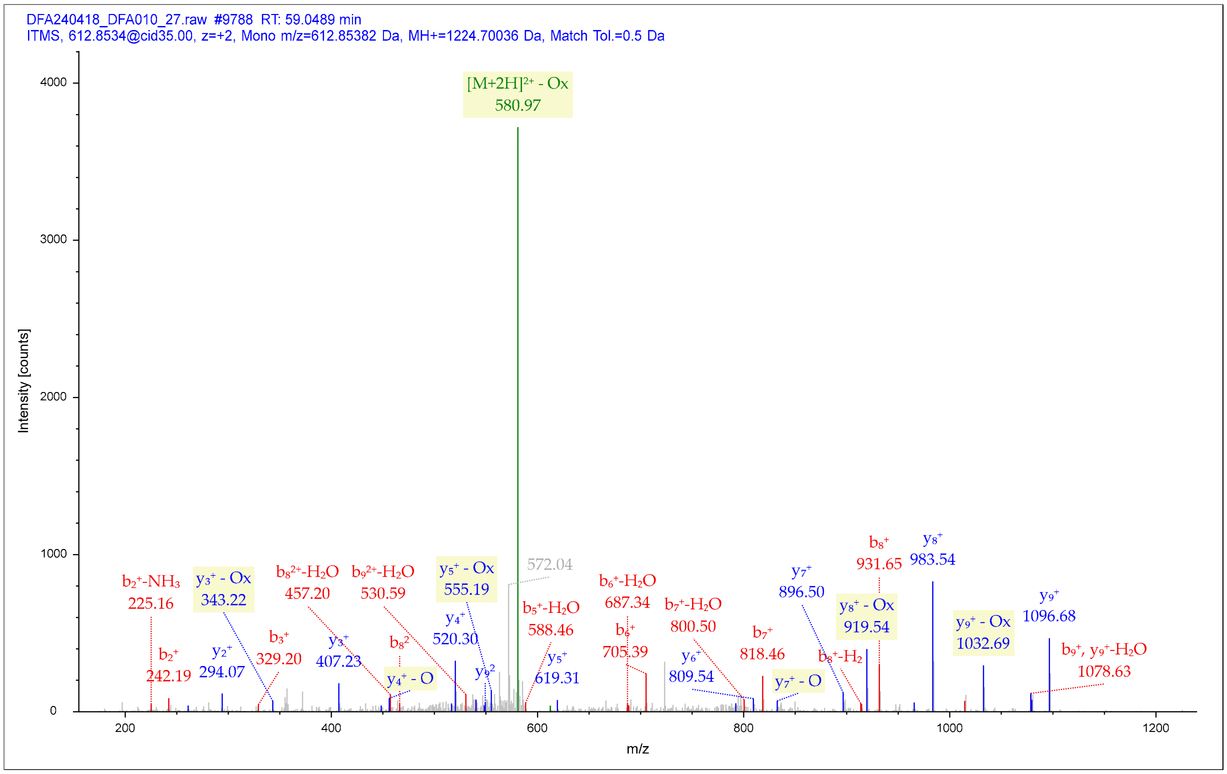

#1	b⁺	b²⁺	Seq.	y⁺	y²⁺	#2
1	129.10224	65.05476	K			10
2	242.18630	121.59679	L	1096.60709	548.80719	9
3	329.21833	165.11280	S	983.52303	492.26515	8
4	416.25036	208.62882	S	896.49100	448.74914	7
5	606.32459	303.66593	W-Trp-> Kynurenin	809.45897	405.23313	6
6	705.39300	353.20014	V	619.38475	310.19601	5
7	818.47707	409.74217	L	520.31633	260.66180	4
8	931.56113	466.28420	L	407.23227	204.11977	3
9	1078.59653	539.80190	M-Oxidation	294.14820	147.57774	2
10			K	147.11280	74.06004	1

60

Sequence: KPDILSIK, K1-Carboxyethyl (72.02113 Da)
Charge: +2, Monoisotopic m/z: 493.30017 Da (+0.12 mmu/+0.25 ppm), MH+: 985.59307 Da, RT: 67.0998 min,
Identified with: Sequest HT (v1.17); XCorr:0.46, Percolator q-Value:3.9e-3, Percolator PEP:3.9e-2, ptmRS: Best Site Probabilities:K1(Carboxyethyl): 100,
Fragment match tolerance used for search: 0.02 Da
Fragments used for search: -H_2O; y; -NH_3; y; b; b; -H_2O; y

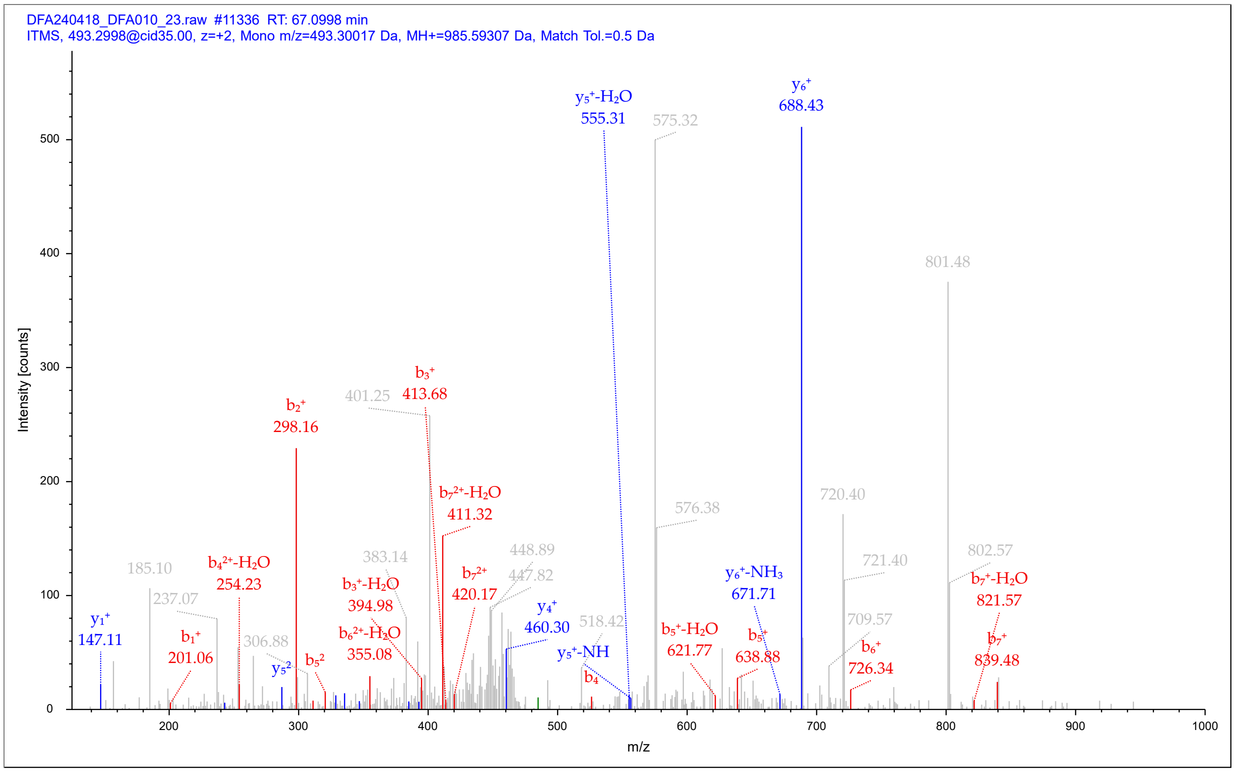

#1	b+	b2+	Seq.	y+	y2+	#2
1	201.12337	101.06532	K-Carboxy-ethyl			8
2	298.17613	149.59170	P	785.47673	393.24200	7
3	413.20308	207.10518	D	688.42397	344.71562	6
4	526.28714	263.64721	I	573.39702	287.20215	5
5	639.37120	320.18924	L	460.31296	230.66012	4
6	726.40323	363.70525	S	347.22890	174.11809	3
7	839.48730	420.24729	I	260.19687	130.60207	2
8			K	147.11280	74.06004	1

Sequence: FDEFFSEGCAPGSK, C9-Carbamidomethyl (57.02146 Da), K14-Ethanalyl (42.01830 Da)
Charge: +2, Monoisotopic m/z: 810.34308 Da (+1.45 mmu/+1.79 ppm), MH+: 1619.67888 Da, RT: 69.8170 min,
Identified with: Sequest HT (v1.17); XCorr:0.92, Percolator q-Value:6.4e-3, Percolator PEP:4.0e-2,
Fragment match tolerance used for search: 0.02 Da
Fragments used for search: -H_2O; y; b; b; -H_2O; y

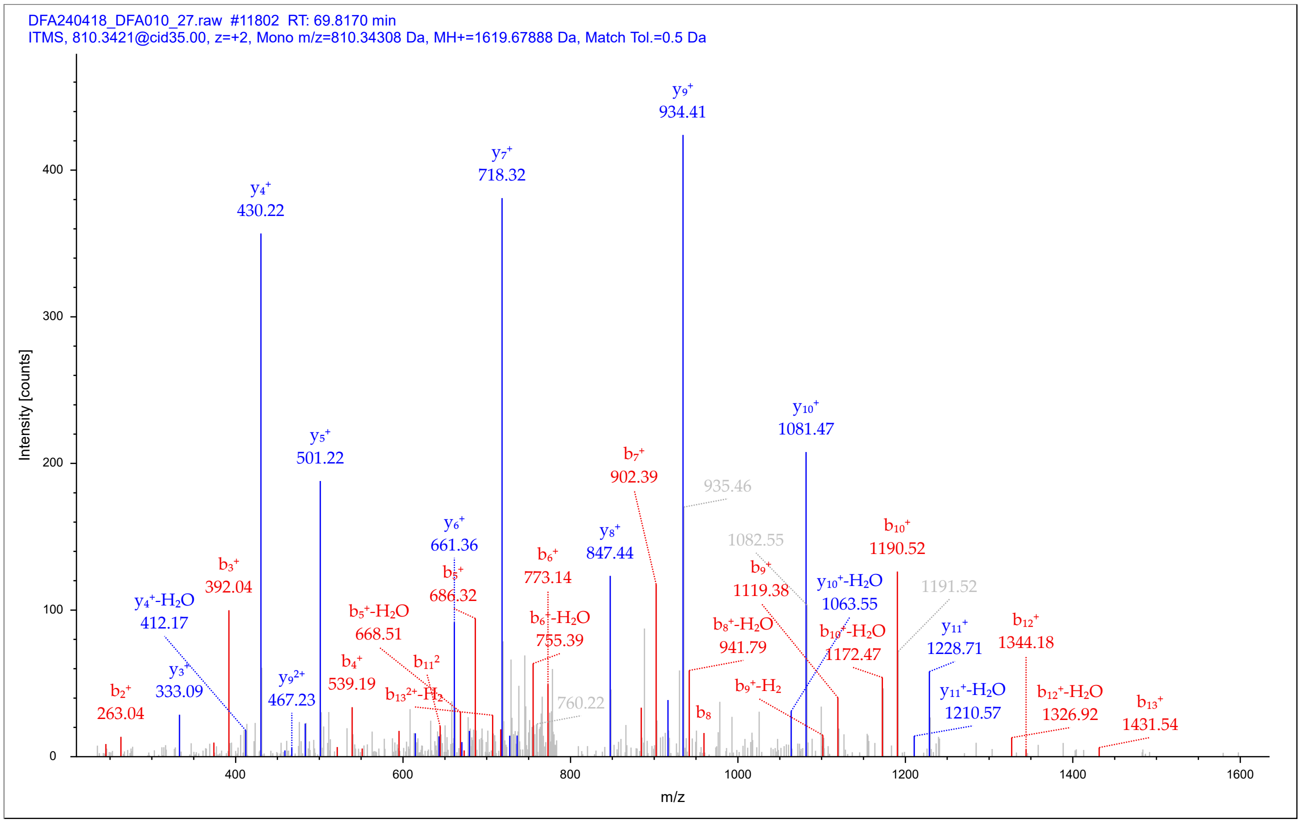

#1	b+	b2+	Seq.	y+	y2+	#2
1	148.07569	74.54148	F			14
2	263.10263	132.05496	D	1472.60757	736.80742	13
3	392.14523	196.57625	E	1357.58063	679.29395	12
4	539.21364	270.11046	F	1228.53804	614.77266	11
5	686.28205	343.64467	F	1081.46962	541.23845	10
6	773.31408	387.16068	S	934.40121	467.70424	9
7	902.35668	451.68198	E	847.36918	424.18823	8
8	959.37814	480.19271	G	718.32659	359.66693	7
9	1119.40879	560.20803	C-Carbamido-methyl	661.30512	331.15620	6
10	1190.44590	595.72659	A	501.27447	251.14088	5
11	1287.49867	644.25297	P	430.23736	215.62232	4
12	1344.52013	672.76370	G	333.18460	167.09594	3
13	1431.55216	716.27972	S	276.16313	138.58520	2
14			K-Ethanalyl	189.13110	95.06919	1

Sequence: YYYAVYDMVVR, Y1-Oxidation (15.99492 Da), Y2-Oxidation (15.99492 Da), Y6-Trioxidation (47.98474 Da), R11-Carboxyethyl (72.02113 Da)
Charge: +3, Monoisotopic m/z: 531.89685 Da (-0.59 mmu/-1.1 ppm), MH+: 1593.67600 Da, RT: 22.3041 min,
Identified with: Sequest HT (v1.17); XCorr:0.63, Percolator q-Value:8.4e-3, Percolator PEP:4.5e-2,
Fragment match tolerance used for search: 0.02 Da
Fragments used for search: -H_2O; y; b; b; -H_2O; y

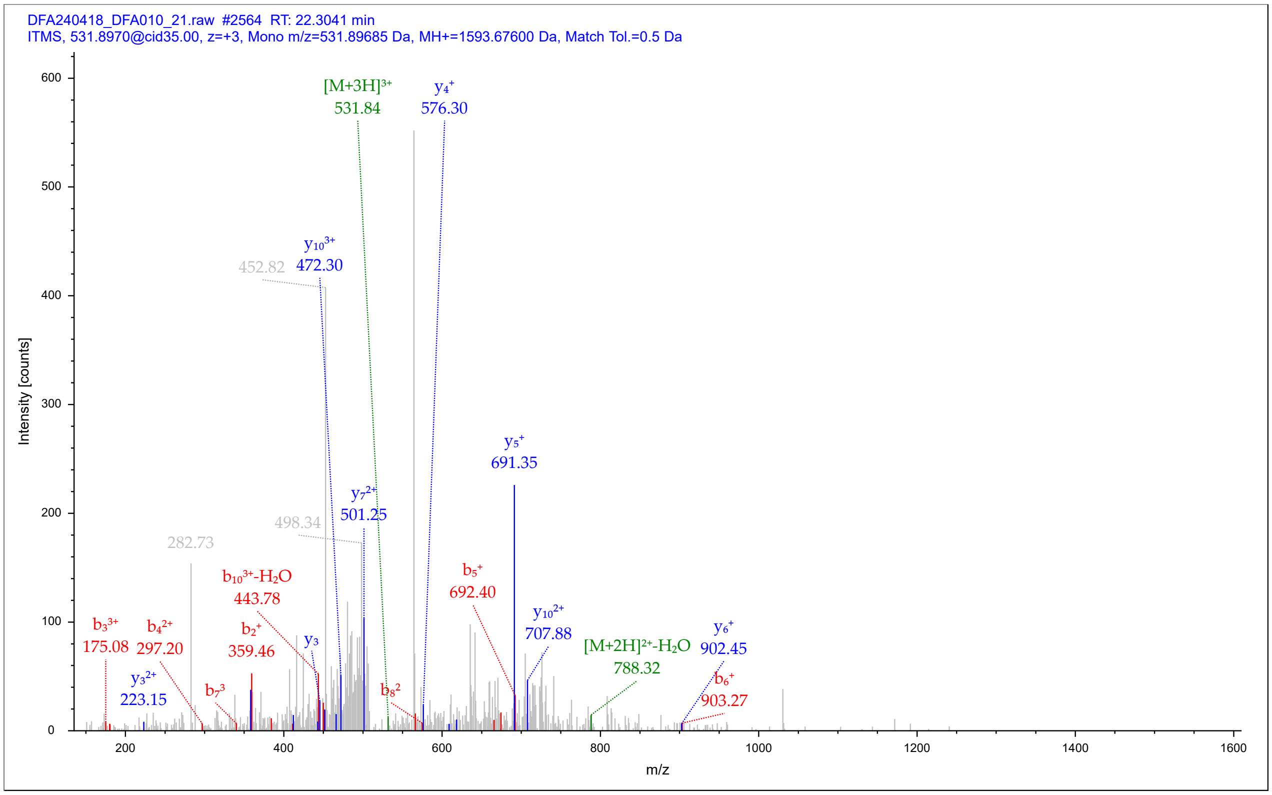

#1	b⁺	b²⁺	b³⁺	Seq.	y⁺	y²⁺	y³⁺	#2
1	180.06552	90.53640	60.69336	Y-Oxidation				11
2	359.12376	180.06552	120.37944	Y-Oxidation	1414.61951	707.81339	472.21135	10
3	522.18709	261.59718	174.73388	Y	1235.56127	618.28427	412.52527	9
4	593.22421	297.11574	198.41292	A	1072.49794	536.75261	358.17083	8
5	692.29262	346.64995	231.43572	V	1001.46082	501.23405	334.49179	7
6	903.34069	452.17398	301.78508	Y-Trioxidation	902.39241	451.69984	301.46899	6
7	1018.36764	509.68746	340.12740	D	691.34434	346.17581	231.11963	5
8	1149.40812	575.20770	383.80756	M	576.31739	288.66234	192.77732	4
9	1248.47653	624.74191	416.83036	V	445.27691	223.14209	149.09715	3
10	1347.54495	674.27611	449.85317	V	346.20850	173.60789	116.07435	2
11				R-Carboxy-ethyl	247.14008	124.07368	83.05154	1

Sequence: THTGEKPYKCTWEGCDWRFAR, C10-Carbamidomethyl (57.02146 Da), C15-Carbamidomethyl (57.02146 Da), K6-Carboxymethyl (58.00548 Da), W17-Trp->Kynurenin (3.99492 Da), R21-Triosyl (72.01840 Da)
Charge: +6, Monoisotopic m/z: 470.71027 Da (-0.35 mmu/-0.75 ppm), MH+: 2819.22521 Da, RT: 20.5289 min,
Identified with: Sequest HT (v1.17); XCorr:0.85, Percolator q-Value:4.5e-3, Percolator PEP:2.8e-2,
Fragment match tolerance used for search: 0.02 Da
Fragments used for search: -H_2O; y; -NH_3; y; b; b; -H_2O; b; -NH_3; y

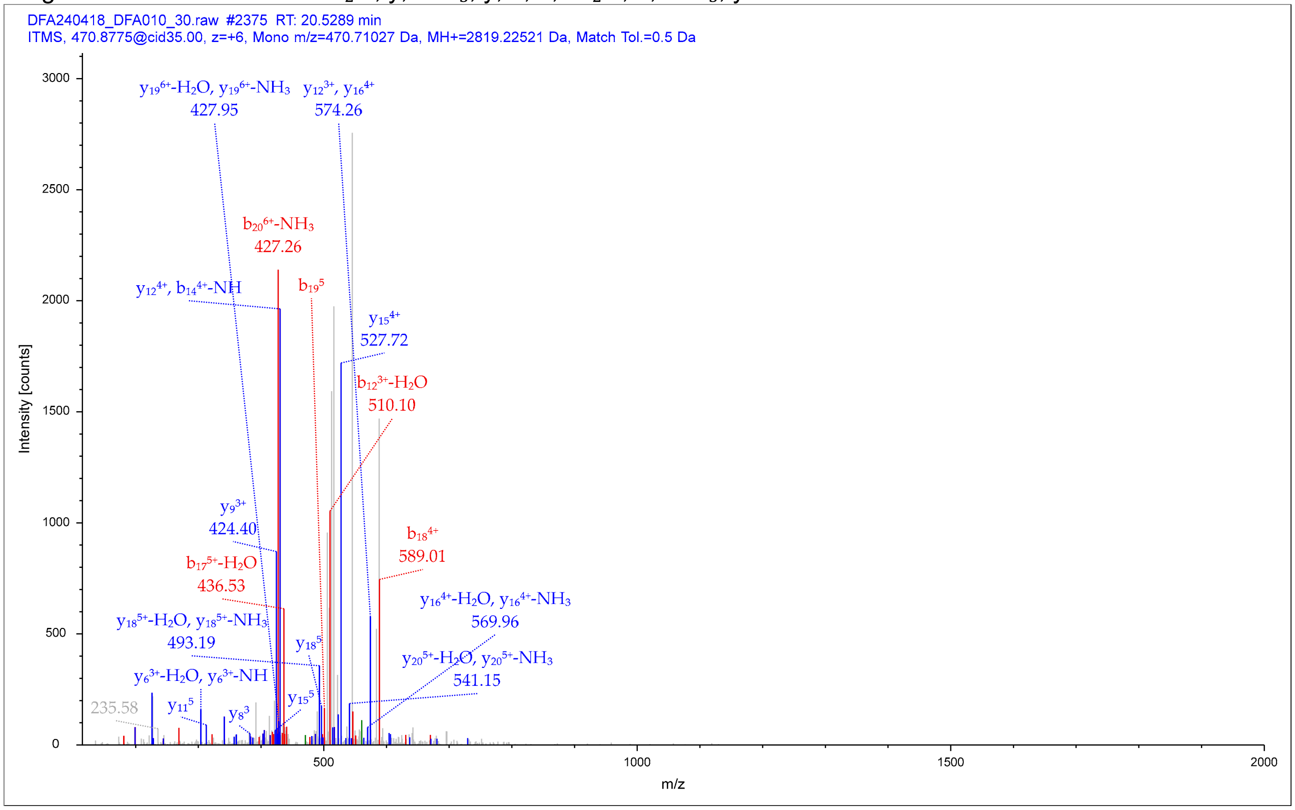

#1	b⁺	b²⁺	b³⁺	b⁴⁺	b⁵⁺	b⁶⁺	Seq.	y⁺	y²⁺	y³⁺	y⁴⁺	y⁵⁺	y⁶⁺	#2
1	102.05496	51.53112	34.68984	26.26920	21.21681	17.84856	T							21
2	239.11387	120.06057	80.37614	60.53392	48.62859	40.69171	H	2718.17965	1359.59346	906.73140	680.30037	544.44175	453.86934	20
3	340.16155	170.58441	114.05870	85.79584	68.83813	57.53299	T	2581.12074	1291.06401	861.04510	646.03564	517.02997	431.02619	19
4	397.18301	199.09514	133.06585	100.05121	80.24242	67.03657	G	2480.07306	1240.54017	827.36254	620.77372	496.82043	414.18491	18
5	526.22560	263.61644	176.08005	132.31186	106.05094	88.54366	E	2423.05160	1212.02944	808.35538	606.51836	485.41614	404.68133	17
6	712.32604	356.66666	238.11353	178.83697	143.27103	119.56040	K-Carboxy-methyl	2294.00900	1147.50814	765.34119	574.25771	459.60762	383.17423	16
7	809.37881	405.19304	270.46445	203.10016	162.68158	135.73587	P	2107.90856	1054.45792	703.30771	527.73260	422.38753	352.15749	15
8	972.44214	486.72471	324.81890	243.86599	195.29425	162.91309	Y	2010.85580	1005.93154	670.95678	503.46941	402.97698	335.98203	14
9	1100.53710	550.77219	367.51722	275.88973	220.91324	184.26225	K	1847.79247	924.39987	616.60234	462.70357	370.36432	308.80481	13
10	1260.56775	630.78751	420.86077	315.89739	252.91937	210.93402	C-Carbamido-methyl	1719.69751	860.35239	573.90402	430.67983	344.74532	287.45565	12
11	1361.61543	681.31135	454.54333	341.15931	273.12891	227.77530	T	1559.66686	780.33707	520.56047	390.67217	312.73919	260.78387	11
12	1547.69474	774.35101	516.56976	387.67914	310.34477	258.78852	W	1458.61918	729.81323	486.87791	365.41025	292.52966	243.94259	10
13	1676.73733	838.87230	559.58396	419.93979	336.15329	280.29562	E	1272.53987	636.77357	424.85147	318.89042	255.31379	212.92937	9
14	1733.75880	867.38304	578.59112	434.19516	347.55758	289.79920	G	1143.49727	572.25228	381.83728	286.62978	229.50528	191.42228	8
15	1893.78944	947.39836	631.93467	474.20282	379.56371	316.47097	C-Carbamido-methyl	1086.47581	543.74154	362.83012	272.37441	218.10098	181.91870	7
16	2008.81639	1004.91183	670.27698	502.95955	402.56910	335.64213	D	926.44516	463.72622	309.48657	232.36675	186.09485	155.24692	6
17	2198.89061	1099.94895	733.63506	550.47811	440.58394	367.32117	W-Trp->Kynurenin	811.41822	406.21275	271.14426	203.61001	163.08947	136.07577	5
18	2354.99173	1177.99950	785.66876	589.50339	471.80417	393.33802	R	621.34399	311.17563	207.78618	156.09146	125.07462	104.39673	4
19	2502.06014	1251.53371	834.69156	626.27049	501.21785	417.84942	F	465.24288	233.12508	155.75248	117.06618	93.85440	78.37988	3
20	2573.09725	1287.05227	858.37060	644.02977	515.42527	429.68894	A	318.17447	159.59087	106.72967	80.29907	64.44071	53.86847	2
21							R-Triosyl	247.13735	124.07231	83.05064	62.53980	50.23329	42.02896	1

Sequence: SGAQATWTELPWPHEK, W7-Trp->Kynurenin (3.99492 Da)
Charge: +3, Monoisotopic m/z: 614.63226 Da (+0.18 mmu/+0.3 ppm), MH+: 1841.88224 Da, RT: 69.7223 min,
Identified with: Sequest HT (v1.17); XCorr:0.99, Percolator q-Value:9.2e-3, Percolator PEP:6.1e-2,
Fragment match tolerance used for search: 0.02 Da
Fragments used for search: -H_2O; y; -NH_3; y; b; b; -H_2O; b; -NH_3; y

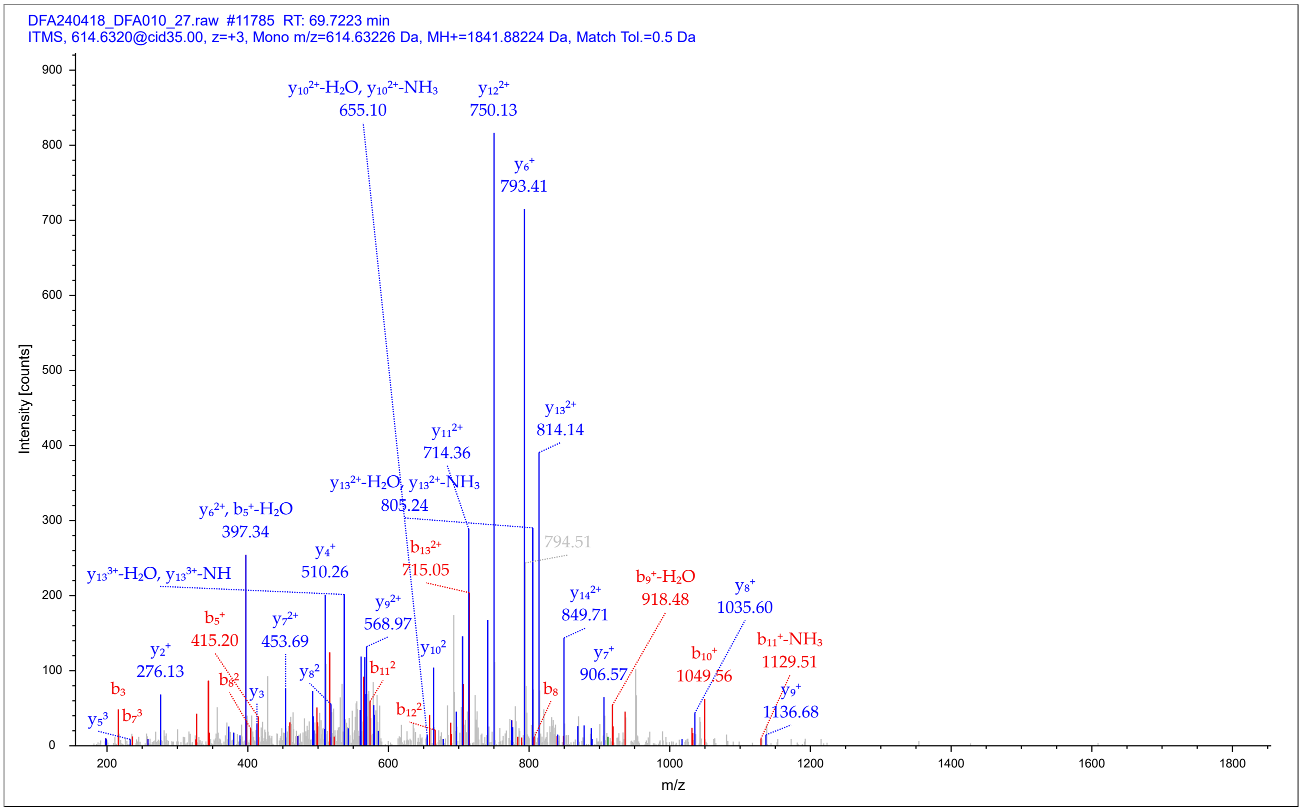

#1	b+	b2+	b3+	Seq.	y+	y2+	y3+	#2
1	88.03930	44.52329	30.01795	S				16
2	145.06077	73.03402	49.02511	G	1754.84966	877.92847	585.62140	15
3	216.09788	108.55258	72.70415	A	1697.82820	849.41774	566.61425	14
4	344.15646	172.58187	115.39034	Q	1626.79108	813.89918	542.93521	13
5	415.19357	208.10043	139.06938	A	1498.73251	749.86989	500.24902	12
6	516.24125	258.62426	172.75194	T	1427.69539	714.35133	476.56998	11
7	706.31548	353.66138	236.11001	W-Trp-> Kynurenin	1326.64771	663.82749	442.88742	10
8	807.36316	404.18522	269.79257	T	1136.57348	568.79038	379.52935	9
9	936.40575	468.70651	312.80677	E	1035.52581	518.26654	345.84679	8
10	1049.48982	525.24855	350.50146	L	906.48321	453.74525	302.83259	7
11	1146.54258	573.77493	382.85238	P	793.39915	397.20321	265.13790	6
12	1332.62189	666.81458	444.87882	W	696.34639	348.67683	232.78698	5
13	1429.67466	715.34097	477.22974	P	510.26707	255.63717	170.76054	4
14	1566.73357	783.87042	522.91604	H	413.21431	207.11079	138.40962	3
15	1695.77616	848.39172	565.93024	E	276.15540	138.58134	92.72332	2
16				K	147.11280	74.06004	49.70912	1

Sequence: WAAEDHVLR, W1-Trioxidation (47.98474 Da), R9-Delta:H(2)C(3)O(1) (54.01057 Da)
Charge: +3, Monoisotopic m/z: 400.18784 Da (+0.08 mmu/+0.19 ppm), MH+: 1198.54895 Da, RT: 16.0559 min,
Identified with: Sequest HT (v1.17); XCorr:0.50, Percolator q-Value:7.3e-3, Percolator PEP:3.9e-2,
Fragment match tolerance used for search: 0.02 Da
Fragments used for search: -H_2O; y; b; b; -H_2O; y

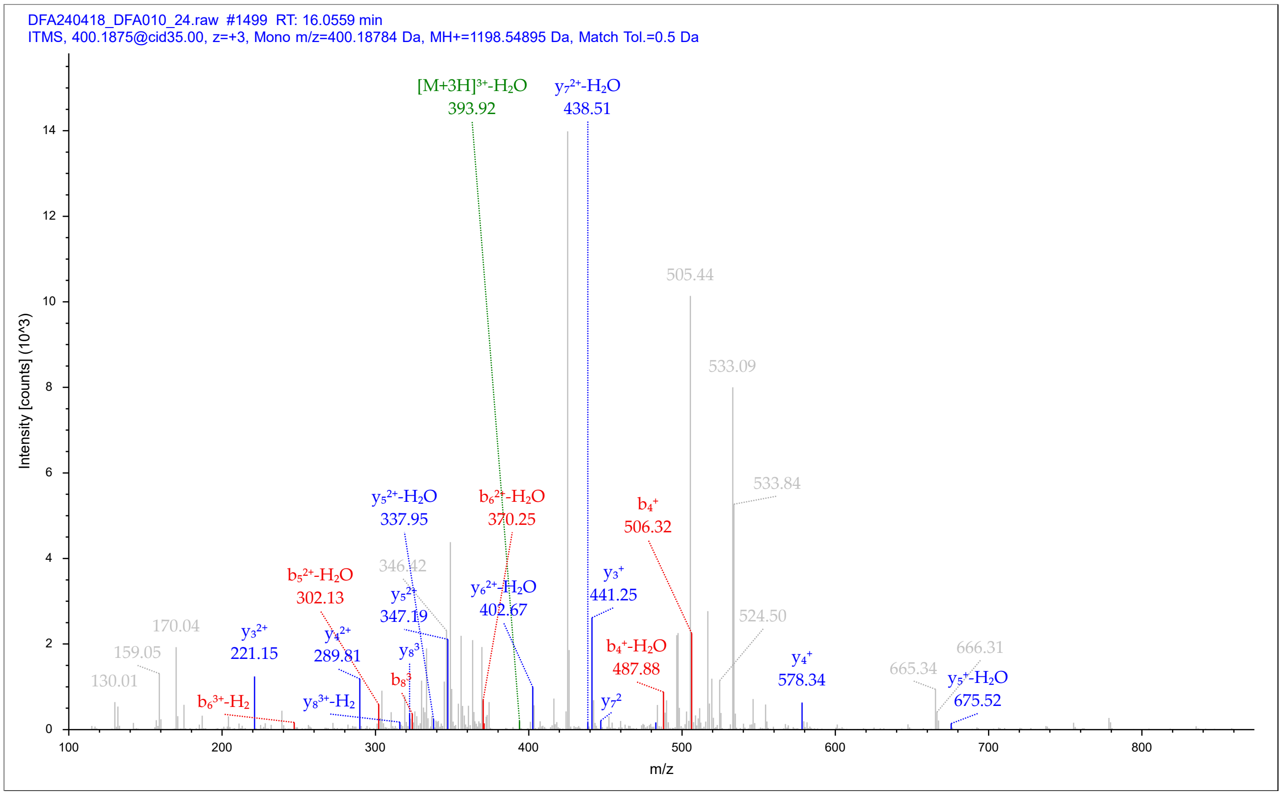

#1	b+	b2+	b3+	Seq.	y+	y2+	y3+	#2
1	235.07133	118.03931	79.02863	W-Trioxidation				9
2	306.10845	153.55786	102.70767	A	964.48467	482.74597	322.16641	8
3	377.14556	189.07642	126.38670	A	893.44756	447.22742	298.48737	7
4	506.18815	253.59772	169.40090	E	822.41044	411.70886	274.80833	6
5	621.21510	311.11119	207.74322	D	693.36785	347.18756	231.79413	5
6	758.27401	379.64064	253.42952	H	578.34091	289.67409	193.45182	4
7	857.34242	429.17485	286.45233	V	441.28200	221.14464	147.76552	3
8	970.42649	485.71688	324.14701	L	342.21358	171.61043	114.74271	2
9				MGH	229.12952	115.06840	77.04802	1

Sequence: TCVADESAENCDK, C2-Carbamidomethyl (57.02146 Da), C11-Carbamidomethyl (57.02146 Da), K13-Ethanalyl (42.01830 Da)
Charge: +2, Monoisotopic m/z: 770.80280 Da (+0.78 mmu/+1.02 ppm), MH+: 1540.59831 Da, RT: 23.3401 min,
Identified with: Sequest HT (v1.17); XCorr:0.67, Percolator q-Value:0.0e0, Percolator PEP:7.8e-4,
Fragment match tolerance used for search: 0.02 Da
Fragments used for search: -H_2O; y; -NH_3; y; b; b; -H_2O; b; -NH_3; y

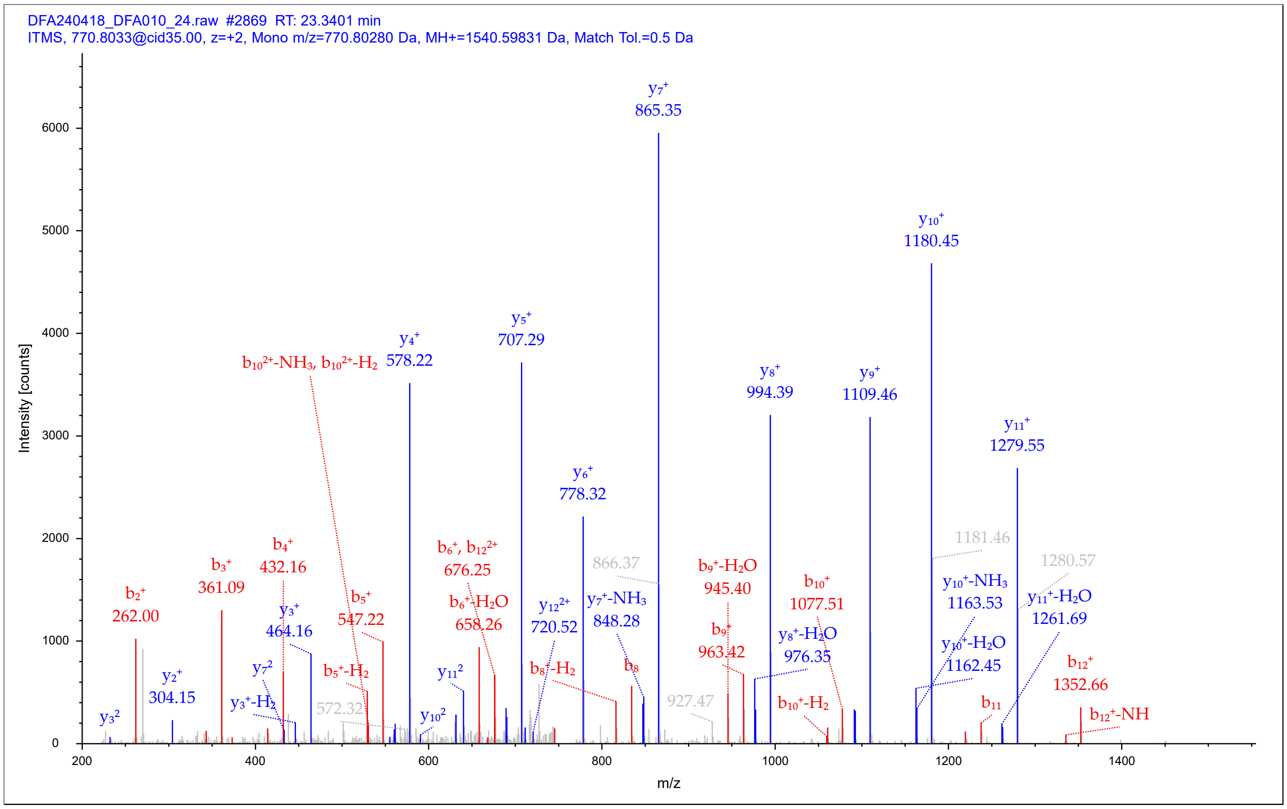

#1	b⁺	b²⁺	Seq.	y⁺	y²⁺	#2
1	102.05496	51.53112	T			13
2	262.08560	131.54644	C-Carbamido-methyl	1439.54907	720.27817	12
3	361.15402	181.08065	V	1279.51842	640.26285	11
4	432.19113	216.59920	A	1180.45001	590.72864	10
5	547.21807	274.11268	D	1109.41289	555.21009	9
6	676.26067	338.63397	E	994.38595	497.69661	8
7	763.29270	382.14999	S	865.34336	433.17532	7
8	834.32981	417.66854	A	778.31133	389.65930	6
9	963.37240	482.18984	E	707.27422	354.14075	5
10	1077.41533	539.21130	N	578.23162	289.61945	4
11	1237.44598	619.22663	C-Carbamido-methyl	464.18870	232.59799	3
12	1352.47292	676.74010	D	304.15805	152.58266	2
13			K-Ethanalyl	189.13110	95.06919	1

Sequence: QFDEIDAWR, W8-Trp->Kynurenin (3.99492 Da), R9-Glarg (39.99949 Da)
Charge: +2, Monoisotopic m/z: 612.27100 Da (-1.3 mmu/-2.13 ppm), MH+: 1223.53472 Da, RT: 30.9776 min,
Identified with: Sequest HT (v1.17); XCorr:0.62, Percolator q-Value:4.9e-3, Percolator PEP:3.7e-2,
Fragment match tolerance used for search: 0.02 Da
Fragments used for search: -H_2O; y; b; b; -H_2O; b; -NH_3; y

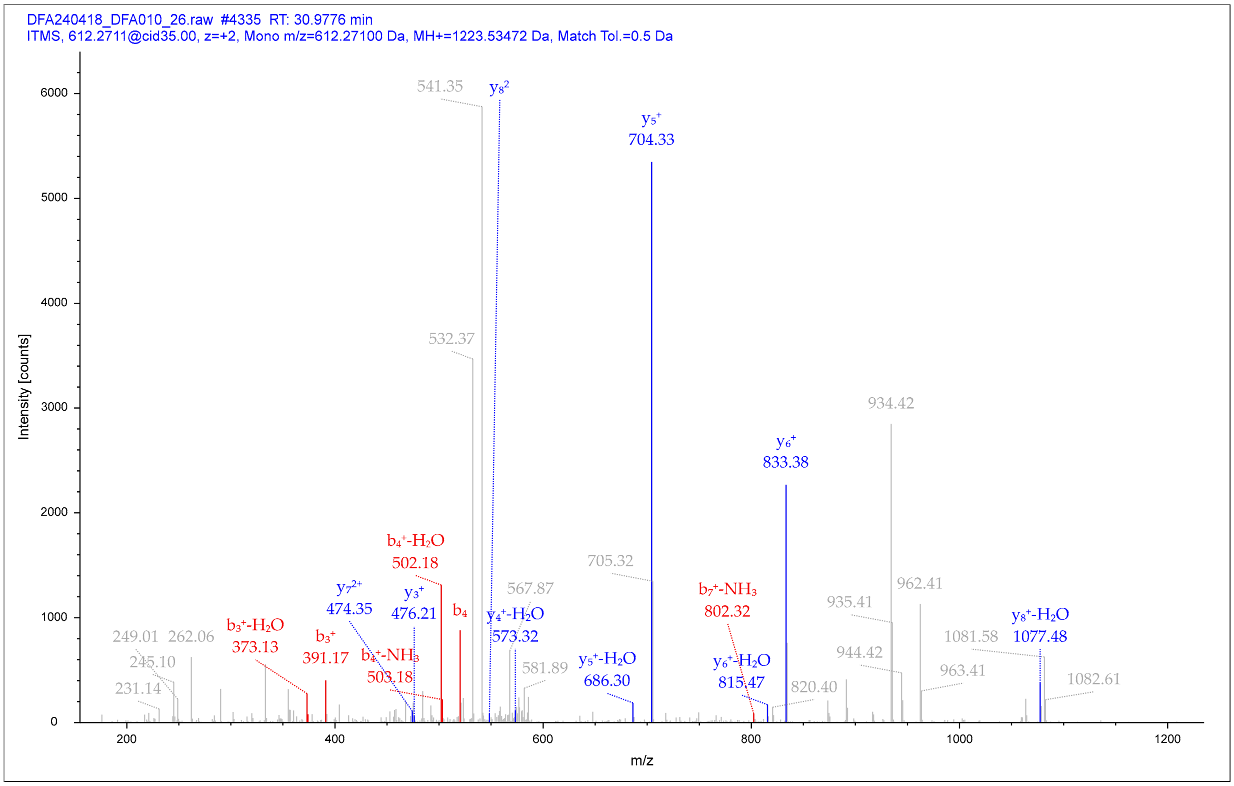

#1	b⁺	b²⁺	Seq.	y⁺	y²⁺	#2
1	129.06585	65.03657	Q			9
2	276.13427	138.57077	F	1095.47874	548.24301	8
3	391.16121	196.08424	D	948.41033	474.70880	7
4	520.20380	260.60554	E	833.38339	417.19533	6
5	633.28787	317.14757	I	704.34079	352.67403	5
6	748.31481	374.66104	D	591.25673	296.13200	4
7	819.35192	410.17960	A	476.22979	238.61853	3
8	1009.42615	505.21671	W-Trp-> Kynurenin	405.19267	203.09997	2
9			R-Glarg	215.11844	108.06286	1

Sequence: QNALYEIEMRK, K11-Acetyl (42.01057 Da), Y5-Oxidation (15.99492 Da), M9-Trioxidation (47.98474 Da), R10-Delta:H(2)C(3)O(1) (54.01057 Da)
Charge: +3, Monoisotopic m/z: 518.90692 Da (-1.41 mmu/-2.73 ppm), MH+: 1554.70621 Da, RT: 32.8988 min,
Identified with: Sequest HT (v1.17); XCorr:1.06, Percolator q-Value:1.8e-3, Percolator PEP:1.3e-2, ptmRS: Best Site Probabilities:K11(Acetyl): 100,
Fragment match tolerance used for search: 0.02 Da
Fragments used for search: $-H_2O$; y; $-NH_3$; y; b; b; $-H_2O$; b; $-NH_3$; y

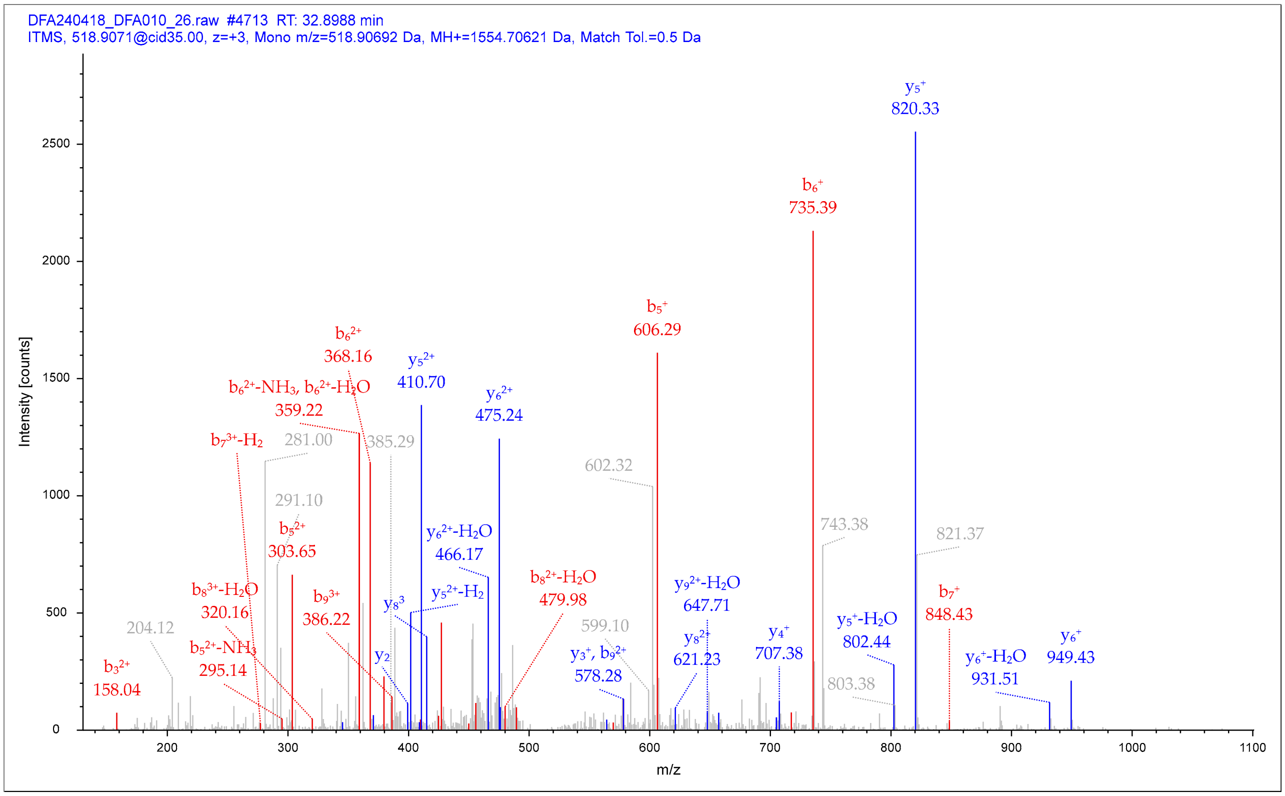

#1	b+	b2+	b3+	Seq.	y+	y2+	y3+	#2
1	129.06585	65.03657	43.69347	Q				11
2	243.10878	122.05803	81.70778	N	1426.65187	713.82957	476.22214	10
3	314.14590	157.57659	105.38682	A	1312.60894	656.80811	438.20783	9
4	427.22996	214.11862	143.08150	L	1241.57183	621.28955	414.52879	8
5	606.28820	303.64774	202.76759	Y-Oxidation	1128.48777	564.74752	376.83411	7
6	735.33080	368.16904	245.78178	E	949.42952	475.21840	317.14803	6
7	848.41486	424.71107	283.47647	I	820.38693	410.69710	274.13383	5
8	977.45745	489.23236	326.49067	E	707.30287	354.15507	236.43914	4
9	1156.48268	578.74498	386.16574	M-Trioxidation	578.26027	289.63378	193.42494	3
10	1366.59436	683.80082	456.20297	MGH	399.23505	200.12116	133.74987	2
11				K-Acetyl	189.12337	95.06532	63.71264	1

Sequence: DLNKKVELELDGER, K4-Acetyl (42.01057 Da), R14-Delta:H(2)C(3)O(1) (54.01057 Da)
Charge: +3, Monoisotopic m/z: 585.30347 Da (-0.27 mmu/-0.47 ppm), MH+: 1753.89585 Da, RT: 37.9544 min,
Identified with: Sequest HT (v1.17); XCorr:1.37, Percolator q-Value:2.8e-3, Percolator PEP:2.1e-2, ptmRS: Best Site Probabilities:K4(Acetyl): 50; K5(Acetyl): 50,
Fragment match tolerance used for search: 0.02 Da
Fragments used for search: -H_2O; y; -NH_3; y; b; b; -H_2O; b; -NH_3; y

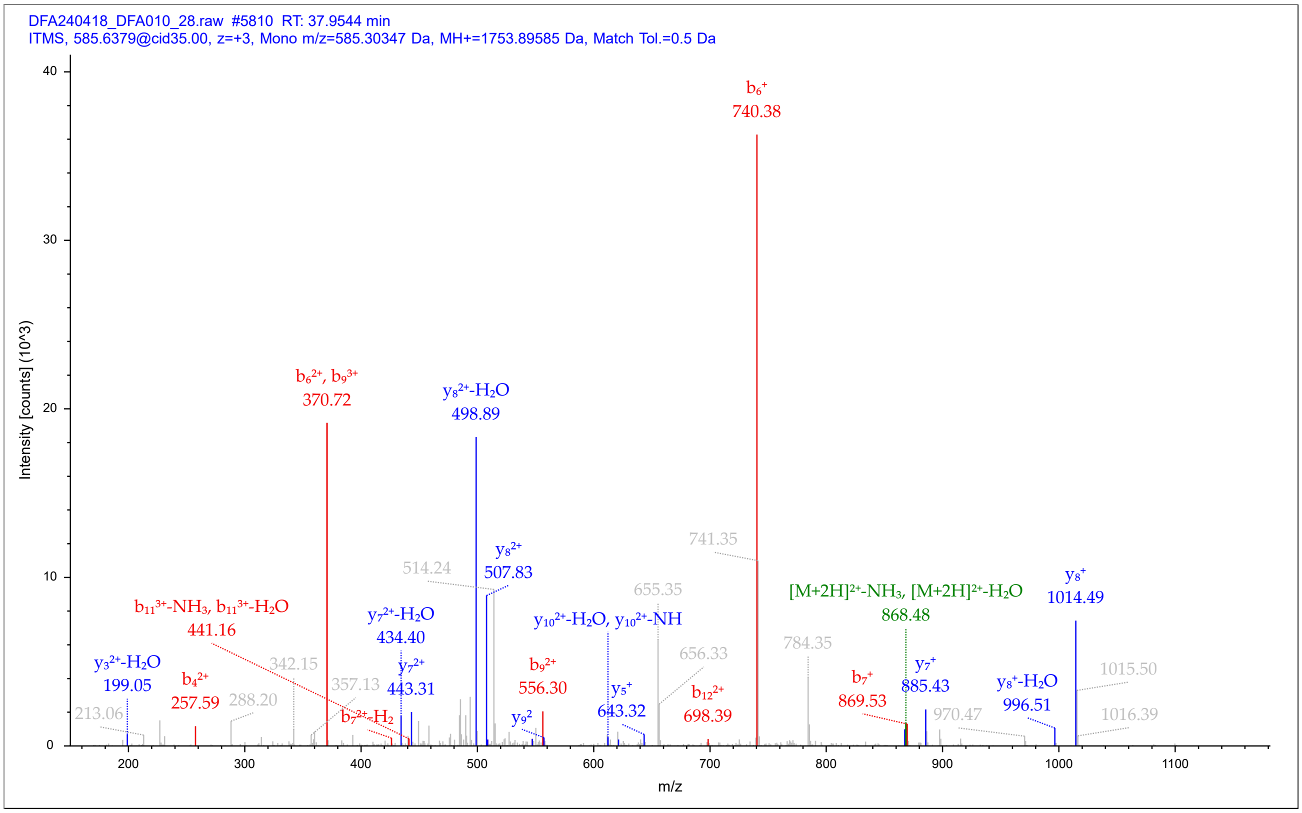

#1	b⁺	b²⁺	b³⁺	Seq.	y⁺	y²⁺	y³⁺	#2
1	116.03422	58.52075	39.34959	D				14
2	229.11828	115.06278	77.04428	L	1638.86973	819.93850	546.96143	13
3	343.16121	172.08424	115.05859	N	1525.78566	763.39647	509.26674	12
4	513.26674	257.13701	171.76043	K-Acetyl	1411.74274	706.37501	471.25243	11
5	641.36170	321.18449	214.45875	K	1241.63721	621.32224	414.55059	10
6	740.43012	370.71870	247.48156	V	1113.54224	557.27476	371.85227	9
7	869.47271	435.23999	290.49575	E	1014.47383	507.74055	338.82946	8
8	982.55677	491.78202	328.19044	L	885.43124	443.21926	295.81526	7
9	1111.59937	556.30332	371.20464	E	772.34717	386.67723	258.12058	6
10	1224.68343	612.84535	408.89933	L	643.30458	322.15593	215.10638	5
11	1339.71037	670.35882	447.24164	D	530.22052	265.61390	177.41169	4
12	1396.73184	698.86956	466.24880	G	415.19357	208.10043	139.06938	3
13	1525.77443	763.39085	509.26299	E	358.17211	179.58969	120.06222	2
14				MGH	229.12952	115.06840	77.04802	1

Sequence: LDFAASQVAVMQR, M11-Dioxidation (31.98983 Da), R13-Delta:H(2)C(3)O(1) (54.01057 Da)
Charge: +3, Monoisotopic m/z: 507.91684 Da (-0.21 mmu/-0.42 ppm), MH+: 1521.73597 Da, RT: 43.2449 min,
Identified with: Sequest HT (v1.17); XCorr:0.82, Percolator q-Value:3.3e-3, Percolator PEP:2.3e-2, ptmRS: Best Site Probabilities:M11(Dioxidation): 100,
Fragment match tolerance used for search: 0.02 Da
Fragments used for search: -H_2O; y; -NH_3; y; b; b; -H_2O; b; -NH_3; y

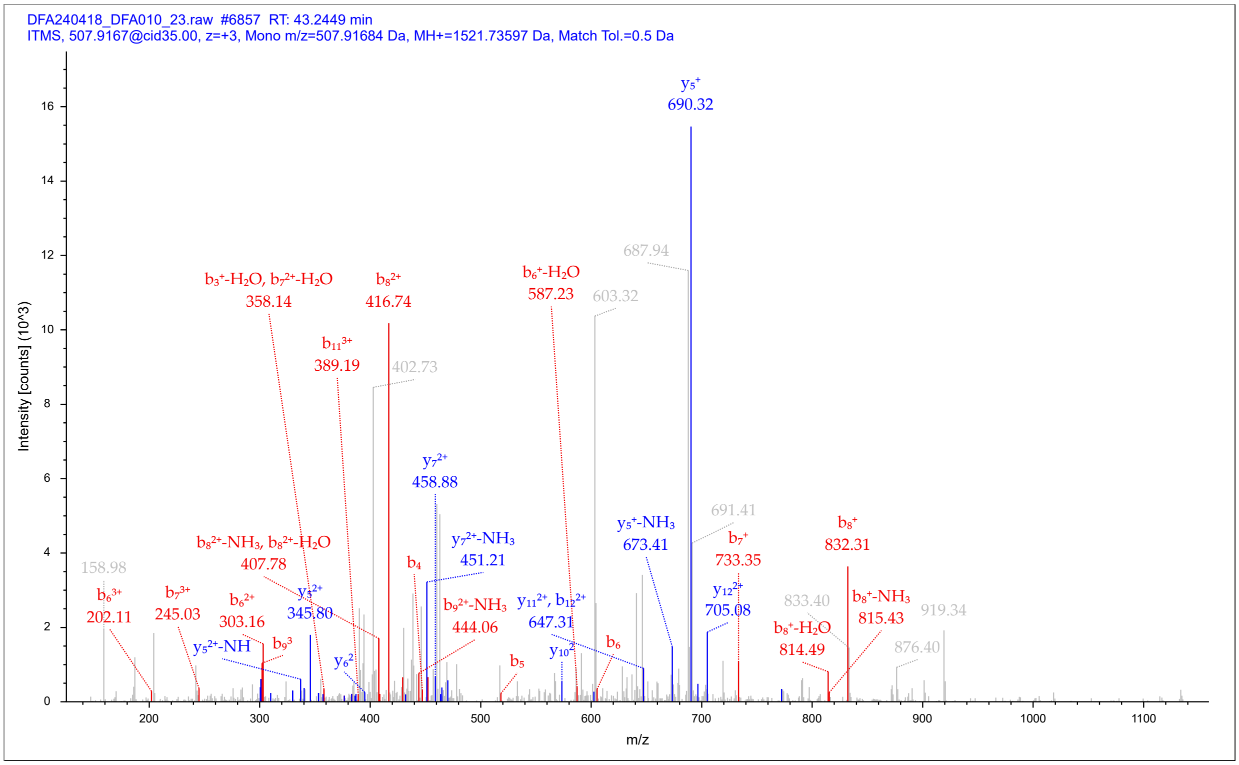

#1	b+	b2+	b3+	Seq.	y+	y2+	y3+	#2
1	114.09134	57.54931	38.70196	L				13
2	229.11828	115.06278	77.04428	D	1408.65254	704.82991	470.22236	12
3	376.18670	188.59699	126.06708	F	1293.62560	647.31644	431.88005	11
4	447.22381	224.11554	149.74612	A	1146.55718	573.78223	382.85725	10
5	518.26092	259.63410	173.42516	A	1075.52007	538.26367	359.17821	9
6	605.29295	303.15011	202.43584	S	1004.48296	502.74512	335.49917	8
7	733.35153	367.17940	245.12203	Q	917.45093	459.22910	306.48849	7
8	832.41994	416.71361	278.14483	V	789.39235	395.19981	263.80230	6
9	903.45706	452.23217	301.82387	A	690.32394	345.66561	230.77950	5
10	1002.52547	501.76637	334.84668	V	619.28682	310.14705	207.10046	4
11	1165.55579	583.28153	389.19011	M-Dioxidation	520.21841	260.61284	174.07765	3
12	1293.61436	647.31082	431.87631	Q	357.18809	179.09769	119.73422	2
13				MGH	229.12952	115.06840	77.04802	1

78_191

Sequence: LYAQRPAGFGCQESR, C11-Carbamidomethyl (57.02146 Da), F9-Oxidation (15.99492 Da), R5-Carboxyethyl (72.02113 Da)
Charge: +4, Monoisotopic m/z: 457.71637 Da (-0.15 mmu/-0.33 ppm), MH+: 1827.84365 Da, RT: 47.1594 min,
Identified with: Sequest HT (v1.17); XCorr:0.76, Percolator q-Value:0.0e0, Percolator PEP:5.2e-3,
Fragment match tolerance used for search: 0.02 Da
Fragments used for search: -H_2O; y; -NH_3; y; b; b; -H_2O; b; -NH_3; y

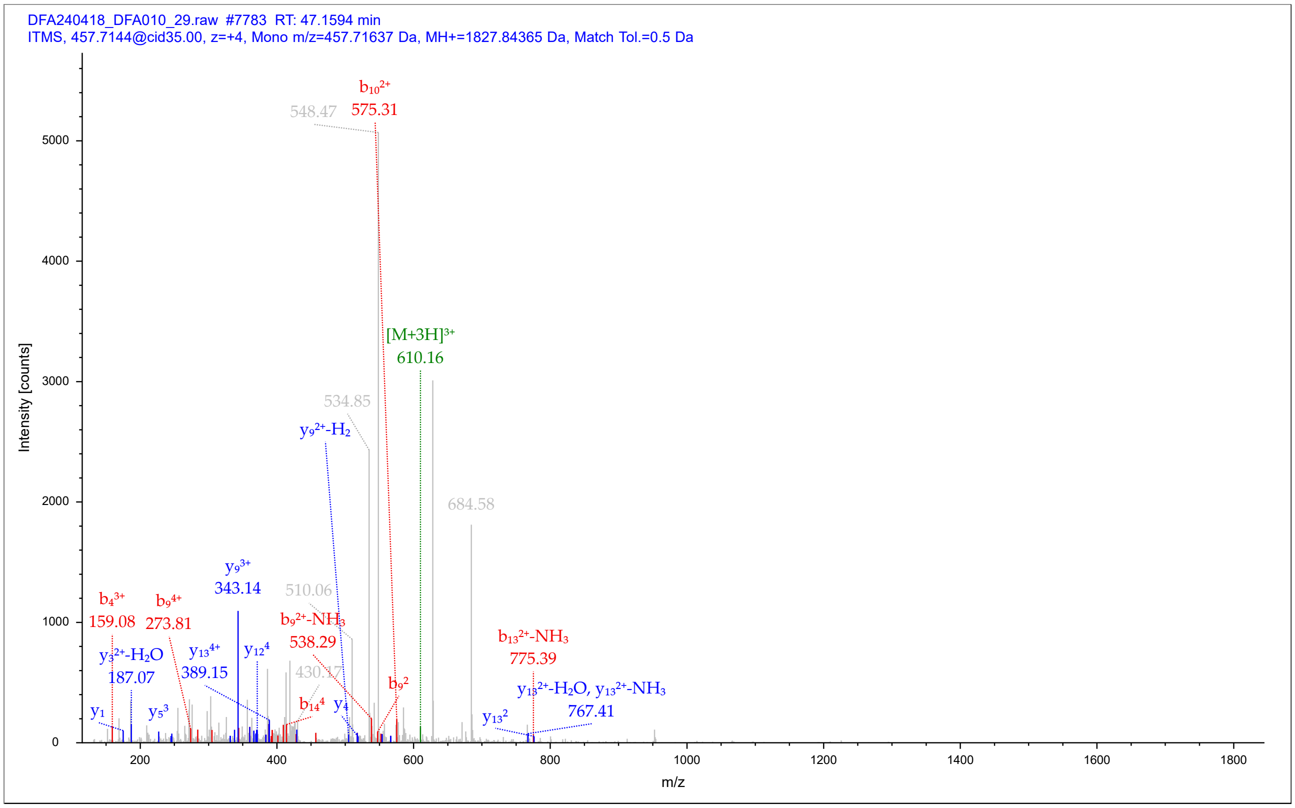

#1	b⁺	b²⁺	b³⁺	b⁴⁺	Seq.	y⁺	y²⁺	y³⁺	y⁴⁺	#2
1	114.09134	57.54931	38.70196	29.27829	L					15
2	277.15467	139.08097	93.05641	70.04412	Y	1714.76019	857.88373	572.25825	429.44551	14
3	348.19178	174.59953	116.73545	87.80340	A	1551.69686	776.35207	517.90381	388.67967	13
4	476.25036	238.62882	159.42164	119.81805	Q	1480.65975	740.83351	494.22477	370.92040	12
5	704.37260	352.68994	235.46238	176.84861	R-Carboxy ethyl	1352.60117	676.80422	451.53858	338.90575	11
6	801.42536	401.21632	267.81331	201.11180	P	1124.47893	562.74310	375.49783	281.87519	10
7	872.46248	436.73488	291.49234	218.87108	A	1027.42617	514.21672	343.14691	257.61200	9
8	929.48394	465.24561	310.49950	233.12644	G	956.38906	478.69817	319.46787	239.85272	8
9	1092.54727	546.77727	364.85394	273.89228	F-Oxidation	899.36759	450.18743	300.46072	225.59736	7
10	1149.56873	575.28801	383.86110	288.14764	G	736.30426	368.65577	246.10627	184.83152	6
11	1309.59938	655.30333	437.20465	328.15530	C-Carbamido methyl	679.28280	340.14504	227.09912	170.57616	5
12	1437.65796	719.33262	479.89084	360.16995	Q	519.25215	260.12971	173.75557	130.56850	4
13	1566.70055	783.85391	522.90504	392.43060	E	391.19357	196.10043	131.06938	98.55385	3
14	1653.73258	827.36993	551.91571	414.18860	S	262.15098	131.57913	88.05518	66.29320	2
15					R	175.11895	88.06311	59.04450	44.53520	1

Sequence: SDVVYTDWK, W8-Trp->Kynurenin (3.99492 Da)
Charge: +2, Monoisotopic m/z: 558.76422 Da (+0.19 mmu/+0.34 ppm), MH+: 1116.52117 Da, RT: 49.5475 min,
Identified with: Sequest HT (v1.17); XCorr:0.62, Percolator q-Value:5.9e-3, Percolator PEP:3.5e-2,
Fragment match tolerance used for search: 0.02 Da
Fragments used for search: -H_2O; y; -NH_3; y; b; b; -H_2O; y

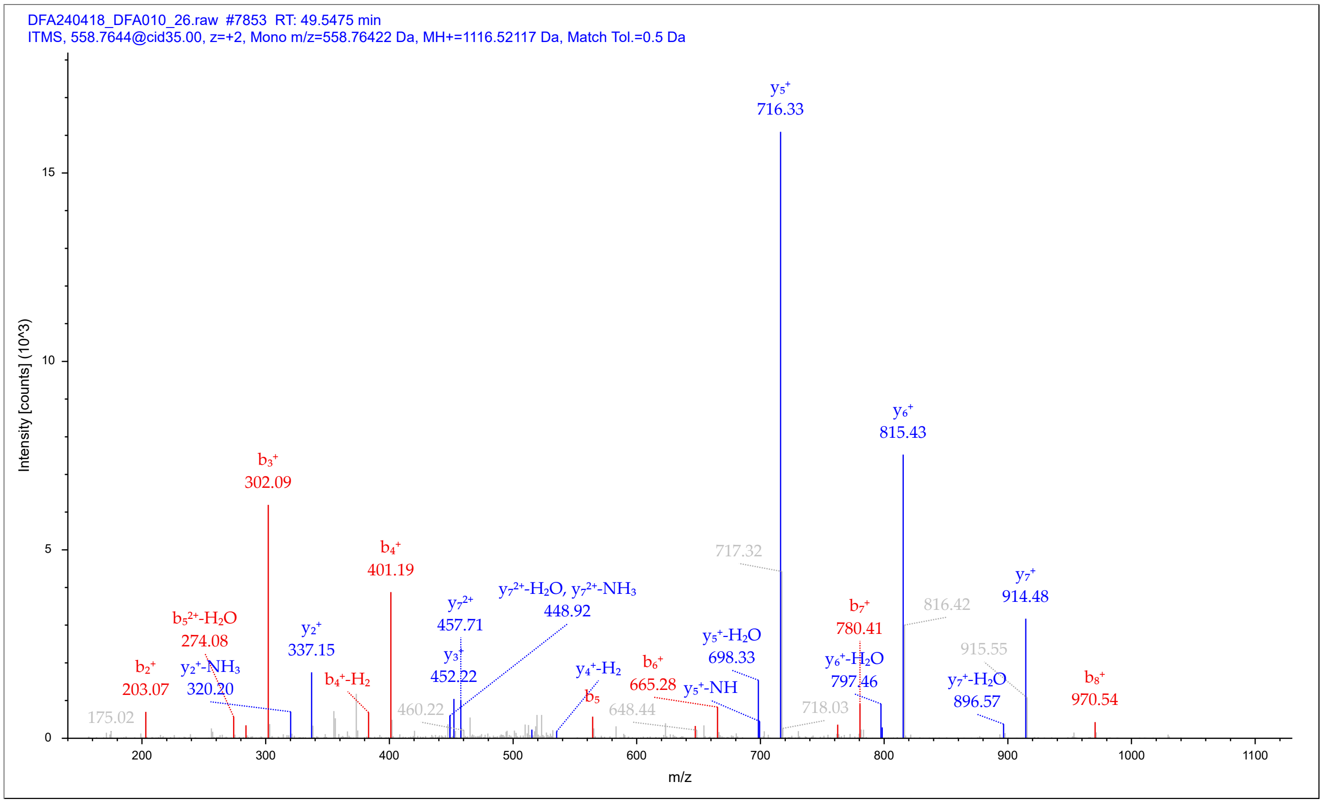

#1	b+	b2+	Seq.	y+	y2+	#2
1	88.03930	44.52329	S			9
2	203.06625	102.03676	D	1029.48875	515.24801	8
3	302.13466	151.57097	V	914.46181	457.73454	7
4	401.20308	201.10518	V	815.39340	408.20034	6
5	564.26640	282.63684	Y	716.32498	358.66613	5
6	665.31408	333.16068	T	553.26165	277.13447	4
7	780.34103	390.67415	D	452.21398	226.61063	3
8	970.41525	485.71127	W-Trp->Kynu-renin	337.18703	169.09715	2
9			K	147.11280	74.06004	1

Sequence: VEAPFIPKFR, F5-Dioxidation (31.98983 Da), F9-Dioxidation (31.98983 Da), R10-Delta:H(2)C(3)O(1) (54.01057 Da)
Charge: +3, Monoisotopic m/z: 441.23135 Da (+0.28 mmu/+0.63 ppm), MH+: 1321.67951 Da, RT: 52.8261 min,
Identified with: Sequest HT (v1.17); XCorr:0.60, Percolator q-Value:2.2e-3, Percolator PEP:1.3e-2,
Fragment match tolerance used for search: 0.02 Da
Fragments used for search: -H_2O; y; -NH_3; y; b; b; -H_2O; b; -NH_3; y

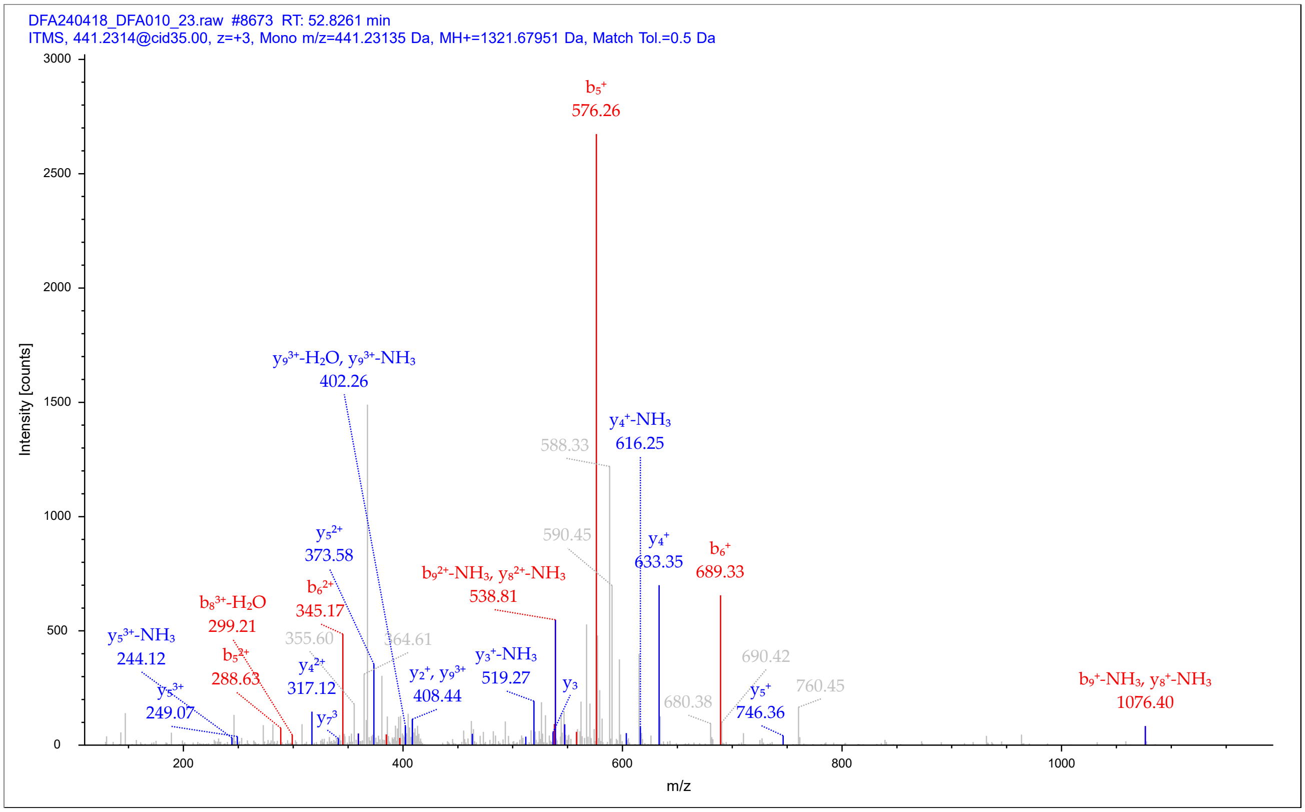

#1	b⁺	b²⁺	b³⁺	Seq.	y⁺	y²⁺	y³⁺	#2
1	100.07569	50.54148	34.03008	V				10
2	229.11828	115.06278	77.04428	E	1222.61026	611.80877	408.20827	9
3	300.15540	150.58134	100.72332	A	1093.56767	547.28747	365.19407	8
4	397.20816	199.10772	133.07424	P	1022.53056	511.76892	341.51504	7
5	576.26640	288.63684	192.76032	F-Dioxidation	925.47779	463.24254	309.16412	6
6	689.35047	345.17887	230.45501	I	746.41955	373.71341	249.47803	5
7	786.40323	393.70525	262.80593	P	633.33549	317.17138	211.78335	4
8	914.49819	457.75274	305.50425	K	536.28272	268.64500	179.43243	3
9	1093.55644	547.28186	365.19033	F-Dioxidation	408.18776	204.59752	136.73410	2
10				MGH	229.12952	115.06840	77.04802	1

Sequence: KVPQVSTPTLVEVSR, K1-Ethanalyl (42.01830 Da)
Charge: +3, Monoisotopic m/z: 561.32501 Da (+1.48 mmu/+2.64 ppm), MH+: 1681.96048 Da, RT: 55.2073 min,
Identified with: Sequest HT (v1.17); XCorr:1.33, Percolator q-Value:4.0e-3, Percolator PEP:2.2e-2,
Fragment match tolerance used for search: 0.02 Da
Fragments used for search: -H_2O; y; -NH_3; y; b; b; -H_2O; b; -NH_3; y

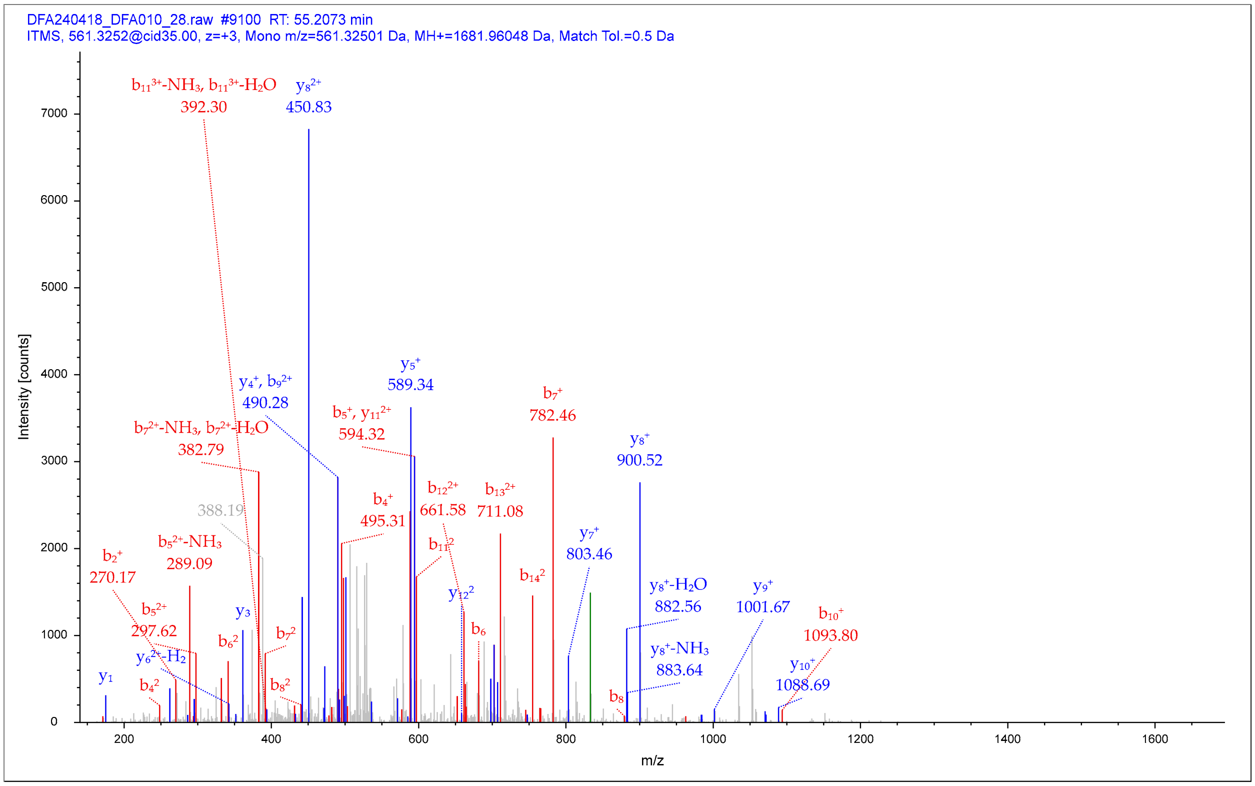

#1	b⁺	b²⁺	b³⁺	Seq.	y⁺	y²⁺	y³⁺	#2
1	171.12054	86.06391	57.71170	K-Ethanalyl				15
2	270.18895	135.59812	90.73450	V	1511.84278	756.42503	504.61911	14
3	367.24172	184.12450	123.08542	P	1412.77437	706.89082	471.59631	13
4	495.30029	248.15379	165.77162	Q	1315.72161	658.36444	439.24539	12
5	594.36871	297.68799	198.79442	V	1187.66303	594.33515	396.55919	11
6	681.40074	341.20401	227.80510	S	1088.59461	544.80095	363.53639	10
7	782.44842	391.72785	261.48766	T	1001.56259	501.28493	334.52571	9
8	879.50118	440.25423	293.83858	P	900.51491	450.76109	300.84315	8
9	980.54886	490.77807	327.52114	T	803.46214	402.23471	268.49223	7
10	1093.63292	547.32010	365.21582	L	702.41447	351.71087	234.80967	6
11	1192.70134	596.85431	398.23863	V	589.33040	295.16884	197.11498	5
12	1321.74393	661.37560	441.25283	E	490.26199	245.63463	164.09218	4
13	1420.81234	710.90981	474.27563	V	361.21939	181.11334	121.07798	3
14	1507.84437	754.42582	503.28631	S	262.15098	131.57913	88.05518	2
15				R	175.11895	88.06311	59.04450	1

Sequence: FNWYVDGVEVHNAK, W3-Trp->Kynurenin (3.99492 Da)
Charge: +2, Monoisotopic m/z: 841.40161 Da (-0.48 mmu/-0.56 ppm), MH+: 1681.79595 Da, RT: 62.6532 min,
Identified with: Sequest HT (v1.17); XCorr:0.96, Percolator q-Value:0.0e0, Percolator PEP:1.2e-2,
Fragment match tolerance used for search: 0.02 Da
Fragments used for search: $-H_2O$; y; $-NH_3$; y; b; b; $-H_2O$; b; $-NH_3$; y

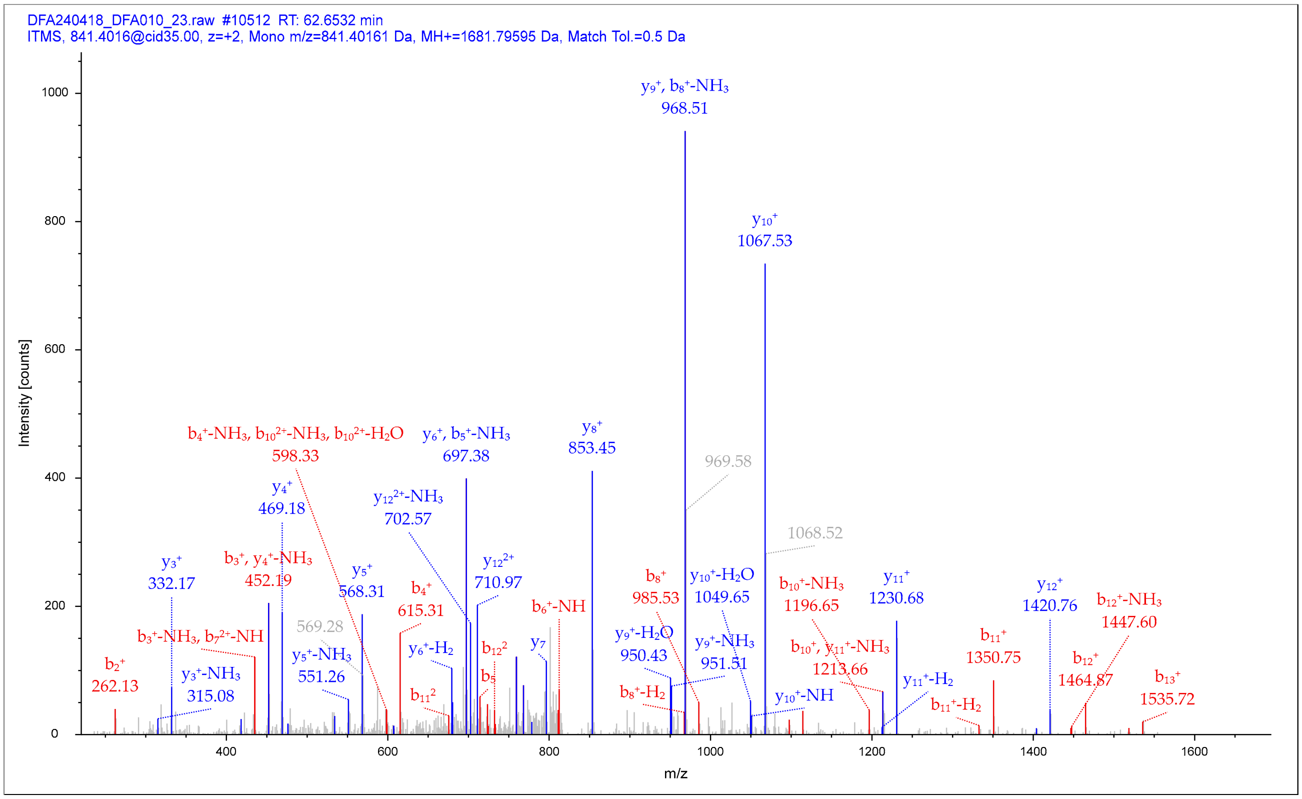

#1	b+	b2+	Seq.	y+	y2+	#2
1	148.07569	74.54148	F			14
2	262.11862	131.56295	N	1534.72848	767.86788	13
3	452.19285	226.60006	W-Trp->Kynu-renin	1420.68555	710.84642	12
4	615.25617	308.13173	Y	1230.61133	615.80930	11
5	714.32459	357.66593	V	1067.54800	534.27764	10
6	829.35153	415.17940	D	968.47958	484.74343	9
7	886.37299	443.69014	G	853.45264	427.22996	8
8	985.44141	493.22434	V	796.43118	398.71923	7
9	1114.48400	557.74564	E	697.36276	349.18502	6
10	1213.55242	607.27985	V	568.32017	284.66372	5
11	1350.61133	675.80930	H	469.25176	235.12952	4
12	1464.65425	732.83077	N	332.19285	166.60006	3
13	1535.69137	768.34932	A	218.14992	109.57860	2
14			K	147.11280	74.06004	1

Sequence: WWFDWGWCNIR, C8-Carbamidomethyl (57.02146 Da), W5-Trp->Kynurenin (3.99492 Da), R11-Ethanalyl (42.01830 Da)
Charge: +2, Monoisotopic m/z: 836.36536 Da (-0.22 mmu/-0.26 ppm), MH+: 1671.72344 Da, RT: 64.0627 min, Identified with: Sequest HT (v1.17); XCorr:0.69, Percolator q-Value:3.6e-3, Percolator PEP:1.8e-2,
Fragment match tolerance used for search: 0.02 Da
Fragments used for search: -H_2O; y; -NH_3; y; b; b; -H_2O; b; -NH_3; y

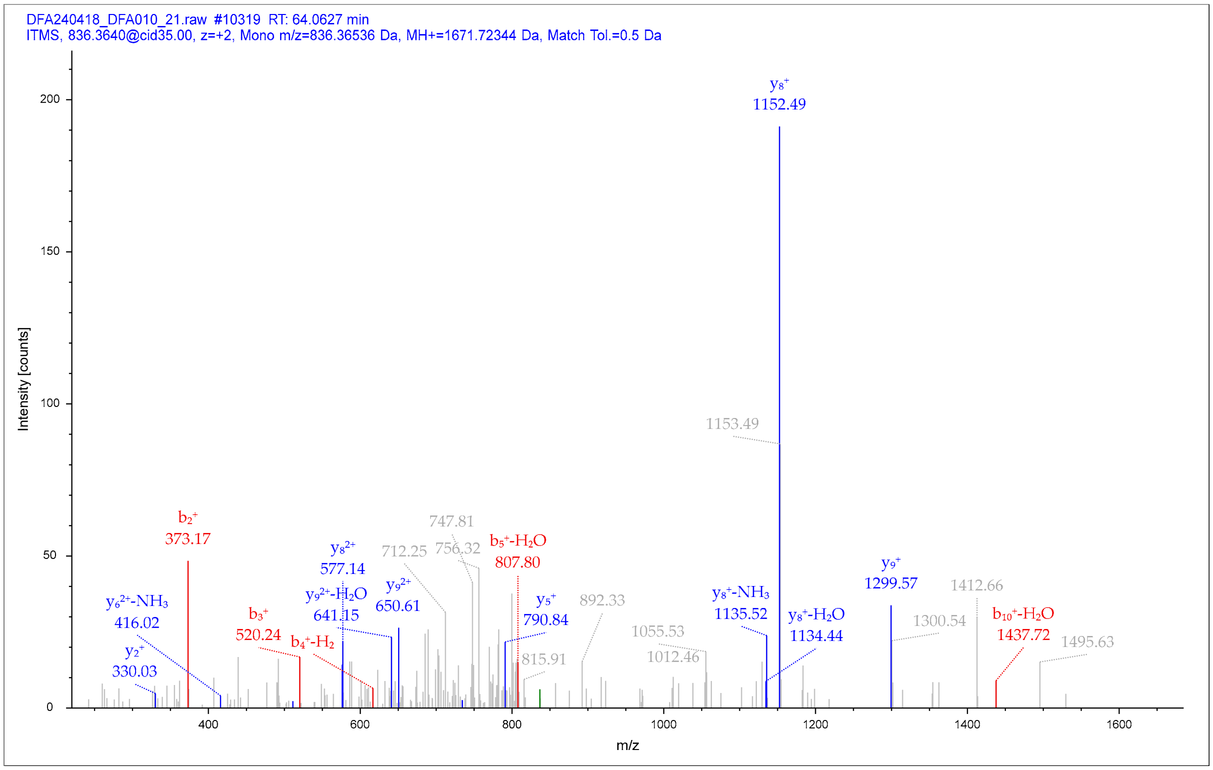

#1	b⁺	b²⁺	Seq.	y⁺	y²⁺	#2
1	187.08659	94.04693	W			11
2	373.16590	187.08659	W	1485.64457	743.32592	10
3	520.23432	260.62080	F	1299.56525	650.28626	9
4	635.26126	318.13427	D	1152.49684	576.75206	8
5	825.33549	413.17138	W-Trp-> Kynurenin	1037.46990	519.23859	7
6	882.35695	441.68211	G	847.39567	424.20147	6
7	1068.43626	534.72177	W	790.37420	395.69074	5
8	1228.46691	614.73709	C-Carbamido methyl	604.29489	302.65108	4
9	1342.50984	671.75856	N	444.26424	222.63576	3
10	1455.59390	728.30059	I	330.22132	165.61430	2
11			R-Ethanalyl	217.13725	109.07226	1

17

Sequence: DASGVTFTWTPSSGK, F7-Oxidation (15.99492 Da), W9-Trp->Kynurenin (3.99492 Da), K15-Formyl (27.99492 Da)
Charge: +2, Monoisotopic m/z: 794.85962 Da (-0.3 mmu/-0.38 ppm), MH+: 1588.71196 Da, RT: 71.8592 min,
Identified with: Sequest HT (v1.17); XCorr:0.80, Percolator q-Value:2.1e-2, Percolator PEP:8.8e-2,
Fragment match tolerance used for search: 0.02 Da
Fragments used for search: -H_2O; y; b; b; -H_2O; y

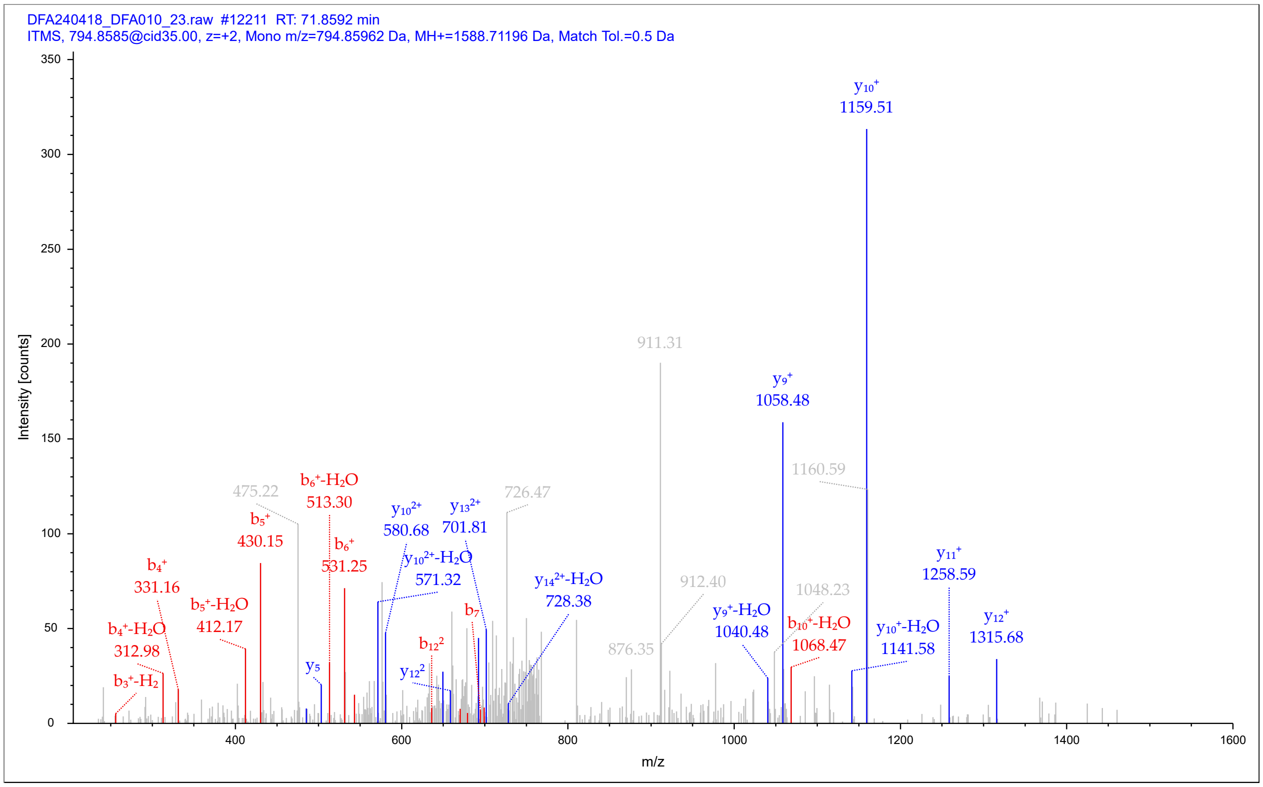

#1	b+	b2+	Seq.	y+	y2+	#2
1	116.03422	58.52075	D			15
2	187.07133	94.03930	A	1473.68562	737.34645	14
3	274.10336	137.55532	S	1402.64850	701.82789	13
4	331.12483	166.06605	G	1315.61647	658.31187	12
5	430.19324	215.60026	V	1258.59501	629.80114	11
6	531.24092	266.12410	T	1159.52660	580.26694	10
7	694.30425	347.65576	F-Oxidation	1058.47892	529.74310	9
8	795.35192	398.17960	T	895.41559	448.21143	8
9	985.42615	493.21671	W-Trp->Kynurenin	794.36791	397.68759	7
10	1086.47383	543.74055	T	604.29368	302.65048	6
11	1183.52660	592.26694	P	503.24600	252.12664	5
12	1270.55862	635.78295	S	406.19324	203.60026	4
13	1357.59065	679.29896	S	319.16121	160.08424	3
14	1414.61212	707.80970	G	232.12918	116.56823	2
15			K-Formyl	175.10772	88.05750	1

43

Sequence: GNPFLKGPPPAQPLAQR, K6-Carboxyethyl (72.02113 Da), R17-Carboxyethyl (72.02113 Da)
Charge: +4, Monoisotopic m/z: 483.76480 Da (+0.9 mmu/+1.86 ppm), MH+: 1932.03737 Da, RT: 70.8826 min,
Identified with: Sequest HT (v1.17); XCorr:0.60, Percolator q-Value:4.2e-3, Percolator PEP:3.4e-2, ptmRS: Best Site Probabilities:K6(Carboxyethyl): 100,
Fragment match tolerance used for search: 0.02 Da
Fragments used for search: -NH_3; y; b; b; -NH_3; y

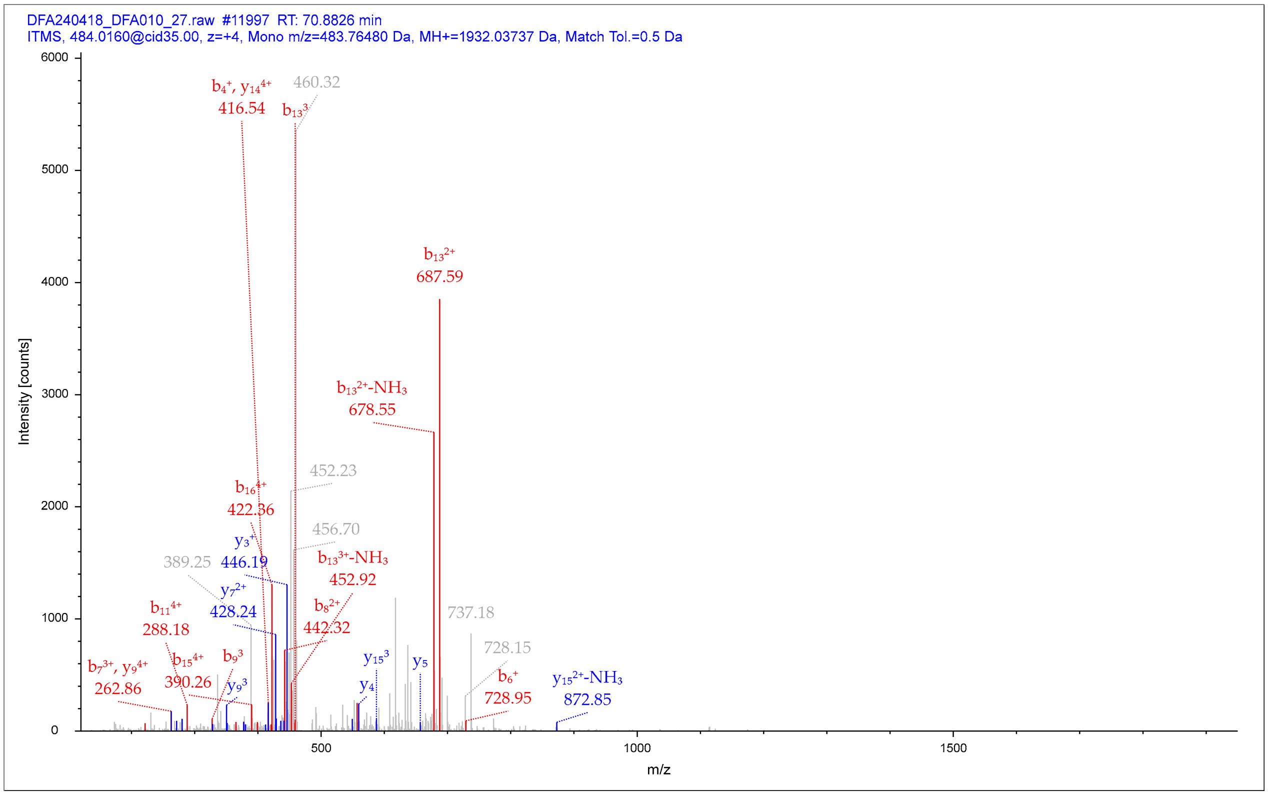

#1	b⁺	b²⁺	b³⁺	b⁴⁺	Seq.	y⁺	y²⁺	y³⁺	y⁴⁺	#2
1	58.02874	29.51801	20.01443	15.26264	G					17
2	172.07167	86.53947	58.02874	43.77337	N	1875.01231	938.00979	625.67562	469.50853	16
3	269.12443	135.06585	90.37966	68.03657	P	1760.96938	880.98833	587.66131	440.99780	15
4	416.19285	208.60006	139.40247	104.80367	F	1663.91662	832.46195	555.31039	416.73461	14
5	529.27691	265.14209	177.09715	133.07468	L	1516.84820	758.92774	506.28759	379.96751	13
6	729.39300	365.20014	243.80252	183.10371	K-Carboxy ethyl	1403.76414	702.38571	468.59290	351.69649	12
7	786.41446	393.71087	262.80967	197.35907	G	1203.64805	602.32766	401.88753	301.66747	11
8	883.46723	442.23725	295.16059	221.62226	P	1146.62658	573.81693	382.88038	287.41210	10
9	980.51999	490.76363	327.51152	245.88546	P	1049.57382	525.29055	350.52946	263.14891	9
10	1077.57276	539.29002	359.86244	270.14865	P	952.52106	476.76417	318.17854	238.88572	8
11	1148.60987	574.80857	383.54147	287.90793	A	855.46829	428.23778	285.82761	214.62253	7
12	1276.66845	638.83786	426.22767	319.92257	Q	784.43118	392.71923	262.14858	196.86325	6
13	1373.72121	687.36424	458.57859	344.18576	P	656.37260	328.68994	219.46238	164.84861	5
14	1486.80528	743.90628	496.27328	372.45678	L	559.31984	280.16356	187.11146	140.58542	4
15	1557.84239	779.42483	519.95231	390.21605	A	446.23577	223.62152	149.41678	112.31440	3
16	1685.90097	843.45412	562.63851	422.23070	Q	375.19866	188.10297	125.73774	94.55512	2
17					R-Carboxy ethyl	247.14008	124.07368	83.05154	62.54048	1

Sequence: TLKPWLGDGLLLSVGDK, W5-Dioxidation (31.98983 Da), K17-Glyoxal-imine (39.99554 Da)
Charge: +2, Monoisotopic m/z: 942.50653 Da (-3.07 mmu/-3.26 ppm), MH+: 1884.00578 Da, RT: 75.3946 min,
Identified with: Sequest HT (v1.17); XCorr:0.71, Percolator q-Value:5.5e-3, Percolator PEP:2.8e-2,
Fragment match tolerance used for search: 0.02 Da
Fragments used for search: $-H_2O$; y; $-NH_3$; y; b; b; $-H_2O$; b; $-NH_3$; y

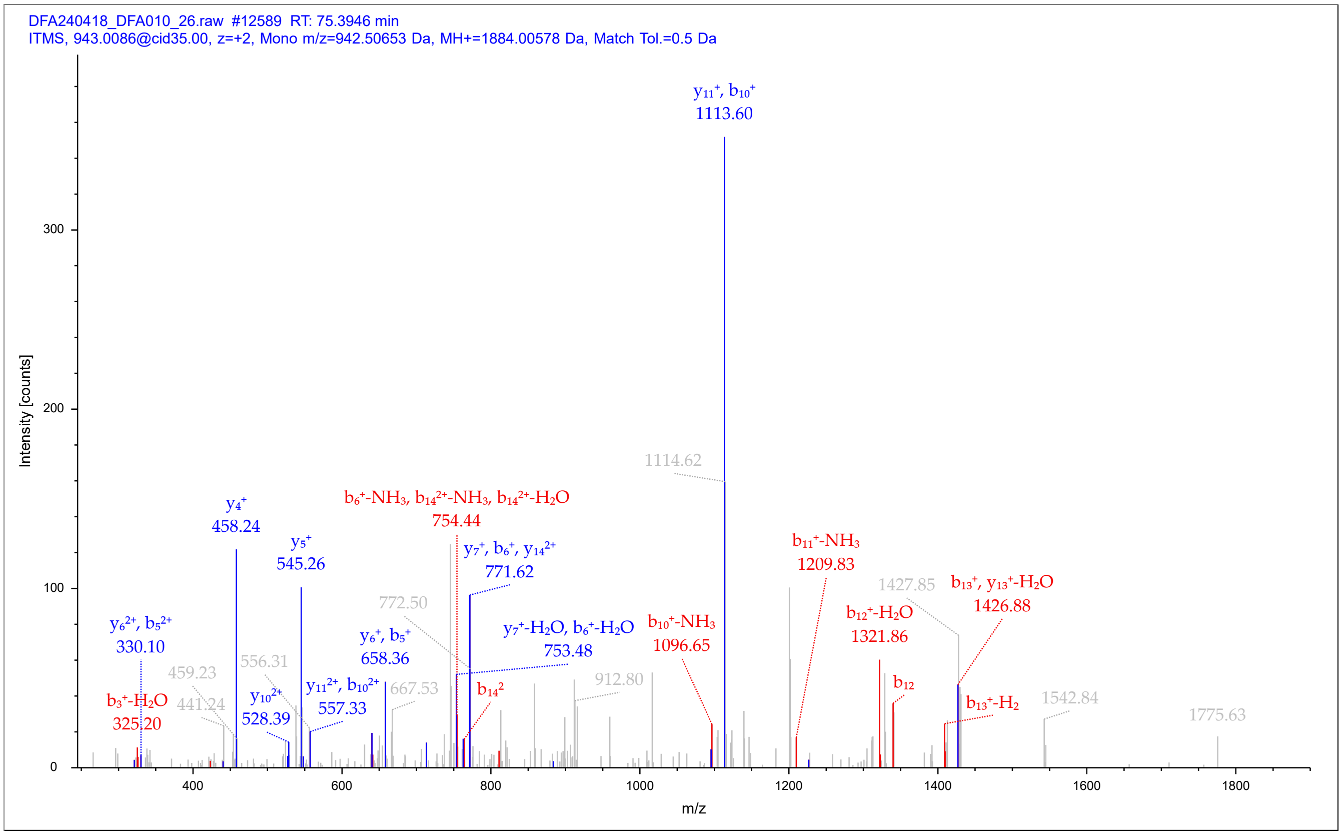

187

Sequence: LLDNWDSVTSTFSK, W5-Trp->Kynurenin (3.99492 Da)
Charge: +2,   Monoisotopic m/z: 808.89368 Da (-0.08 mmu/-0.1 ppm),   MH+: 1616.78008 Da,   RT: 81.1475 min,
Identified with: Sequest HT (v1.17); XCorr:0.57, Percolator q-Value:7.2e-3, Percolator PEP:3.8e-2,
Fragment match tolerance used for search: 0.02 Da
Fragments used for search: -H_2O; y; -NH_3; y; b; b; -H_2O; b; -NH_3; y

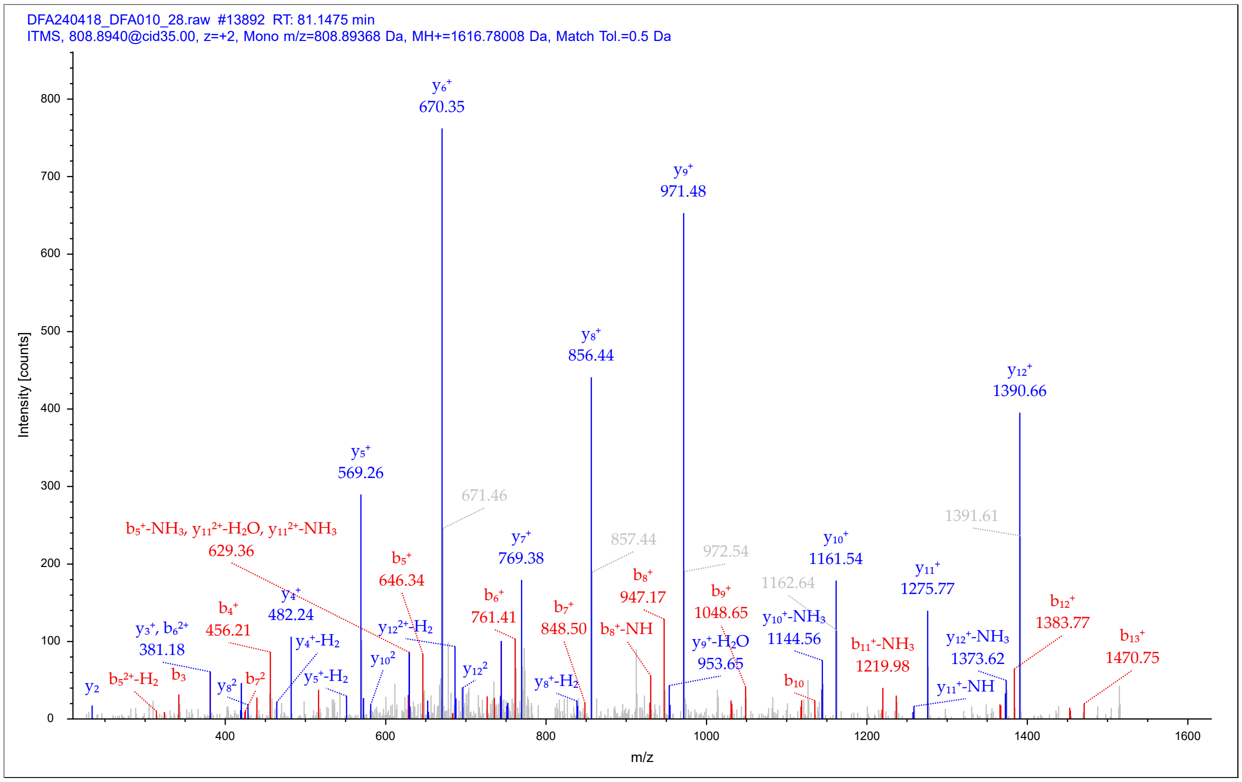

#1	b⁺	b²⁺	Seq.	y⁺	y²⁺	#2
1	114.09134	57.54931	L			14
2	227.17540	114.09134	L	1503.69618	752.35173	13
3	342.20235	171.60481	D	1390.61212	695.80970	12
4	456.24527	228.62628	N	1275.58517	638.29622	11
5	646.31950	323.66339	W-Trp-> Kynurenin	1161.54224	581.27476	10
6	761.34645	381.17686	D	971.46802	486.23765	9
7	848.37847	424.69288	S	856.44107	428.72418	8
8	947.44689	474.22708	V	769.40905	385.20816	7
9	1048.49457	524.75092	T	670.34063	335.67395	6
10	1135.52659	568.26694	S	569.29295	285.15011	5
11	1236.57427	618.79077	T	482.26092	241.63410	4
12	1383.64269	692.32498	F	381.21325	191.11026	3
13	1470.67472	735.84100	S	234.14483	117.57605	2
14			K	147.11280	74.06004	1

21

Sequence: DLKEYDKDNIPVTVMQK, K17-Acetyl (42.01057 Da), M15-Trioxidation (47.98474 Da)
Charge: +4, Monoisotopic m/z: 532.26288 Da (-0.62 mmu/-1.17 ppm), MH+: 2126.02968 Da, RT: 48.6539 min,
Identified with: Sequest HT (v1.17); XCorr:0.86, Percolator q-Value:2.0e-3, Percolator PEP:1.5e-2, ptmRS: Best Site Probabilities:K17(Acetyl): 100,
Fragment match tolerance used for search: 0.02 Da
Fragments used for search: -H_2O; y; -NH_3; y; b; b; -H_2O; b; -NH_3; y

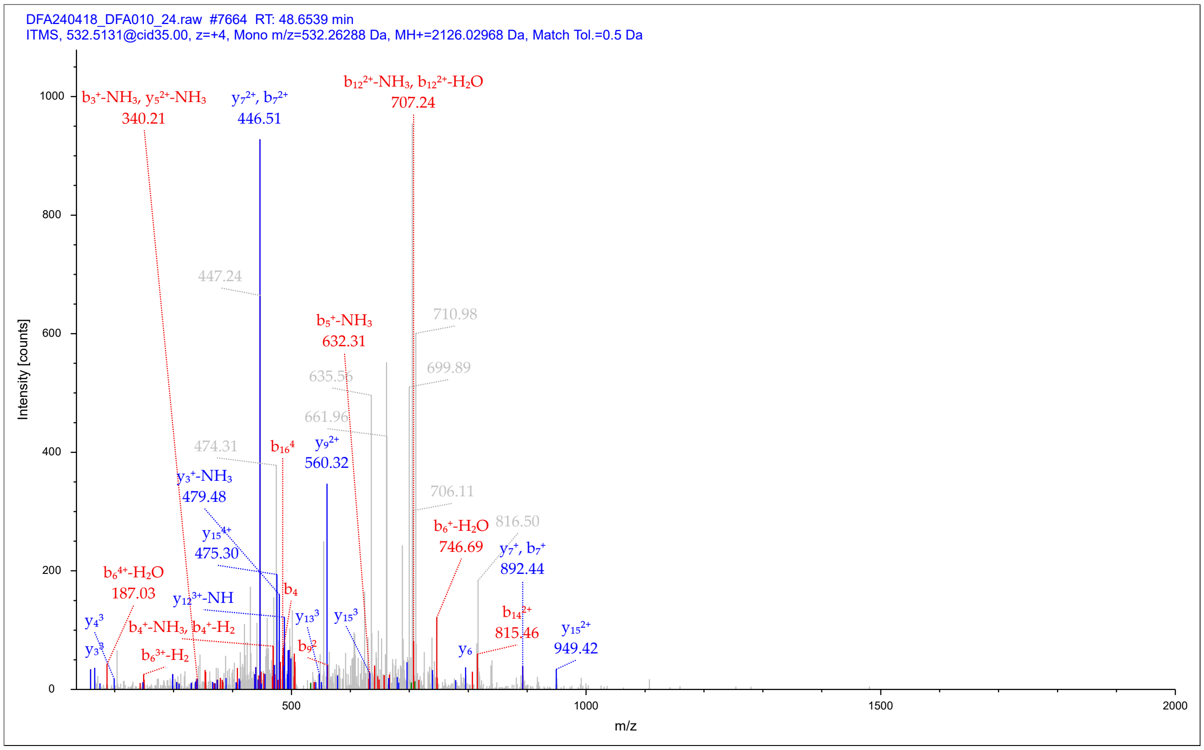

#1	b+	b2+	b3+	b4+	Seq.	y+	y2+	y3+	y4+	#2
1	116.03422	58.52075	39.34959	29.76401	D					17
2	229.11828	115.06278	77.04428	58.03503	L	2011.00523	1006.00626	671.00660	503.50677	16
3	357.21325	179.11026	119.74260	90.05877	K	1897.92117	949.46422	633.31191	475.23575	15
4	486.25584	243.63156	162.75680	122.31942	E	1769.82621	885.41674	590.61359	443.21201	14
5	649.31917	325.16322	217.11124	163.08525	Y	1640.78361	820.89545	547.59939	410.95136	13
6	764.34611	382.67669	255.45355	191.84199	D	1477.72029	739.36378	493.24495	370.18553	12
7	892.44107	446.72418	298.15188	223.86573	K	1362.69334	681.85031	454.90263	341.42879	11
8	1007.46802	504.23765	336.49419	252.62246	D	1234.59838	617.80283	412.20431	309.40505	10
9	1121.51094	561.25911	374.50850	281.13319	N	1119.57144	560.28936	373.86200	280.64832	9
10	1234.59501	617.80114	412.20319	309.40421	I	1005.52851	503.26789	335.84769	252.13758	8
11	1331.64777	666.32752	444.55411	333.66740	P	892.44445	446.72586	298.15300	223.86657	7
12	1430.71619	715.86173	477.57691	358.43450	V	795.39168	398.19948	265.80208	199.60338	6
13	1531.76386	766.38557	511.25947	383.69642	T	696.32327	348.66527	232.77927	174.83627	5
14	1630.83228	815.91978	544.28228	408.46353	V	595.27559	298.14143	199.09671	149.57435	4
15	1809.85751	905.43239	603.95735	453.21983	M-Trioxidation	496.20718	248.60723	166.07391	124.80725	3
16	1937.91608	969.46168	646.64355	485.23448	Q	317.18195	159.09461	106.39883	80.05094	2
17					K-Acetyl	189.12337	95.06532	63.71264	48.03630	1

Sequence: KIEKEAAQLQGR, R12-Carboxymethyl (58.00548 Da), K4-C4-amide (118.02724 Da)
Charge: +2, Monoisotopic m/z: 773.90112 Da (-6.4 mmu/-8.27 ppm), MH+: 1546.79497 Da, RT: 22.9129 min,
Identified with: Sequest HT (v1.17); XCorr:1.58, Percolator q-Value:3.4e-3, Percolator PEP:6.9e-3,
Fragment match tolerance used for search: 0.02 Da
Fragments used for search: -H_2O; y; -NH_3; y; b; b; -H_2O; b; -NH_3; y

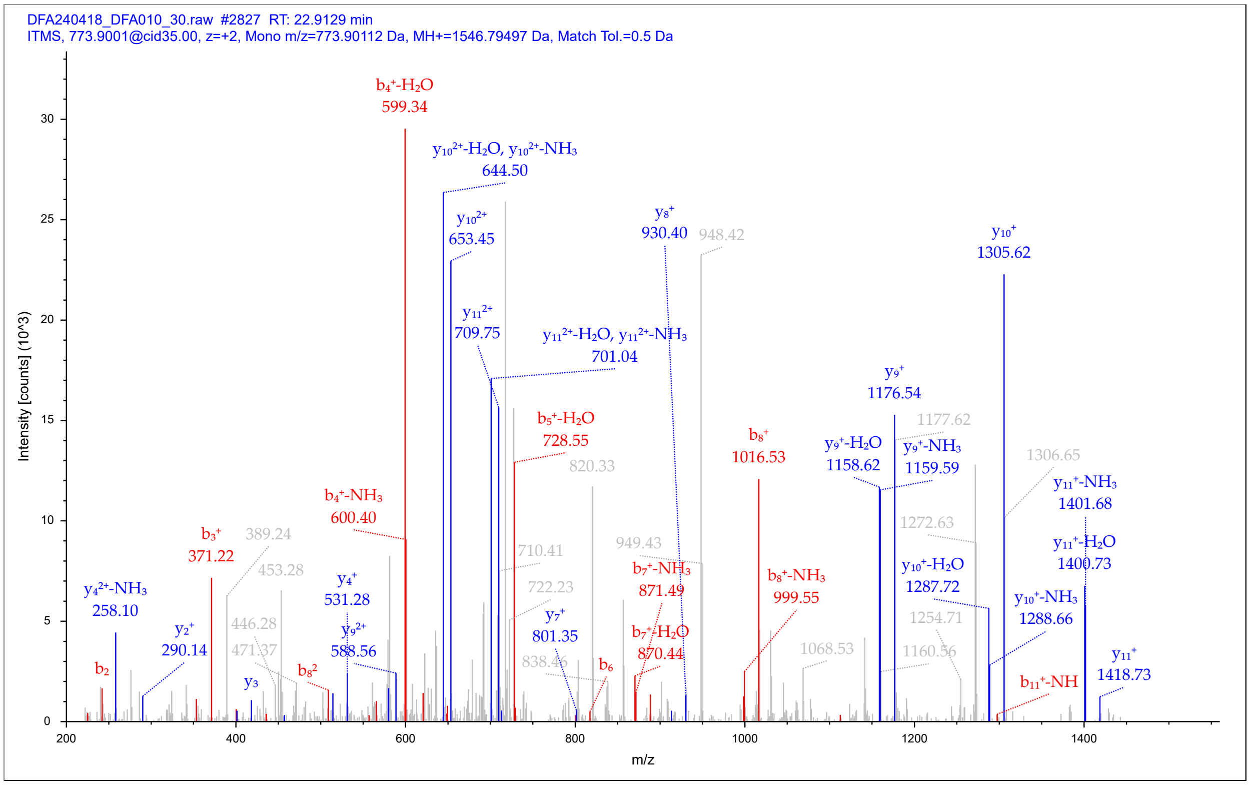

#1	b⁺	b²⁺	Seq.	y⁺	y²⁺	#2
1	129.10224	65.05476	K			12
2	242.18630	121.59679	I	1418.71279	709.86004	11
3	371.22890	186.11809	E	1305.62873	653.31800	10
4	617.35110	309.17919	K-C4-amide	1176.58614	588.79671	9
5	746.39369	373.70048	E	930.46393	465.73561	8
6	817.43081	409.21904	A	801.42134	401.21431	7
7	888.46792	444.73760	A	730.38423	365.69575	6
8	1016.52650	508.76689	Q	659.34711	330.17720	5
9	1129.61056	565.30892	L	531.28854	266.14791	4
10	1257.66914	629.33821	Q	418.20447	209.60587	3
11	1314.69060	657.84894	G	290.14589	145.57659	2
12			R-Carboxymethyl	233.12443	117.06585	1

Sequence: YICENQDSISSK, C3-Carbamidomethyl (57.02146 Da), K12-Ethanalyl (42.01830 Da)
Charge: +2, Monoisotopic m/z: 743.33362 Da (-0.19 mmu/-0.25 ppm), MH+: 1485.65996 Da, RT: 35.2237 min,
Identified with: Sequest HT (v1.17); XCorr:1.06, Percolator q-Value:1.9e-3, Percolator PEP:7.7e-3,
Fragment match tolerance used for search: 0.02 Da
Fragments used for search: -H_2O; y; -NH_3; y; b; b; -H_2O; b; -NH_3; y

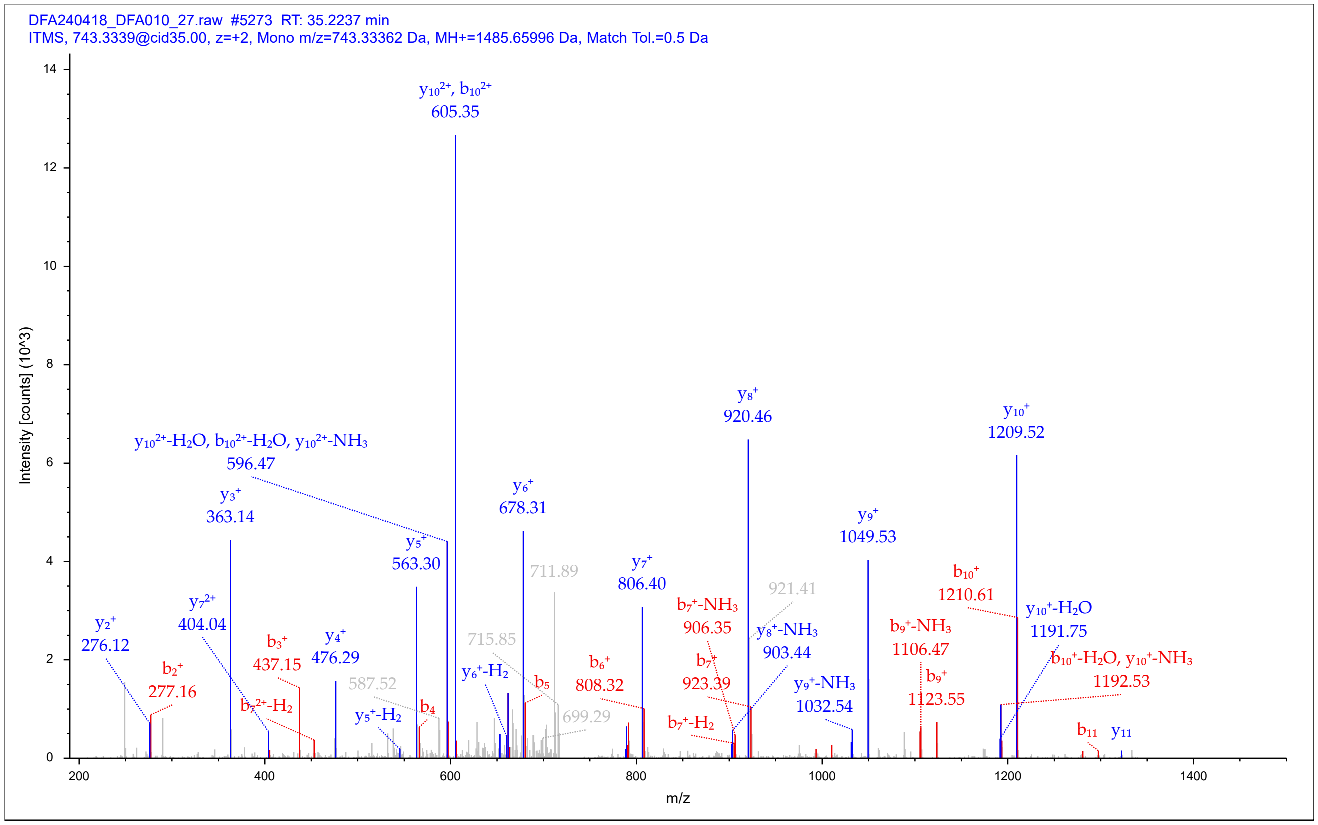

#1	b+	b2+	Seq.	y+	y2+	#2
1	164.07061	82.53894	Y			12
2	277.15467	139.08097	I	1322.59701	661.80214	11
3	437.18532	219.09630	C-Carbamido methyl	1209.51294	605.26011	10
4	566.22791	283.61759	E	1049.48229	525.24479	9
5	680.27084	340.63906	N	920.43970	460.72349	8
6	808.32942	404.66835	Q	806.39677	403.70203	7
7	923.35636	462.18182	D	678.33820	339.67274	6
8	1010.38839	505.69783	S	563.31125	282.15926	5
9	1123.47245	562.23986	I	476.27922	238.64325	4
10	1210.50448	605.75588	S	363.19516	182.10122	3
11	1297.53651	649.27189	S	276.16313	138.58520	2
12			K-Ethanalyl	189.13110	95.06919	1

Sequence: ETYGEMADCCAK, C9-Carbamidomethyl (57.02146 Da), C10-Carbamidomethyl (57.02146 Da), K12-Ethanalyl (42.01830 Da)
Charge: +2, Monoisotopic m/z: 738.77954 Da (+0.05 mmu/+0.06 ppm), MH+: 1476.55181 Da, RT: 38.6772 min,
Identified with: Sequest HT (v1.17); XCorr:0.70, Percolator q-Value:6.4e-3, Percolator PEP:4.2e-2,
Fragment match tolerance used for search: 0.02 Da
Fragments used for search: -H_2O; y; b; b; -H_2O; y

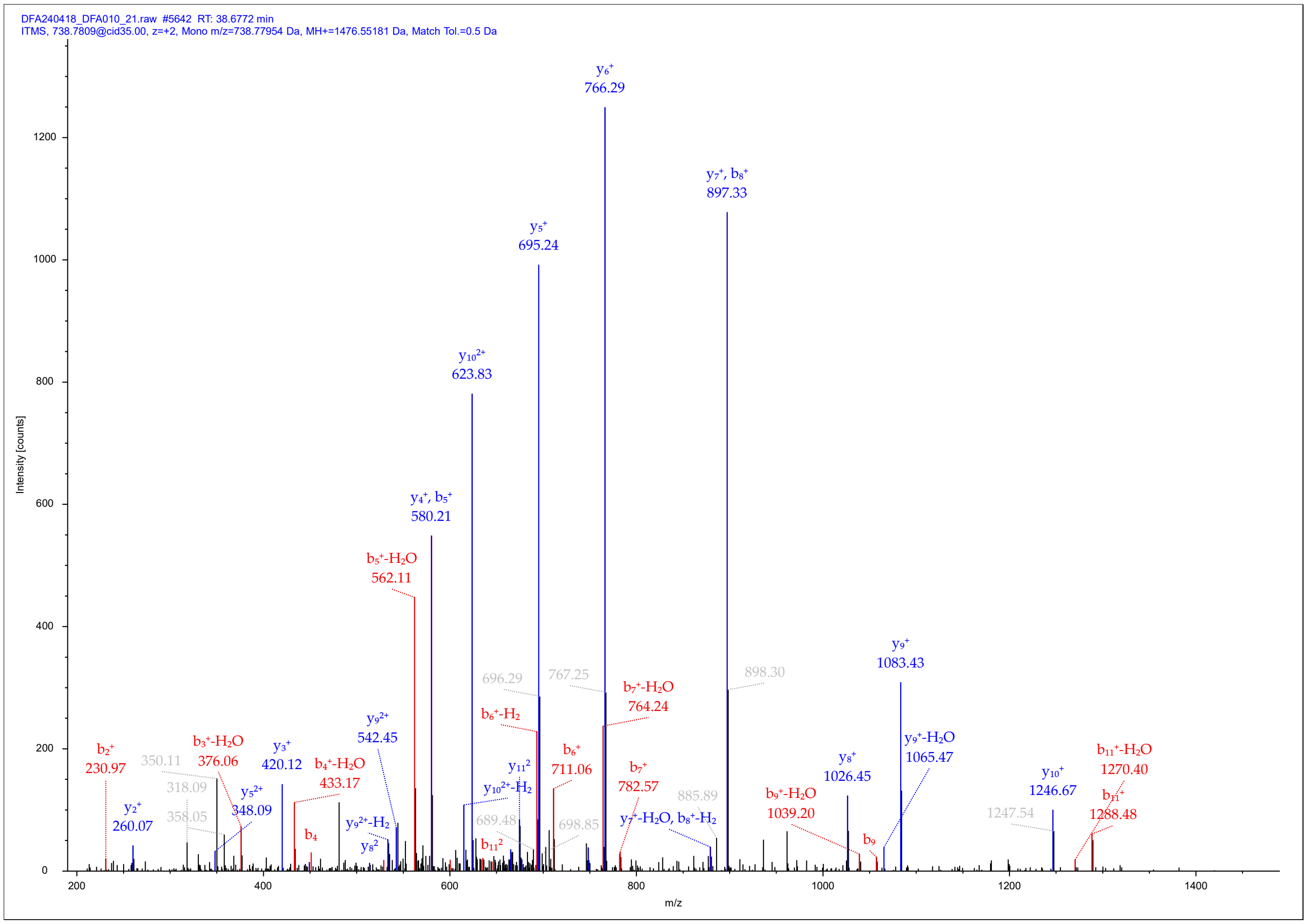

#1	b$^+$	b^{2+}	Seq.	y$^+$	y^{2+}	#2
1	130.04987	65.52857	E			12
2	231.09755	116.05241	T	1347.50912	674.25820	11
3	394.16088	197.58408	Y	1246.46144	623.73436	10
4	451.18234	226.09481	G	1083.39811	542.20269	9
5	580.22493	290.61610	E	1026.37665	513.69196	8
6	711.26542	356.13635	M	897.33406	449.17067	7
7	782.30253	391.65490	A	766.29357	383.65042	6
8	897.32947	449.16838	D	695.25646	348.13187	5
9	1057.36012	529.18370	Carbamidometl	580.22951	290.61840	4
10	1217.39077	609.19902	Carbamidometl	420.19887	210.60307	3
11	1288.42789	644.71758	A	260.16822	130.58775	2
12			K-Ethanalyl	189.13110	95.06919	1

Sequence: TEDTAVYYCAK, C9-Carbamidomethyl (57.02146 Da), Y8-Oxidation (15.99492 Da), K11-Ethanalyl (42.01830 Da)
Charge: +2, Monoisotopic m/z: 689.79980 Da (+0.74 mmu/+1.07 ppm), MH+: 1378.59233 Da, RT: 39.6127 min,
Identified with: Sequest HT (v1.17); XCorr:1.04, Percolator q-Value:5.0e-3, Percolator PEP:3.1e-2,
Fragment match tolerance used for search: 0.02 Da
Fragments used for search: -H_2O; y; b; b; -H_2O; y

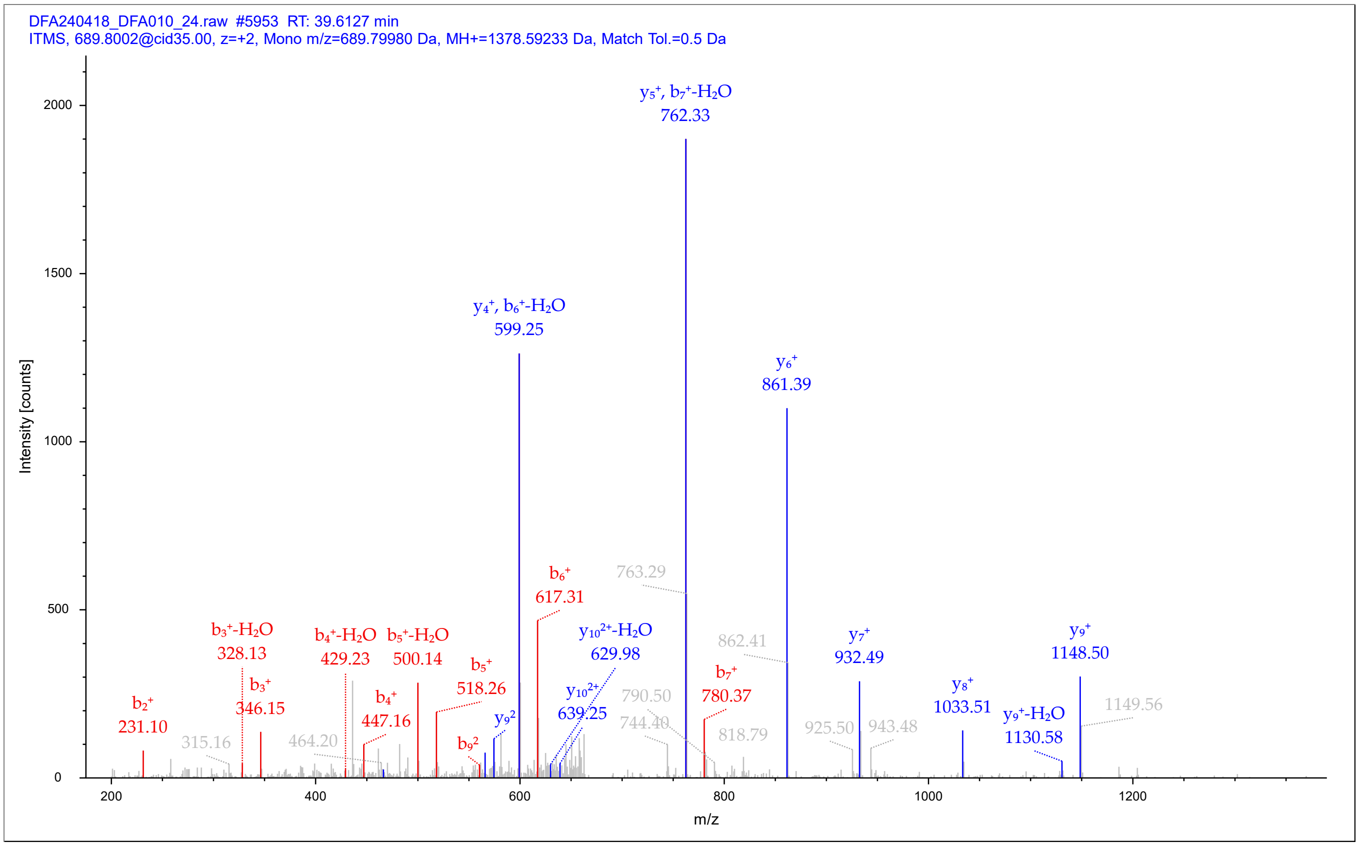

#1	b+	b2+	Seq.	y+	y2+	#2
1	102.05496	51.53112	T			11
2	231.09755	116.05241	E	1277.54318	639.27523	10
3	346.12449	173.56588	D	1148.50059	574.75393	9
4	447.17217	224.08972	T	1033.47364	517.24046	8
5	518.20928	259.60828	A	932.42597	466.71662	7
6	617.27770	309.14249	V	861.38885	431.19806	6
7	780.34103	390.67415	Y	762.32044	381.66386	5
8	959.39927	480.20327	Y-Oxidation	599.25711	300.13219	4
9	1119.42992	560.21860	C-Carbamidomethyl	420.19887	210.60307	3
10	1190.46703	595.73715	A	260.16822	130.58775	2
11			K-Ethanalyl	189.13110	95.06919	1

Sequence: VTSIQDWVQK, W7-Trp->Kynurenin (3.99492 Da), K10-Formyl (27.99492 Da)
Charge: +2, Monoisotopic m/z: 618.31561 Da (-1.35 mmu/-2.18 ppm), MH+: 1235.62395 Da, RT: 53.9392 min,
Identified with: Sequest HT (v1.17); XCorr:0.66, Percolator q-Value:3.5e-2, Percolator PEP:1.2e-1,
Fragment match tolerance used for search: 0.02 Da
Fragments used for search: -H_2O; y; -NH_3; y; b; b; -H_2O; b; -NH_3; y

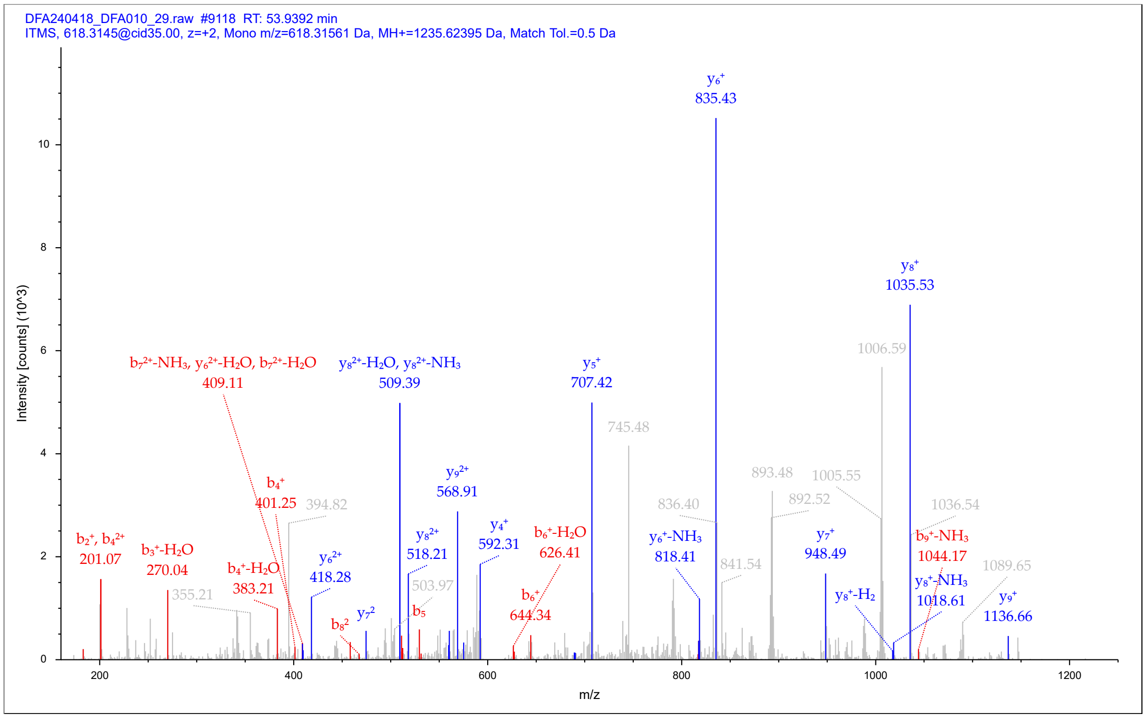

#1	b⁺	b²⁺	Seq.	y⁺	y²⁺	#2
1	100.07569	50.54148	V			10
2	201.12337	101.06532	T	1136.55823	568.78275	9
3	288.15540	144.58134	S	1035.51055	518.25891	8
4	401.23946	201.12337	I	948.47852	474.74290	7
5	529.29804	265.15266	Q	835.39446	418.20087	6
6	644.32498	322.66613	D	707.33588	354.17158	5
7	834.39921	417.70324	W-Trp-> Kynurenin	592.30894	296.65811	4
8	933.46762	467.23745	V	402.23471	201.62099	3
9	1061.52620	531.26674	Q	303.16630	152.08679	2
10			K-Formyl	175.10772	88.05750	1

Sequence: SPWWTPSTSCRPETVFHK, C10-Carbamidomethyl (57.02146 Da), W4-Dioxidation (31.98983 Da), K18-GLAP (109.02805 Da)
Charge: +4, Monoisotopic m/z: 586.76990 Da (+0.24 mmu/+0.41 ppm), MH+: 2344.05777 Da, RT: 46.9035 min,
Identified with: Sequest HT (v1.17); XCorr:0.77, Percolator q-Value:3.5e-3, Percolator PEP:1.8e-2,
Fragment match tolerance used for search: 0.02 Da
Fragments used for search: -H_2O; y; -NH_3; y; b; b; -H_2O; b; -NH_3; y

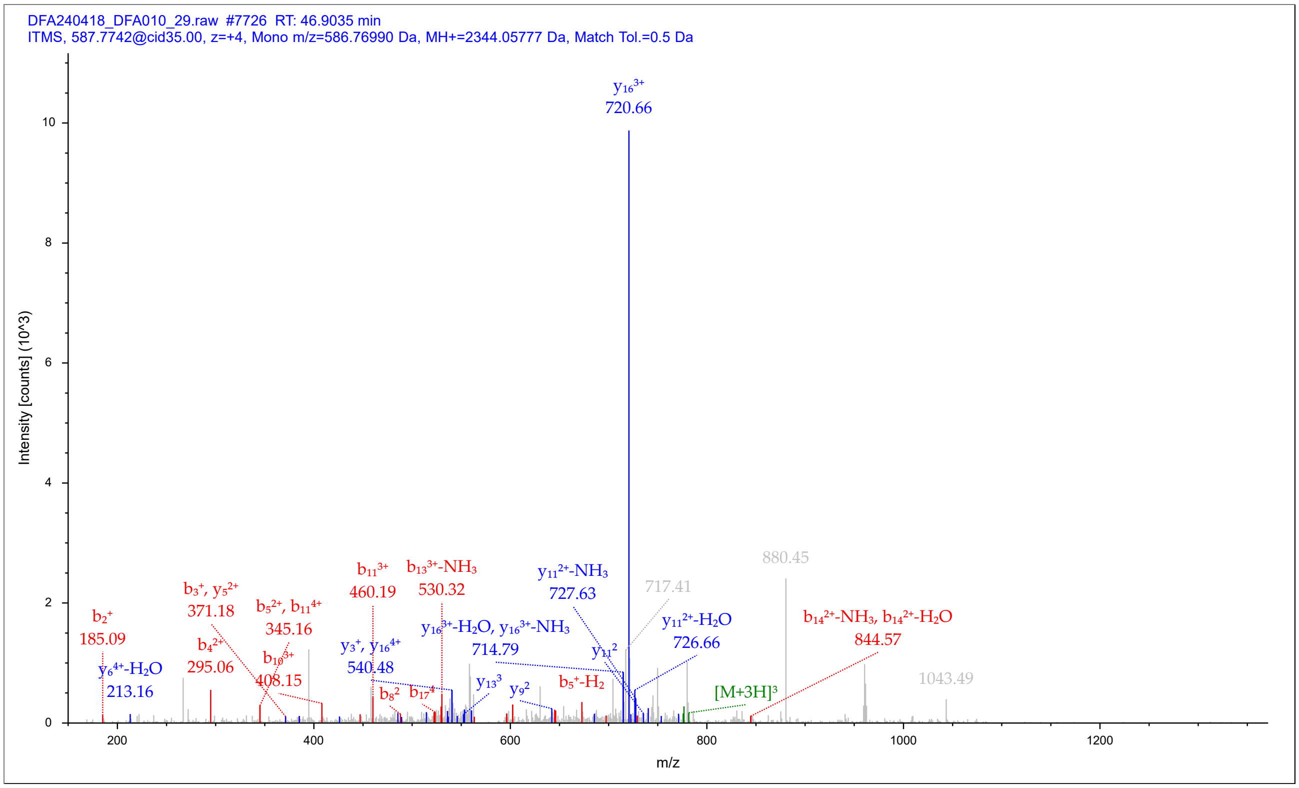

#1	b⁺	b²⁺	b³⁺	b⁴⁺	Seq.	y⁺	y²⁺	y³⁺	y⁴⁺	#2
1	88.03930	44.52329	30.01795	22.76528	S					18
2	185.09207	93.04967	62.36887	47.02847	P	2257.02478	1129.01603	753.01311	565.01165	17
3	371.17138	186.08933	124.39531	93.54830	W	2159.97202	1080.48965	720.66219	540.74846	16
4	589.24052	295.12390	197.08503	148.06559	W-Dioxidation	1973.89271	987.44999	658.63575	494.22863	15
5	690.28820	345.64774	230.76759	173.32751	T	1755.82357	878.41542	585.94604	439.71135	14
6	787.34097	394.17412	263.11851	197.59070	P	1654.77589	827.89158	552.26348	414.44943	13
7	874.37299	437.69014	292.12918	219.34871	S	1557.72312	779.36520	519.91256	390.18624	12
8	975.42067	488.21397	325.81174	244.61063	T	1470.69110	735.84919	490.90188	368.42823	11
9	1062.45270	531.72999	354.82242	266.36863	S	1369.64342	685.32535	457.21932	343.16631	10
10	1222.48335	611.74531	408.16597	306.37629	C-Carbamidomethyl	1282.61139	641.80933	428.20865	321.40830	9
11	1378.58446	689.79587	460.19967	345.40157	R	1122.58074	561.79401	374.86510	281.40064	8
12	1475.63722	738.32225	492.55059	369.66476	P	966.47963	483.74345	322.83139	242.37536	7
13	1604.67982	802.84355	535.56479	401.92541	E	869.42687	435.21707	290.48047	218.11217	6
14	1705.72750	853.36739	569.24735	427.18733	T	740.38427	370.69577	247.46628	185.85153	5
15	1804.79591	902.90159	602.27015	451.95443	V	639.33659	320.17194	213.78372	160.58961	4
16	1951.86432	976.43580	651.29296	488.72154	F	540.26818	270.63773	180.76091	135.82250	3
17	2088.92324	1044.96526	696.97926	522.98627	H	393.19977	197.10352	131.73811	99.05540	2
18					K-GLAP	256.14085	128.57407	86.05180	64.79067	1

Sequence: KAADDTWEPFASGK, W7-Dioxidation (31.98983 Da)
Charge: +3, Monoisotopic m/z: 518.90717 Da (-0.04 mmu/-0.09 ppm), MH+: 1554.70694 Da, RT: 48.2269 min,
Identified with: Sequest HT (v1.17); XCorr:0.71, Percolator q-Value:8.3e-3, Percolator PEP:2.5e-2,
Fragment match tolerance used for search: 0.02 Da
Fragments used for search: -H_2O; y; -NH_3; y; b; b; -H_2O; b; -NH_3; y

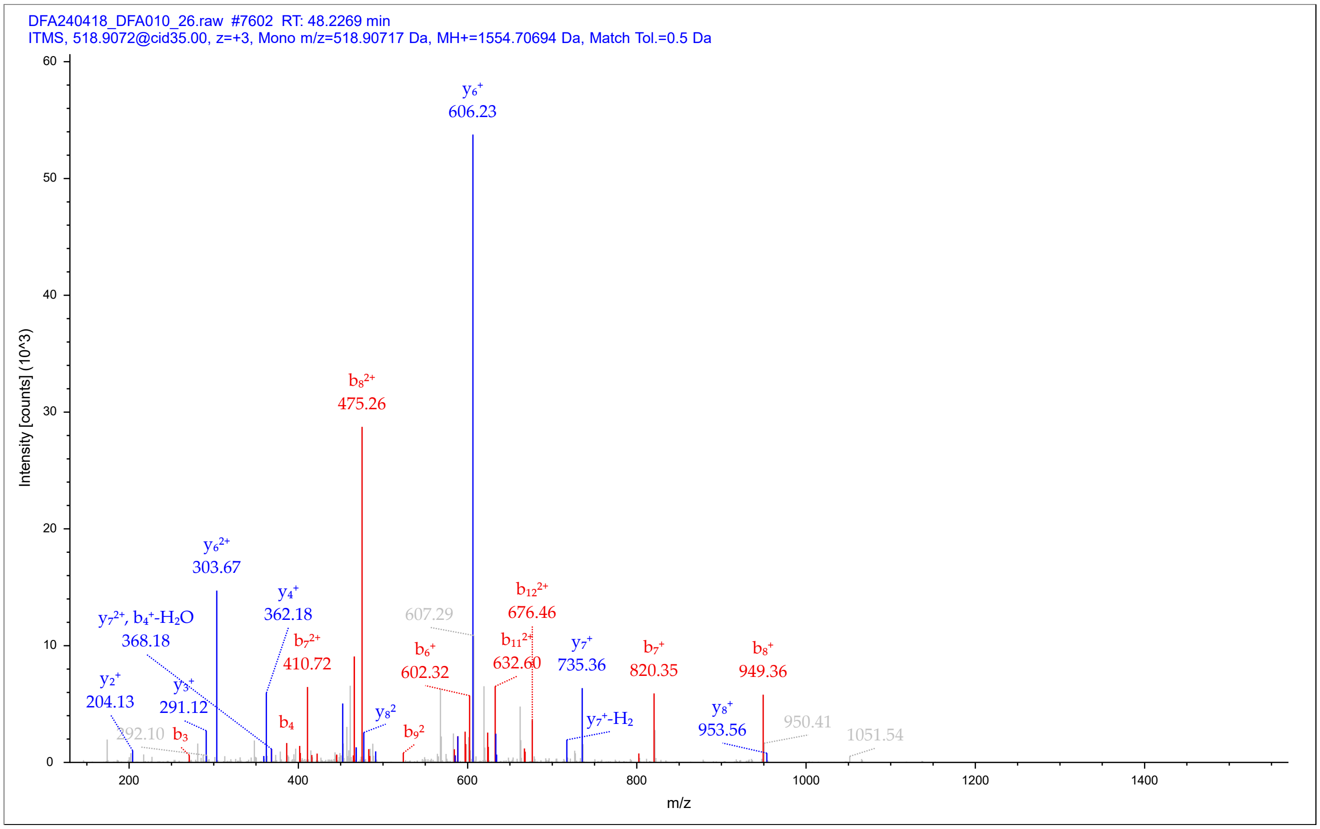

Sequence: AGALSGGNLQK, K11-b-ketonium ion (96.02056 Da)
Charge: +2, Monoisotopic m/z: 556.29034 Da (-0.12 mmu/-0.21 ppm), MH+: 1111.57341 Da, RT: 48.5703 min,
Identified with: Sequest HT (v1.17); XCorr:0.70, Percolator q-Value:5.1e-3, Percolator PEP:4.0e-2,
Fragment match tolerance used for search: 0.02 Da
Fragments used for search: -H_2O; y; -NH_3; y; b; b; -H_2O; b; -NH_3; y

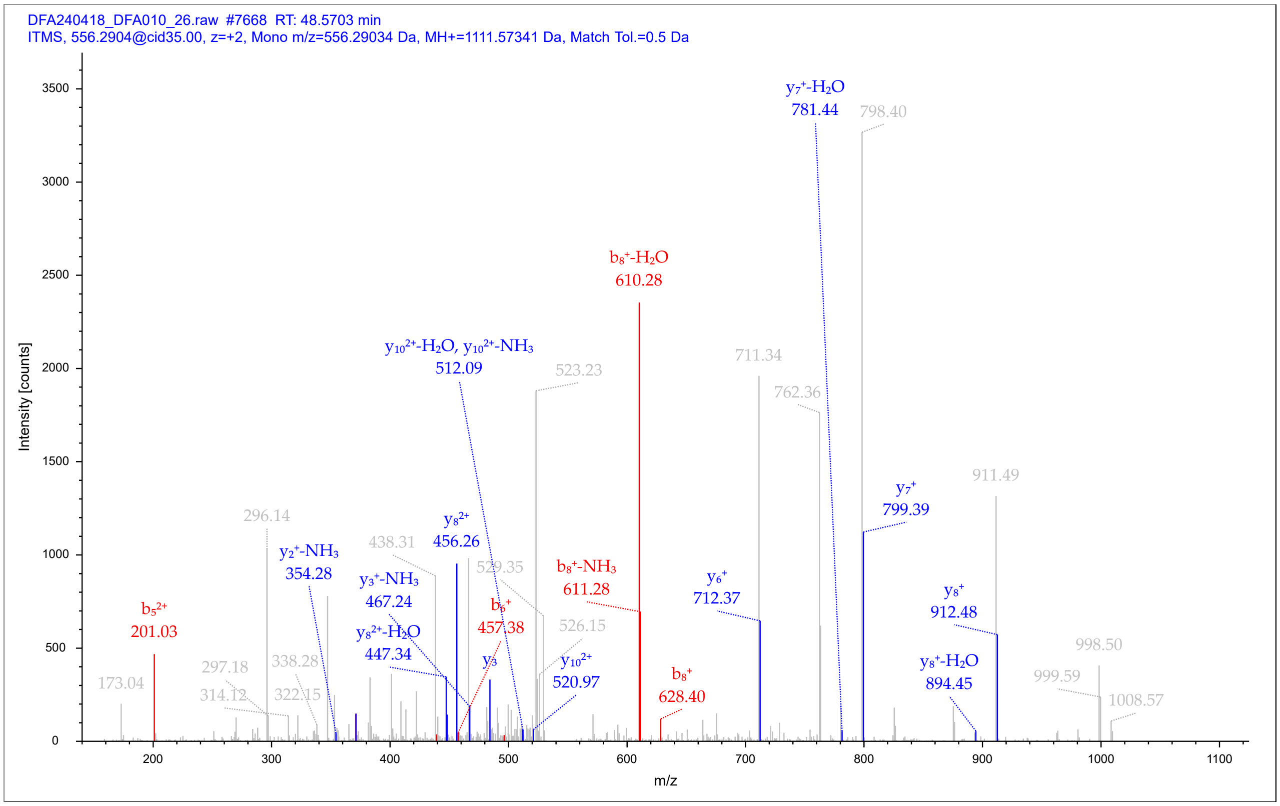

#1	b⁺	b²⁺	Seq.	y⁺	y²⁺	#2
1	72.04439	36.52583	A			11
2	129.06585	65.03657	G	1040.53653	520.77190	10
3	200.10297	100.55512	A	983.51507	492.26117	9
4	313.18703	157.09715	L	912.47795	456.74261	8
5	400.21906	200.61317	S	799.39389	400.20058	7
6	457.24052	229.12390	G	712.36186	356.68457	6
7	514.26199	257.63463	G	655.34040	328.17384	5
8	628.30491	314.65610	N	598.31893	299.66310	4
9	741.38898	371.19813	L	484.27601	242.64164	3
10	869.44756	435.22742	Q	371.19194	186.09961	2
11			K-b-ketonium ion	243.13336	122.07032	1

Sequence: GKPSSDTVPKPK, K2-Carboxymethyl (58.00548 Da), K10-Glycerinyl (88.01674 Da)
Charge: +2, Monoisotopic m/z: 693.85925 Da (-0.28 mmu/-0.41 ppm), MH+: 1386.71123 Da, RT: 57.8223 min,
Identified with: Sequest HT (v1.17); XCorr:0.61, Percolator q-Value:5.5e-3, Percolator PEP:4.8e-2,
Fragment match tolerance used for search: 0.02 Da
Fragments used for search: -H_2O; y; -NH_3; y; b; b; -H_2O; y

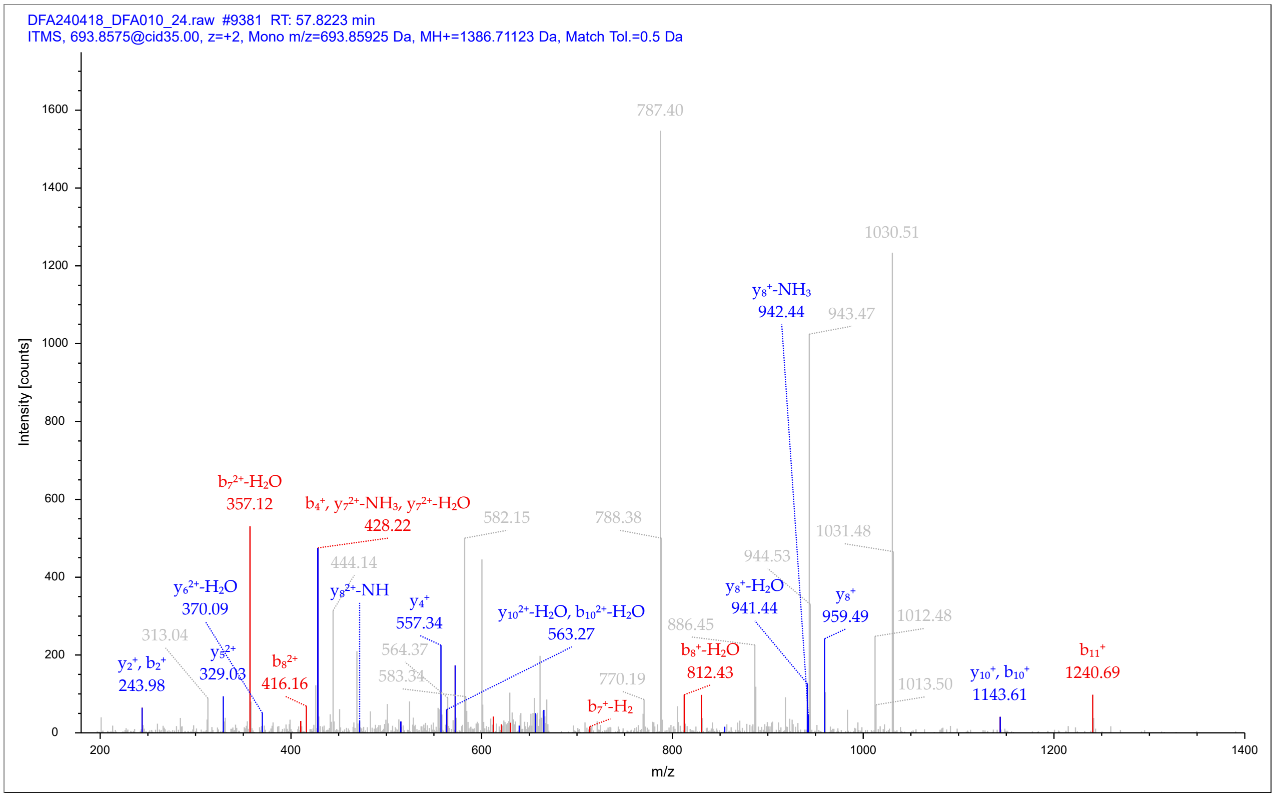

#1	b⁺	b²⁺	Seq.	y⁺	y²⁺	#2
1	58.02874	29.51801	G			12
2	244.12918	122.56823	K-Carboxy-methyl	1329.69033	665.34880	11
3	341.18195	171.09461	P	1143.58989	572.29858	10
4	428.21397	214.61063	S	1046.53713	523.77220	9
5	515.24600	258.12664	S	959.50510	480.25619	8
6	630.27295	315.64011	D	872.47307	436.74017	7
7	731.32062	366.16395	T	757.44613	379.22670	6
8	830.38904	415.69816	V	656.39845	328.70286	5
9	927.44180	464.22454	P	557.33003	279.16866	4
10	1143.55350	572.28039	K-Glycerinyl	460.27727	230.64227	3
11	1240.60627	620.80677	P	244.16557	122.58642	2
12			K	147.11280	74.06004	1

Sequence: WWQPYHSSQR, Y5-Oxidation (15.99492 Da), W1-Trp->Kynurenin (3.99492 Da), W2-Trp->Kynurenin (3.99492 Da)
Charge: +3, Monoisotopic m/z: 466.87714 Da (-0.56 mmu/-1.2 ppm), MH+: 1398.61686 Da, RT: 62.3827 min,
Identified with: Sequest HT (v1.17); XCorr:0.93, Percolator q-Value:4.8e-3, Percolator PEP:2.4e-2,
Fragment match tolerance used for search: 0.02 Da
Fragments used for search: -H_2O; y; -NH_3; y; b; b; -H_2O; b; -NH_3; y

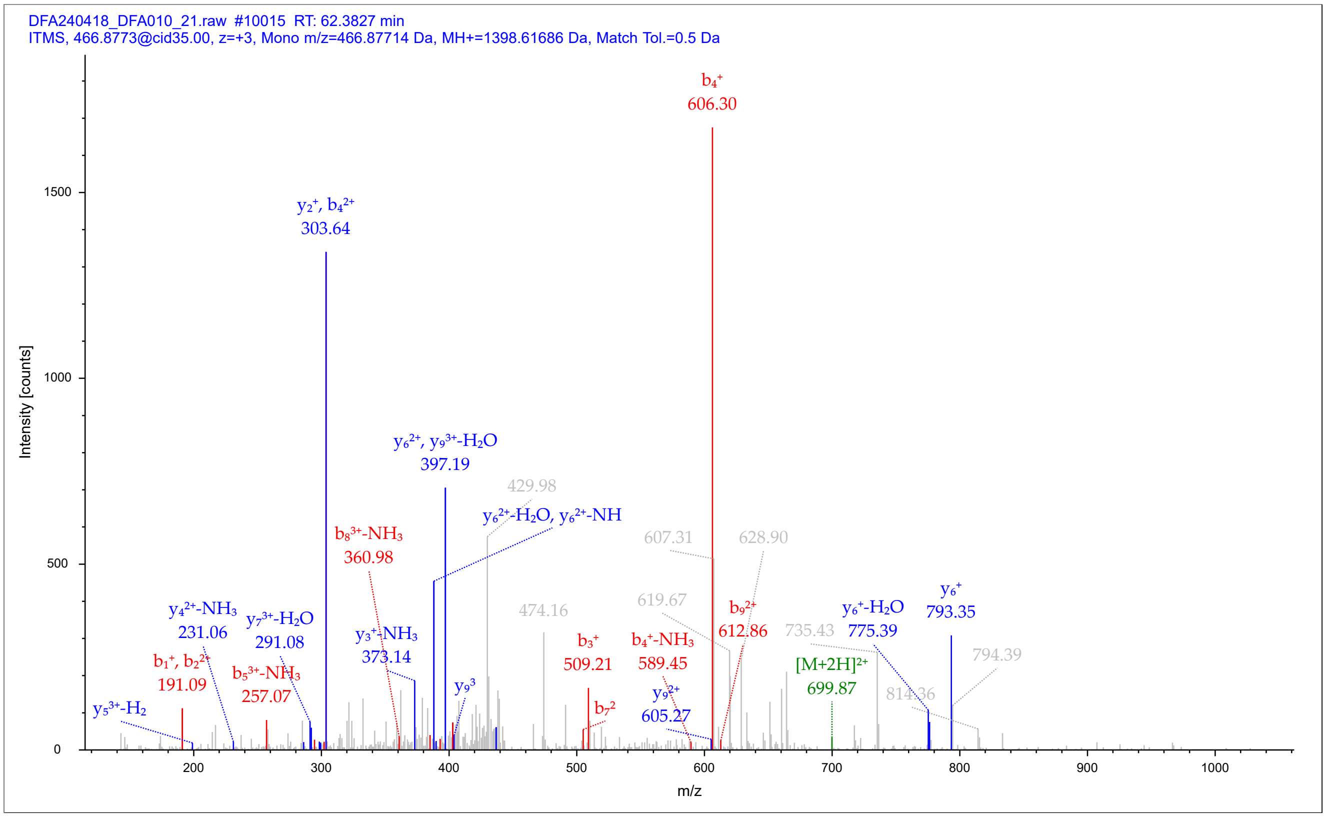

#1	b+	b2+	b3+	Seq.	y+	y2+	y3+	#2
1	191.08150	96.04439	64.36535	W-Trp->Kynurenin				10
2	381.15573	191.08150	127.72343	W-Trp->Kynurenin	1208.54431	604.77579	403.51962	9
3	509.21431	255.11079	170.40962	Q	1018.47008	509.73868	340.16155	8
4	606.26707	303.63718	202.76054	P	890.41151	445.70939	297.47535	7
5	785.32532	393.16630	262.44662	Y-Oxidation	793.35874	397.18301	265.12443	6
6	922.38423	461.69575	308.13293	H	614.30050	307.65389	205.43835	5
7	1009.41626	505.21177	337.14360	S	477.24159	239.12443	159.75205	4
8	1096.44829	548.72778	366.15428	S	390.20956	195.60842	130.74137	3
9	1224.50686	612.75707	408.84047	Q	303.17753	152.09240	101.73069	2
10				R	175.11895	88.06311	59.04450	1

Sequence: AADDTWEPFASGK, W6-Trp->Kynurenin (3.99492 Da)
Charge: +2, Monoisotopic m/z: 699.81226 Da (+0.02 mmu/+0.02 ppm), MH+: 1398.61724 Da, RT: 62.5143 min,
Identified with: Sequest HT (v1.17); XCorr:1.00, Percolator q-Value:2.1e-3, Percolator PEP:1.6e-2,
Fragment match tolerance used for search: 0.02 Da
Fragments used for search: $-H_2O$; y; $-NH_3$; y; b; b; $-H_2O$; y

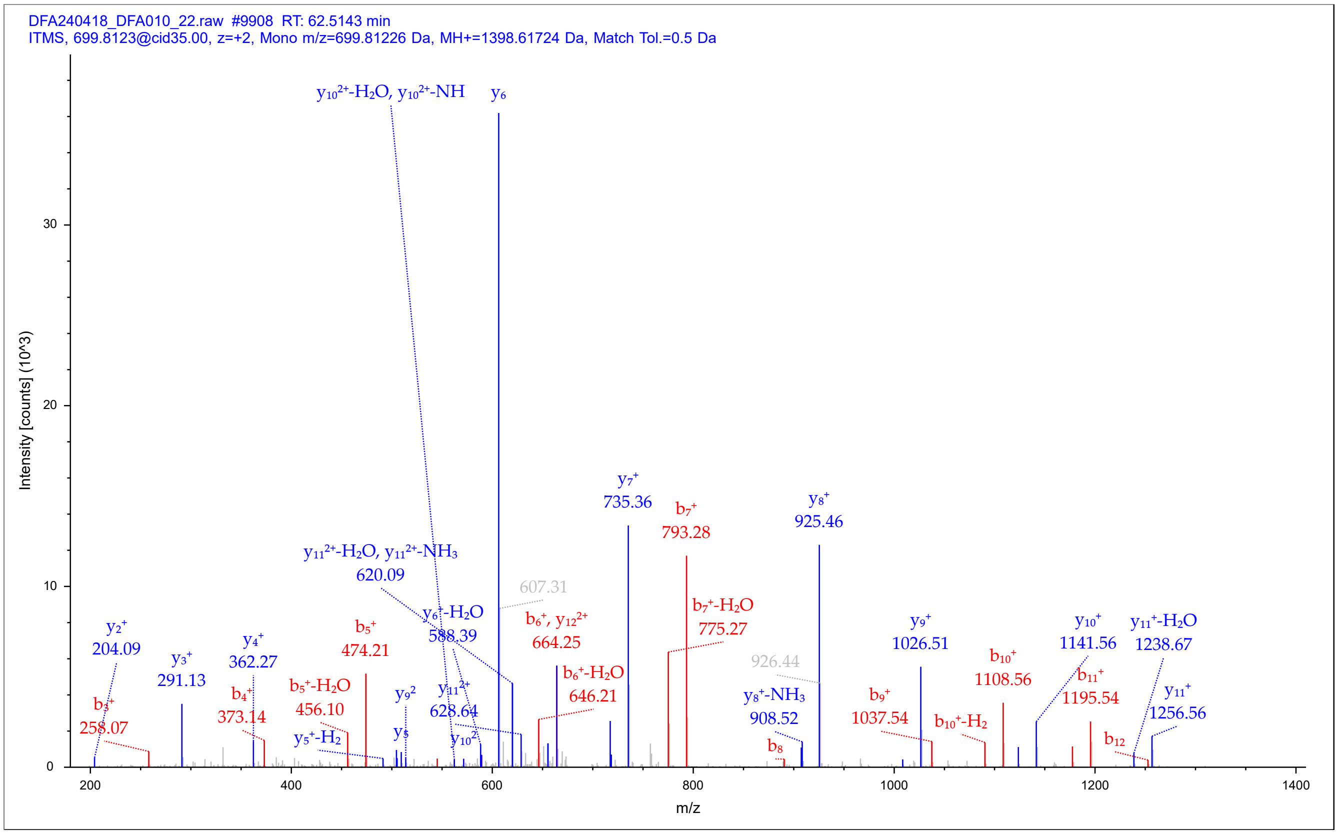

#1	b⁺	b²⁺	Seq.	y⁺	y²⁺	#2
1	72.04439	36.52583	A			13
2	143.08150	72.04439	A	1327.58009	664.29368	12
3	258.10845	129.55786	D	1256.54297	628.77512	11
4	373.13539	187.07133	D	1141.51603	571.26165	10
5	474.18307	237.59517	T	1026.48909	513.74818	9
6	664.25730	332.63229	W-Trp->Kynu-renin	925.44141	463.22434	8
7	793.29989	397.15358	E	735.36718	368.18723	7
8	890.35265	445.67996	P	606.32459	303.66593	6
9	1037.42107	519.21417	F	509.27182	255.13955	5
10	1108.45818	554.73273	A	362.20341	181.60534	4
11	1195.49021	598.24874	S	291.16630	146.08679	3
12	1252.51167	626.75947	G	204.13427	102.57077	2
13			K	147.11280	74.06004	1

49

Sequence: IKQFTLEEK, K2-Carboxyethyl (72.02113 Da), K9-C5-amide (148.03784 Da)
Charge: +3, Monoisotopic m/z: 452.56970 Da (-0.06 mmu/-0.12 ppm), MH+: 1355.69455 Da, RT: 66.2189 min,
Identified with: Sequest HT (v1.17); XCorr:0.87, Percolator q-Value:5.9e-3, Percolator PEP:3.2e-2, ptmRS: Best Site Probabilities:K2(Carboxyethyl): 98.94,
Fragment match tolerance used for search: 0.02 Da
Fragments used for search: -H_2O; y; -NH_3; y; b; b; -H_2O; b; -NH_3; y

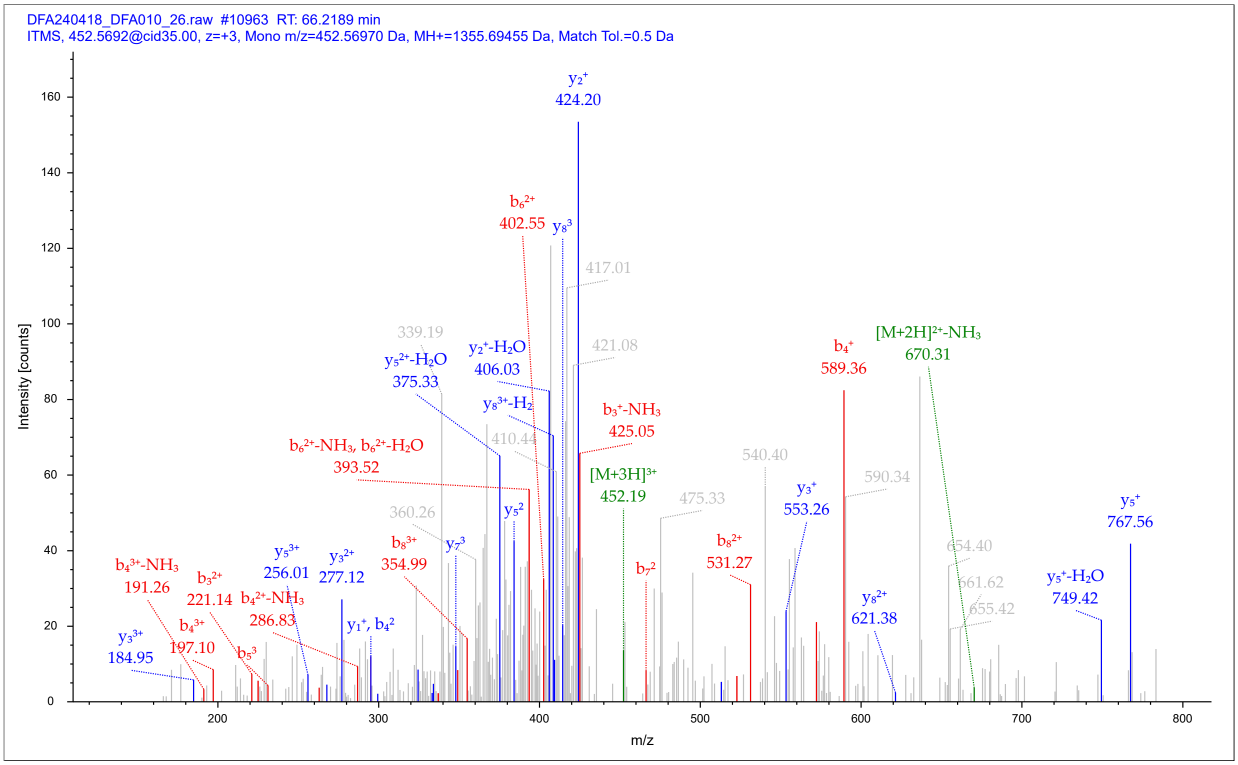

#1	b⁺	b²⁺	b³⁺	Seq.	y⁺	y²⁺	y³⁺	#2
1	114.09134	57.54931	38.70196	I				9
2	314.20743	157.60735	105.40733	K-Carboxy ethyl	1242.61066	621.80897	414.87507	8
3	442.26601	221.63664	148.09352	Q	1042.49456	521.75092	348.16971	7
4	589.33442	295.17085	197.11633	F	914.43599	457.72163	305.48351	6
5	690.38210	345.69469	230.79889	T	767.36757	384.18742	256.46071	5
6	803.46617	402.23672	268.49357	L	666.31989	333.66359	222.77815	4
7	932.50876	466.75802	311.50777	E	553.23583	277.12155	185.08346	3
8	1061.55135	531.27931	354.52197	E	424.19324	212.60026	142.06926	2
9				K-C5-amide	295.15064	148.07896	99.05507	1

171 (CEX/MGH)

Sequence: DASGVTFTWTPSSGK, F7-Oxidation (15.99492 Da), W9-Trp->Kynurenin (3.99492 Da), K15-Formyl (27.99492 Da)
Charge: +2, Monoisotopic m/z: 794.85962 Da (-0.3 mmu/-0.38 ppm), MH+: 1588.71196 Da, RT: 71.8592 min,
Identified with: Sequest HT (v1.17); XCorr:0.80, Percolator q-Value:2.1e-2, Percolator PEP:8.8e-2,
Fragment match tolerance used for search: 0.02 Da
Fragments used for search: -H_2O; y; b; b; -H_2O; y

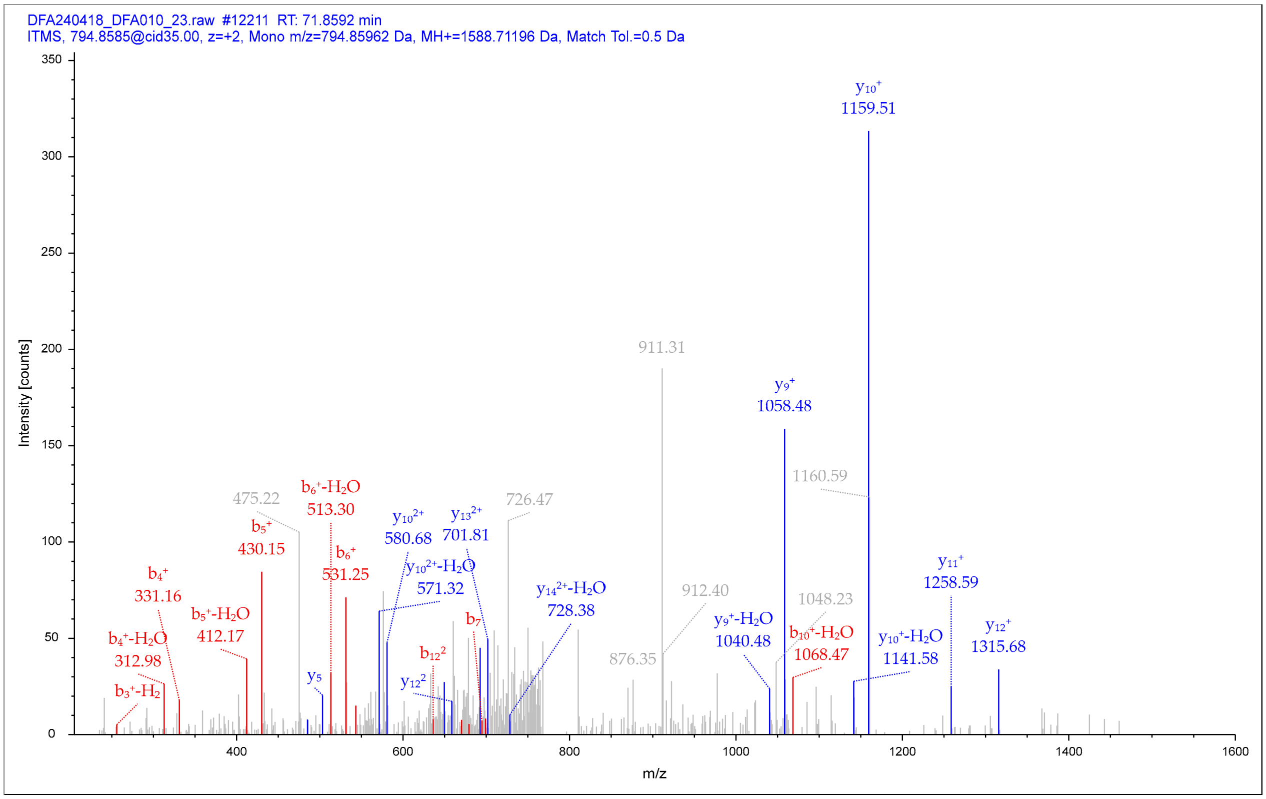

#1	b⁺	b²⁺	Seq.	y⁺	y²⁺	#2
1	116.03422	58.52075	D			15
2	187.07133	94.03930	A	1473.68562	737.34645	14
3	274.10336	137.55532	S	1402.64850	701.82789	13
4	331.12483	166.06605	G	1315.61647	658.31187	12
5	430.19324	215.60026	V	1258.59501	629.80114	11
6	531.24092	266.12410	T	1159.52660	580.26694	10
7	694.30425	347.65576	F-Oxidation	1058.47892	529.74310	9
8	795.35192	398.17960	T	895.41559	448.21143	8
9	985.42615	493.21671	W-Trp-> Kynurenin	794.36791	397.68759	7
10	1086.47383	543.74055	T	604.29368	302.65048	6
11	1183.52660	592.26694	P	503.24600	252.12664	5
12	1270.55862	635.78295	S	406.19324	203.60026	4
13	1357.59065	679.29896	S	319.16121	160.08424	3
14	1414.61212	707.80970	G	232.12918	116.56823	2
15			K-Formyl	175.10772	88.05750	1

199

Sequence: WDRPPDPENWVVK, K13-Acetyl (42.01057 Da), W10-Trp->Kynurenin (3.99492 Da), R3-Glarg (39.99949 Da)
Charge: +3, Monoisotopic m/z: 575.27576 Da (+0.23 mmu/+0.39 ppm), MH+: 1723.81272 Da, RT: 63.0125 min, Identified with: Sequest HT (v1.17); XCorr:0.65, Percolator q-Value:3.2e-3, Percolator PEP:2.9e-2,
Fragment match tolerance used for search: 0.02 Da
Fragments used for search: -H_2O; y; -NH_3; y; b; b; -H_2O; b; -NH_3; y

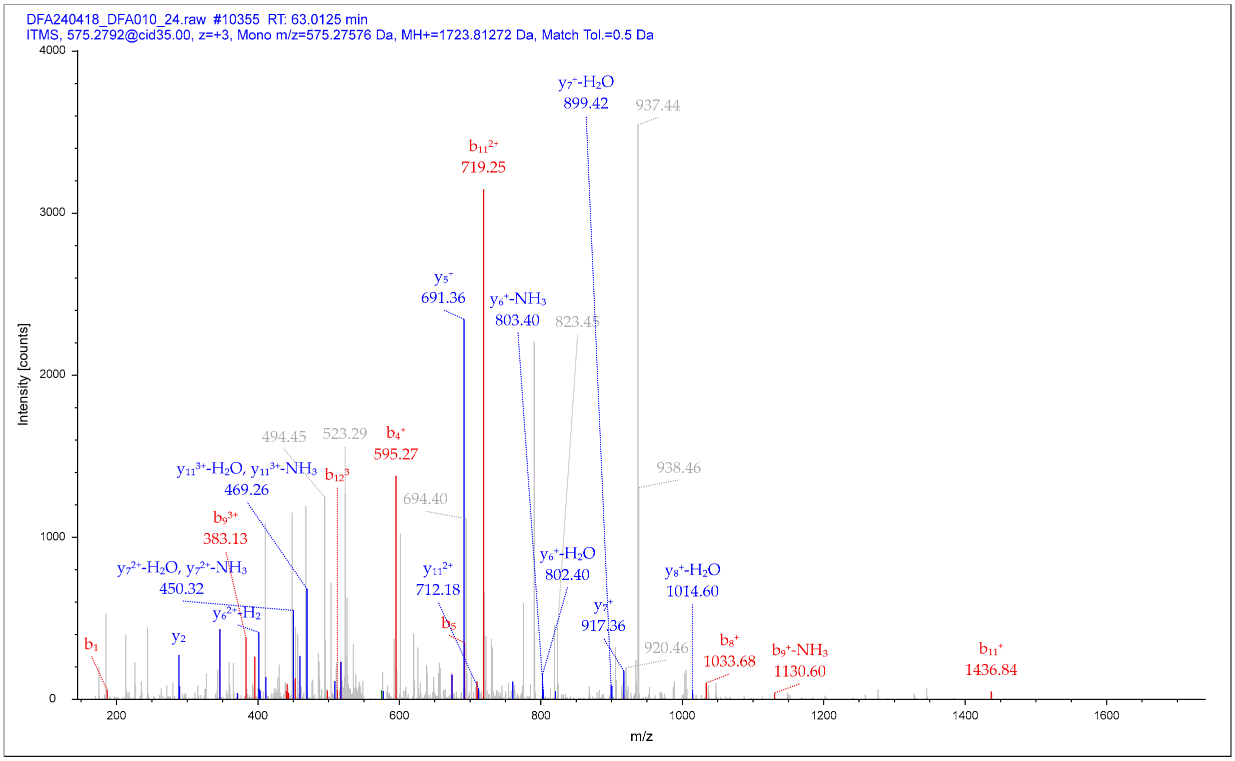

#1	b⁺	b²⁺	b³⁺	Seq.	y⁺	y²⁺	y³⁺	#2
1	187.08659	94.04693	63.03371	W				13
2	302.11353	151.56040	101.37603	D	1537.73273	769.37000	513.24909	12
3	498.21414	249.61071	166.74290	R-Glarg	1422.70578	711.85653	474.90678	11
4	595.26690	298.13709	199.09382	P	1226.60518	613.80623	409.53991	10
5	692.31966	346.66347	231.44474	P	1129.55242	565.27985	377.18899	9
6	807.34661	404.17694	269.78705	D	1032.49965	516.75346	344.83807	8
7	904.39937	452.70332	302.13797	P	917.47271	459.23999	306.49575	7
8	1033.44196	517.22462	345.15217	E	820.41995	410.71361	274.14483	6
9	1147.48489	574.24608	383.16648	N	691.37735	346.19231	231.13064	5
10	1337.55912	669.28320	446.52456	W-Trp->Kynurenin	577.33442	289.17085	193.11633	4
11	1436.62753	718.81740	479.54736	V	387.26020	194.13374	129.75825	3
12	1535.69595	768.35161	512.57017	V	288.19178	144.59953	96.73545	2
13				K-Acetyl	189.12337	95.06532	63.71264	1

135

Sequence: TQTAGGEDPGALR, R13-Tetrosyl (102.01840 Da)
Charge: +2,   Monoisotopic m/z: 687.82037 Da (-1.4 mmu/-2.04 ppm),   MH+: 1374.63347 Da,   RT: 52.0533 min,
Identified with: Sequest HT (v1.17); XCorr:0.65, Percolator q-Value:5.0e-3, Percolator PEP:2.9e-2,
Fragment match tolerance used for search: 0.02 Da
Fragments used for search: -H_2O; y; -NH_3; y; b; b; -H_2O; b; -NH_3; y

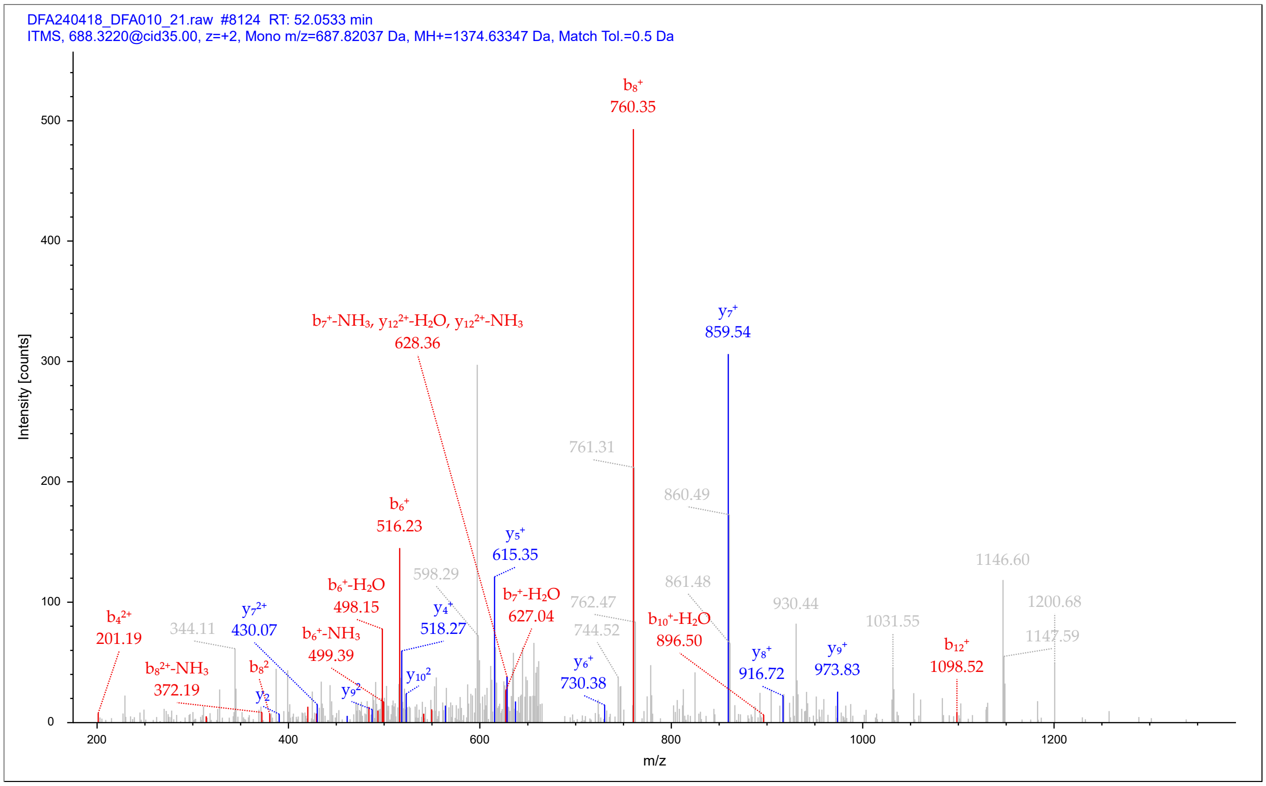

#1	b⁺	b²⁺	Seq.	y⁺	y²⁺	#2
1	102.05496	51.53112	T			13
2	230.11353	115.56040	Q	1273.58859	637.29793	12
3	331.16121	166.08424	T	1145.53001	573.26864	11
4	402.19832	201.60280	A	1044.48233	522.74481	10
5	459.21979	230.11353	G	973.44522	487.22625	9
6	516.24125	258.62426	G	916.42376	458.71552	8
7	645.28385	323.14556	E	859.40229	430.20479	7
8	760.31079	380.65903	D	730.35970	365.68349	6
9	857.36355	429.18541	P	615.33276	308.17002	5
10	914.38502	457.69615	G	518.27999	259.64364	4
11	985.42213	493.21470	A	461.25853	231.13290	3
12	1098.50619	549.75673	L	390.22142	195.61435	2
13			R-Tetrosyl	277.13735	139.07231	1

Sequence: MIQAVVDNVCWQMSLDR, C10-Carbamidomethyl (57.02146 Da), R17-Carboxymethyl (58.00548 Da), M1-Oxidation (15.99492 Da), W11-Trioxidation (47.98474 Da), M13-Trioxidation (47.98474 Da)
Charge: +5, Monoisotopic m/z: 447.79263 Da (-0.44 mmu/-0.98 ppm), MH+: 2234.93406 Da, RT: 14.8028 min,
Identified with: Sequest HT (v1.17); XCorr:0.73, Percolator q-Value:3.4e-3, Percolator PEP:3.9e-2,
Fragment match tolerance used for search: 0.02 Da
Fragments used for search: $-H_2O$; y; $-NH_3$; y; b; b; $-H_2O$; b; $-NH_3$; y

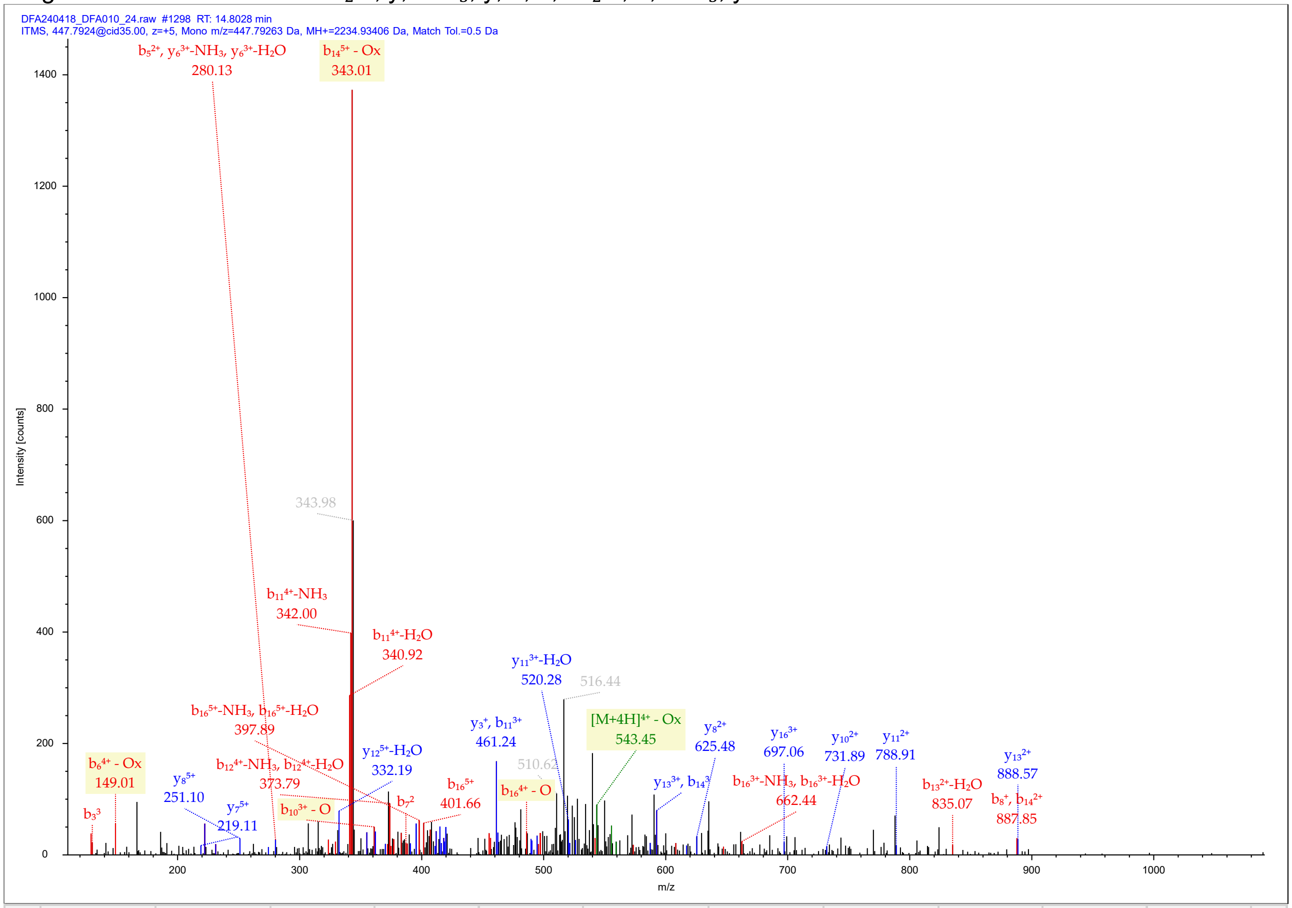

#1	b⁺	b²⁺	b³⁺	b⁴⁺	b⁵⁺	Seq.	y⁺	y²⁺	y³⁺	y⁴⁺	y⁵⁺	#2
1	148.04268	74.52498	50.01908	37.76613	30.41436	M-Oxidation						17
2	261.12674	131.06701	87.71376	66.03714	53.03117	I	2087.90084	1044.45406	696.63847	522.73067	418.38599	16
3	389.18532	195.09630	130.39996	98.05179	78.64288	Q	1974.81678	987.91203	658.94378	494.45965	395.76918	15
4	460.22243	230.61485	154.07899	115.81107	92.85031	A	1846.75820	923.88274	616.25759	462.44501	370.15746	14
5	559.29085	280.14906	187.10180	140.57817	112.66399	V	1775.72109	888.36418	592.57855	444.68573	355.95004	13
6	658.35926	329.68327	220.12460	165.34527	132.47767	V	1676.65268	838.82998	559.55574	419.91863	336.13636	12
7	773.38620	387.19674	258.46692	194.10201	155.48306	D	1577.58426	789.29577	526.53294	395.15152	316.32267	11
8	887.42913	444.21820	296.48123	222.61274	178.29165	N	1462.55732	731.78230	488.19062	366.39479	293.31729	10
9	986.49754	493.75241	329.50403	247.37984	198.10533	V	1348.51439	674.76083	450.17631	337.88406	270.50870	9
10	1146.52819	573.76773	382.84758	287.38751	230.11146	C-Carbamido-methyl	1249.44598	625.22663	417.15351	313.11695	250.69502	8
11	1380.59225	690.79976	460.86893	345.90352	276.92427	W-Trioxidation	1089.41533	545.21130	363.80996	273.10929	218.68889	7
12	1508.65083	754.82905	503.55513	377.91816	302.53599	Q	855.35127	428.17927	285.78861	214.59328	171.87608	6
13	1687.67605	844.34167	563.23020	422.67447	338.34103	M-Trioxidation	727.29270	364.14999	243.10242	182.57863	146.26436	5
14	1774.70808	887.85768	592.24088	444.43248	355.74744	S	548.26747	274.63737	183.42734	137.82232	110.45931	4
15	1887.79215	944.39971	629.93557	472.70349	378.36425	L	461.23544	231.12136	154.41666	116.06432	93.05291	3
16	2002.81909	1001.91318	668.27788	501.46023	401.36964	D	348.15137	174.57933	116.72198	87.79330	70.43610	2
17						R-Carboxy-methyl	233.12443	117.06585	78.37966	59.03657	47.43071	1

Sequence: EASKQLLLWR, W9-Oxidation (15.99492 Da), R10-Delta:H(2)C(3)O(1) (54.01057 Da)
Charge: +3, Monoisotopic m/z: 438.57715 Da (-1.44 mmu/-3.29 ppm), MH+: 1313.71689 Da, RT: 24.0889 min,
Identified with: Sequest HT (v1.17); XCorr:1.29, Percolator q-Value:6.3e-3, Percolator PEP:3.3e-2,
Fragment match tolerance used for search: 0.02 Da
Fragments used for search: -H_2O; y; -NH_3; y; b; b; -H_2O; b; -NH_3; y

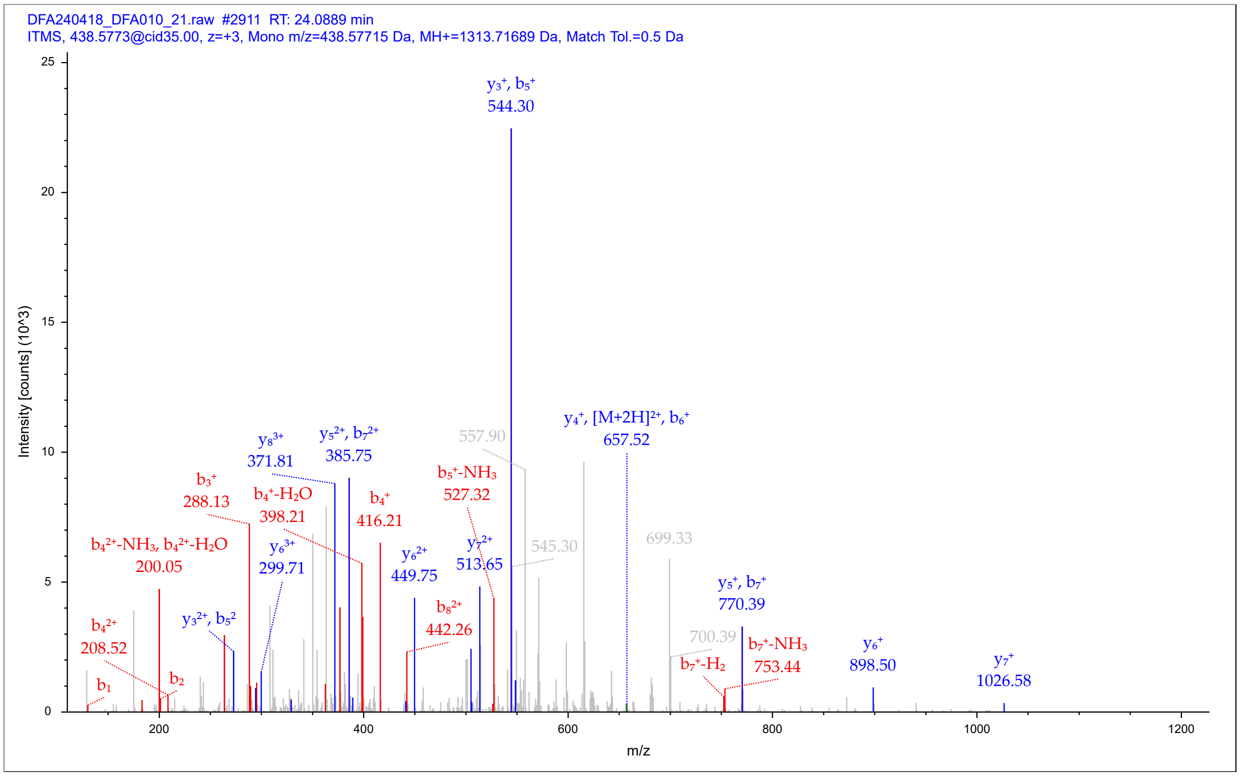

#1	b+	b2+	b3+	Seq.	y+	y2+	y3+	#2
1	130.04987	65.52857	44.02147	E				10
2	201.08698	101.04713	67.70051	A	1184.67862	592.84295	395.56439	9
3	288.11901	144.56314	96.71119	S	1113.64151	557.32439	371.88535	8
4	416.21397	208.61063	139.40951	K	1026.60948	513.80838	342.87468	7
5	544.27255	272.63991	182.09570	Q	898.51451	449.76090	300.17636	6
6	657.35662	329.18195	219.79039	L	770.45594	385.73161	257.49016	5
7	770.44068	385.72398	257.48508	L	657.37187	329.18957	219.79548	4
8	883.52474	442.26601	295.17977	L	544.28781	272.64754	182.10079	3
9	1085.59897	543.30312	362.53784	W-Oxidation	431.20375	216.10551	144.40610	2
10				MGH	229.12952	115.06840	77.04802	1

Sequence: WQQQGDLQDTK, W1-Trp->Kynurenin (3.99492 Da)
Charge: +2, Monoisotopic m/z: 675.81580 Da (-2.06 mmu/-3.05 ppm), MH+: 1350.62432 Da, RT: 26.8859 min,
Identified with: Sequest HT (v1.17); XCorr:0.88, Percolator q-Value:2.1e-3, Percolator PEP:2.0e-2,
Fragment match tolerance used for search: 0.02 Da
Fragments used for search: -H_2O; y; -NH_3; y; b; b; -H_2O; b; -NH_3; y

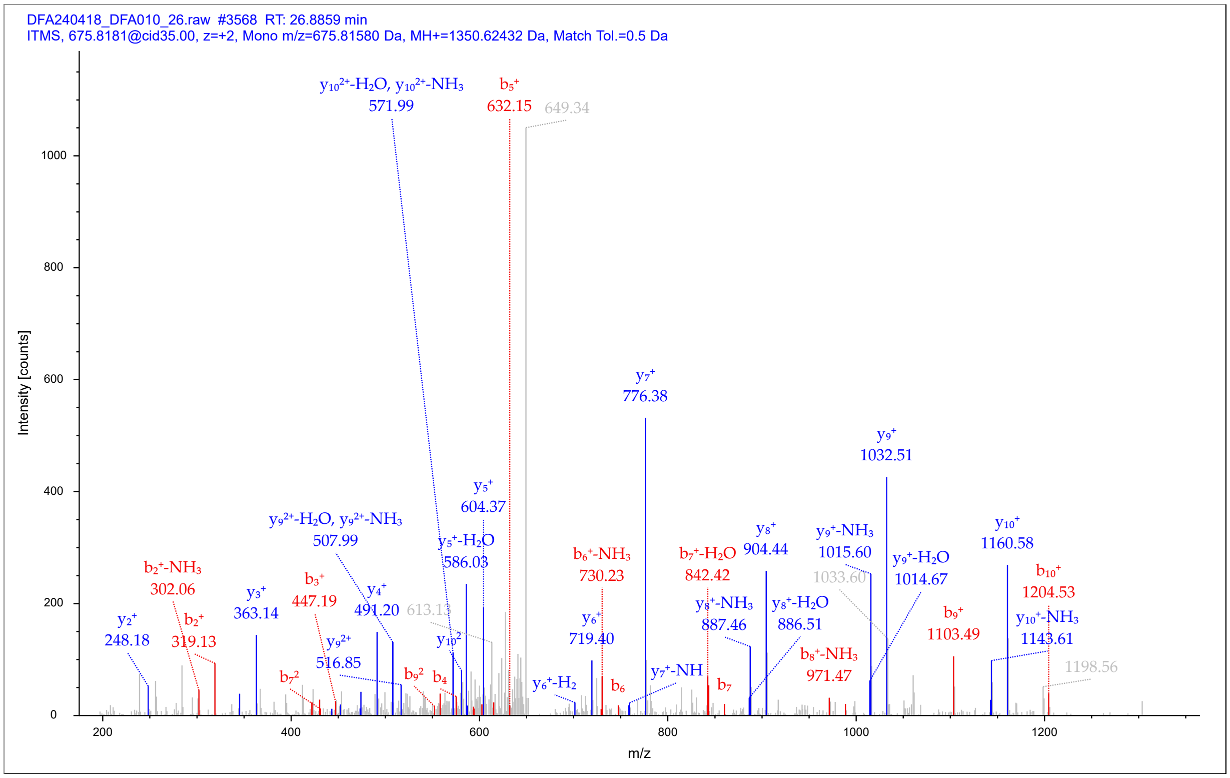

#1	b⁺	b²⁺	Seq.	y⁺	y²⁺	#2
1	191.08150	96.04439	W-Trp->Kynurenin			11
2	319.14008	160.07368	Q	1160.55421	580.78074	10
3	447.19866	224.10297	Q	1032.49563	516.75145	9
4	575.25724	288.13226	Q	904.43705	452.72216	8
5	632.27870	316.64299	G	776.37847	388.69288	7
6	747.30564	374.15646	D	719.35701	360.18214	6
7	860.38971	430.69849	L	604.33007	302.66867	5
8	988.44828	494.72778	Q	491.24600	246.12664	4
9	1103.47523	552.24125	D	363.18743	182.09735	3
10	1204.52291	602.76509	T	248.16048	124.58388	2
11			K	147.11280	74.06004	1

Sequence: QINDYVAK, K8-Carboxymethyl (58.00548 Da)
Charge: +2, Monoisotopic m/z: 504.75369 Da (+0.23 mmu/+0.45 ppm), MH+: 1008.50011 Da, RT: 30.4579 min,
Identified with: Sequest HT (v1.17); XCorr:0.67, Percolator q-Value:6.0e-3, Percolator PEP:4.2e-2,
Fragment match tolerance used for search: 0.02 Da
Fragments used for search: -H_2O; y; -NH_3; y; b; b; -H_2O; b; -NH_3; y

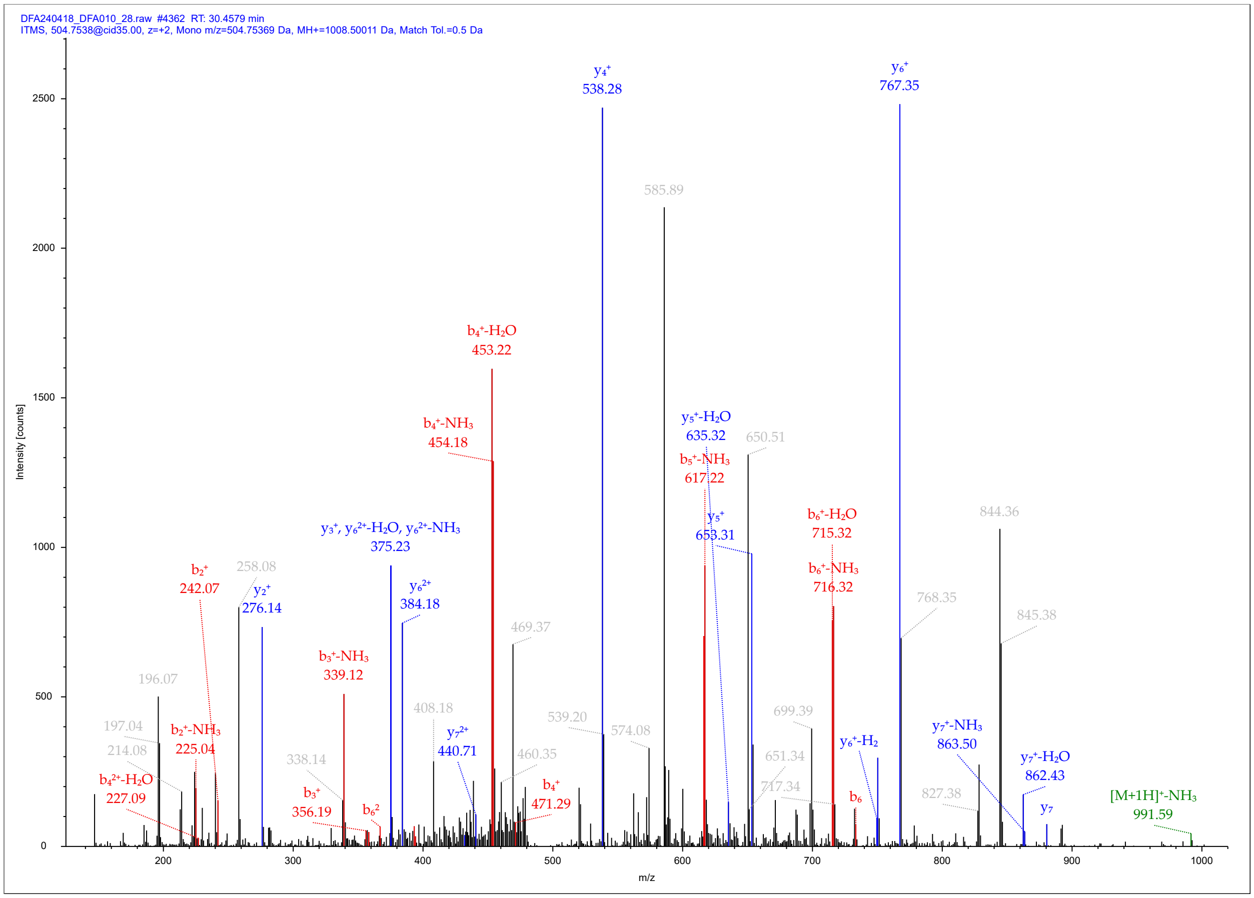

#1	b+	b2+	Seq.	y+	y2+	#2
1	129.06585	65.03657	Q			8
2	242.14992	121.57860	I	880.44107	440.72418	7
3	356.19285	178.60006	N	767.35701	384.18214	6
4	471.21979	236.11353	D	653.31408	327.16068	5
5	634.28312	317.64520	Y	538.28714	269.64721	4
6	733.35153	367.17940	V	375.22381	188.11554	3
7	804.38864	402.69796	A	276.15540	138.58134	2
8			K-Carboxymethyl	205.11828	103.06278	1

Sequence: AQPAERFFDPNQR, F7-Dioxidation (31.98983 Da), F8-Dioxidation (31.98983 Da), R13-2(Fructosyl) (324.10560 Da)
Charge: +2, Monoisotopic m/z: 982.42896 Da (-0.44 mmu/-0.45 ppm), MH+: 1963.85063 Da, RT: 30.9688 min,
Identified with: Sequest HT (v1.17); XCorr:0.81, Percolator q-Value:5.2e-3, Percolator PEP:1.1e-2,
Fragment match tolerance used for search: 0.02 Da
Fragments used for search: $-H_2O$; y; $-NH_3$; y; b; b; $-H_2O$; b; $-NH_3$; y

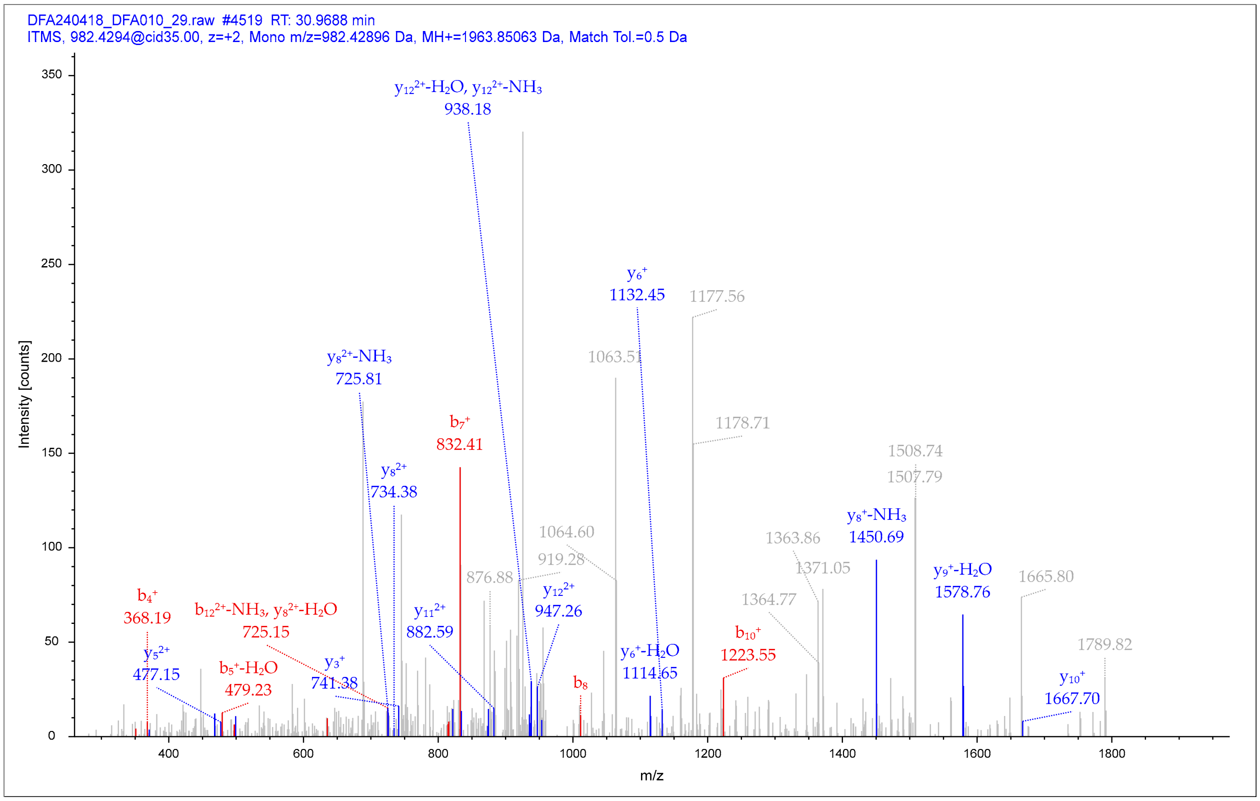

#1	b⁺	b²⁺	Seq.	y⁺	y²⁺	#2
1	72.04439	36.52583	A			13
2	200.10297	100.55512	Q	1892.81441	946.91084	12
3	297.15573	149.08150	P	1764.75583	882.88155	11
4	368.19285	184.60006	A	1667.70307	834.35517	10
5	497.23544	249.12136	E	1596.66595	798.83662	9
6	653.33655	327.17191	R	1467.62336	734.31532	8
7	832.39479	416.70103	F-Dioxidation	1311.52225	656.26476	7
8	1011.45304	506.23016	F-Dioxidation	1132.46401	566.73564	6
9	1126.47998	563.74363	D	953.40576	477.20652	5
10	1223.53274	612.27001	P	838.37882	419.69305	4
11	1337.57567	669.29147	N	741.32606	371.16667	3
12	1465.63425	733.32076	Q	627.28313	314.14520	2
13			R-2(Fructosyl)	499.22455	250.11591	1

69

Sequence: LHGWAPGPDYQK, W4-Oxidation (15.99492 Da), Y10-Oxidation (15.99492 Da), K12-Lederers pentosone (114.03234 Da)
Charge: +3, Monoisotopic m/z: 505.56940 Da (+0.65 mmu/+1.29 ppm), MH+: 1514.69364 Da, RT: 32.5866 min,
Identified with: Sequest HT (v1.17); XCorr:0.91, Percolator q-Value:1.4e-3, Percolator PEP:1.6e-2,
Fragment match tolerance used for search: 0.02 Da
Fragments used for search: -H_2O; y; -NH_3; y; b; b; -H_2O; b; -NH_3; y

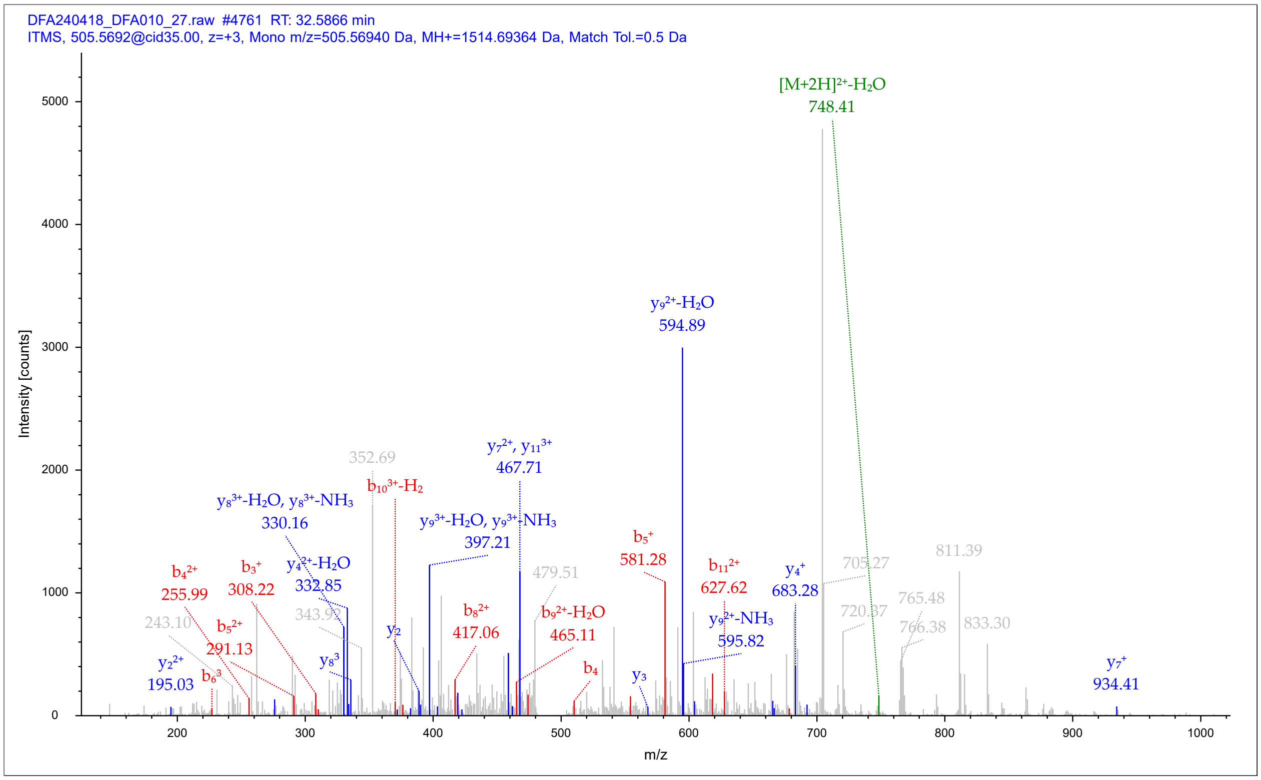

#1	b⁺	b²⁺	b³⁺	Seq.	y⁺	y²⁺	y³⁺	#2
1	114.09134	57.54931	38.70196	L				12
2	251.15025	126.07876	84.38827	H	1401.60762	701.30745	467.87406	11
3	308.17172	154.58950	103.39542	G	1264.54870	632.77799	422.18775	10
4	510.24594	255.62661	170.75350	W-Oxidation	1207.52724	604.26726	403.18060	9
5	581.28306	291.14517	194.43254	A	1005.45301	503.23014	335.82252	8
6	678.33582	339.67155	226.78346	P	934.41590	467.71159	312.14348	7
7	735.35729	368.18228	245.79061	G	837.36314	419.18521	279.79256	6
8	832.41005	416.70866	278.14153	P	780.34167	390.67447	260.78541	5
9	947.43699	474.22213	316.48385	D	683.28891	342.14809	228.43449	4
10	1126.49524	563.75126	376.16993	Y-Oxidation	568.26197	284.63462	190.09217	3
11	1254.55381	627.78054	418.85612	Q	389.20372	195.10550	130.40609	2
12				K-Lederers pentosone	261.14514	131.07621	87.71990	1

Sequence: LCSFQEEMAK, C2-Carbamidomethyl (57.02146 Da), K10-Carboxymethyl (58.00548 Da)
Charge: +3, Monoisotopic m/z: 434.19107 Da (+1.29 mmu/+2.97 ppm), MH+: 1300.55866 Da, RT: 33.9748 min,
Identified with: Sequest HT (v1.17); XCorr:0.89, Percolator q-Value:4.8e-3, Percolator PEP:3.2e-2,
Fragment match tolerance used for search: 0.02 Da
Fragments used for search: $-H_2O$; y; $-NH_3$; y; b; b; $-H_2O$; b; $-NH_3$; y

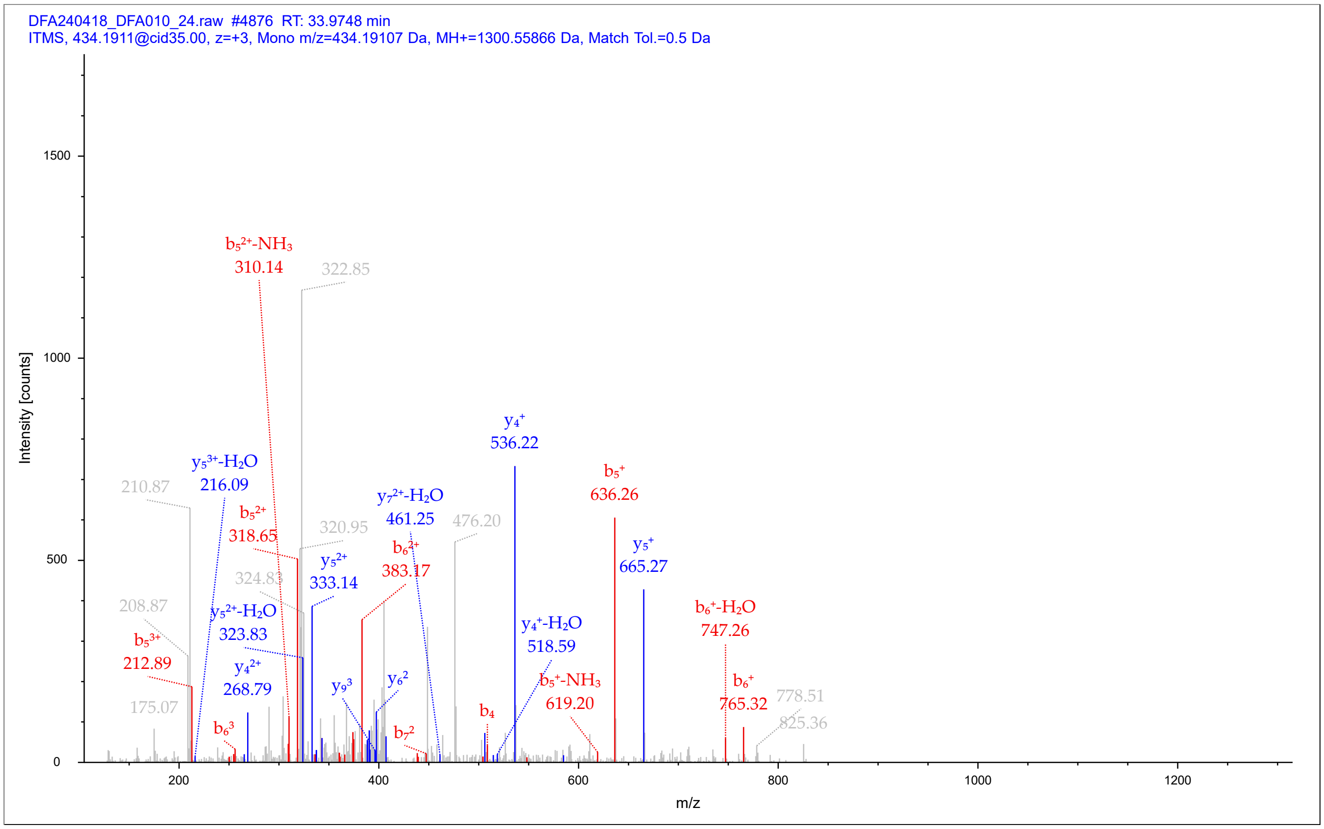

#1	b⁺	b²⁺	b³⁺	Seq.	y⁺	y²⁺	y³⁺	#2
1	114.09134	57.54931	38.70196	L				10
2	274.12199	137.56463	92.04551	C-Carbamido-methyl	1187.47074	594.23901	396.49510	9
3	361.15402	181.08065	121.05619	S	1027.44009	514.22368	343.15155	8
4	508.22243	254.61485	170.07899	F	940.40806	470.70767	314.14087	7
5	636.28101	318.64414	212.76519	Q	793.33965	397.17346	265.11807	6
6	765.32360	383.16544	255.77938	E	665.28107	333.14417	222.43187	5
7	894.36619	447.68674	298.79358	E	536.23847	268.62288	179.41768	4
8	1025.40668	513.20698	342.47374	M	407.19588	204.10158	136.40348	3
9	1096.44379	548.72553	366.15278	A	276.15540	138.58134	92.72332	2
10				K-Carboxy-methyl	205.11828	103.06278	69.04428	1

35 (CMX)

Sequence: FMQAVTGWK, F1-Oxidation (15.99492 Da), W8-Trp->Kynurenin (3.99492 Da)
Charge: +2, Monoisotopic m/z: 544.26459 Da (-1.1 mmu/-2.02 ppm), MH+: 1087.52190 Da, RT: 42.4771 min,
Identified with: Sequest HT (v1.17); XCorr:1.06, Percolator q-Value:9.2e-3, Percolator PEP:6.3e-2,
Fragment match tolerance used for search: 0.02 Da
Fragments used for search: -H_2O; y; -NH_3; y; b; b; -H_2O; b; -NH_3; y

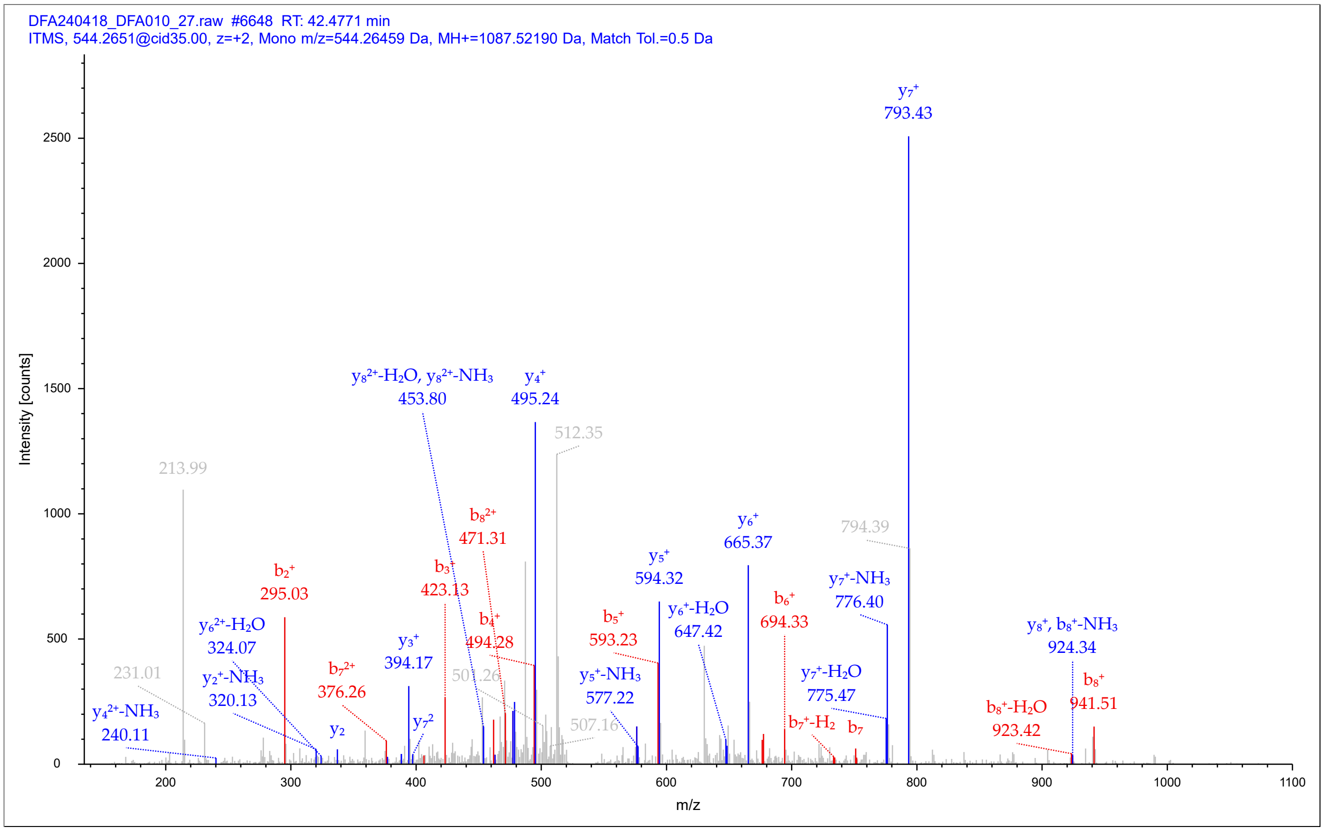

#1	b+	b2+	Seq.	y+	y2+	#2
1	164.07061	82.53894	F-Oxidation			9
2	295.11109	148.05918	M	924.46076	462.73402	8
3	423.16967	212.08847	Q	793.42028	397.21378	7
4	494.20678	247.60703	A	665.36170	333.18449	6
5	593.27520	297.14124	V	594.32459	297.66593	5
6	694.32287	347.66508	T	495.25617	248.13173	4
7	751.34434	376.17581	G	394.20850	197.60789	3
8	941.41857	471.21292	W-Trp->Kynurenin	337.18703	169.09715	2
9			K	147.11280	74.06004	1

35 (CEX)

Sequence: FMQAVTGWK, F1-Oxidation (15.99492 Da), W8-Trp->Kynurenin (3.99492 Da), K9-Formyl (27.99492 Da)
Charge: +2, Monoisotopic m/z: 558.26215 Da (-1 mmu/-1.79 ppm), MH+: 1115.51702 Da, RT: 42.1351 min,
Identified with: Sequest HT (v1.17); XCorr:1.04, Percolator q-Value:3.4e-2, Percolator PEP:1.4e-1,
Fragment match tolerance used for search: 0.02 Da
Fragments used for search: $-H_2O$; y; $-NH_3$; y; b; b; $-H_2O$; b; $-NH_3$; y

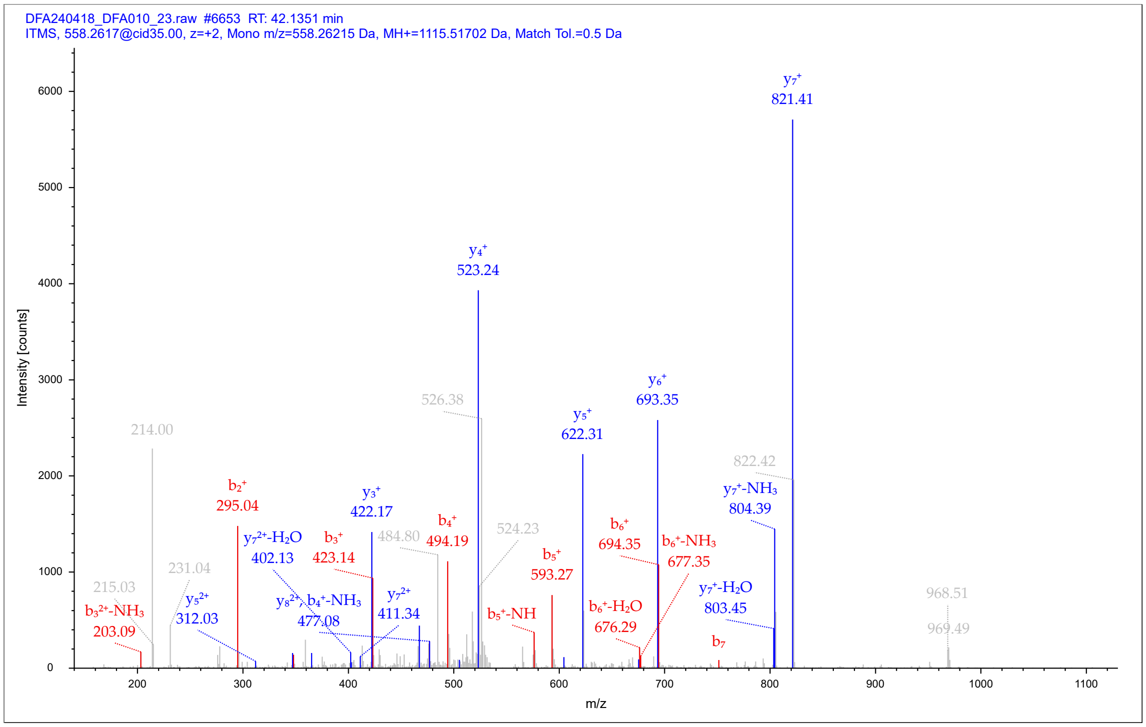

#1	b⁺	b²⁺	Seq.	y⁺	y²⁺	#2
1	164.07061	82.53894	F-Oxidation			9
2	295.11109	148.05918	M	952.45568	476.73148	8
3	423.16967	212.08847	Q	821.41519	411.21124	7
4	494.20678	247.60703	A	693.35662	347.18195	6
5	593.27520	297.14124	V	622.31950	311.66339	5
6	694.32287	347.66508	T	523.25109	262.12918	4
7	751.34434	376.17581	G	422.20341	211.60534	3
8	941.41857	471.21292	W-Trp-> Kynurenin	365.18195	183.09461	2
9			K-Formyl	175.10772	88.05750	1

Sequence: SHSMGYFSTSVSRPGR, R13-Carboxymethyl (58.00548 Da), R16-Carboxymethyl (58.00548 Da), M4-Oxidation (15.99492 Da), Y6-Oxidation (15.99492 Da)
Charge: +2, Monoisotopic m/z: 952.41101 Da (-4.59 mmu/-4.82 ppm), MH+: 1903.81474 Da, RT: 44.9288 min,
Identified with: Sequest HT (v1.17); XCorr:0.68, Percolator q-Value:5.0e-3, Percolator PEP:3.1e-2,
Fragment match tolerance used for search: 0.02 Da
Fragments used for search: -H_2O; y; b; b; -H_2O; y

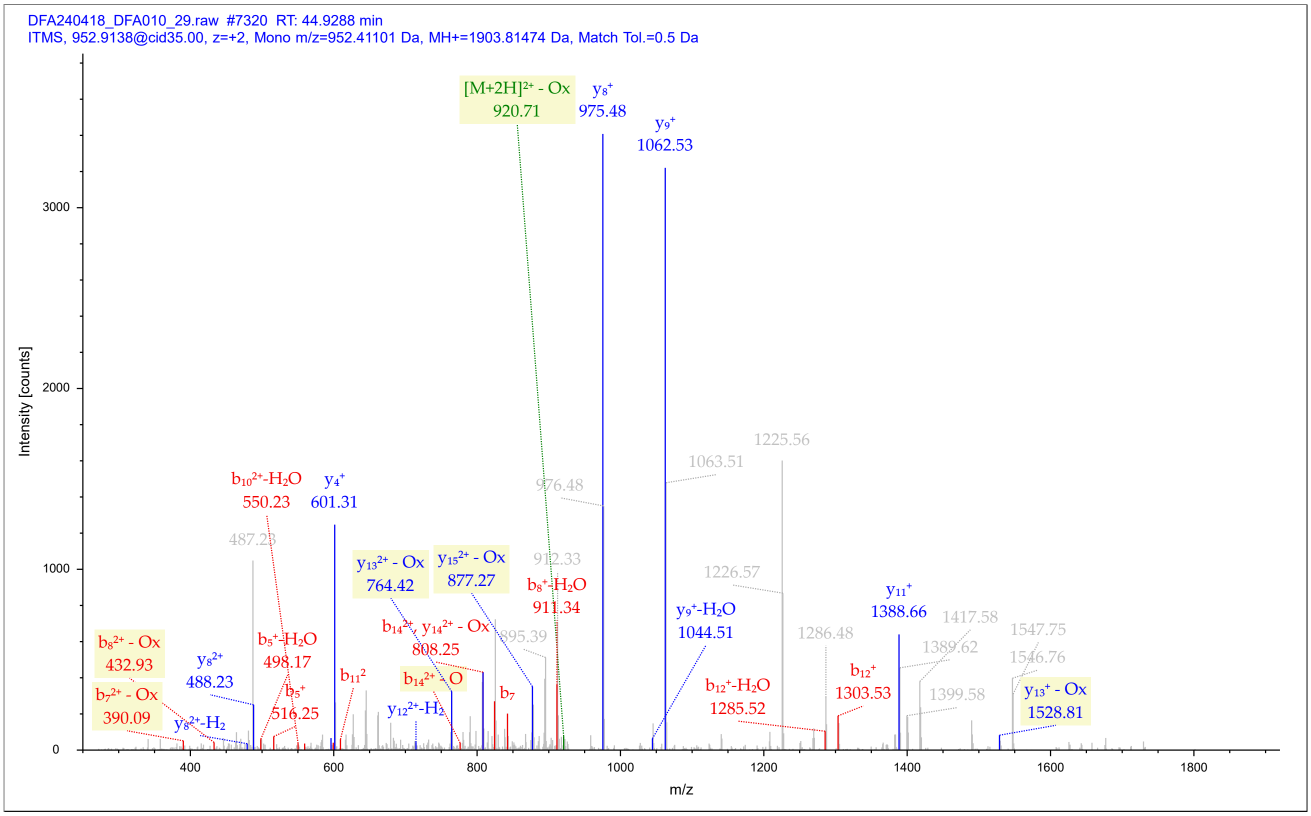

#1	b⁺	b²⁺	Seq.	y⁺	y²⁺	#2
1	88.03930	44.52329	S			16
2	225.09822	113.05275	H	1816.79189	908.89958	15
3	312.13025	156.56876	S	1679.73297	840.37013	14
4	459.16564	230.08646	M-Oxidation	1592.70095	796.85411	13
5	516.18711	258.59719	G	1445.66555	723.33641	12
6	695.24535	348.12631	Y-Oxidation	1388.64408	694.82568	11
7	842.31377	421.66052	F	1209.58584	605.29656	10
8	929.34579	465.17654	S	1062.51743	531.76235	9
9	1030.39347	515.70037	T	975.48540	488.24634	8
10	1117.42550	559.21639	S	874.43772	437.72250	7
11	1216.49391	608.75060	V	787.40569	394.20648	6
12	1303.52594	652.26661	S	688.33728	344.67228	5
13	1517.63253	759.31990	R-Carboxy methyl	601.30525	301.15626	4
14	1614.68530	807.84629	P	387.19866	194.10297	3
15	1671.70676	836.35702	G	290.14589	145.57659	2
16			R-Carboxy methyl	233.12443	117.06585	1

13

equence: AYNILIGELDCSK, C11-Carbamidomethyl (57.02146 Da), Y2-Trioxidation (47.98474 Da), K13-Lederers pentosone (114.03234 Da)
Charge: +3, Monoisotopic m/z: 553.25916 Da (-0.09 mmu/-0.17 ppm), MH+: 1657.76291 Da, RT: 49.0649 min,
Identified with: Sequest HT (v1.17); XCorr:0.72, Percolator q-Value:1.4e-3, Percolator PEP:2.6e-2,
Fragment match tolerance used for search: 0.02 Da
Fragments used for search: -H_2O; y; -NH_3; y; b; b; -H_2O; b; -NH_3; y

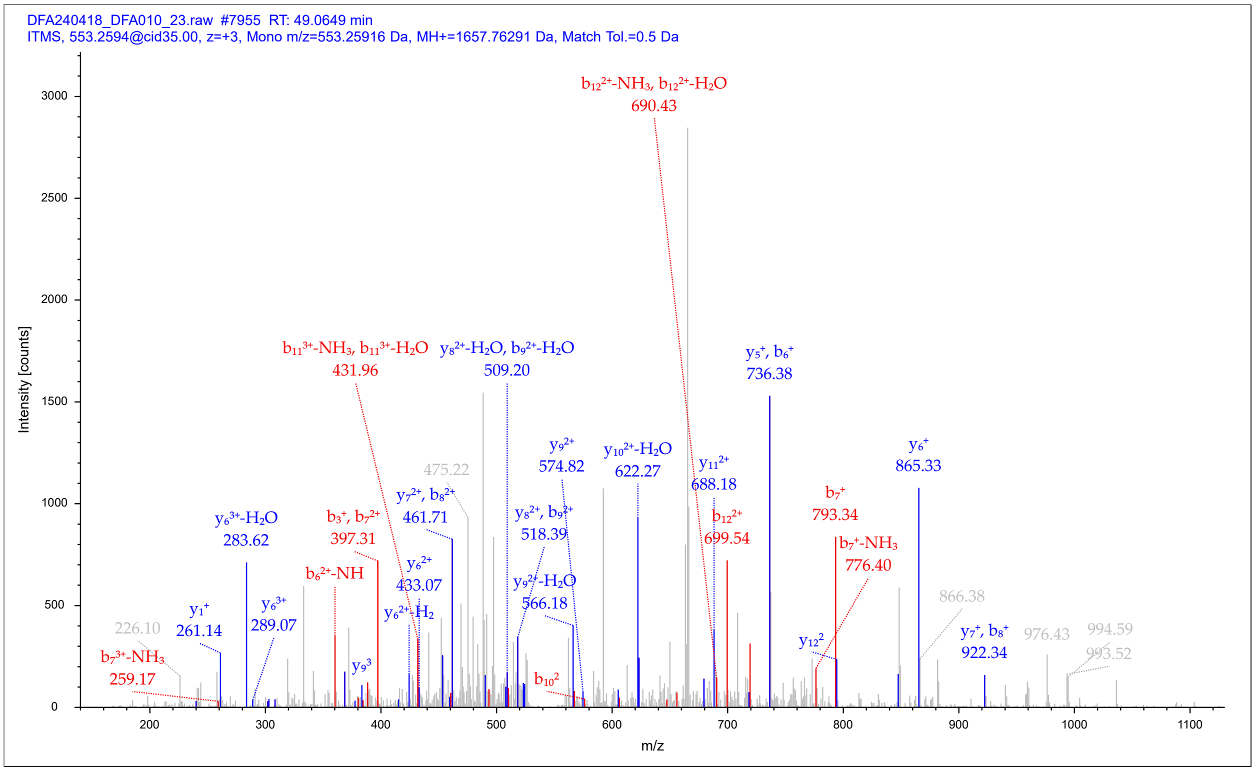

#1	b⁺	b²⁺	b³⁺	Seq.	y⁺	y²⁺	y³⁺	#2
1	72.04439	36.52583	24.68631	A				13
2	283.09246	142.04987	95.03567	Y-Trioxidation	1586.72608	793.86668	529.58021	12
3	397.13539	199.07133	133.04998	N	1375.67800	688.34264	459.23085	11
4	510.21945	255.61337	170.74467	I	1261.63508	631.32118	421.21654	10
5	623.30352	312.15540	208.43936	L	1148.55101	574.77914	383.52186	9
6	736.38758	368.69743	246.13405	I	1035.46695	518.23711	345.82717	8
7	793.40905	397.20816	265.14120	G	922.38288	461.69508	308.13248	7
8	922.45164	461.72946	308.15540	E	865.36142	433.18435	289.12532	6
9	1035.53570	518.27149	345.85009	L	736.31883	368.66305	246.11113	5
10	1150.56265	575.78496	384.19240	D	623.23476	312.12102	208.41644	4
11	1310.59329	655.80029	437.53595	C-Carbamido methyl	508.20782	254.60755	170.07412	3
12	1397.62532	699.31630	466.54663	S	348.17717	174.59222	116.73058	2
13				K-Lederers pentosone	261.14514	131.07621	87.71990	1

Sequence: LEFFDHKGSSSGGGR, R15-Delta:H(2)C(3)O(1) (54.01057 Da)
Charge: +2, Monoisotopic m/z: 817.88165 Da (+0.13 mmu/+0.16 ppm), MH+: 1634.75603 Da, RT: 50.4283 min,
Identified with: Sequest HT (v1.17); XCorr:0.67, Percolator q-Value:7.4e-3, Percolator PEP:4.0e-2,
Fragment match tolerance used for search: 0.02 Da
Fragments used for search: -H_2O; y; -NH_3; y; b; b; -H_2O; b; -NH_3; y

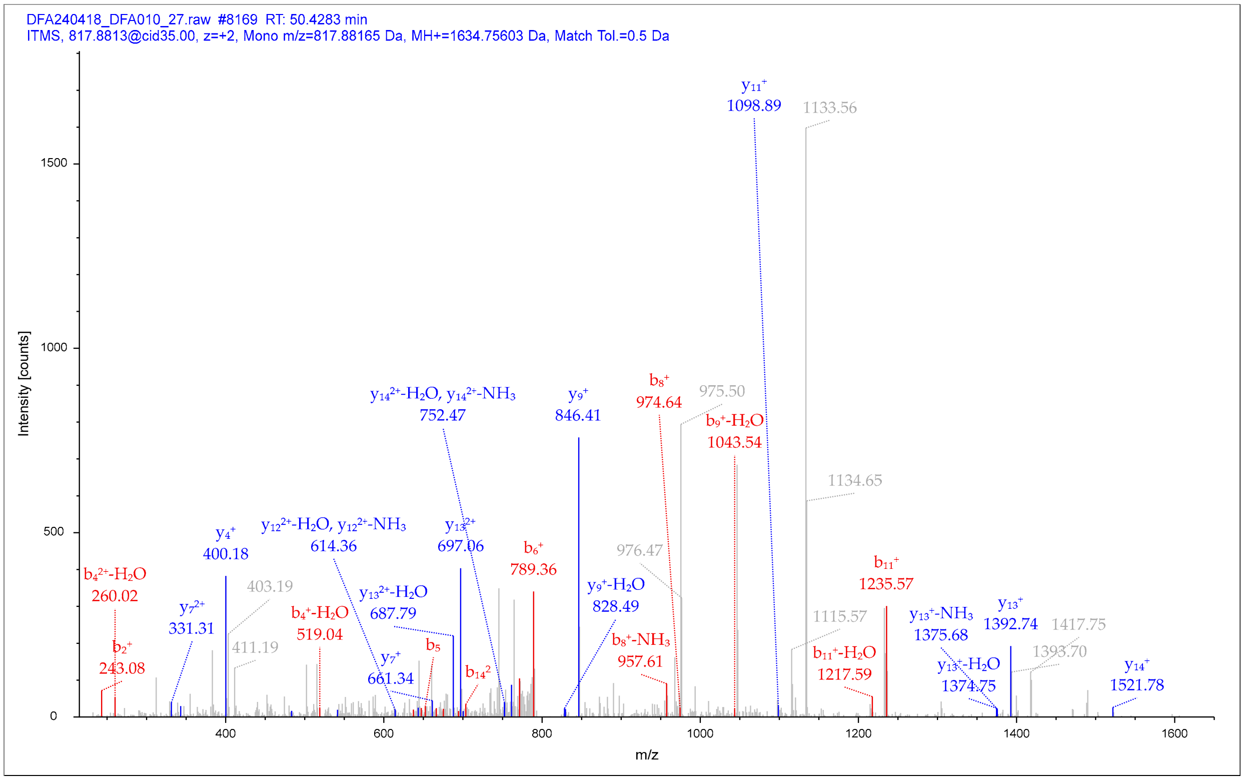

#1	b⁺	b²⁺	Seq.	y⁺	y²⁺	#2
1	114.09134	57.54931	L			15
2	243.13393	122.07061	E	1521.67170	761.33949	14
3	390.20235	195.60481	F	1392.62910	696.81819	13
4	537.27076	269.13902	F	1245.56069	623.28398	12
5	652.29770	326.65249	D	1098.49227	549.74978	11
6	789.35662	395.18195	H	983.46533	492.23630	10
7	917.45158	459.22943	K	846.40642	423.70685	9
8	974.47304	487.74016	G	718.31146	359.65937	8
9	1061.50507	531.25617	S	661.28999	331.14864	7
10	1148.53710	574.77219	S	574.25797	287.63262	6
11	1235.56913	618.28820	S	487.22594	244.11661	5
12	1292.59059	646.79893	G	400.19391	200.60059	4
13	1349.61206	675.30967	G	343.17244	172.08986	3
14	1406.63352	703.82040	G	286.15098	143.57913	2
15			MGH	229.12952	115.06840	1

183

Sequence: GYEENYFFIFR, R11-Carboxyethyl (72.02113 Da), F8-Dioxidation (31.98983 Da), F10-Dioxidation (31.98983 Da)
Charge: +2, Monoisotopic m/z: 810.84668 Da (+0.4 mmu/+0.5 ppm), MH+: 1620.68608 Da, RT: 52.6048 min,
Identified with: Sequest HT (v1.17); XCorr:0.50, Percolator q-Value:5.4e-3, Percolator PEP:1.9e-2,
Fragment match tolerance used for search: 0.02 Da
Fragments used for search: $-H_2O$; y; $-NH_3$; y; b; b; $-H_2O$; b; $-NH_3$; y

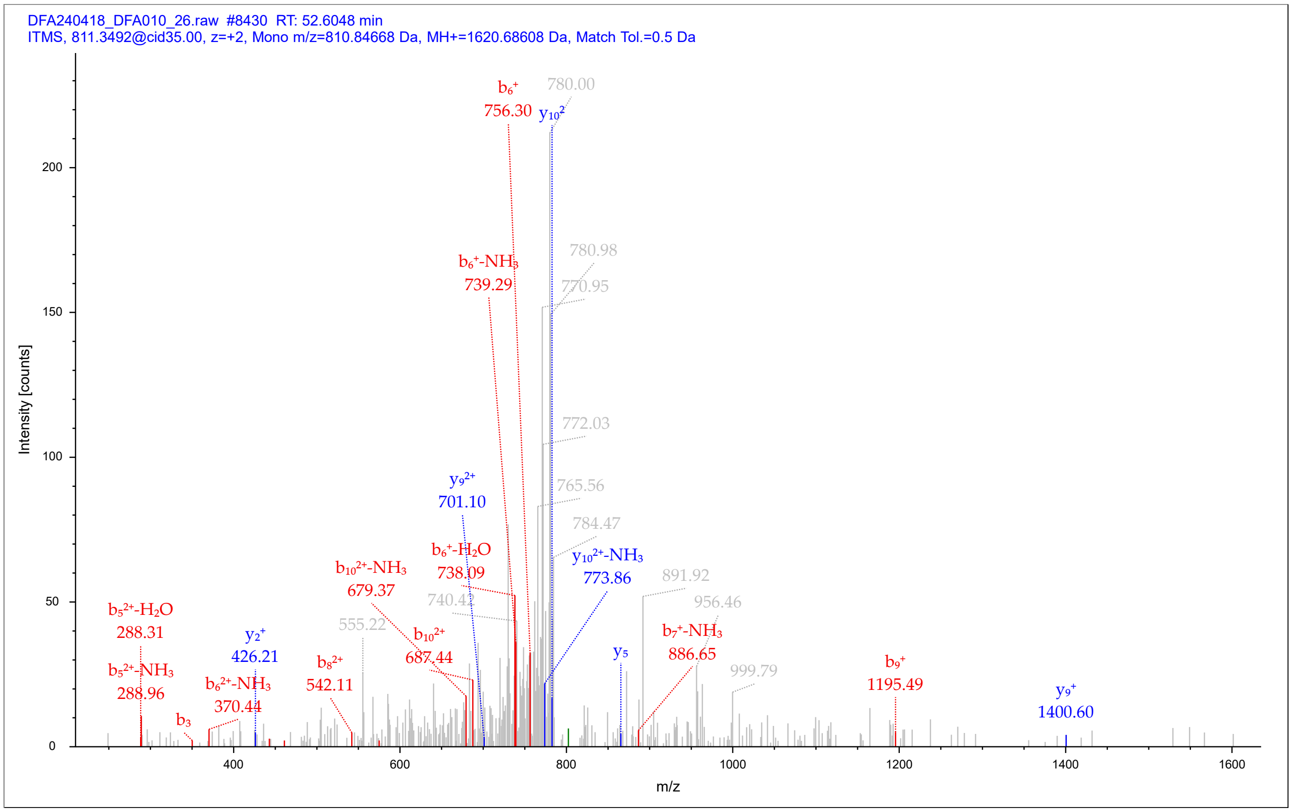

#1	b⁺	b²⁺	Seq.	y⁺	y²⁺	#2
1	58.02874	29.51801	G			11
2	221.09207	111.04967	Y	1563.66382	782.33555	10
3	350.13466	175.57097	E	1400.60049	700.80388	9
4	479.17725	240.09227	E	1271.55789	636.28259	8
5	593.22018	297.11373	N	1142.51530	571.76129	7
6	756.28351	378.64539	Y	1028.47237	514.73982	6
7	903.35192	452.17960	F	865.40904	433.20816	5
8	1082.41017	541.70872	F-Dioxidation	718.34063	359.67395	4
9	1195.49423	598.25075	I	539.28239	270.14483	3
10	1374.55247	687.77988	F-Dioxidation	426.19832	213.60280	2
11			R-Carboxy-ethyl	247.14008	124.07368	1

Sequence: CMELEKYVEK, C1-Carbamidomethyl (57.02146 Da), M2-Oxidation (15.99492 Da), Y7-Oxidation (15.99492 Da), K6-GLAP (109.02805 Da), K10-GLAP (109.02805 Da)
Charge: +2, Monoisotopic m/z: 789.83716 Da (-0.69 mmu/-0.87 ppm), MH+: 1578.66704 Da, RT: 55.7618 min,
Identified with: Sequest HT (v1.17); XCorr:0.61, Percolator q-Value:6.7e-3, Percolator PEP:3.2e-2,
Fragment match tolerance used for search: 0.02 Da
Fragments used for search: -H_2O; y; b; b; -H_2O; y

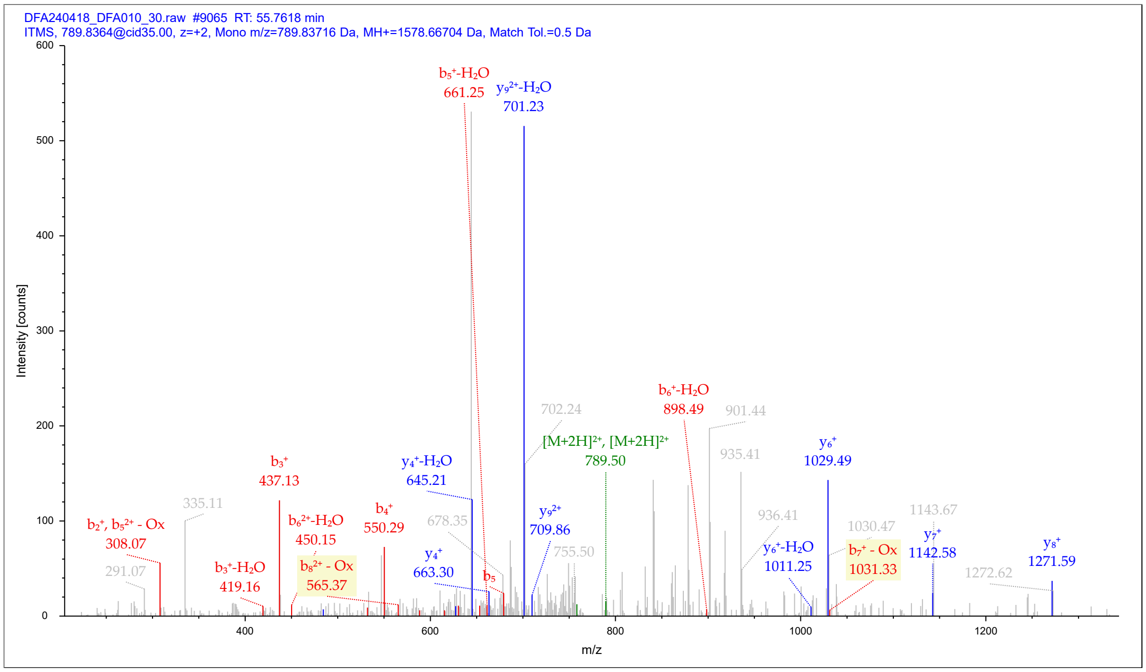

#1	b⁺	b²⁺	Seq.	y⁺	y²⁺	#2
1	161.03793	81.02260	C-Carbamidomethyl			10
2	308.07332	154.54030	M-Oxidation	1418.63777	709.82252	9
3	437.11592	219.06160	E	1271.60237	636.30482	8
4	550.19998	275.60363	L	1142.55977	571.78353	7
5	679.24257	340.12493	E	1029.47571	515.24149	6
6	916.36559	458.68643	K-GLAP	900.43312	450.72020	5
7	1095.42383	548.21555	Y-Oxidation	663.31010	332.15869	4
8	1194.49224	597.74976	V	484.25186	242.62957	3
9	1323.53484	662.27106	E	385.18345	193.09536	2
10			K-GLAP	256.14085	128.57407	1

Sequence: KEGAVLAK, K1-b-ketonium ion (96.02056 Da)
Charge: +2, Monoisotopic m/z: 456.26358 Da (+0.4 mmu/+0.87 ppm), MH+: 911.51988 Da, RT: 69.8435 min,
Identified with: Sequest HT (v1.17); XCorr:1.30, Percolator q-Value:0.0e0, Percolator PEP:1.6e-2,
Fragment match tolerance used for search: 0.02 Da
Fragments used for search: -H_2O; y; -NH_3; y; b; b; -H_2O; y

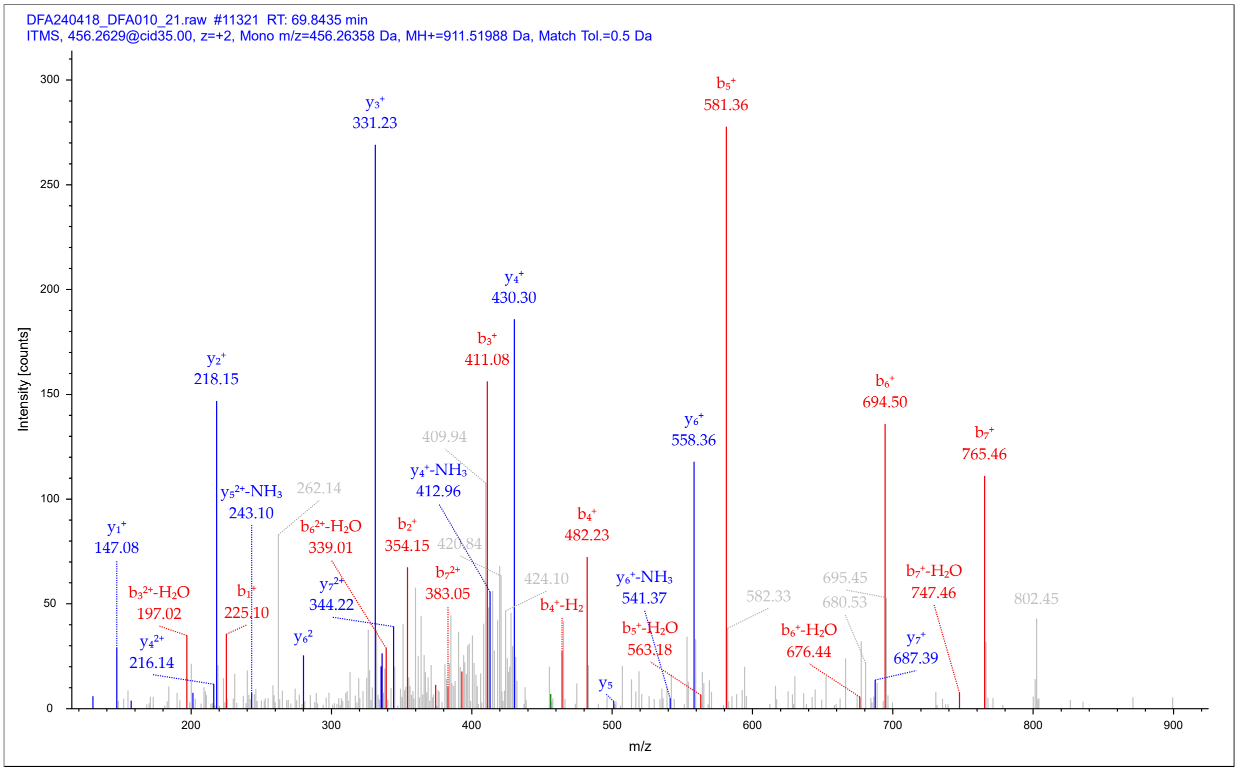

#1	b+	b2+	Seq.	y+	y2+	#2
1	225.12280	113.06504	K-b-keto-nium ion			8
2	354.16539	177.58633	E	687.40357	344.20542	7
3	411.18686	206.09707	G	558.36097	279.68412	6
4	482.22397	241.61562	A	501.33951	251.17339	5
5	581.29238	291.14983	V	430.30240	215.65484	4
6	694.37645	347.69186	L	331.23398	166.12063	3
7	765.41356	383.21042	A	218.14992	109.57860	2
8			K	147.11280	74.06004	1

94_95 (CMX)

Sequence: NMQQSRPFIGMSSAPR, M2-Dioxidation (31.98983 Da), R16-Triosyl (72.01840 Da)
Charge: +2, Monoisotopic m/z: 955.94000 Da (-4.64 mmu/-4.86 ppm), MH+: 1910.87273 Da, RT: 70.6776 min,
Identified with: Sequest HT (v1.17); XCorr:1.08, Percolator q-Value:0.0e0, Percolator PEP:9.4e-3, ptmRS: Best Site Probabilities:M2(Dioxidation): 100,
Fragment match tolerance used for search: 0.02 Da
Fragments used for search: -H_2O; y; -NH_3; y; b; b; -H_2O; b; -NH_3; y

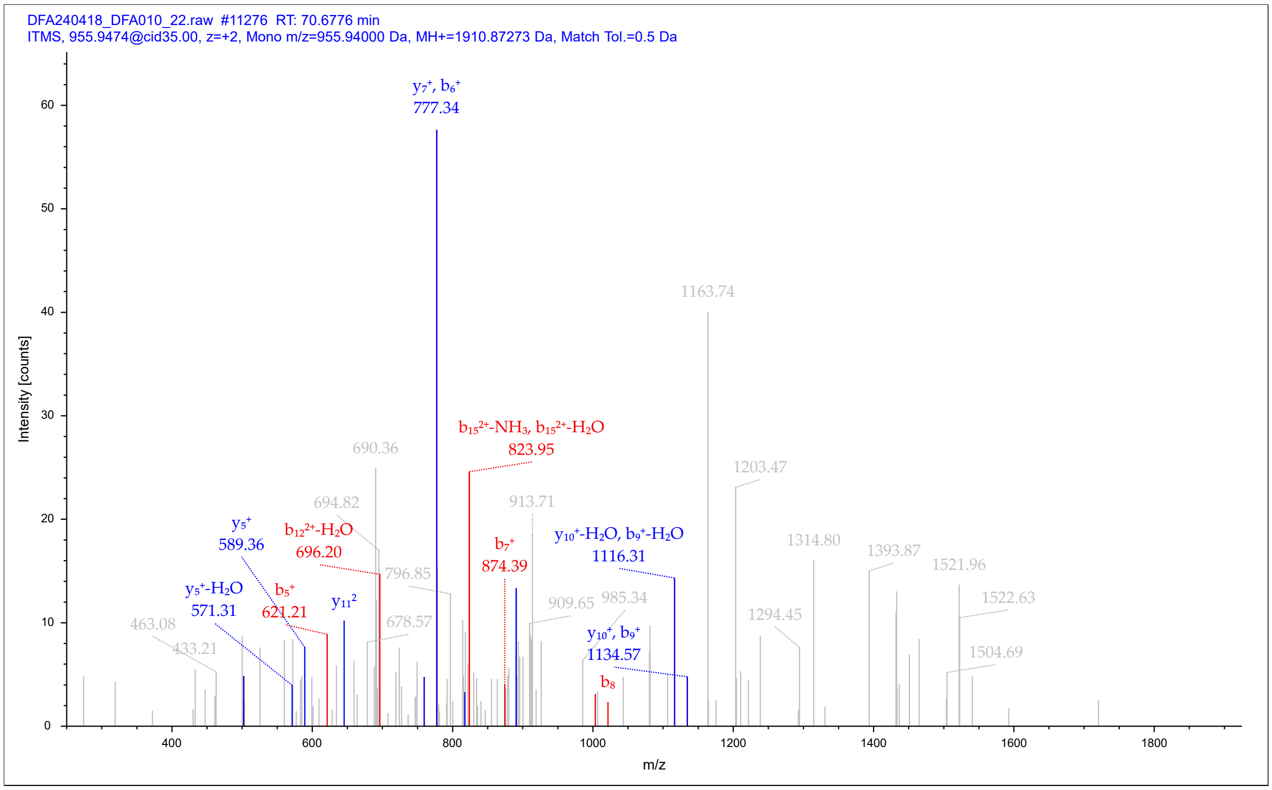

#1	b⁺	b²⁺	Seq.	y⁺	y²⁺	#2
1	115.05020	58.02874	N			16
2	278.08052	139.54390	M-Dioxidation	1796.83908	898.92318	15
3	406.13910	203.57319	Q	1633.80877	817.40802	14
4	534.19767	267.60247	Q	1505.75019	753.37873	13
5	621.22970	311.11849	S	1377.69162	689.34945	12
6	777.33081	389.16904	R	1290.65959	645.83343	11
7	874.38358	437.69543	P	1134.55848	567.78288	10
8	1021.45199	511.22963	F	1037.50571	519.25649	9
9	1134.53605	567.77167	I	890.43730	445.72229	8
10	1191.55752	596.28240	G	777.35323	389.18026	7
11	1322.59800	661.80264	M	720.33177	360.66952	6
12	1409.63003	705.31865	S	589.29129	295.14928	5
13	1496.66206	748.83467	S	502.25926	251.63327	4
14	1567.69917	784.35322	A	415.22723	208.11725	3
15	1664.75194	832.87961	P	344.19012	172.59870	2
16			R-Triosyl	247.13735	124.07231	1

94_95 (CEX)

Sequence: NMQQSRPFIGMSSAPR, R16-Carboxyethyl (72.02113 Da), M2-Dioxidation (31.98983 Da)
Charge: +2, Monoisotopic m/z: 955.94000 Da (-6.01 mmu/-6.29 ppm), MH+: 1910.87273 Da, RT: 70.6776 min,
Identified with: Sequest HT (v1.17); XCorr:1.08, Percolator q-Value:3.9e-3, Percolator PEP:6.9e-3, ptmRS: Best Site Probabilities:M2(Dioxidation): 100,
Fragment match tolerance used for search: 0.02 Da
Fragments used for search: -H_2O; y; -NH_3; y; b; b; -H_2O; b; -NH_3; y

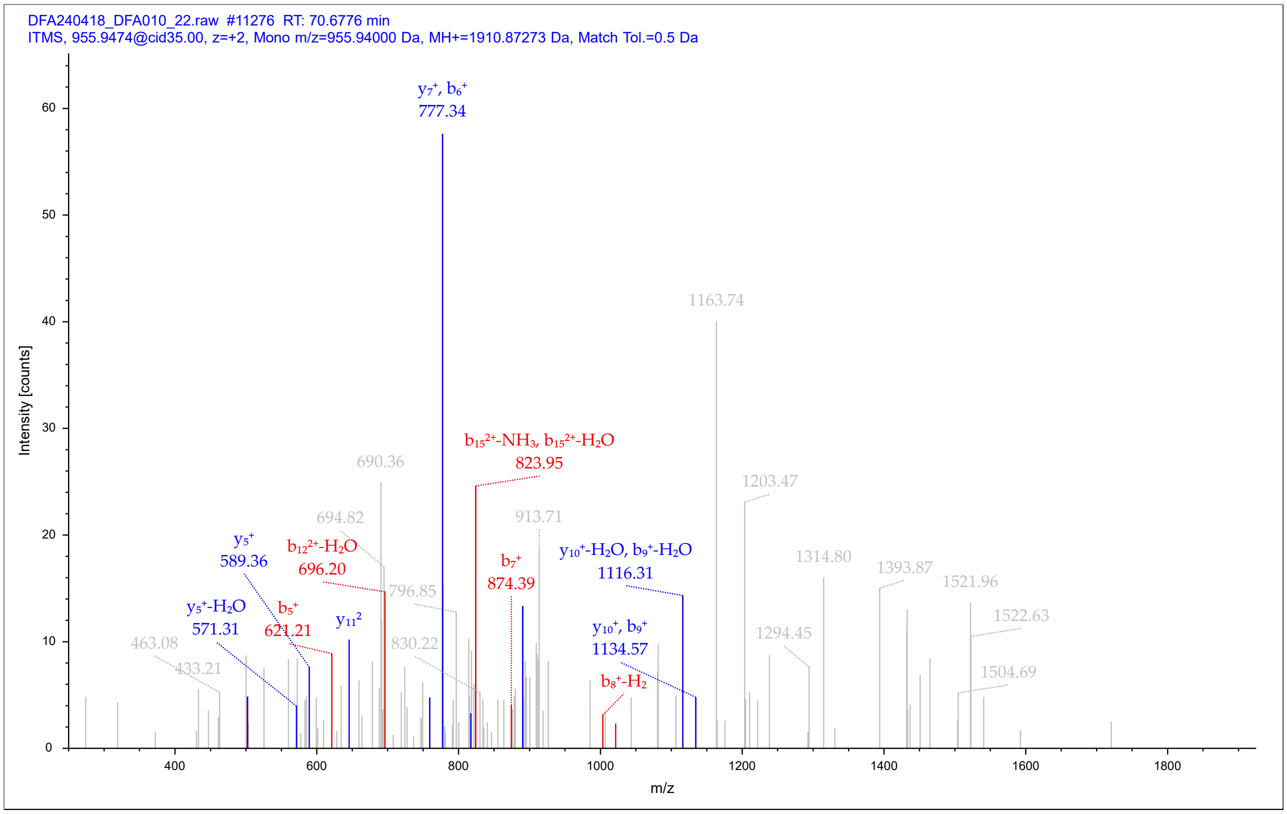

#1	b⁺	b²⁺	Seq.	y⁺	y²⁺	#2
1	115.05020	58.02874	N			16
2	278.08052	139.54390	M-Dioxidation	1796.84181	898.92454	15
3	406.13910	203.57319	Q	1633.81150	817.40939	14
4	534.19767	267.60247	Q	1505.75292	753.38010	13
5	621.22970	311.11849	S	1377.69434	689.35081	12
6	777.33081	389.16904	R	1290.66232	645.83480	11
7	874.38358	437.69543	P	1134.56121	567.78424	10
8	1021.45199	511.22963	F	1037.50844	519.25786	9
9	1134.53605	567.77167	I	890.44003	445.72365	8
10	1191.55752	596.28240	G	777.35596	389.18162	7
11	1322.59800	661.80264	M	720.33450	360.67089	6
12	1409.63003	705.31865	S	589.29402	295.15065	5
13	1496.66206	748.83467	S	502.26199	251.63463	4
14	1567.69917	784.35322	A	415.22996	208.11862	3
15	1664.75194	832.87961	P	344.19285	172.60006	2
16			R-Carboxy-ethyl	247.14008	124.07368	1

Sequence: DASGVTFTWTPSSGK, W9-Trp->Kynurenin (3.99492 Da)
Charge: +2, Monoisotopic m/z: 772.86560 Da (+0.6 mmu/+0.77 ppm), MH+: 1544.72392 Da, RT: 70.8705 min,
Identified with: Sequest HT (v1.17); XCorr:1.08, Percolator q-Value:0.0e0, Percolator PEP:1.6e-3,
Fragment match tolerance used for search: 0.02 Da
Fragments used for search: -H_2O; y; -NH_3; y; b; b; -H_2O; y

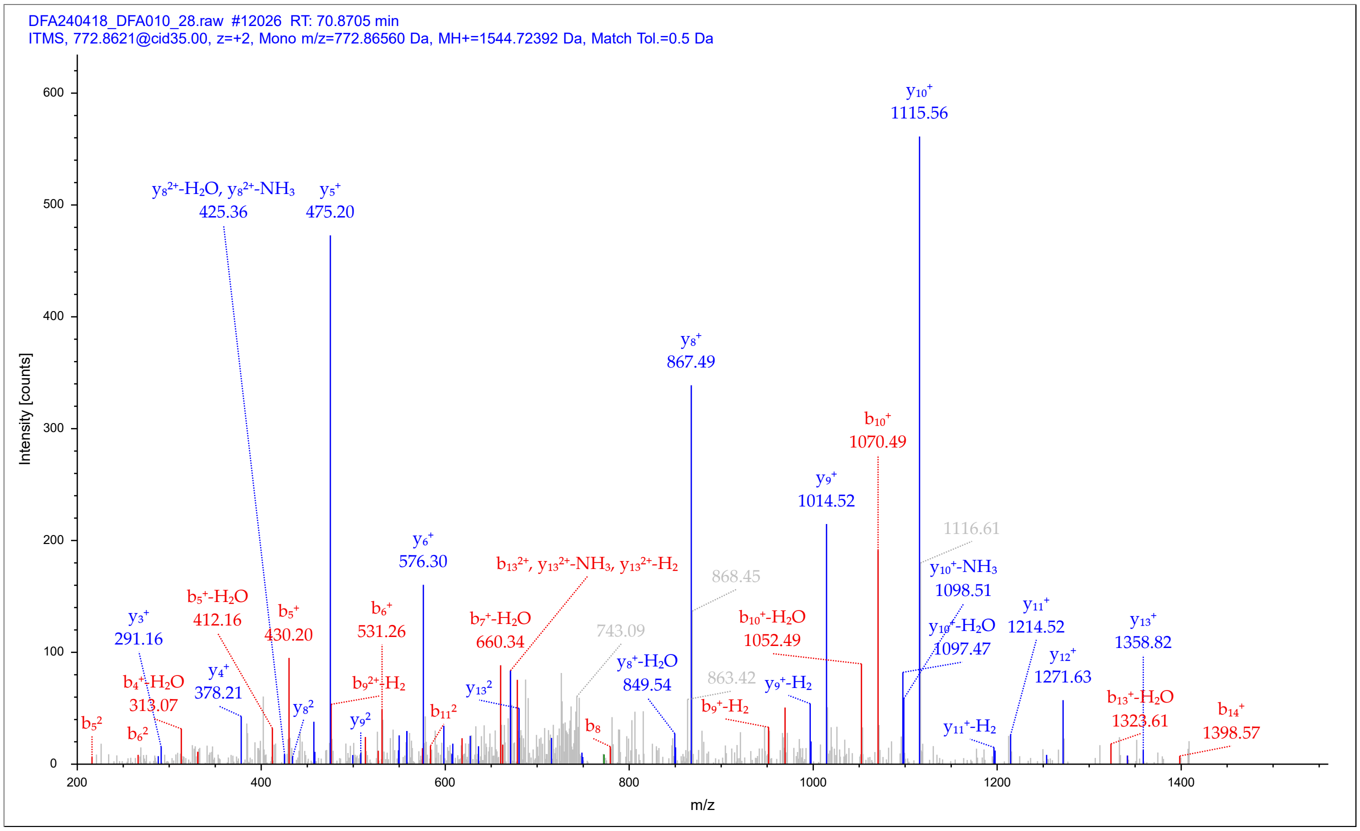

#1	b⁺	b²⁺	Seq.	y⁺	y²⁺	#2
1	116.03422	58.52075	D			15
2	187.07133	94.03930	A	1429.69579	715.35153	14
3	274.10336	137.55532	S	1358.65867	679.83297	13
4	331.12483	166.06605	G	1271.62664	636.31696	12
5	430.19324	215.60026	V	1214.60518	607.80623	11
6	531.24092	266.12410	T	1115.53677	558.27202	10
7	678.30933	339.65830	F	1014.48909	507.74818	9
8	779.35701	390.18214	T	867.42067	434.21397	8
9	969.43124	485.21926	W-Trp->Kynu-renin	766.37299	383.69014	7
10	1070.47892	535.74310	T	576.29877	288.65302	6
11	1167.53168	584.26948	P	475.25109	238.12918	5
12	1254.56371	627.78549	S	378.19832	189.60280	4
13	1341.59574	671.30151	S	291.16630	146.08679	3
14	1398.61720	699.81224	G	204.13427	102.57077	2
15			K	147.11280	74.06004	1

Sequence: MNLLPNIESPVTRQEK, M1-Trioxidation (47.98474 Da), R13-Argpyrimidine (80.02620 Da)
Charge: +2, Monoisotopic m/z: 998.99841 Da (-5.63 mmu/-5.63 ppm), MH+: 1996.98955 Da, RT: 77.5115 min,
Identified with: Sequest HT (v1.17); XCorr:0.59, Percolator q-Value:3.8e-3, Percolator PEP:2.1e-2,
Fragment match tolerance used for search: 0.02 Da
Fragments used for search: $-H_2O$; y; $-NH_3$; y; b; b; $-H_2O$; b; $-NH_3$; y

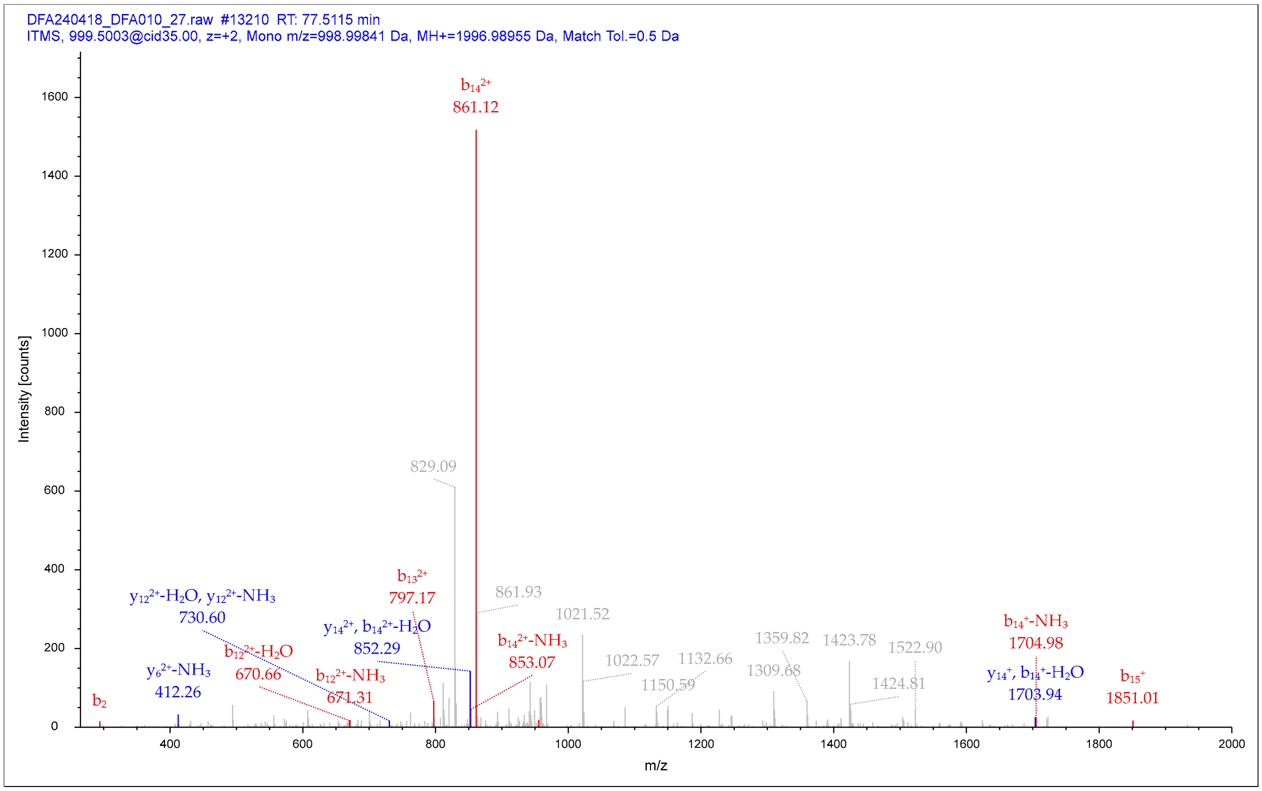

#1	b⁺	b²⁺	Seq.	y⁺	y²⁺	#2
1	180.03251	90.51989	M-Trioxidation			16
2	294.07543	147.54135	N	1817.97557	909.49143	15
3	407.15950	204.08339	L	1703.93265	852.46996	14
4	520.24356	260.62542	L	1590.84858	795.92793	13
5	617.29632	309.15180	P	1477.76452	739.38590	12
6	731.33925	366.17326	N	1380.71175	690.85952	11
7	844.42332	422.71530	I	1266.66883	633.83805	10
8	973.46591	487.23659	E	1153.58476	577.29602	9
9	1060.49794	530.75261	S	1024.54217	512.77472	8
10	1157.55070	579.27899	P	937.51014	469.25871	7
11	1256.61911	628.81320	V	840.45738	420.73233	6
12	1357.66679	679.33703	T	741.38896	371.19812	5
13	1593.79410	797.40069	R-Argpyrimidine	640.34129	320.67428	4
14	1721.85268	861.42998	Q	404.21397	202.61063	3
15	1850.89527	925.95128	E	276.15540	138.58134	2
16			K	147.11280	74.06004	1

Sequence: YAASSYLSLTPEQWR, W14-Trp->Kynurenin (3.99492 Da)
Charge: +2, Monoisotopic m/z: 888.42609 Da (-7.5 mmu/-8.44 ppm), MH+: 1775.84490 Da, RT: 80.3702 min,
Identified with: Sequest HT (v1.17); XCorr:0.76, Percolator q-Value:3.7e-2, Percolator PEP:1.1e-1,
Fragment match tolerance used for search: 0.02 Da
Fragments used for search: $-H_2O$; y; $-NH_3$; y; b; b; $-H_2O$; b; $-NH_3$; y

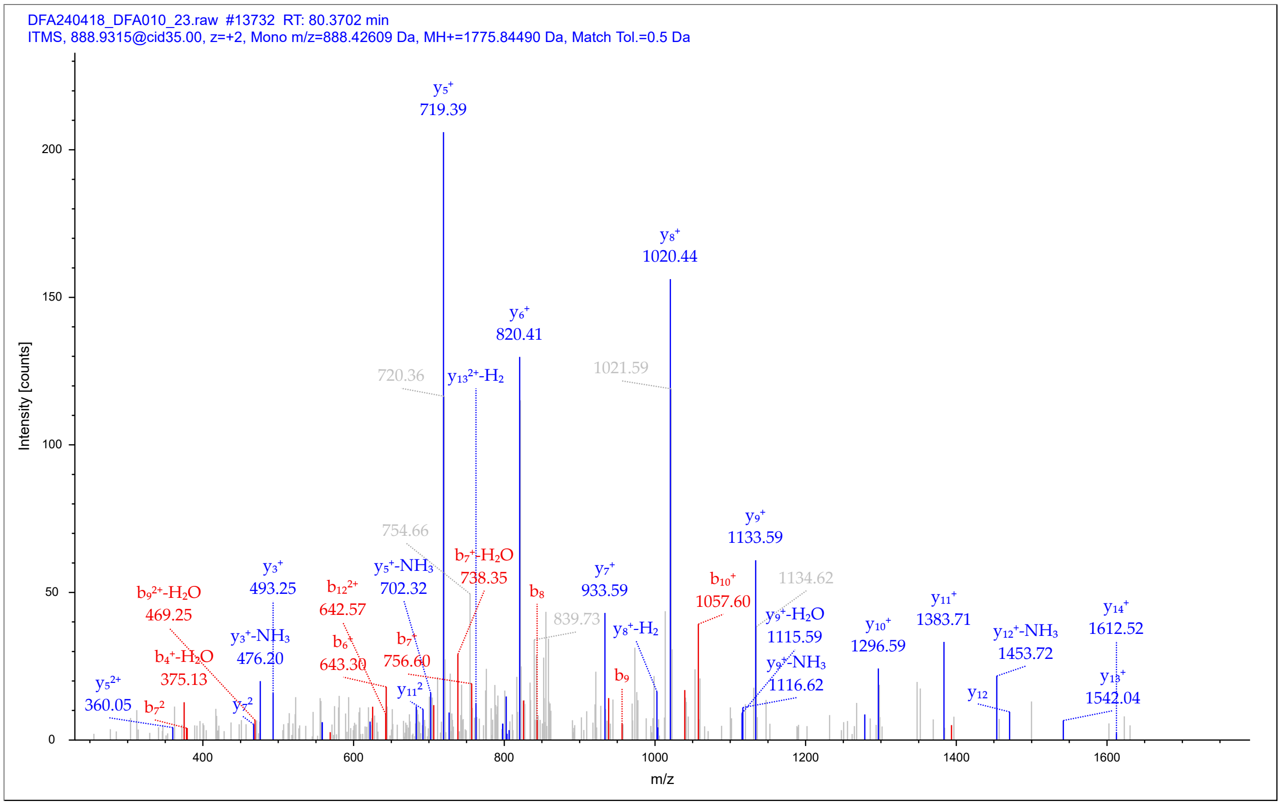

#1	b⁺	b²⁺	Seq.	y⁺	y²⁺	#2
1	164.07061	82.53894	Y			15
2	235.10772	118.05750	A	1612.79656	806.90192	14
3	306.14483	153.57605	A	1541.75945	771.38336	13
4	393.17686	197.09207	S	1470.72233	735.86481	12
5	480.20889	240.60808	S	1383.69031	692.34879	11
6	643.27222	322.13975	Y	1296.65828	648.83278	10
7	756.35628	378.68178	L	1133.59495	567.30111	9
8	843.38831	422.19779	S	1020.51089	510.75908	8
9	956.47237	478.73983	L	933.47886	467.24307	7
10	1057.52005	529.26366	T	820.39479	410.70103	6
11	1154.57282	577.79005	P	719.34711	360.17720	5
12	1283.61541	642.31134	E	622.29435	311.65081	4
13	1411.67399	706.34063	Q	493.25176	247.12952	3
14	1601.74821	801.37775	W-Trp->Kynurenin	365.19318	183.10023	2
15			R	175.11895	88.06311	1

Sequence: SSIPRPRSWALGR, R7-Fructosyl (162.05354 Da), R13-Glarg (39.99949 Da)
Charge: +4, Monoisotopic m/z: 421.97519 Da (-0.73 mmu/-1.73 ppm), MH+: 1684.87893 Da, RT: 29.5472 min,
Identified with: Sequest HT (v1.17); XCorr:0.87, Percolator q-Value:4.0e-3, Percolator PEP:2.8e-2,
Fragment match tolerance used for search: 0.02 Da
Fragments used for search: -H_2O; y; -NH_3; y; b; b; -H_2O; b; -NH_3; y

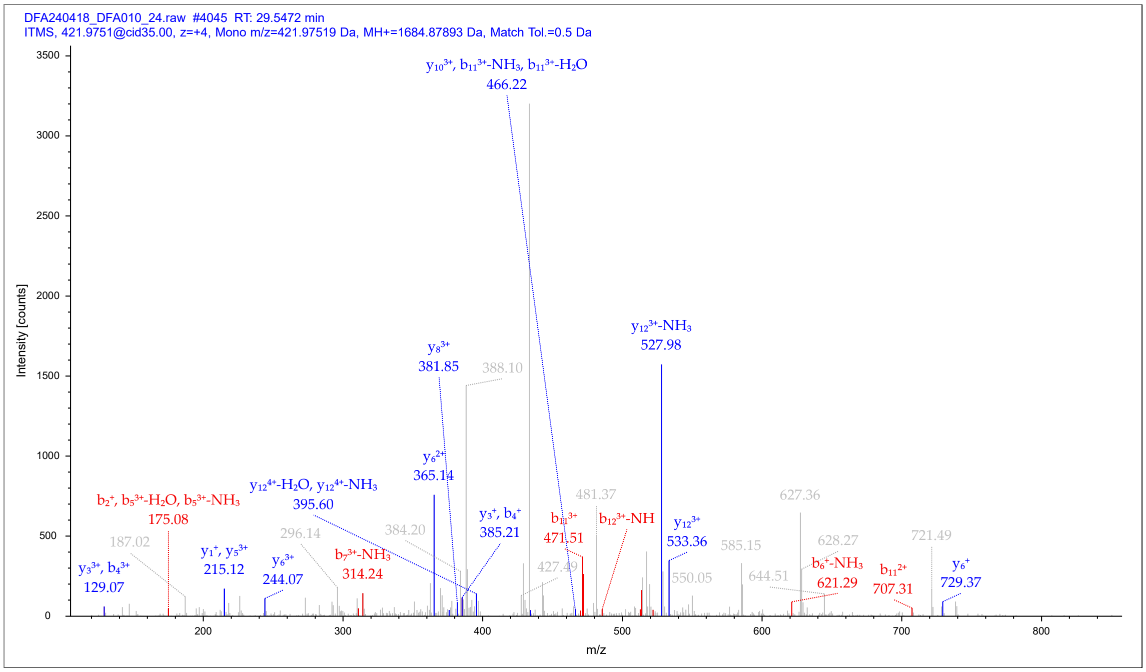

#1	b⁺	b²⁺	b³⁺	b⁴⁺	Seq.	y⁺	y²⁺	y³⁺	y⁴⁺	#2
1	88.03930	44.52329	30.01795	22.76528	S					13
2	175.07133	88.03930	59.02863	44.52329	S	1597.84981	799.42854	533.28812	400.21791	12
3	288.15540	144.58134	96.72332	72.79431	I	1510.81778	755.91253	504.27744	378.45990	11
4	385.20816	193.10772	129.07424	97.05750	P	1397.73372	699.37050	466.58276	350.18889	10
5	541.30927	271.15827	181.10794	136.08278	R	1300.68095	650.84411	434.23184	325.92570	9
6	638.36204	319.68466	213.45886	160.34597	P	1144.57984	572.79356	382.19813	286.90042	8
7	956.51669	478.76198	319.51041	239.88463	R-Fructosyl	1047.52708	524.26718	349.84721	262.63723	7
8	1043.54872	522.27800	348.52109	261.64264	S	729.37243	365.18985	243.79566	183.09856	6
9	1229.62803	615.31765	410.54753	308.16246	W	642.34040	321.67384	214.78498	161.34056	5
10	1300.66514	650.83621	434.22657	325.92174	A	456.26109	228.63418	152.75855	114.82073	4
11	1413.74921	707.37824	471.92125	354.19276	L	385.22397	193.11562	129.07951	97.06145	3
12	1470.77067	735.88897	490.92841	368.44812	G	272.13991	136.57359	91.38482	68.79043	2
13					R-Glarg	215.11844	108.06286	72.37767	54.53507	1

Sequence: WPQSNPKFLCADYAEQDELDYHR, C10-Carbamidomethyl (57.02146 Da), F8-Oxidation (15.99492 Da), W1-Trioxidation (47.98474 Da), K7-Carboxyethyl (72.02113 Da)
Charge: +3, Monoisotopic m/z: 1006.76776 Da (+1.36 mmu/+1.35 ppm), MH+: 3018.28873 Da, RT: 41.0523 min, Identified with: Sequest HT (v1.17); XCorr:1.37, Percolator q-Value:3.9e-3, Percolator PEP:3.6e-2, ptmRS: Best Site Probabilities:K7(Carboxyethyl): 100,
Fragment match tolerance used for search: 0.02 Da
Fragments used for search: $-H_2O$; y; $-NH_3$; y; b; b; $-H_2O$; b; $-NH_3$; y

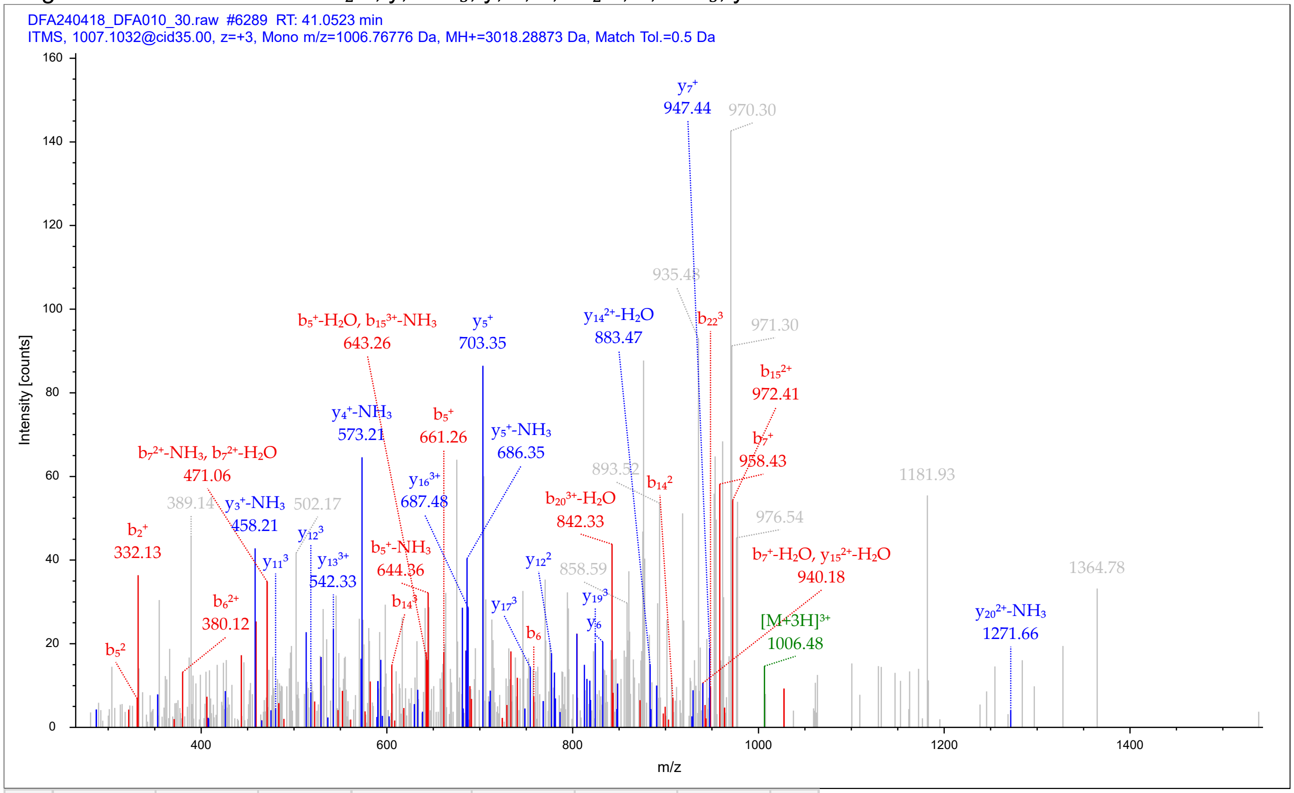

#1	b+	b2+	b3+	Seq.	y+	y2+	y3+	#2
1	235.07133	118.03931	79.02863	W-Trioxidation				23
2	332.12410	166.56569	111.37955	P	2784.22060	1392.61394	928.74505	22
3	460.18267	230.59498	154.06574	Q	2687.16783	1344.08756	896.39413	21
4	547.21470	274.11099	183.07642	S	2559.10926	1280.05827	853.70794	20
5	661.25763	331.13245	221.09073	N	2472.07723	1236.54225	824.69726	19
6	758.31039	379.65884	253.44165	P	2358.03430	1179.52079	786.68295	18
7	958.42649	479.71688	320.14701	K-Carboxy ethyl	2260.98154	1130.99441	754.33203	17
8	1121.48982	561.24855	374.50146	F-Oxidation	2060.86545	1030.93636	687.62667	16
9	1234.57388	617.79058	412.19614	L	1897.80212	949.40470	633.27222	15
10	1394.60453	697.80590	465.53969	C-Carbamido methyl	1784.71805	892.86266	595.57754	14
11	1465.64164	733.32446	489.21873	A	1624.68740	812.84734	542.23399	13
12	1580.66858	790.83793	527.56105	D	1553.65029	777.32878	518.55495	12
13	1743.73191	872.36959	581.91549	Y	1438.62335	719.81531	480.21263	11
14	1814.76903	907.88815	605.59453	A	1275.56002	638.28365	425.85819	10
15	1943.81162	972.40945	648.60872	E	1204.52291	602.76509	402.17915	9
16	2071.87020	1036.43874	691.29492	Q	1075.48031	538.24379	359.16496	8
17	2186.89714	1093.95221	729.63723	D	947.42174	474.21451	316.47876	7
18	2315.93973	1158.47350	772.65143	E	832.39479	416.70103	278.13645	6
19	2429.02380	1215.01554	810.34612	L	703.35220	352.17974	235.12225	5
20	2544.05074	1272.52901	848.68843	D	590.26814	295.63771	197.42756	4
21	2707.11407	1354.06067	903.04287	Y	475.24119	238.12423	159.08525	3
22	2844.17298	1422.59013	948.72918	H	312.17786	156.59257	104.73081	2
23				R	175.11895	88.06311	59.04450	1

19

Sequence: DGLTPVPPLAPAPRPPSSGLPAVLNPRPTLTPGR, R34-Glarg (39.99949 Da)

Charge: +6, Monoisotopic m/z: 575.65363 Da (-4.26 mmu/-7.41 ppm), MH+: 3448.88537 Da, RT: 33.9151 min,

Identified with: Sequest HT (v1.17); XCorr:0.92, Percolator q-Value:1.5e-3, Percolator PEP:1.5e-2,

Fragment match tolerance used for search: 0.02 Da

Fragments used for search: $-H_2O$; y; $-NH_3$; y; b; b; $-H_2O$; b; $-NH_3$; y

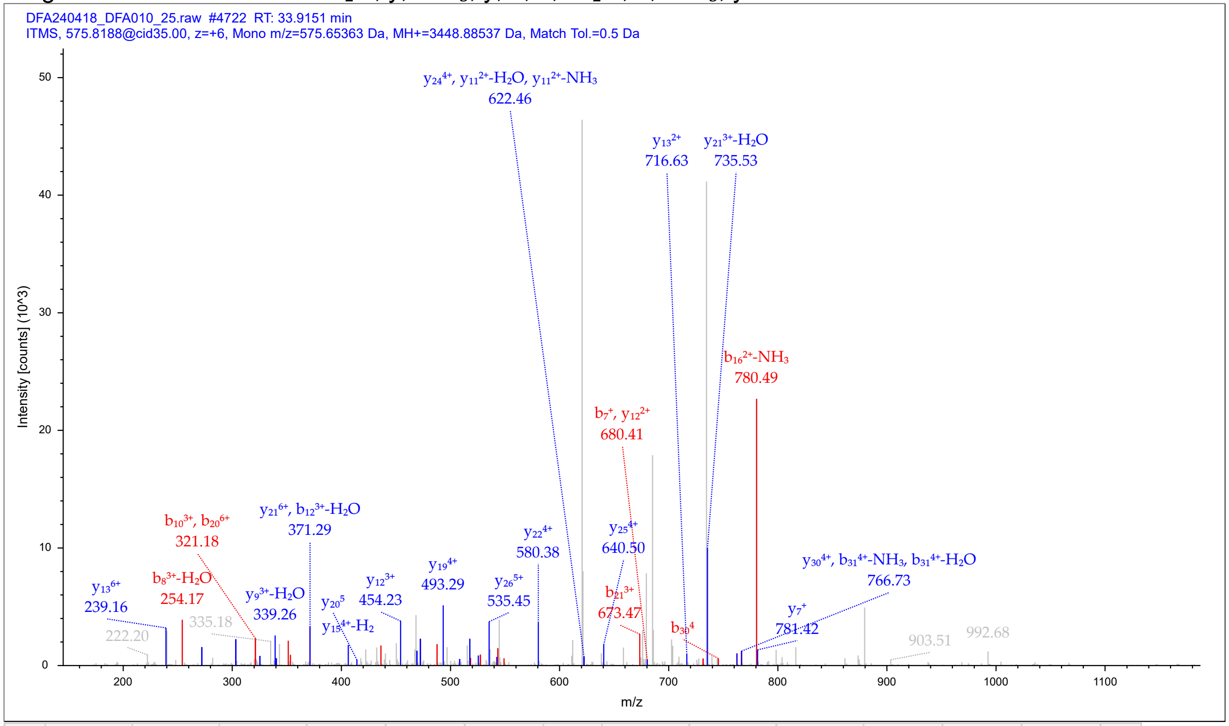

#1	b⁺	b²⁺	b³⁺	b⁴⁺	b⁵⁺	b⁶⁺	Seq.	y⁺	y²⁺	y³⁺	y⁴⁺	y⁵⁺	y⁶⁺	#2
1	116.03422	58.52075	39.34959	29.76401	24.01267	20.17843	D							34
2	173.05568	87.03148	58.35675	44.01938	35.41696	29.68201	G	3333.88397	1667.44562	1111.96617	834.22645	667.58261	556.48673	33
3	286.13975	143.57351	96.05143	72.29039	58.03377	48.52936	L	3276.86250	1638.93489	1092.95902	819.97108	656.17832	546.98315	32
4	387.18743	194.09735	129.73399	97.55231	78.24331	65.37063	T	3163.77844	1582.39286	1055.26433	791.70007	633.56151	528.13580	31
5	484.24019	242.62373	162.08491	121.81550	97.65386	81.54610	P	3062.73076	1531.86902	1021.58177	766.43815	613.35197	511.29452	30
6	583.30860	292.15794	195.10772	146.58261	117.46754	98.05750	V	2965.67800	1483.34264	989.23085	742.17496	593.94142	495.11906	29
7	680.36137	340.68432	227.45864	170.84580	136.87809	114.23296	P	2866.60958	1433.80843	956.20805	717.40785	574.12774	478.60766	28
8	777.41413	389.21070	259.80956	195.10899	156.28865	130.40842	P	2769.55682	1385.28205	923.85712	693.14466	554.71719	462.43220	27
9	890.49819	445.75274	297.50425	223.38001	178.90546	149.25576	L	2672.50406	1336.75567	891.50620	668.88147	535.30663	446.25674	26
10	961.53531	481.27129	321.18329	241.13928	193.11288	161.09528	A	2559.41999	1280.21363	853.81152	640.61046	512.68982	427.40940	25
11	1058.58807	529.79767	353.53421	265.40248	212.52344	177.27074	P	2488.38288	1244.69508	830.13248	622.85118	498.48240	415.56988	24
12	1129.62519	565.31623	377.21325	283.16175	226.73086	189.11026	A	2391.33011	1196.16870	797.78156	598.58799	479.07184	399.39442	23
13	1226.67795	613.84261	409.56417	307.42494	246.14141	205.28572	P	2320.29300	1160.65014	774.10252	580.82871	464.86442	387.55490	22
14	1382.77906	691.89317	461.59787	346.45022	277.36163	231.30257	R	2223.24024	1112.12376	741.75160	556.56552	445.45387	371.37944	21
15	1479.83182	740.41955	493.94879	370.71341	296.77219	247.47803	P	2067.13913	1034.07320	689.71789	517.54024	414.23365	345.36258	20
16	1576.88459	788.94593	526.29971	394.97660	316.18274	263.65350	P	1970.08636	985.54682	657.36697	493.27705	394.82309	329.18712	19
17	1663.91662	832.46195	555.31039	416.73461	333.58914	278.15883	S	1873.03360	937.02044	625.01605	469.01386	375.41254	313.01166	18
18	1750.94865	875.97796	584.32107	438.49262	350.99555	292.66417	S	1786.00157	893.50442	596.00537	447.25585	358.00614	298.50633	17
19	1807.97011	904.48869	603.32822	452.74798	362.39984	302.16775	G	1698.96954	849.98841	566.99470	425.49784	340.59973	284.00099	16
20	1921.05417	961.03072	641.02291	481.01900	385.01666	321.01509	L	1641.94808	821.47768	547.98754	411.24248	329.19544	274.49741	15
21	2018.10694	1009.55711	673.37383	505.28219	404.42721	337.19055	P	1528.86401	764.93565	510.29286	382.97146	306.57862	255.65007	14
22	2089.14405	1045.07566	697.05287	523.04147	418.63463	349.03007	A	1431.81125	716.40926	477.94193	358.70827	287.16807	239.47461	13
23	2188.21246	1094.60987	730.07567	547.80857	438.44831	365.54147	V	1360.77414	680.89071	454.26290	340.94899	272.96065	227.63509	12
24	2301.29653	1151.15190	767.77036	576.07959	461.06513	384.38882	L	1261.70572	631.35650	421.24009	316.18189	253.14697	211.12368	11
25	2415.33946	1208.17337	805.78467	604.59032	483.87371	403.39597	N	1148.62166	574.81447	383.54540	287.91087	230.53015	192.27634	10
26	2512.39222	1256.69975	838.13559	628.85351	503.28427	419.57143	P	1034.57873	517.79300	345.53109	259.40014	207.72157	173.26919	9
27	2668.49333	1334.75030	890.16929	667.87879	534.50449	445.58829	R	937.52597	469.26662	313.18017	235.13695	188.31101	157.09373	8
28	2765.54609	1383.27669	922.52022	692.14198	553.91504	461.76375	P	781.42486	391.21607	261.14647	196.11167	157.09079	131.07687	7
29	2866.59377	1433.80052	956.20278	717.40390	574.12458	478.60503	T	684.37209	342.68968	228.79555	171.84848	137.68024	114.90141	6
30	2979.67784	1490.34256	993.89746	745.67492	596.74139	497.45237	L	583.32441	292.16585	195.11299	146.58656	117.47070	98.06013	5
31	3080.72551	1540.86640	1027.58002	770.93684	616.95092	514.29365	T	470.24035	235.62381	157.41830	118.31555	94.85389	79.21279	4
32	3177.77828	1589.39278	1059.93094	795.20003	636.36148	530.46911	P	369.19267	185.09997	123.73574	93.05363	74.64436	62.37151	3
33	3234.79974	1617.90351	1078.93810	809.45539	647.76577	539.97269	G	272.13991	136.57359	91.38482	68.79043	55.23380	46.19605	2
34							R-Glarg	215.11844	108.06286	72.37767	54.53507	43.82951	36.69247	1

Sequence: NEGFFALCKGFWPNWLR, C8-Carbamidomethyl (57.02146 Da), F4-Oxidation (15.99492 Da), W15-Trioxidation (47.98474 Da), W12-Trp->Kynurenin (3.99492 Da), K9-3-deoxyglucosone intermediate 2 (126.03170 Da)
Charge: +5, Monoisotopic m/z: 468.01358 Da (-1.06 mmu/-2.26 ppm), MH+: 2336.03880 Da, RT: 35.3888 min,
Identified with: Sequest HT (v1.17); XCorr:0.93, Percolator q-Value:4.1e-3, Percolator PEP:2.3e-2,
Fragment match tolerance used for search: 0.02 Da
Fragments used for search: -H_2O; y; -NH_3; y; b; b; -H_2O; b; -NH_3; y

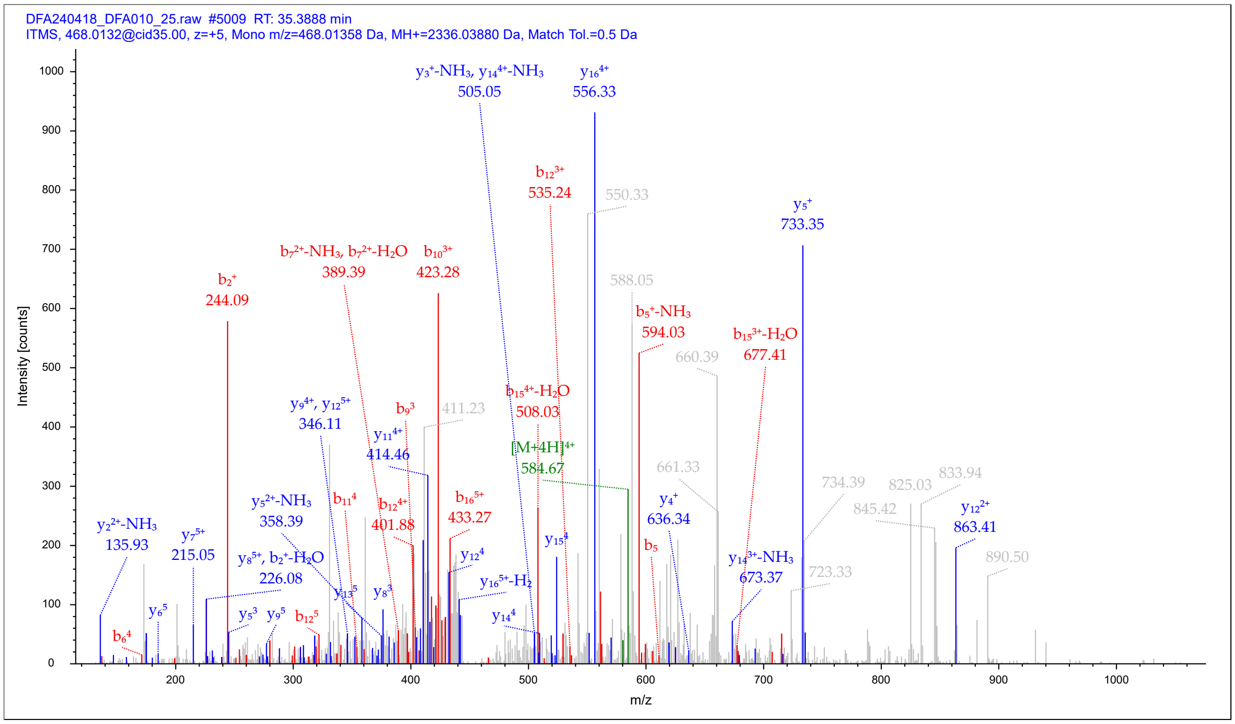

b⁺	b²⁺	b³⁺	b⁴⁺	b⁵⁺	Seq.	y⁺	y²⁺	y³⁺	y⁴⁺	y⁵⁺	#2
115.05020	58.02874	39.02159	29.51801	23.81586	N						17
244.09280	122.55004	82.03578	61.77866	49.62438	E	2222.00116	1111.50422	741.33857	556.25575	445.20605	16
301.11426	151.06077	101.04294	76.03402	61.02867	G	2092.95857	1046.98292	698.32437	523.99510	419.39753	15
464.17759	232.59243	155.39738	116.79985	93.64134	F-Oxidation	2035.93710	1018.47219	679.31722	509.73973	407.99324	14
611.24600	306.12664	204.42019	153.56696	123.05502	F	1872.87377	936.94052	624.96278	468.97390	375.38058	13
682.28312	341.64520	228.09922	171.32624	137.26244	A	1725.80536	863.40632	575.93997	432.20680	345.96689	12
795.36718	398.18723	265.79391	199.59725	159.87926	L	1654.76825	827.88776	552.26093	414.44752	331.75947	11
955.39783	478.20255	319.13746	239.60491	191.88539	C-Carbamido methyl	1541.68418	771.34573	514.56624	386.17650	309.14266	10
1209.52449	605.26588	403.84635	303.13658	242.71072	K-3-deoxyglucosone intermediate 2	1381.65353	691.33040	461.22270	346.16884	277.13653	9
1266.54596	633.77662	422.85350	317.39195	254.11501	G	1127.52687	564.26707	376.51381	282.63717	226.31120	8
1413.61437	707.31082	471.87631	354.15905	283.52870	F	1070.50541	535.75634	357.50665	268.38181	214.90690	7
1603.68860	802.34794	535.23438	401.67761	321.54354	W-Trp-> Kynurenin	923.43699	462.22213	308.48385	231.61471	185.49322	6
1700.74136	850.87432	567.58531	425.94080	340.95409	P	733.36276	367.18502	245.12577	184.09615	147.47837	5
1814.78429	907.89578	605.59961	454.45153	363.76268	N	636.31000	318.65864	212.77485	159.83296	128.06782	4
2048.84835	1024.92781	683.62097	512.96754	410.57549	W-Trioxidation	522.26707	261.63717	174.76054	131.32223	105.25924	3
2161.93241	1081.46984	721.31565	541.23856	433.19230	L	288.20302	144.60515	96.73919	72.80621	58.44642	2
					R	175.11895	88.06311	59.04450	44.53520	35.82961	1

Sequence: DLCTVGTHNSSCPVSCEEIEYPATISYSSFPSQK, C3-Carbamidomethyl (57.02146 Da), C12-Carbamidomethyl (57.02146 Da), C16-Carbamidomethyl (57.02146 Da), K34-Glycerinyl (88.01674 Da)
Charge: +7, Monoisotopic m/z: 563.53558 Da (+0.09 mmu/+0.15 ppm), MH+: 3938.70542 Da, RT: 38.3687 min,
Identified with: Sequest HT (v1.17); XCorr:0.83, Percolator q-Value:2.0e-3, Percolator PEP:8.1e-3,
Fragment match tolerance used for search: 0.02 Da
Fragments used for search: -H_2O; y; -NH_3; y; b; b; -H_2O; b; -NH_3; y

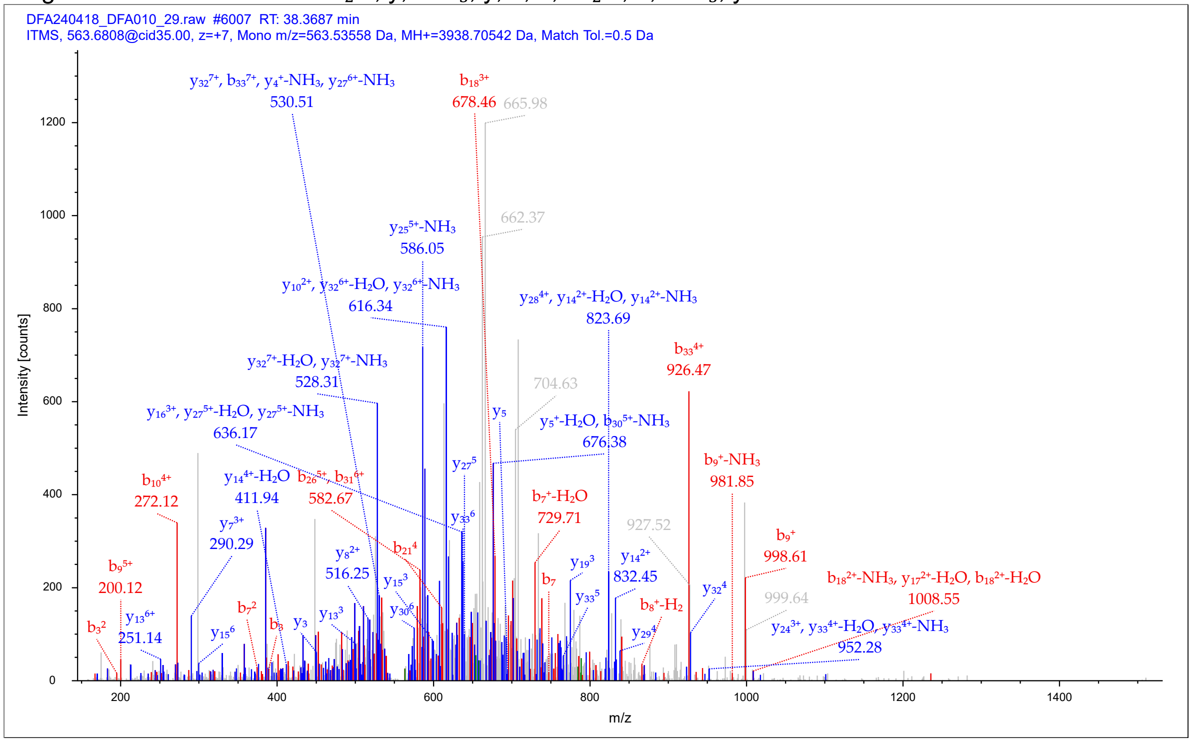

#1	b⁺	b²⁺	b³⁺	b⁴⁺	b⁵⁺	b⁶⁺	b⁷⁺	Seq.	y⁺	y²⁺	y³⁺	y⁴⁺	y⁵⁺	y⁶⁺	y⁷⁺	#2
1	116.03422	58.52075	39.34959	29.76401	24.01267	20.17843	17.43970	D								34
2	229.11828	115.06278	77.04428	58.03503	46.62948	39.02578	33.59456	L	3823.67788	1912.34258	1275.23081	956.67493	765.54140	638.11904	547.10308	33
3	389.14893	195.07810	130.38783	98.04269	78.63561	65.69755	56.45608	C-Carbamido-methyl	3710.59381	1855.80055	1237.53612	928.40391	742.92458	619.27170	530.94821	32
4	490.19661	245.60194	164.07039	123.30461	98.84514	82.53883	70.89147	T	3550.56317	1775.78522	1184.19257	888.39625	710.91845	592.59992	508.08669	31
5	589.26502	295.13615	197.09319	148.07171	118.65883	99.05023	85.04410	V	3449.51549	1725.26138	1150.51001	863.13433	690.70892	575.75865	493.65131	30
6	646.28649	323.64688	216.10035	162.32708	130.06312	108.55381	93.19002	G	3350.44707	1675.72718	1117.48721	838.36723	670.89524	559.24724	479.49868	29
7	747.33417	374.17072	249.78291	187.58900	150.27265	125.39509	107.62540	T	3293.42561	1647.21644	1098.48005	824.11186	659.49094	549.74367	471.35275	28
8	884.39308	442.70018	295.46921	221.85373	177.68444	148.23824	127.20525	H	3192.37793	1596.69260	1064.79749	798.84994	639.28141	532.90239	456.91737	27
9	998.43601	499.72164	333.48352	250.36446	200.49302	167.24540	143.49710	N	3055.31902	1528.16315	1019.11119	764.58521	611.86963	510.05923	437.33753	26
10	1085.46803	543.23766	362.49420	272.12247	217.89943	181.75074	155.93024	S	2941.27609	1471.14168	981.09688	736.07448	589.06104	491.05208	421.04568	25
11	1172.50006	586.75367	391.50487	293.88047	235.30583	196.25607	168.36339	S	2854.24406	1427.62567	952.08621	714.31647	571.65463	476.54674	408.61253	24
12	1332.53071	666.76899	444.84842	333.88814	267.31196	222.92785	191.22491	C-Carbamido-methyl	2767.21204	1384.10966	923.07553	692.55847	554.24823	462.04140	396.17939	23
13	1429.58347	715.29538	477.19934	358.15133	286.72252	239.10331	205.08959	P	2607.18139	1304.09433	869.73198	652.55080	522.24210	435.36963	373.31786	22
14	1528.65189	764.82958	510.22215	382.91843	306.53620	255.61471	219.24222	V	2510.12862	1255.56795	837.38106	628.28761	502.83155	419.19417	359.45318	21
15	1615.68392	808.34560	539.23282	404.67644	323.94260	270.12005	231.67537	S	2411.06021	1206.03374	804.35825	603.52051	483.01786	402.68277	345.30055	20
16	1775.71457	888.36092	592.57637	444.68410	355.94873	296.79182	254.53689	C-Carbamido-methyl	2324.02818	1162.51773	775.34758	581.76250	465.61146	388.17743	332.86741	19
17	1904.75716	952.88222	635.59057	476.94475	381.75725	318.29892	272.97155	E	2163.99753	1082.50240	722.00403	541.75484	433.60533	361.50565	310.00588	18
18	2033.79975	1017.40351	678.60477	509.20540	407.56577	339.80602	291.40620	E	2034.95494	1017.98111	678.98983	509.49419	407.79681	339.99855	291.57123	17
19	2146.88382	1073.94555	716.29946	537.47641	430.18258	358.65337	307.56107	I	1905.91235	953.45981	635.97563	477.23354	381.98829	318.49145	273.13657	16
20	2275.92641	1138.46684	759.31365	569.73706	455.99110	380.16047	325.99572	E	1792.82828	896.91778	598.28095	448.96253	359.37148	299.64411	256.98171	15
21	2438.98974	1219.99851	813.66810	610.50289	488.60377	407.33769	349.29049	Y	1663.78569	832.39648	555.26675	416.70188	333.56296	278.13701	238.54705	14
22	2536.04250	1268.52489	846.01902	634.76608	508.01432	423.51315	363.15517	P	1500.72236	750.86482	500.91230	375.93605	300.95029	250.95979	215.25229	13
23	2607.07961	1304.04345	869.69806	652.52536	522.22174	435.35267	373.30332	A	1403.66960	702.33844	468.56138	351.67286	281.53974	234.78433	201.38761	12
24	2708.12729	1354.56728	903.38062	677.78728	542.43128	452.19395	387.73871	T	1332.63248	666.81988	444.88235	333.91358	267.33232	222.94481	191.23945	11
25	2821.21136	1411.10932	941.07530	706.05830	565.04809	471.04129	403.89357	I	1231.58481	616.29604	411.19979	308.65166	247.12278	206.10353	176.80407	10
26	2908.24338	1454.62533	970.08598	727.81630	582.45450	485.54663	416.32672	S	1118.50074	559.75401	373.50510	280.38064	224.50597	187.25619	160.64920	9
27	3071.30671	1536.15699	1024.44042	768.58214	615.06716	512.72385	439.62148	Y	1031.46871	516.23799	344.49442	258.62264	207.09956	172.75085	148.21605	8
28	3158.33874	1579.67301	1053.45110	790.34014	632.47357	527.22919	452.05463	S	868.40538	434.70633	290.13998	217.85680	174.48690	145.57363	124.92129	7
29	3245.37077	1623.18902	1082.46177	812.09815	649.87998	541.73453	464.48778	S	781.37336	391.19032	261.12930	196.09880	157.08049	131.06829	112.48815	6
30	3392.43918	1696.72323	1131.48458	848.86525	679.29366	566.24593	485.49755	F	694.34133	347.67430	232.11863	174.34079	139.67409	116.56295	100.05500	5
31	3489.49195	1745.24961	1163.83550	873.12844	698.70421	582.42139	499.36223	P	547.27291	274.14010	183.09582	137.57369	110.26040	92.05155	79.04522	4
32	3576.52398	1788.76563	1192.84618	894.88645	716.11062	596.92673	511.79538	S	450.22015	225.61371	150.74490	113.31049	90.84985	75.87609	65.18054	3
33	3704.58255	1852.79492	1235.53237	926.90110	741.72233	618.26982	530.08946	Q	363.18812	182.09770	121.73422	91.55249	73.44345	61.37075	52.74740	2
34								K-Glycerinyl	235.12954	118.06841	79.04803	59.53784	47.83173	40.02765	34.45331	1

Sequence: YVLSYTSSSGDDDFSFYIYHLYWWEFNVHSK, K31-Methyl (14.01565 Da), Y5-Oxidation (15.99492 Da), Y1-Trioxidation (47.98474 Da), W24-Trp->Kynurenin (3.99492 Da)
Charge: +6, Monoisotopic m/z: 657.12524 Da (-0.49 mmu/-0.75 ppm), MH+: 3937.71508 Da, RT: 38.4893 min, Identified with: Sequest HT (v1.17); XCorr:0.65, Percolator q-Value:2.1e-3, Percolator PEP:2.0e-2, ptmRS: Best Site Probabilities:K31(Methyl): 100,
Fragment match tolerance used for search: 0.02 Da
Fragments used for search: -H_2O; y; -NH_3; y; b; b; -H_2O; b; -NH_3; y

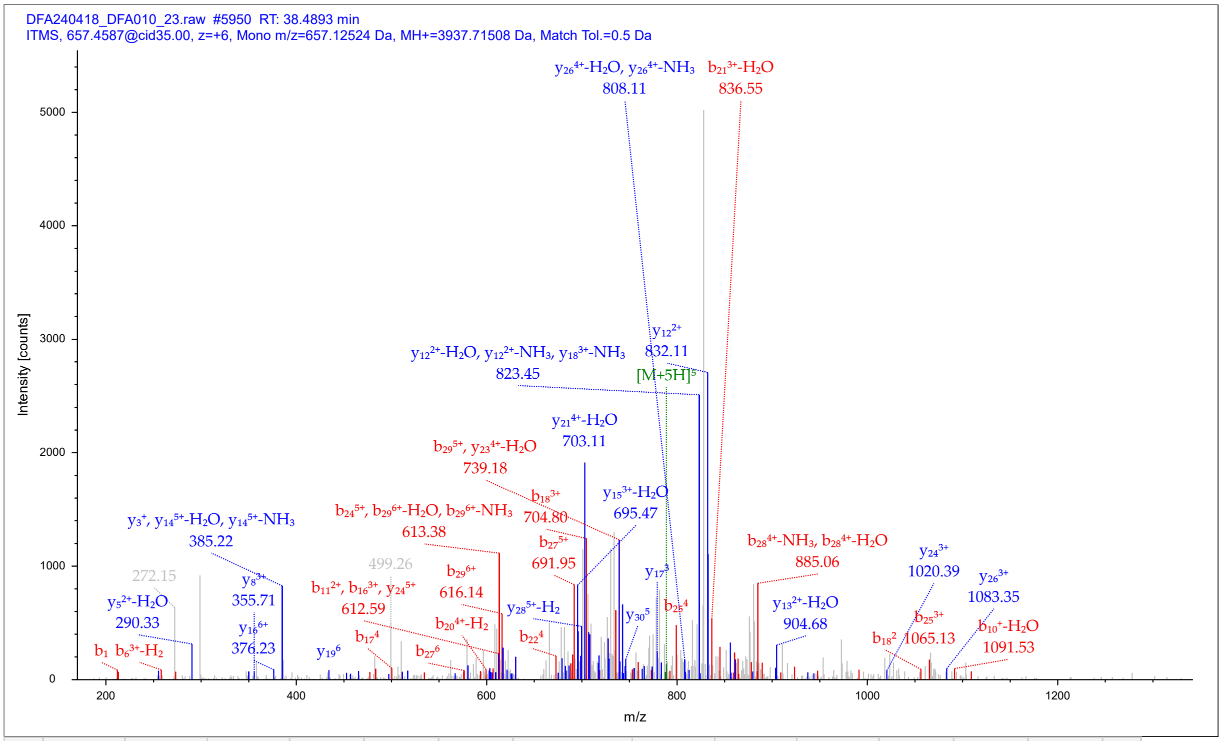

#1	b⁺	b²⁺	b³⁺	b⁴⁺	b⁵⁺	b⁶⁺	Seq.	y⁺	y²⁺	y³⁺	y⁴⁺	y⁵⁺	y⁶⁺	#2
1	212.05535	106.53131	71.35663	53.76929	43.21689	36.18196	Y-Trioxidation							31
2	311.12376	156.06552	104.37944	78.53640	63.03057	52.69336	V	3726.66997	1863.83862	1242.89484	932.42295	746.13982	621.95106	30
3	424.20783	212.60755	142.07413	106.80741	85.64739	71.54070	L	3627.60156	1814.30442	1209.87204	907.65585	726.32613	605.43966	29
4	511.23986	256.12357	171.08480	128.56542	103.05379	86.04604	S	3514.51749	1757.76238	1172.17735	879.38483	703.70932	586.59231	28
5	690.29810	345.65269	230.77088	173.32998	138.86544	115.88908	Y-Oxidation	3427.48546	1714.24637	1143.16667	857.62682	686.30291	572.08697	27
6	791.34578	396.17653	264.45344	198.59190	159.07498	132.73036	T	3248.42722	1624.71725	1083.48059	812.86226	650.49127	542.24393	26
7	878.37781	439.69254	293.46412	220.34991	176.48138	147.23570	S	3147.37954	1574.19341	1049.79803	787.60034	630.28173	525.40265	25
8	965.40983	483.20856	322.47480	242.10792	193.88779	161.74104	S	3060.34751	1530.67740	1020.78736	765.84234	612.87532	510.89732	24
9	1052.44186	526.72457	351.48547	263.86592	211.29419	176.24637	S	2973.31549	1487.16138	991.77668	744.08433	595.46892	496.39198	23
10	1109.46333	555.23530	370.49263	278.12129	222.69849	185.74995	G	2886.28346	1443.64537	962.76600	722.32632	578.06251	481.88664	22
11	1224.49027	612.74877	408.83494	306.87802	245.70388	204.92111	D	2829.26199	1415.13463	943.75885	708.07096	566.65822	472.38306	21
12	1339.51721	670.26224	447.17726	335.63476	268.70926	224.09227	D	2714.23505	1357.62116	905.41653	679.31422	543.65283	453.21191	20
13	1454.54415	727.77572	485.51957	364.39150	291.71465	243.26342	D	2599.20811	1300.10769	867.07422	650.55748	520.64744	434.04075	19
14	1601.61257	801.30992	534.54237	401.15860	321.12834	267.77483	F	2484.18116	1242.59422	828.73191	621.80075	497.64205	414.86959	18
15	1688.64460	844.82594	563.55305	422.91661	338.53474	282.28016	S	2337.11275	1169.06001	779.70910	585.03365	468.22837	390.35819	17
16	1835.71301	918.36014	612.57585	459.68371	367.94842	306.79157	F	2250.08072	1125.54400	750.69843	563.27564	450.82197	375.85285	16
17	1998.77634	999.89181	666.93030	500.44954	400.56109	333.96879	Y	2103.01231	1052.00979	701.67562	526.50853	421.40828	351.34145	15
18	2111.86040	1056.43384	704.62499	528.72056	423.17790	352.81613	I	1939.94898	970.47813	647.32118	485.74270	388.79562	324.16423	14
19	2274.92373	1137.96550	758.97943	569.48639	455.79057	379.99335	Y	1826.86492	913.93610	609.62649	457.47169	366.17880	305.31688	13
20	2411.98264	1206.49496	804.66573	603.75112	483.20235	402.83650	H	1663.80159	832.40443	555.27205	416.70585	333.56614	278.13966	12
21	2525.06671	1263.03699	842.36042	632.02213	505.81916	421.68385	L	1526.74268	763.87498	509.58574	382.44113	306.15436	255.29651	11
22	2688.13004	1344.56866	896.71486	672.78797	538.43183	448.86107	Y	1413.65861	707.33294	471.89106	354.17011	283.53754	236.44917	10
23	2874.20935	1437.60831	958.74130	719.30779	575.64769	479.87429	W	1250.59528	625.80128	417.53661	313.40428	250.92488	209.27194	9
24	3064.28358	1532.64543	1022.09938	766.82635	613.66254	511.55333	W-Trp->Kynurenin	1064.51597	532.76162	355.51017	266.88445	213.70902	178.25873	8
25	3193.32617	1597.16672	1065.11357	799.08700	639.47106	533.06043	E	874.44174	437.72451	292.15210	219.36589	175.69417	146.57969	7
26	3340.39458	1670.70093	1114.13638	835.85410	668.88474	557.57183	F	745.39915	373.20321	249.13790	187.10524	149.88565	125.07259	6
27	3454.43751	1727.72239	1152.15069	864.36484	691.69332	576.57898	N	598.33074	299.66901	200.11510	150.33814	120.47197	100.56119	5
28	3553.50592	1777.25660	1185.17349	889.13194	711.50701	593.09038	V	484.28781	242.64754	162.10079	121.82741	97.66338	81.55403	4
29	3690.56484	1845.78606	1230.85980	923.39667	738.91879	615.93354	H	385.21939	193.11334	129.07798	97.06031	77.84970	65.04263	3
30	3777.59686	1889.30207	1259.87047	945.15467	756.32519	630.43887	S	248.16048	124.58388	83.39168	62.79558	50.43792	42.19948	2
31							K-Methyl	161.12845	81.06787	54.38100	41.03757	33.03151	27.69414	1

Sequence: EKMLSR, K2-Carboxyethyl (72.02113 Da)
Charge: +2, Monoisotopic m/z: 418.22076 Da (+0.02 mmu/+0.05 ppm), MH+: 835.43425 Da, RT: 43.7507 min,
Identified with: Sequest HT (v1.17); XCorr:0.80, Percolator q-Value:5.2e-3, Percolator PEP:3.1e-2, ptmRS: Best Site Probabilities:K2(Carboxyethyl): 100,
Fragment match tolerance used for search: 0.02 Da
Fragments used for search: $-H_2O$; y; $-NH_3$; y; b; b; $-H_2O$; y

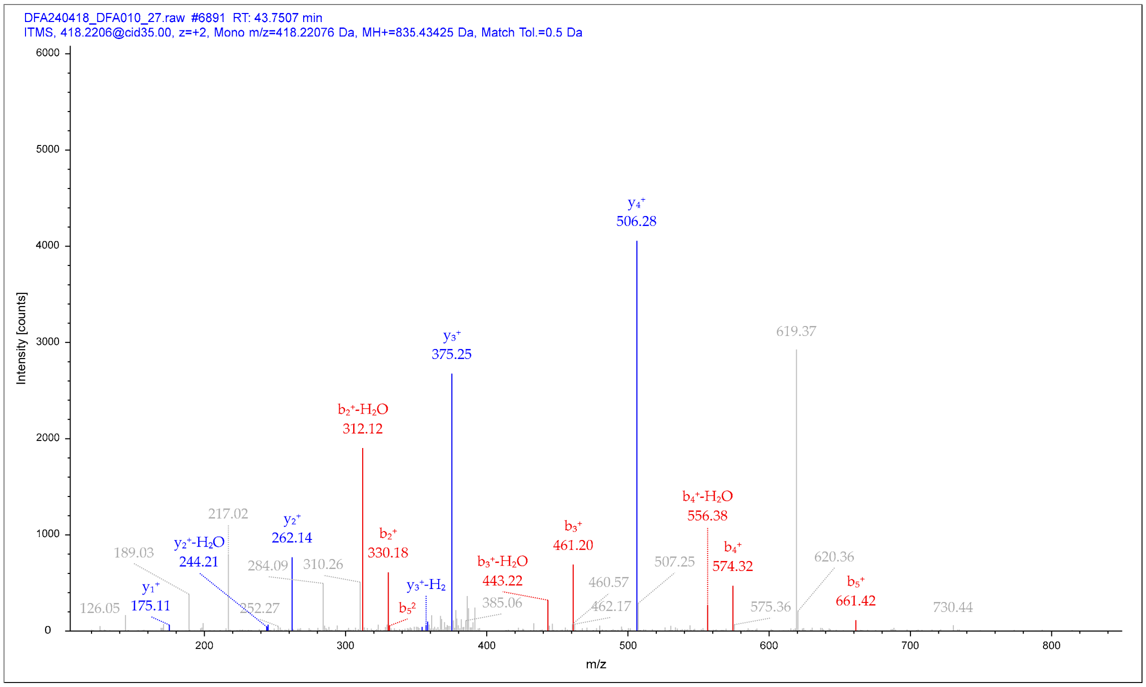

#1	b⁺	b²⁺	Seq.	y⁺	y²⁺	#2
1	130.04987	65.52857	E			6
2	330.16596	165.58662	K-Carboxyethyl	706.39162	353.69945	5
3	461.20645	231.10686	M	506.27553	253.64140	4
4	574.29051	287.64889	L	375.23504	188.12116	3
5	661.32254	331.16491	S	262.15098	131.57913	2
6			R	175.11895	88.06311	1

Sequence: MGAGGVLEYPWSK, W11-Trp->Oxolactone (13.97927 Da), M1-Dioxidation (31.98983 Da)
Charge: +2, Monoisotopic m/z: 720.82727 Da (+0.44 mmu/+0.61 ppm), MH+: 1440.64726 Da, RT: 44.0806 min,
Identified with: Sequest HT (v1.17); XCorr:0.71, Percolator q-Value:2.9e-3, Percolator PEP:3.0e-2, ptmRS: Best Site Probabilities:M1(Dioxidation): 100,
Fragment match tolerance used for search: 0.02 Da
Fragments used for search: -H_2O; y; -NH_3; y; b; b; -H_2O; y

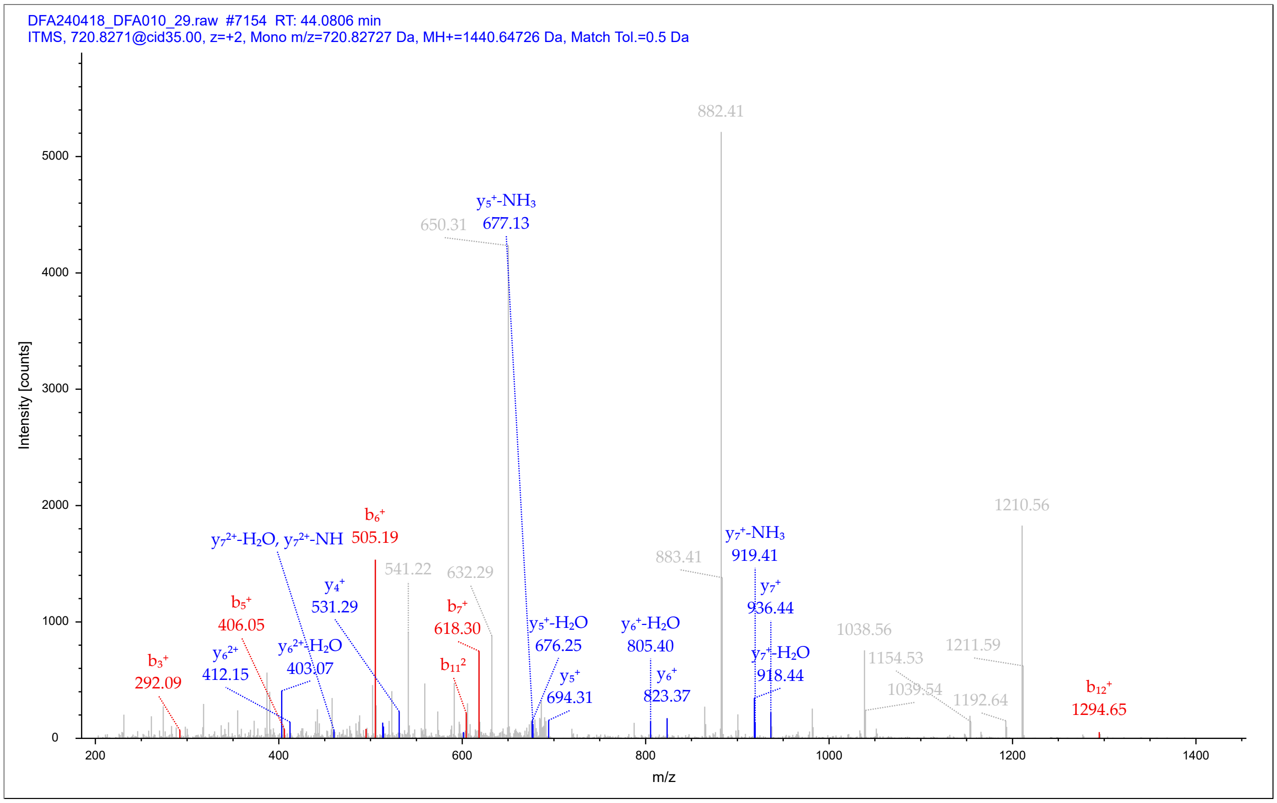

#1	b⁺	b²⁺	Seq.	y⁺	y²⁺	#2
1	164.03759	82.52243	M-Dioxidation			13
2	221.05905	111.03317	G	1277.61608	639.31168	12
3	292.09617	146.55172	A	1220.59461	610.80095	11
4	349.11763	175.06245	G	1149.55750	575.28239	10
5	406.13910	203.57319	G	1092.53604	546.77166	9
6	505.20751	253.10739	V	1035.51457	518.26093	8
7	618.29157	309.64942	L	936.44616	468.72672	7
8	747.33417	374.17072	E	823.36210	412.18469	6
9	910.39749	455.70239	Y	694.31950	347.66339	5
10	1007.45026	504.22877	P	531.25617	266.13173	4
11	1207.50884	604.25806	W-Trp->Oxo lactone	434.20341	217.60534	3
12	1294.54086	647.77407	S	234.14483	117.57605	2
13			K	147.11280	74.06004	1

Sequence: TEDSALYYCAK, C9-Carbamidomethyl (57.02146 Da), K11-Formyl (27.99492 Da)
Charge: +2, Monoisotopic m/z: 674.79352 Da (+3.6 mmu/+5.34 ppm), MH+: 1348.57976 Da, RT: 47.5396 min,
Identified with: Sequest HT (v1.17); XCorr:0.66, Percolator q-Value:5.1e-3, Percolator PEP:3.2e-2,
Fragment match tolerance used for search: 0.02 Da
Fragments used for search: -H_2O; y; b; b; -H_2O; y

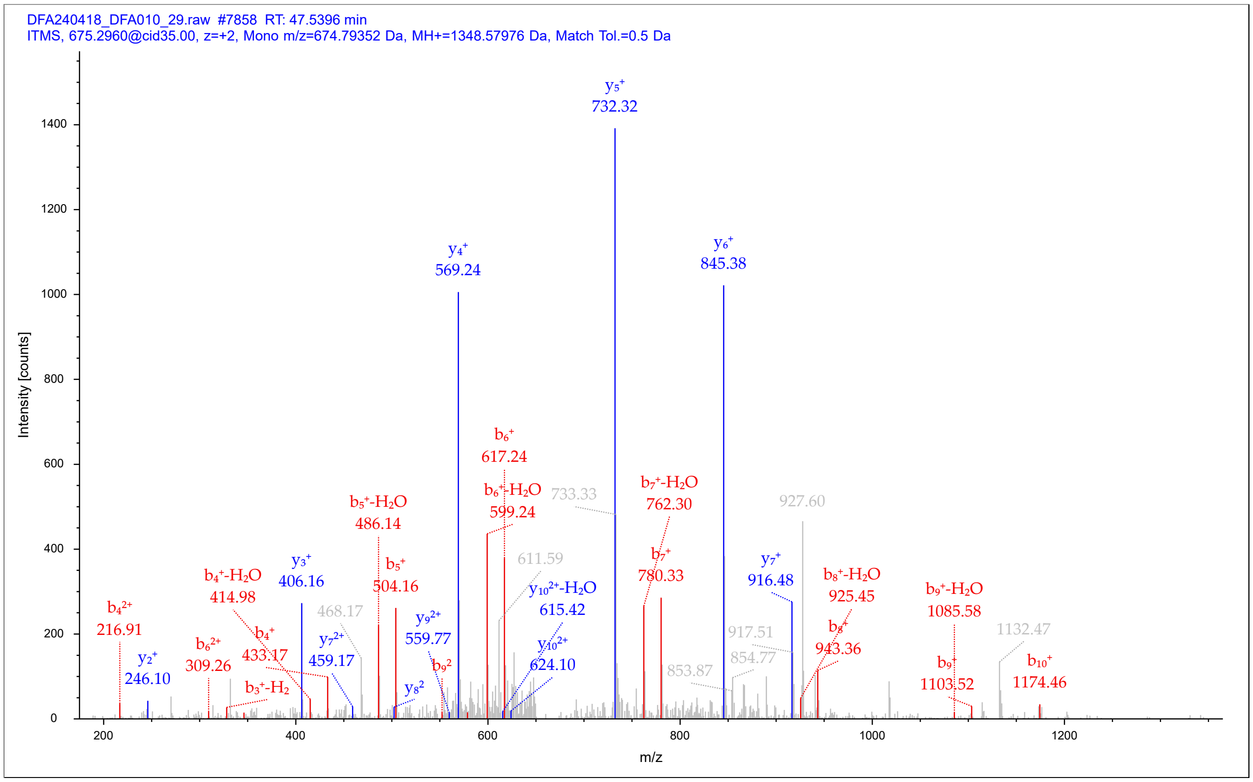

b⁺	b²⁺	Seq.	y⁺	y²⁺	#2
102.05496	51.53112	T			11
231.09755	116.05241	E	1247.52488	624.26608	10
346.12449	173.56588	D	1118.48229	559.74478	9
433.15652	217.08190	S	1003.45534	502.23131	8
504.19363	252.60045	A	916.42332	458.71530	7
617.27770	309.14249	L	845.38620	423.19674	6
780.34103	390.67415	Y	732.30214	366.65471	5
943.40435	472.20582	Y	569.23881	285.12304	4
1103.43500	552.22114	C-Carbamido methyl	406.17548	203.59138	3
1174.47212	587.73970	A	246.14483	123.57605	2
		K-Formyl	175.10772	88.05750	1

75

Sequence: LSDAFLGELAEK, F5-Oxidation (15.99492 Da), K12-HydroxymethylOP (108.02113 Da)
Charge: +3, Monoisotopic m/z: 472.90088 Da (-0.41 mmu/-0.86 ppm), MH+: 1416.68808 Da, RT: 49.4765 min,
Identified with: Sequest HT (v1.17); XCorr:0.42, Percolator q-Value:2.0e-3, Percolator PEP:1.1e-2,
Fragment match tolerance used for search: 0.02 Da
Fragments used for search: -H_2O; y; b; b; -H_2O; y

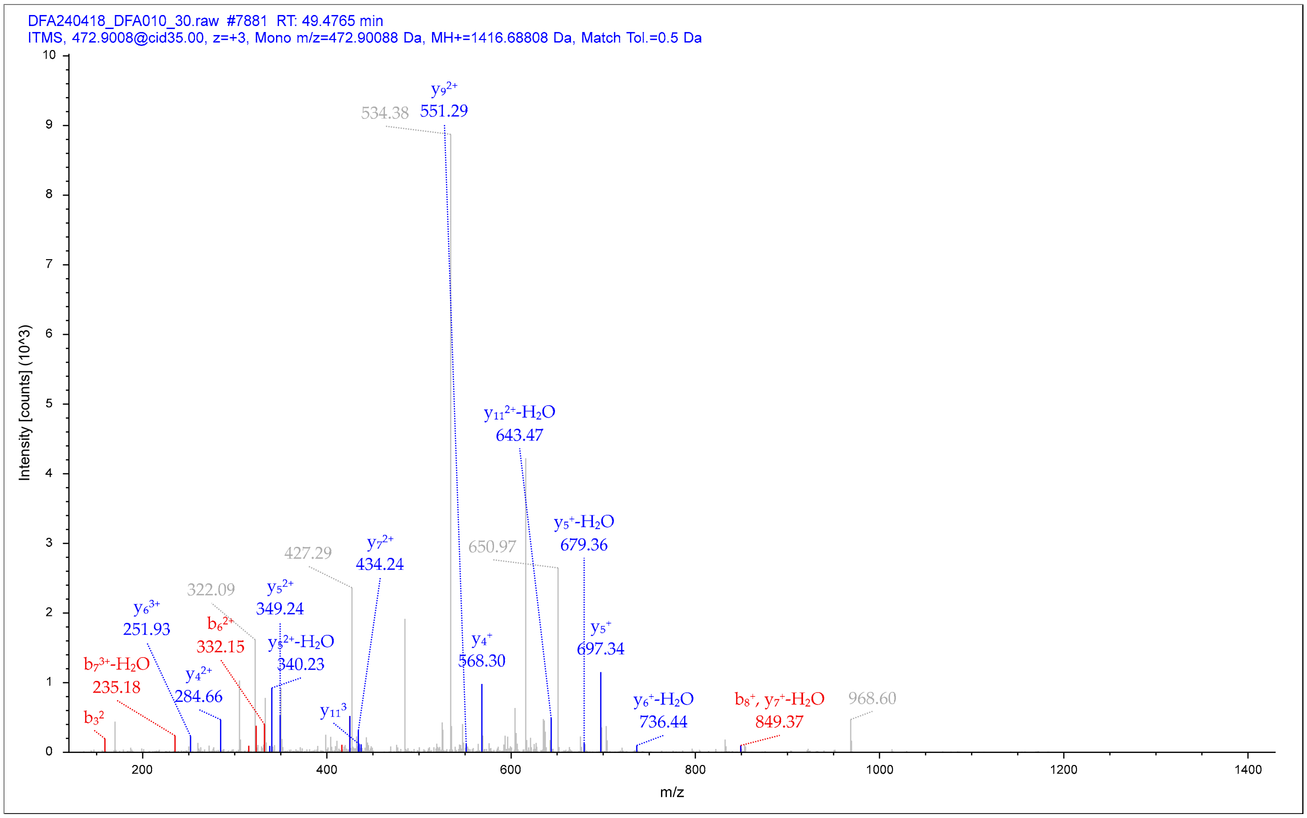

#1	b⁺	b²⁺	b³⁺	Seq.	y⁺	y²⁺	y³⁺	#2
1	114.09134	57.54931	38.70196	L				12
2	201.12337	101.06532	67.71264	S	1303.60524	652.30626	435.20660	11
3	316.15031	158.57879	106.05496	D	1216.57321	608.79024	406.19592	10
4	387.18743	194.09735	129.73399	A	1101.54627	551.27677	367.85361	9
5	550.25075	275.62902	184.08844	F-Oxidation	1030.50915	515.75822	344.17457	8
6	663.33482	332.17105	221.78312	L	867.44582	434.22655	289.82013	7
7	720.35628	360.68178	240.79028	G	754.36176	377.68452	252.12544	6
8	849.39888	425.20308	283.80448	E	697.34030	349.17379	233.11828	5
9	962.48294	481.74511	321.49916	L	568.29770	284.65249	190.10409	4
10	1033.52005	517.26366	345.17820	A	455.21364	228.11046	152.40940	3
11	1162.56265	581.78496	388.19240	E	384.17653	192.59190	128.73036	2
12				K-Hydroxy methylOP	255.13393	128.07060	85.71616	1

Sequence: AADDTWEPFASGK, W6-Trp->Hydroxykynurenin (19.98983 Da)
Charge: +2, Monoisotopic m/z: 707.80994 Da (+0.24 mmu/+0.34 ppm), MH+: 1414.61260 Da, RT: 56.7085 min,
Identified with: Sequest HT (v1.17); XCorr:0.71, Percolator q-Value:6.0e-3, Percolator PEP:1.9e-2,
Fragment match tolerance used for search: 0.02 Da
Fragments used for search: -H_2O; y; -NH_3; y; b; b; -H_2O; y

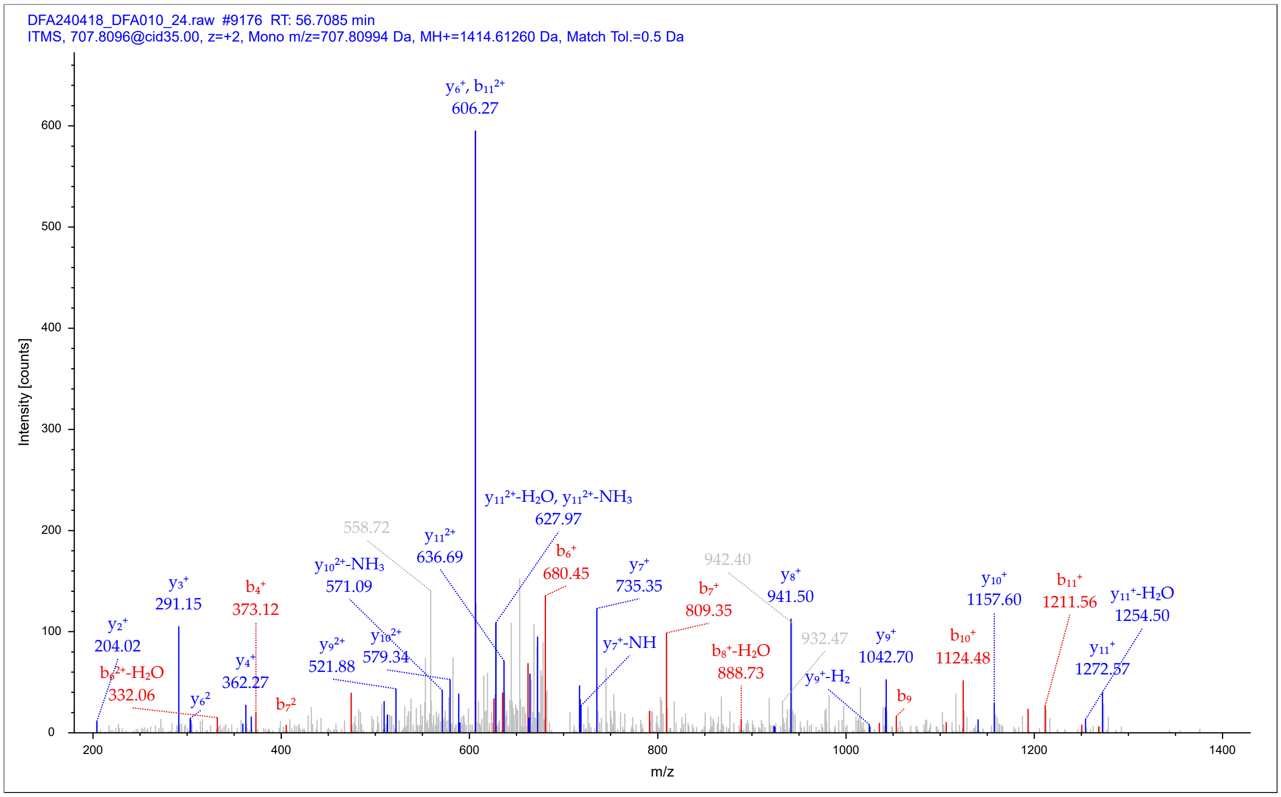

#1	b+	b2+	Seq.	y+	y2+	#2
1	72.04439	36.52583	A			13
2	143.08150	72.04439	A	1343.57500	672.29114	12
3	258.10845	129.55786	D	1272.53789	636.77258	11
4	373.13539	187.07133	D	1157.51094	579.25911	10
5	474.18307	237.59517	T	1042.48400	521.74564	9
6	680.25221	340.62974	W-Trp->Hydroxy kynurenin	941.43632	471.22180	8
7	809.29480	405.15104	E	735.36718	368.18723	7
8	906.34757	453.67742	P	606.32459	303.66593	6
9	1053.41598	527.21163	F	509.27182	255.13955	5
10	1124.45309	562.73019	A	362.20341	181.60534	4
11	1211.48512	606.24620	S	291.16630	146.08679	3
12	1268.50659	634.75693	G	204.13427	102.57077	2
13			K	147.11280	74.06004	1

41

Sequence: GLMSFVEDHSNEFFTDLKLR, K18-Pyrazine (46.99344 Da)
Charge: +3, Monoisotopic m/z: 811.38708 Da (-0.37 mmu/-0.45 ppm), MH+: 2432.14670 Da, RT: 58.2589 min,
Identified with: Sequest HT (v1.17); XCorr:0.77, Percolator q-Value:5.0e-3, Percolator PEP:1.2e-2,
Fragment match tolerance used for search: 0.02 Da
Fragments used for search: $-H_2O$; y; $-NH_3$; y; b; b; $-H_2O$; b; $-NH_3$; y

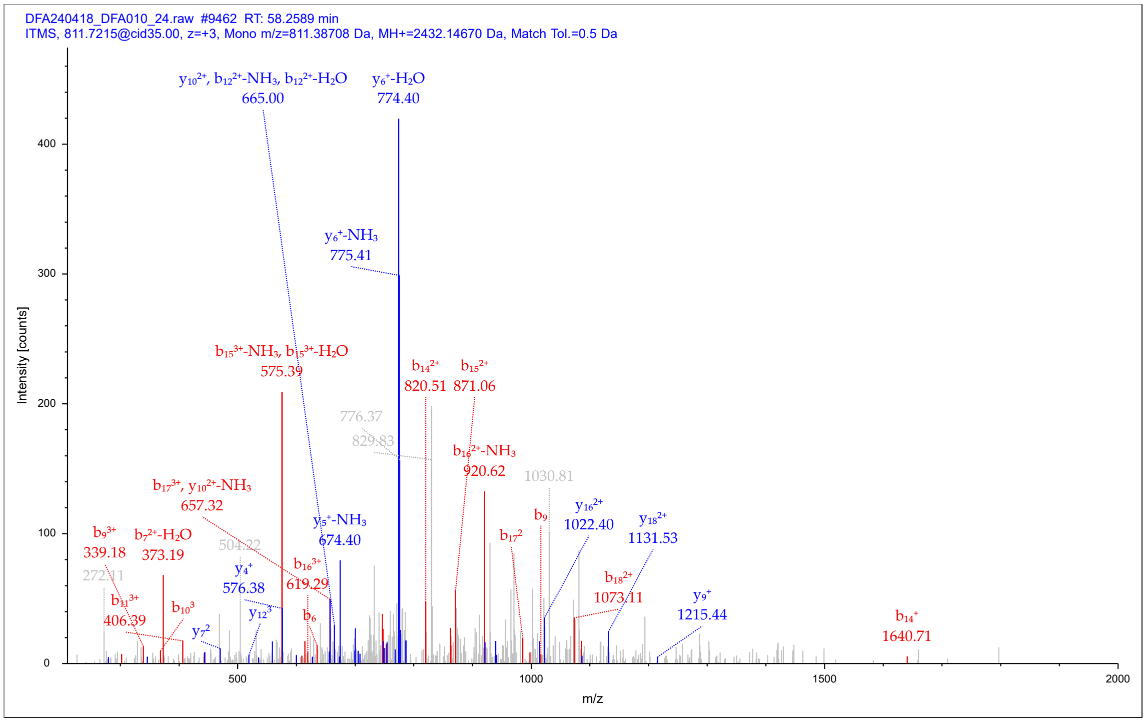

b⁺	b²⁺	b³⁺	Seq.	y⁺	y²⁺	y³⁺	#2
58.02874	29.51801	20.01443	G				20
171.11280	86.06004	57.70912	L	2375.12633	1188.06680	792.38030	19
302.15329	151.58028	101.38928	M	2262.04227	1131.52477	754.68561	18
389.18532	195.09630	130.39996	S	2131.00178	1066.00453	711.00545	17
536.25373	268.63050	179.42276	F	2043.96976	1022.48852	681.99477	16
635.32214	318.16471	212.44557	V	1896.90134	948.95431	632.97197	15
764.36474	382.68601	255.45976	E	1797.83293	899.42010	599.94916	14
879.39168	440.19948	293.80208	D	1668.79034	834.89881	556.93496	13
1016.45059	508.72893	339.48838	H	1553.76339	777.38533	518.59265	12
1103.48262	552.24495	368.49906	S	1416.70448	708.85588	472.90634	11
1217.52555	609.26641	406.51337	N	1329.67245	665.33986	443.89567	10
1346.56814	673.78771	449.52756	E	1215.62953	608.31840	405.88136	9
1493.63656	747.32192	498.55037	F	1086.58693	543.79710	362.86716	8
1640.70497	820.85612	547.57317	F	939.51852	470.26290	313.84436	7
1741.75265	871.37996	581.25573	T	792.45010	396.72869	264.82155	6
1856.77959	928.89343	619.59805	D	691.40243	346.20485	231.13899	5
1969.86365	985.43547	657.29274	L	576.37548	288.69138	192.79668	4
2144.95206	1072.97967	715.65554	K-Pyrazine	463.29142	232.14935	155.10199	3
2258.03612	1129.52170	753.35022	L	288.20302	144.60515	96.73919	2
			R	175.11895	88.06311	59.04450	1

Sequence: IEHFMPLLVQR, M5-Oxidation (15.99492 Da), R11-Carboxyethyl (72.02113 Da)
Charge: +2, Monoisotopic m/z: 735.89270 Da (+0.39 mmu/+0.53 ppm), MH+: 1470.77812 Da, RT: 62.9311 min,
Identified with: Sequest HT (v1.17); XCorr:0.92, Percolator q-Value:4.8e-3, Percolator PEP:2.6e-2,
Fragment match tolerance used for search: 0.02 Da
Fragments used for search: -H_2O; y; -NH_3; y; b; b; -H_2O; b; -NH_3; y

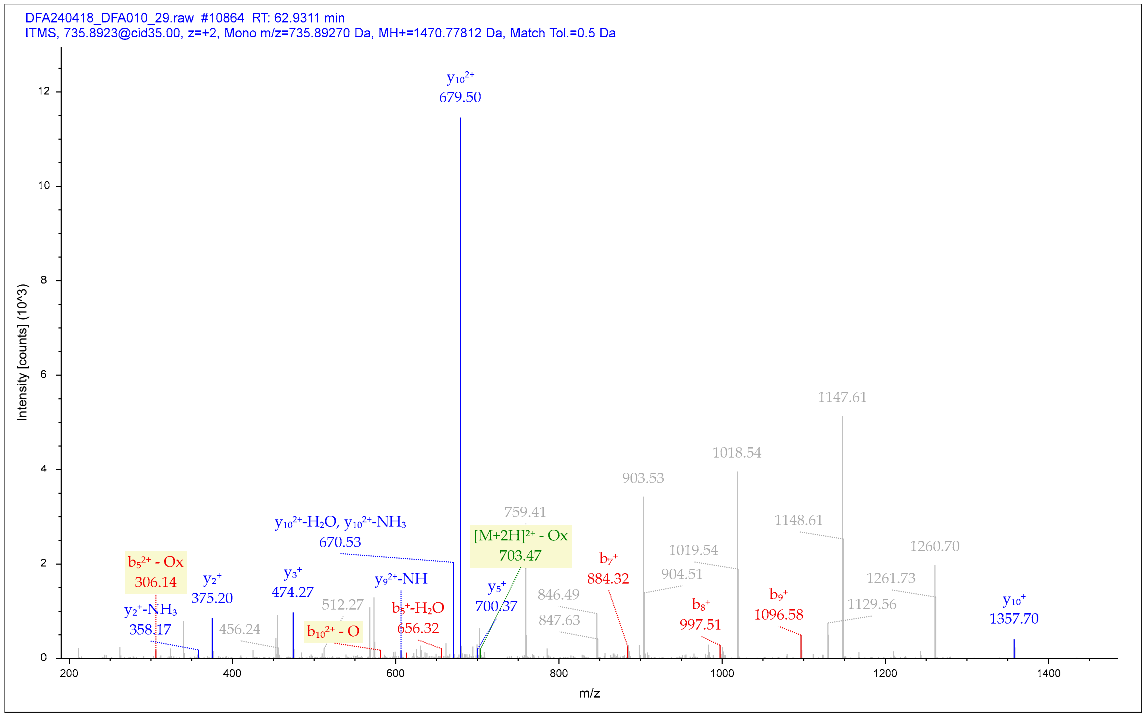

#1	b⁺	b²⁺	Seq.	y⁺	y²⁺	#2
1	114.09134	57.54931	I			11
2	243.13393	122.07061	E	1357.69328	679.35028	10
3	380.19285	190.60006	H	1228.65069	614.82898	9
4	527.26126	264.13427	F	1091.59178	546.29953	8
5	674.29666	337.65197	M-Oxidation	944.52336	472.76532	7
6	771.34942	386.17835	P	797.48796	399.24762	6
7	884.43349	442.72038	L	700.43520	350.72124	5
8	997.51755	499.26241	L	587.35114	294.17921	4
9	1096.58596	548.79662	V	474.26707	237.63717	3
10	1224.64454	612.82591	Q	375.19866	188.10297	2
11			R-Carboxy ethyl	247.14008	124.07368	1

Sequence: AVVEMSPWFLQQWR, M5-Oxidation (15.99492 Da), W8-Oxidation (15.99492 Da), W13-Trioxidation (47.98474 Da), R14-Triosyl (72.01840 Da)
Charge: +2, Monoisotopic m/z: 964.94501 Da (+0.37 mmu/+0.38 ppm), MH+: 1928.88274 Da, RT: 63.0070 min,
Identified with: Sequest HT (v1.17); XCorr:0.78, Percolator q-Value:6.2e-3, Percolator PEP:4.8e-2,
Fragment match tolerance used for search: 0.02 Da
Fragments used for search: -H_2O; y; -NH_3; y; b; b; -H_2O; b; -NH_3; y

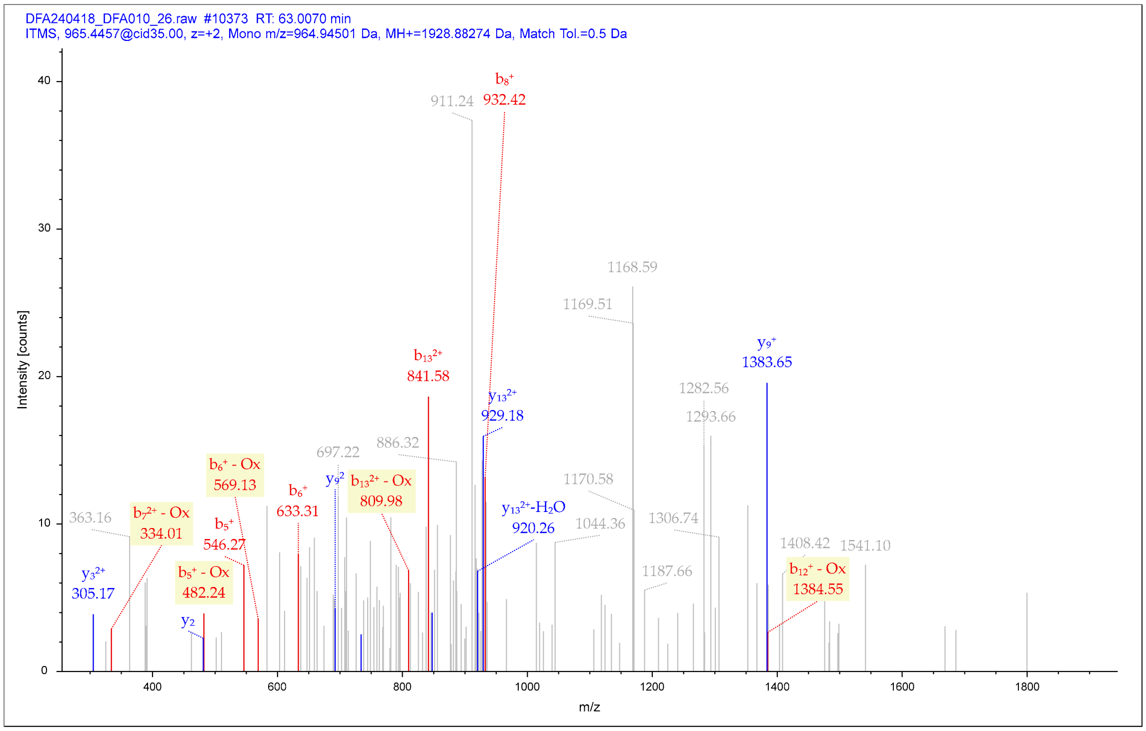

#1	b+	b2+	Seq.	y+	y2+	#2
1	72.04439	36.52583	A			14
2	171.11280	86.06004	V	1857.84488	929.42608	13
3	270.18122	135.59425	V	1758.77647	879.89187	12
4	399.22381	200.11554	E	1659.70805	830.35767	11
5	546.25921	273.63324	M-Oxidation	1530.66546	765.83637	10
6	633.29124	317.14926	S	1383.63006	692.31867	9
7	730.34400	365.67564	P	1296.59803	648.80266	8
8	932.41823	466.71275	W-Oxidation	1199.54527	600.27627	7
9	1079.48664	540.24696	F	997.47104	499.23916	6
10	1192.57071	596.78899	L	850.40263	425.70495	5
11	1320.62929	660.81828	Q	737.31856	369.16292	4
12	1448.68786	724.84757	Q	609.25999	305.13363	3
13	1682.75192	841.87960	W-Trioxidation	481.20141	241.10434	2
14			R-Triosyl	247.13735	124.07231	1

Sequence: TEEVLLFTDQTDDLAK, K16-Carboxymethyl (58.00548 Da)
Charge: +3, Monoisotopic m/z: 632.64203 Da (-0.17 mmu/-0.27 ppm), MH+: 1895.91153 Da, RT: 68.5400 min,
Identified with: Sequest HT (v1.17); XCorr:0.69, Percolator q-Value:3.4e-3, Percolator PEP:3.9e-2,
Fragment match tolerance used for search: 0.02 Da
Fragments used for search: -H_2O; y; -NH_3; y; b; b; -H_2O; b; -NH_3; y

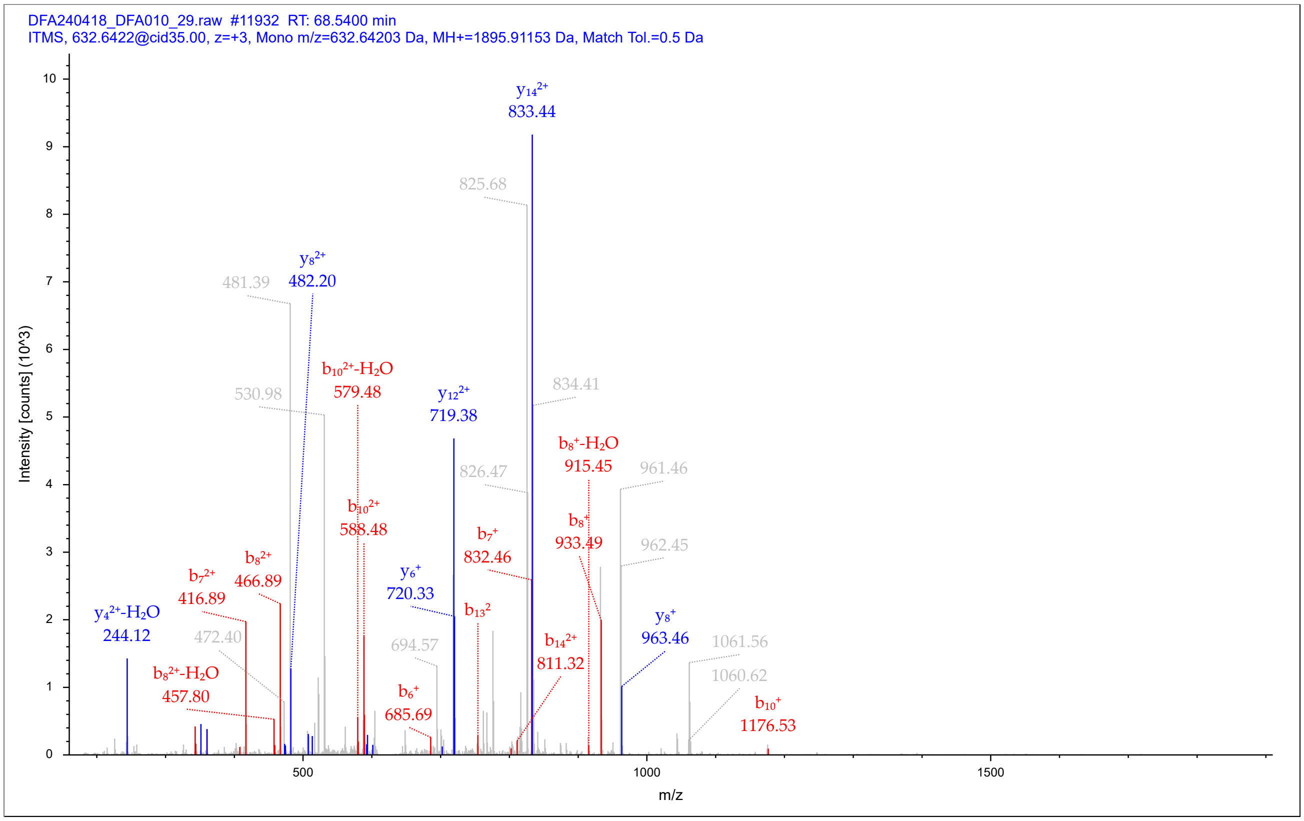

#1	b⁺	b²⁺	b³⁺	Seq.	y⁺	y²⁺	y³⁺	#2
1	102.05496	51.53112	34.68984	T				16
2	231.09755	116.05241	77.70403	E	1794.86437	897.93582	598.95964	15
3	360.14014	180.57371	120.71823	E	1665.82177	833.41452	555.94544	14
4	459.20855	230.10792	153.74104	V	1536.77918	768.89323	512.93124	13
5	572.29262	286.64995	191.43572	L	1437.71077	719.35902	479.90844	12
6	685.37668	343.19198	229.13041	L	1324.62670	662.81699	442.21375	11
7	832.44510	416.72619	278.15322	F	1211.54264	606.27496	404.51906	10
8	933.49278	467.25003	311.83578	T	1064.47422	532.74075	355.49626	9
9	1048.51972	524.76350	350.17809	D	963.42655	482.21691	321.81370	8
10	1176.57830	588.79279	392.86428	Q	848.39960	424.70344	283.47139	7
11	1277.62597	639.31663	426.54684	T	720.34103	360.67415	240.78519	6
12	1392.65292	696.83010	464.88916	D	619.29335	310.15031	207.10263	5
13	1507.67986	754.34357	503.23147	D	504.26640	252.63684	168.76032	4
14	1620.76392	810.88560	540.92616	L	389.23946	195.12337	130.41800	3
15	1691.80104	846.40416	564.60520	A	276.15540	138.58134	92.72332	2
16				K-Carboxy methyl	205.11828	103.06278	69.04428	1
