# Supplementary material for: Peptide-Bound Glycative, AGE and Oxidative Modifications as Biomarkers for the Diagnosis of Alzheimer’s Disease—A Feasibility Study
Source: Biomedicines. 2024 Sep 19;12(9):2127. doi: 10.3390/biomedicines12092127 (PMC11428617; doi:10.3390/biomedicines12092127)
Supplement: Supplementary file 1 [file biomedicines-12-02127-s001.zip › Figures S1&2.pdf]

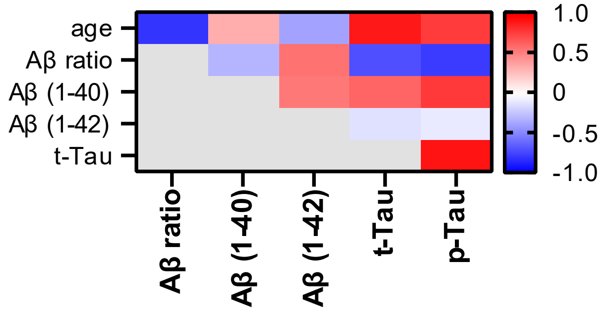

Figure S1. Spearman's rank correlation coefficients of biochemical core biomarkers and age. Correlations can assume values between -1 and 1. Negative correlations are colored in blue, positive correlations are colored in red.

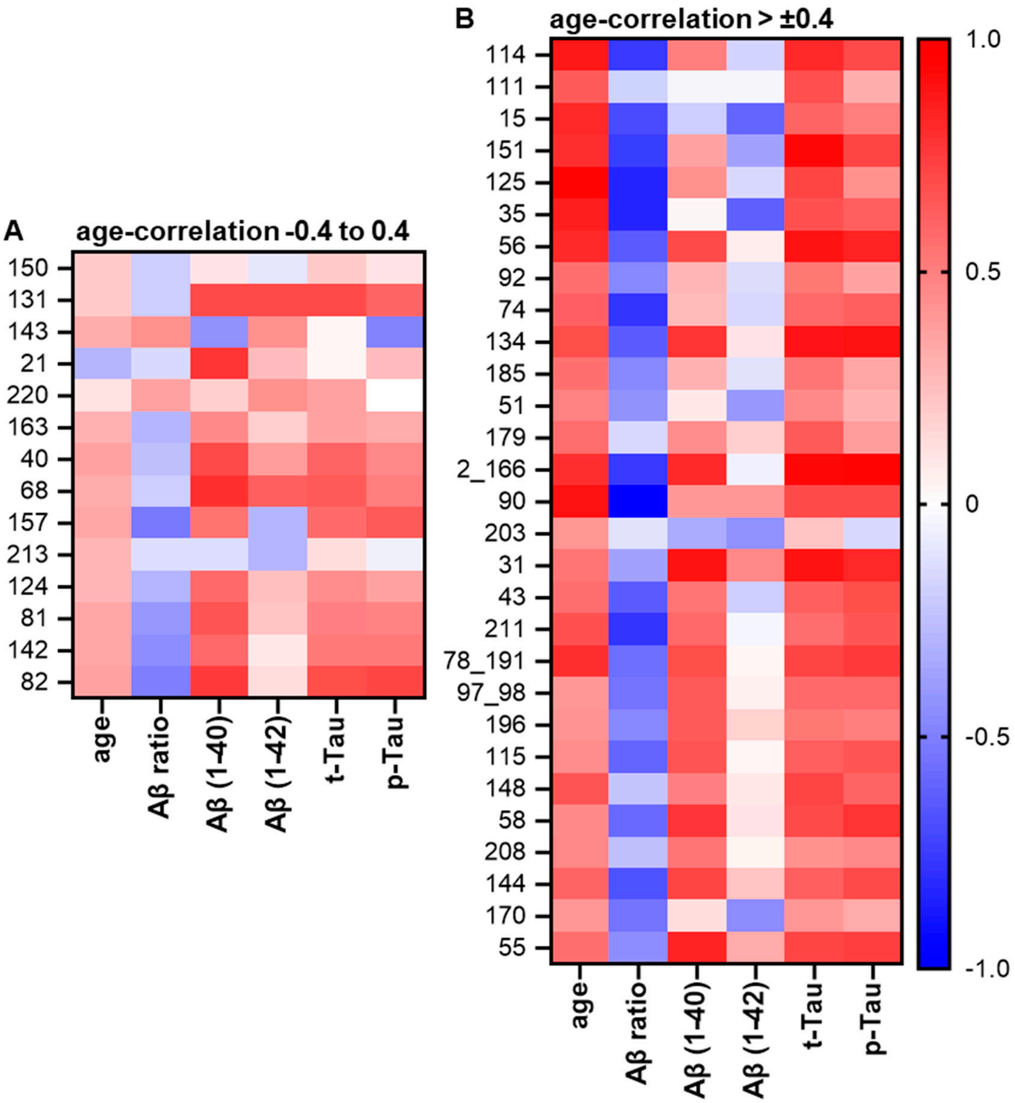

Figure S2. Spearman's rank correlation coefficients of biochemical core biomarkers and age with peptide signals from candidates with a ROC-AUC > 0.7 and **(A)** only very weak to weak (-0.4 to 0.4) correlation with age and **(B)** moderate to very strong correlation to age. Correlations can assume values between -1 and 1. Negative correlations are colored in blue, positive correlations are colored in red.
