# Supplementary material for: Peptide-Bound Glycative, AGE and Oxidative Modifications as Biomarkers for the Diagnosis of Alzheimer’s Disease—A Feasibility Study
Source: Biomedicines. 2024 Sep 19;12(9):2127. doi: 10.3390/biomedicines12092127 (PMC11428617; doi:10.3390/biomedicines12092127)
Supplement: Supplementary file 1 [file biomedicines-12-02127-s001.zip › Table S1.pdf]

Database search settings (Proteome Discoverer 2.2)		
Search Engine	SEQUEST	
Protease	Trypsin	
Missed cleavage sites	2	
MS (peptide) tolerance	10 ppm	
MS/MS tolerance	0.02 *m/z*	
FDR	0.01	
**Modification (Abbrev.)**	**Mass increment (Da)**	**amino acids**
Carbamidomethyl (cam)	57.021	C [a]
Oxidation (ox)	15.995	C, M, W, F, Y
Dioxidation (2ox)	31.990	C, M, W, F, Y
Trioxidation (3ox)	47.985	C, W, F, Y
Tryp->kynurenine (kyn)	3.995	W
Tryp->oxolactone (oxo)	13.9792	W
Tryp->hydroxykynurenine (h-kyn)	19.990	W
Argpyrimidine (ArgPyr)	80.026	R
Carboxymethyl (cm)	58.01	R, K
Glarg	39.995	R
MGH	54.011	R
Tetrahydropyrimidine	144.042	R
Pyrraline (pyr)	108.021	K
GLAP	109.029	K
Carboxyethyl (ce)	72.0211	R, K
Formyl	27.9955	K
Acetyl	42.0112	K
Glycerinyl (glyc)	88.0167	K
Glyoxal-Imine (gi)	39.9955	K
C4-amide (C4)	118.0272	K
C5-amide (C5)	148.0378	K
Fructosyl (fruc)	162.0535	R, K
Pyrazine (pyra)	46.9934	K
3-Deoxyglucosone intermediate 2 (3dg-i2)	126.0317	K
Tetraethyl	102.018	R, K
Methyl	14.016	R, K
Lederers pentosone	114.032	K
2-Fructosyl	324.106	R
Triosyl	72.018	R, K
Ethnalyl	42.018	R, K
b-ketonium ion	96.021	K
Lederers glucosone	144.043	K
Pentosyl	132.040	R, K

Suppl. Table 1: Proteome Discoverer settings, used for Sequest database search and fixed and variable modifications. [a] fixed modification
